# [2]-Ladderanes as isosteres for *meta*-substituted aromatic rings and rigidified cyclohexanes

**DOI:** 10.1038/s41467-022-33827-3

**Published:** 2022-10-13

**Authors:** Rachel C. Epplin, Shashwati Paul, Loïc Herter, Christophe Salome, Erin N. Hancock, Jay F. Larrow, Erich W. Baum, David R. Dunstan, Carol Ginsburg-Moraff, Thomas C. Fessard, M. Kevin Brown

**Affiliations:** 1 Department of Chemistry, Indiana University, 800 E. Kirkwood Ave, Bloomington, IN 47405 USA; 2 SpiroChem AG, Mattenstrasse 22, 4058 Basel, Switzerland; 3 Novartis Institutes for BioMedical Research, 250 Massachusetts Ave, Cambridge, MA 02139 USA; 4 Present Address: Corteva Agriscience, 9330 Zionsville Rd, Indianapolis, IN 46268 USA; 5 Present Address: Relay Therapeutics, 399 Binney St., Cambridge, MA 02139 USA

**Keywords:** Synthetic chemistry methodology, Lead optimization

## Abstract

Aromatic ring isosteres and rigidified saturated hydrocarbons are important motifs to enable drug discovery. Herein we disclose [2]-ladderanes as a class of *meta*-substituted aromatic ring isosteres and rigidified cyclohexanes. A straightforward synthesis of the building blocks is presented along with representative derivatization. Preliminary studies reveal that the [2]-ladderanes offer similar metabolic and physicochemical properties thus establishing this class of molecules as interesting motifs.

## Abstract (web summary)

The development of new classes of isosteres and building blocks is crucial to the advancement of medicinal chemistry programs. Here, the authors report the synthesis and development of ladderanes to act as replacements for meta-substituted aromatic rings and cyclohexanes.

## Introduction

**Fig. 1:**
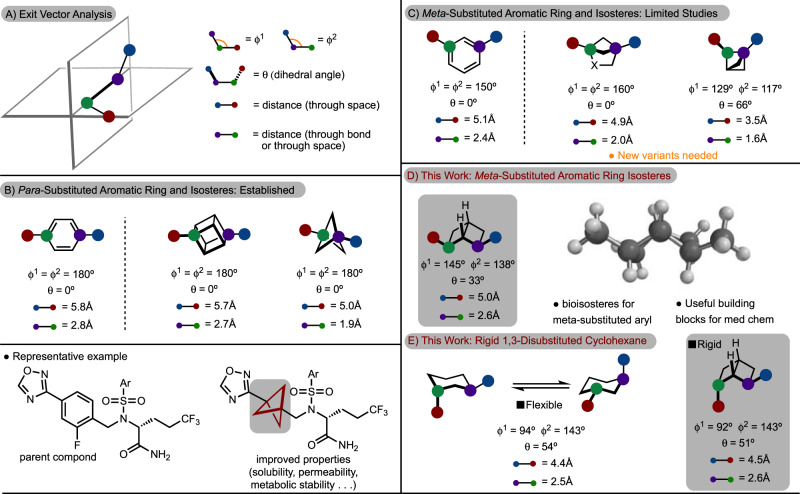
Background. **A** Exit vector analysis to establish the 3D positions of substituents relative to each other. **B** Known aromatic ring isosteres for *para*-substituted aromatic rings with a representative example. **C** Known aromatic ring isosteres for *meta*-substituted aromatic rings. **D** [2]-Ladderane as isosteres for aromatic rings **E** [2]-Ladderane as rigidified cyclohexanes.

The introduction of building blocks is an important direction in modern medicinal chemistry. One key focus is the development of isosteres to manipulate the physicochemical properties of molecules^1,2^. Classical examples include replacement of C–H bonds with C–F or C–D bonds, typically to prolong in vivo half-life^1^. More recently, non-classical isosteres have emerged, such as exchange of carbonyls for oxetane rings^3–5^. Along these lines, a recent surge in interest has been directed toward identifying isosteres for substituted aromatic rings^6,7^. Several approaches have been developed to identify probable isosteres including, but not limited to, the use of exit vector analysis to allow for comparison of chemical space covered by disubstituted scaffolds (Fig. 1A)^8^. In particular, cubane and [1.1.1]-bicyclopentanes have shown promise as replacements for *para*-substituted aromatic rings due to the similar positioning of substituents as shown in Fig. 1B^9–16^. This was exemplified in a 2012 report in which the [1.1.1]-bicyclopentane showed improved properties compared to the parent structure (Fig. 1B)^11^. As a result, significant effort has been directed toward the synthesis and derivatization of cubane and [1.1.1]-bicyclopentanes^17–20^.

Isostere replacement for *meta*-substituted aromatic rings has primarily seen development along the lines of the 1,4-disubstituted-[2.1.1]-bicyclohexane (Fig. 1C)^17^. Despite successful exemplification of this isostere, the angle between substituents (ϕ), and hence exit vectors, are deviated from the parent compound. It should be noted that recent efforts have shown that oxa-^21^ and aza-variants^22^ are useful in increasing water solubility of this scaffold. In addition, a recent study described the introduction of 1,2-disubstituted-[1.1.1]-bicyclopentanes to serve as *meta*- and *ortho*-substituted aromatic ring replacements (Fig. 1C)^23^. While this study is a notable advance, the positioning of substituents more closely aligns with an *ortho*-substituted aromatic ring. Other molecules have been proposed to be *meta*-substituted aromatic ring isosteres, such as 1,3-substituted cubanes, however, synthetic routes have not been established^17,24^.

Our lab^25–27^ and others^28–30^ have been interested in the chemistry of strained ring systems, particularly cyclobutanes. Within this program of research, we developed routes toward the synthesis of the highly unusual ladderane lipids^25–27^. In the course of these studies, we became interested in the use of [2.2.0]-bicyclohexanes ([2]-ladderanes) as building blocks for discovery chemistry. Here, we report a straightforward synthesis of a *cis-* and *trans*-2,6-disubstituted [2]-ladderane as replacements for *meta*-substituted aromatic rings and cyclohexanes, representative derivatizations, and preliminary ADME comparisons.

## Results and discussion

During our investigation into the ladderanes, we made the observation that the positioning of a *cis*-2,6-disubstituted [2]-ladderane has a similar arrangement of the substituents relative to a *meta*-substituted aromatic ring (Fig. 1D). This scaffold not only constitutes a hitherto unknown isostere, but also ventures into realms of chemical space that have remained unexplored, to our knowledge, in existing patents. In addition, a 2,6-disubstituted [2]-ladderane can also act as a rigidified 1,3-disubstituted cyclohexane (Fig. 1E). Rigidification of lead compounds is an established strategy in medicinal chemistry^31^. Anti-1,3-disubstituted cyclohexane are conformationally flexible, however, the analogous [2]-ladderane structure is rigid and is thus of additional value.

**Fig. 2:**
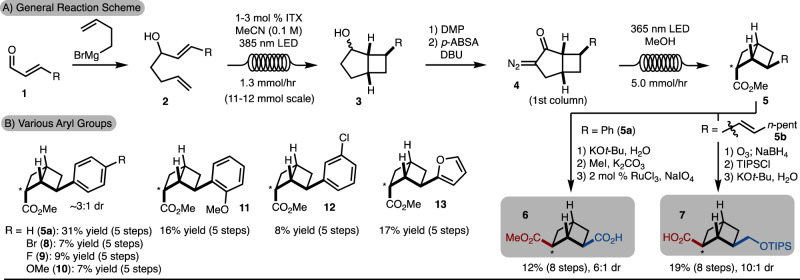
Synthetic strategy. **A** Synthesis of [2]-ladderane building blocks. **B** Other examples that can be prepared by the outlined route. ITX 2-*i*-Prthioxanthone, DMP Dess Martin periodinane, LED light emitting diode, p-ABSA asdfasdfasd p-acetamidosulfonylazide, TIPS Triisopropylsilyl.

After exploration of several strategies, a robust and scalable route to the 2,6-disubstituted [2]-ladderane was developed (Fig. 2A). The general route begins with addition of homoallyl Grignard to either cinnamaldehyde and its derivatives or (*E*,*E*)-2,4-decadienal (all common and commercially available reagents). Photosensitized [2 + 2] cycloaddition provided access to cyclobutane **3**^32,33^. While this reaction could be conducted in batch (see SI, General Procedure C for batch set-up), scale up of the process was more easily performed in a flow photochemical reactor. Oxidation of the secondary alcohol with DMP followed by diazoketone synthesis provided access to **4**. This is the first chromatography performed in the sequence. Irradiation of the diazoketone with 365 nm LEDs in the presence of MeOH provided access to ester **5** with the endo-diastereomer predominating. At this stage, two final elaborations to useful building blocks were established. Epimerization of **5** to the exo-diastereomer when R = Ph followed by esterification and exhaustive oxidation led to synthesis of ester/acid **6**^34^. When R = heptene, oxidative cleavage followed by silyl protection and epimerization allowed for the synthesis of acid/silyl ether building block **7**. Both routes could easily be conducted to provide hundreds of milligrams of **6** and **7**. At this stage of development, the current bottleneck is the conversion of **2** to **3** with our current commercial photochemical flow setup due to challenges with lamp cooling over extended reaction times. Finally, various aryl groups were tolerated and allowed for synthesis of **8**–**13** (Fig. 2B).

**Fig. 3:**
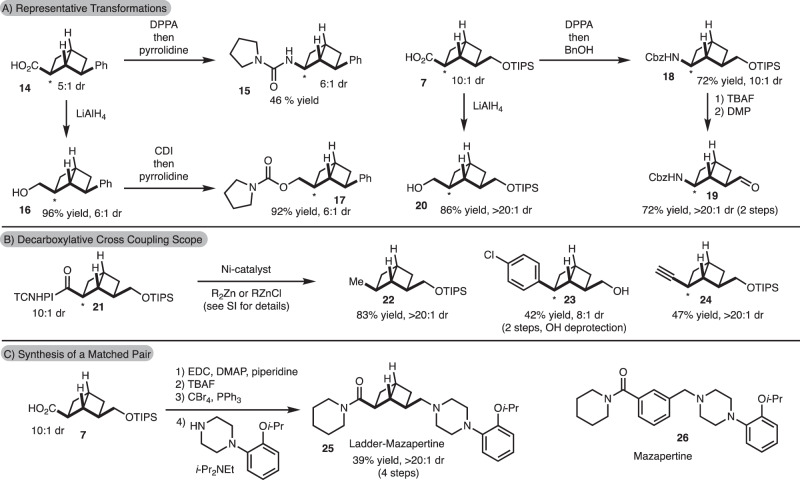
Derivatization of [2]-ladderane building blocks. **A** Transformation of the ladderane building block to other structures of relevance in medicinal chemistry. **B** Ni-catalyzed cross-coupling of redox-active esters. **C** Synthesis of ladder-mazapertine. DPPA diphenylphosphoryl azide, CDI carbonyl diimidazole, TBAF tetrabutylammonium fluoride, DMP Dess Martin periodinane, EDC 1-ethyl-3-(3-dimethylaminopropyl)carbodiimide, DMAP 4-dimethylaminopyridine.

With these building blocks in hand, a variety of derivatizations were carried out to demonstrate synthetic utility (Fig. 3A). It is important to note that the yields shown are reported for the diastereomeric mixture that was isolated. Curtius rearrangement of carboxylic acid **14** induced by DPPA allowed for synthesis of urea **15**. In addition, LiAlH_4_ reduction of **14** and carbamate formation led to **17**. Carboxylic acid **7** could also undergo Curtius rearrangement to generate **18**. Further elaboration by silyl deprotection and oxidation allowed for the synthesis of **19**. In addition, LiAlH_4_ reduction of **7** led to generation of a mono-protected diol **20**.

Due to the presence of a carboxylic acid moiety, decarboxylative cross-coupling reactions were explored^35,36^. In the case of redox-active esters derived from [2]-ladderane building blocks that incorporate ester (derived from **6**) or Ph substituents (derived from **14**), ring opening was observed in addition to desired product. However, in the case of the redox-active ester **21** derived from acid/silyl ether building block **7**, cross-coupling was effective (Fig. 3B). In addition, due to the rigid bicyclic nature of the [2]-ladderane, the cross-coupling proceeded with good diastereoselectivity for incorporation of the substituent on the exo-face. The incorporation of alkyl (**22**)^37^, aryl (**23**)^15^ and alkynyl (**24**)^38^ units could all be achieved. Finally, as illustrated by the reactions shown in Fig. 3, as well as additional control studies, the [2]-ladderanes are generally stable to acidic (see the SI stability experiments section), basic (see the SI stability experiments section), reducing (see **7**–**20**, Fig. 3A), and oxidizing conditions (see **5**–**6** and **7**, Fig. 2A).

To demonstrate how these building blocks could be incorporated into discovery chemistry programs, we targeted the [2]-ladderane matched pair of mazapertine (**26**) (Fig. 3C)^39^. Simple amide bond formation, deprotection, and substitution with a piperazine allowed for synthesis of ladderane-mazapertine (**25**) in 39% yield over 4 steps from **7**. It is important to note that the present study does not aim to specifically test a biological hypothesis, but rather demonstrate that a [2]-ladderane can serve as an alternative building block to an aromatic ring that can be incorporated to many ongoing medicinal chemistry programs around the world.

**Fig. 4:**
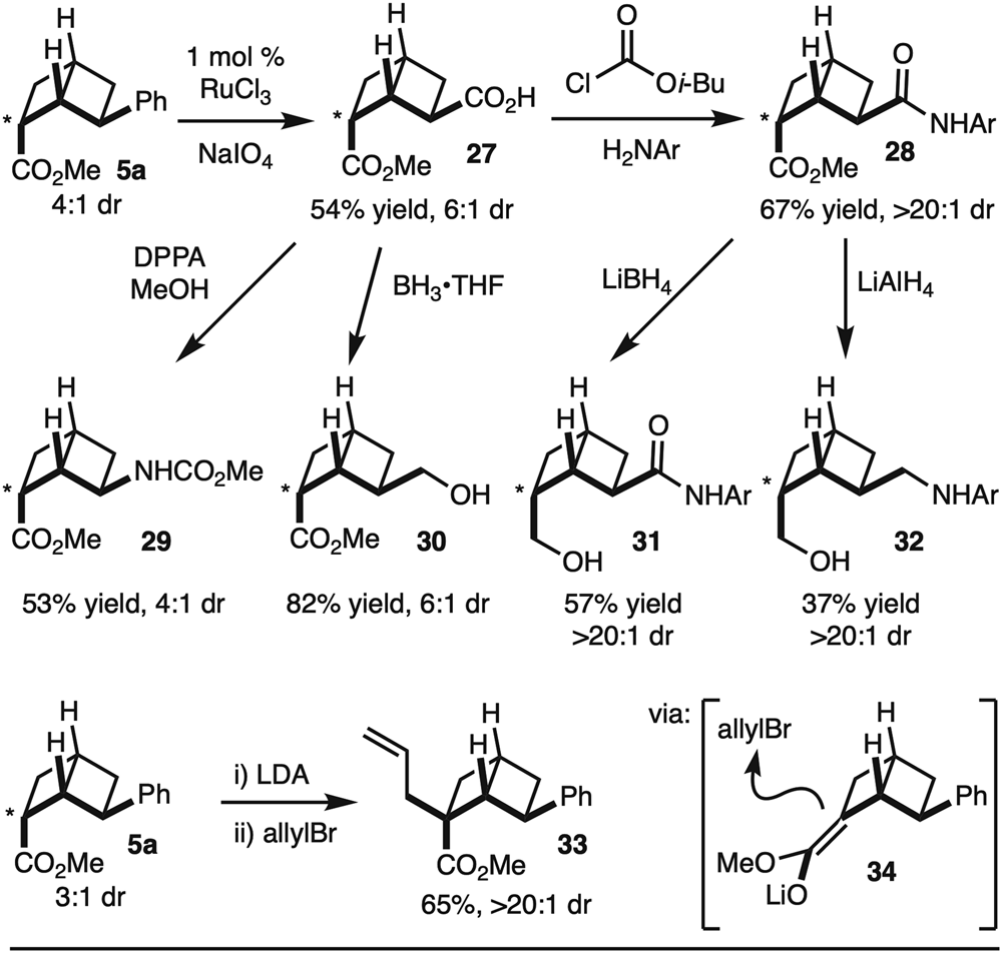
[2]-Ladderane as rigidified 1,3-cyclohexane. The ladderane compounds can be converted via standard chemical reactions to functional groups common to drug like molecules. DPPA dipenylphosphoryl azide, LDA lithium diisopropylamide.

Finally, as noted in the synthesis of [2]-ladderane building blocks, the Wolff rearrangement gives rise to the anti-isomer as the major product. When comparing the exit vectors of this structure, it displays similarity to that of an anti-1,3-substituted cyclohexane (Fig. 2E). Due the flexibility of the anti-2,6-substituted cyclohexane, it was envisioned that the anti-[2]-ladderane isomer could act as a rigidified variant, as this is often favorable in medicinal chemistry^31^. In light of this, we carried out several representative derivatizations to demonstrate the utility of this scaffold (Fig. 4). For example, the [2]-ladderane **5** was easily oxidized to generate building block **27**. From this intermediate, amide bond formation, Curtius rearrangement, and chemoselective reduction all proceeded smoothly to generate **28**, **29**, and **30**, respectively. From intermediate **28** (isolated as a single observable diastereomer), depending on reduction conditions employed, **31** and **32** could be prepared. Finally, deprotonation of **5a** with LDA and trapping of the enolate with allylbromide (via **34**) resulted in the formation of **33** as a single observable isomer.

**Fig. 5:**
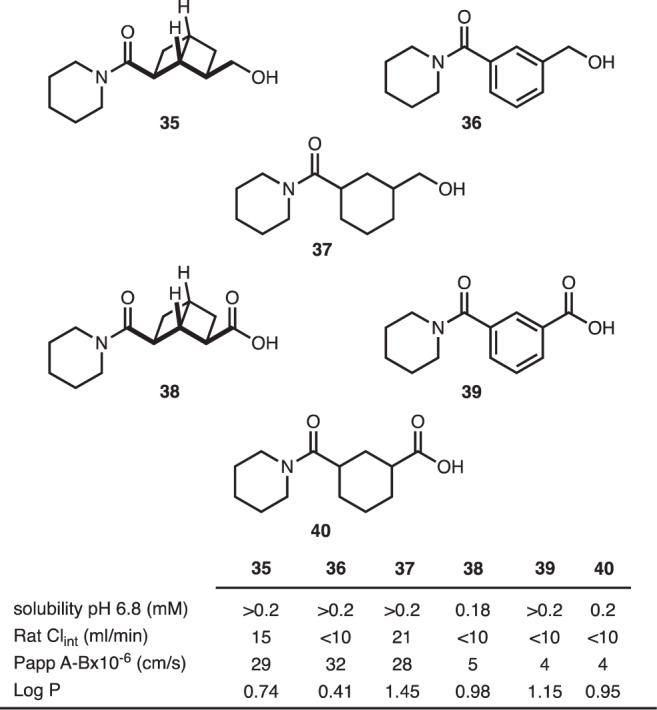
Preliminary comparisons to arenes/cyclohexanes. Evaluation of the ladderane, aryl, and cyclohexyl compounds shows little difference in the physicochemical properties.

The metabolic and physicochemical properties of the [2]-ladderanes compared to aryl/cyclohexyl matched pairs were also studied (compare **35** with **36**/**37** and **38** with **39**/**40**) (Fig. 5). These structures were chosen because they bear functional groups that are common in drug discovery, yet unlikely to pose any inherent risks. In addition, solubility, permeability, rat liver microsomal intrinsic clearance, and LogP were selected to be evaluated as these are common parameters modulated in drug discovery programs to improve the pharmacokinetic/pharmacodynamic and safety profiles of potential drug candidates. Though this is a small data set, the results of these studies demonstrate that [2]-ladderanes behave similarly to that of the aryl/cyclohexyl matched pairs. In fact, there is no appreciable difference in the parameters evaluated, suggesting that [2]-ladderanes should not negatively affect the metabolic or physicochemical properties of a compound when used as an isosteric replacement for a *meta*-substituted aromatic or cyclohexane ring.

In summary, [2]-ladderanes, has been introduced as a class of building blocks. These can be utilized as isosteres for *meta*-substituted aromatic rings or rigidified variants of anti-1,3-substituted cyclohexanes. Representative functionalizations provide access to an array of molecular diversity. These studies now establish these structures within the repertoire of building blocks to enable medicinal chemistry.

### Reporting summary

Further information on research design is available in the Nature Research Reporting Summary linked to this article.

## Supplementary information

Supplementary Information

Reporting Summary

## Data Availability

Experimental procedures, analytical data for all new compounds can be found in the Supplementary Information. The material is available free of charge.
