# Supplementary material for: [2]-Ladderanes as isosteres for meta-substituted aromatic rings and rigidified cyclohexanes
Source: Nat Commun. 2022 Oct 13;13:6056. doi: 10.1038/s41467-022-33827-3 (PMC9561113; doi:10.1038/s41467-022-33827-3)
Supplement: Supplementary file 1 — Supplementary Information [file 41467_2022_33827_MOESM1_ESM.pdf]

Supplementary Information

[2]-Ladderanes as Isosteres for *Meta*-Substituted Aromatic Rings and Rigidified Cyclohexanes

Rachel C. Epplin, Shashwati Paul, Loïc Herter, Christophe Salome, Erin N. Hancock, Jay F. Larrow, Erich W. Baum, David R. Dunstan, Carol Ginsburg-Moraff, Thomas C. Fessard, M. Kevin Brown**

Table of Contents

Supplementary Methods S3
General Information S3
Reagents and Catalysts S3
Synthesis of Reagents S5
General Procedures S9
Characterization Data of Intermediates and Bicyclo[2.2.0] Final Substrates S13
1. Precursors to Bicyclo[3.2.0]heptanes S13
2. [2+2] Cycloaddition to Bicyclo[3.2.0] Systems S16
3. DMP Oxidations S20
4. Formation of the α-Diazoketones S23
5. Bicyclo[2.2.0]hexane Formation S27
Functional Group Manipulations S31
1. Transformations of Cinnamaldehyde-Derived Products S31
2. Transformations of (*E,E*)-2,4-decadienal-Derived Products S36
3. Decarboxylative Cross Coupling Scope S40
4. Synthesis of Ladder-Mazapertine S43
Functional Group Manipulations of *endo*-[2]-Ladderane S46
Synthetic Procedures and Characterization Data of Compounds Submitted for ADME Data S51
ADME Procedures S54
Supplementary Discussion S56
Stability Experiments S55
Spectra S56
Supplementary References S207

Supplementary Methods

General Information

Infrared (IR) spectra were recorded on a Bruker Tensor II FT- IR Spectrometer, ν_{max} in cm^{-1}. Bands are characterized as broad (br), strong (s), medium (m), and weak (w). ^{1}H NMR spectra were recorded at room temperature unless otherwise noted on either a Varian 300 MHz, Varian I400 (400 MHz), Varian VXR400 (400 MHz), Varian I500 (500 MHz), a Varian I600 (600 MHz), or a Bruker Ascend™ 500 MHz (equipped with cryoprobe) spectrometer. Chemical shifts are reported in ppm from tetramethylsilane with the solvent resonance as the internal standard ($CHCl_3$: δ 7.26 ppm, C_3H_6O: δ 2.05 ppm, CH_3OH: δ 3.31 ppm, or C_6H_6: δ 7.16 ppm). Data are reported as follows: chemical shift, multiplicity (s = singlet, d = doublet, t = triplet, q = quartet, br = broad, m = multiplet), coupling constants (Hz), and integration. ^{13}C NMR spectra were recorded on a Varian 300 MHz (75 MHz), I400 (101 MHz), a Varian I500 (126 MHz), or a Bruker Ascend™ 500 MHz (126 MHz) spectrometer with complete proton decoupling. Chemical shifts are reported in ppm from tetramethylsilane with the solvent resonance as the internal standard ($CHCl_3$: δ 77.16 ppm, C_3H_6O: δ 29.84, CH_3OH: δ 49.00 ppm, or C_6H_6: δ 128.06 ppm). High-resolution mass spectrometry (HRMS) was performed on either an Agilent 7890B GC with Agilent 7250 QTOF and Gerstel MPS and TDS3 or a Thermo Scientific Finnigan LTQ Orbitrap XL Mass Spectrometer. Unless otherwise noted, all reactions have been carried out with distilled and degassed solvents under an atmosphere of dry N_2 in flame-dried glassware with standard vacuum-line. Tetrahydrofuran (THF) and *N,N*-Dimethylformamide (DMF) were purified under a positive pressure of dry argon by passage through two columns of activated alumina. Toluene was purified under a positive pressure of dry argon by passage through columns of activated alumina and Q5 (Grubbs apparatus). Photo flow reactions were performed using a Vapourtec Easy-Photochem E-series continuous flow reactor. The size of the reactor (tubing being directly irradiated) was 10 mL with tubing made of fluoropolymer and the Bore and Wall 1.3 x 0.15 mm. All work-up and purification procedures were carried out with reagent grade solvents (purchased from Sigma-Aldrich) in air. Standard column chromatography techniques using ZEOprep 60/40-63 µm silica gel or medium pressure liquid chromatography (MPLC) using a CombiFlash NextGen 100 with pre-packed silica cartridges were used for purification.

Reagents and Catalysts

***p*-Acetamidobenzenesulfonyl azide (*p*-ABSA)** was purchased from TCI and used as received.
Bathophenanthroline (BPhen) was purchased from CombiBlocks and used as received.
***rac*-BINAP** was purchased from CombiBlocks and handled in a nitrogen-atmosphere glovebox.
Biphenyl-3-carboxylic acid was purchased from Oakwood and used at received.
[1,1'-biphenyl]-3-ylmethanol was purchased from Ambeed and used as received.
Borane tetrahydrofuran complex solution, 1.0 M in THF (BH_3·THF) was purchased from Sigma Aldrich and used as received.
1-bromo-4-chlorobenzene was purchased from Sigma-Aldrich purified through a short plug of SiO_2.
4-bromo-1-butene was purchased from CombiBlocks and used as received.
2-bromophenol was purchased from Oakwood and used as received.
2-bromopropane was purchased from Combi Blocks and purified through a pad of silica.

Carbon tetrabromide (CBr_4) was purchased from Oakwood. It was purified by dissolving in CH_2Cl_2 and washing three times with brine. It was dried over $MgSO_4$, filtered, and concentrated under reduced pressure.

Carbon tetrachloride, anhydrous (CCl_4) was purchased from Sigma Aldrich and used as received.

1,1'-carbonyldiimidazole (CDI) was purchased from Oakwood and used as received.

(*E*)-cinnamaldehyde was purchased from Oakwood and used as received.

Dess-Martin periodinane (DMP) was purchased from Synthonix and used as received.

1,8-diazabicyclo[5.4.0]undec-7-ene (DBU) was purchased from Oakwood and used as received.

4,4′-di-*tert*-butyl-2,2′-dipyridyl was purchased from Combi Blocks and used as received.

***N,N′*-dicyclohexylcarbodiimide (DCC)** was purchased from Alfa Aesar and used as received.

***N,N′*-Diisopropylcarbodiimide (DIC)** was purchased from Sigma Aldrich and used as received.

***N,N*-di-*iso*-propylethylamine (*i*-Pr_2NEt)** was purchased from Macron and distilled under nitrogen over CaH_2.

4-dimethylaminopyridine (DMAP) was purchased from Oakwood and used as received.

4-4′-Dimethoxy-2-2′-bipyridine was purchased from Sigma-Aldrich and used as received.

1,4-dioxane, **anhydrous** was purchased from Sigma Aldrich and used as received.

Diphenylphosphoryl azide (DPPA) was purchased from Oakwood and used as received.

Ethanol was purchased from Pharmco and used as received.

Ethyl Acetate (EtOAc) was purchased from Macron and used as received.

1-ethyl-3-(3-dimethylaminopropyl)carbodiimide (EDC) was purchased form Oakwood and used as received.

Ethynylmagnesium bromide, 0.5 M in THF was purchased from Sigma-Aldrich and titrated with salicylaldehyde hydrazone in THF**.**

Hydrogen (H_2) was purchased from Airgas and used as received.

***N*-hydroxytetrachlorophthalimide (TCNHPI)** was purchased from Sigma Aldrich and used as received.

Imidazole was purchased from Oakwood and used as received.

Iodine (I_2) was purchased from Alfa Aesar and used as received.

Iodomethane (MeI) was purchased from Sigma Aldrich and used as received.

$(Ir[dF(CF_3)ppy]_2(dtbpy))PF_6$ was purchased from Sigma Aldrich and used as received.

2-*iso*-propyl-9H-thioxanthen-9-one (ITX) was purchased from CombiBlocks and handled in an argon-atmosphere glovebox.

Lithium aluminum hydride (LAH) was purchased from Sigma Aldrich and used as received.

Lithium borohyride ($LiBH_4$) was purchased from Sigma Aldrich and used as received.

3-(Methoxycarbonyl)cyclohexanecarboxylic acid was purchased from Ambeed and used as received.

Magnesium turnings (Mg^0) were purchased from Strem and ground in a mortar and pestle.

Methanol (MeOH) was purchased from Macron and used as received.

Methanol, anhydrous was purchased from Sigma Aldrich and used as received.

3-methylbenzyl alcohol was purchased from Oakwood and used as received.

Methylmagnesium bromide solution, 3.0 M in diethyl ether, (MeMgBr) was purchased from Sigma Aldrich and titrated with salicylaldehyde hydrazone in THF.

4-methylmorpholine *N*-oxide monohydrate ($NMO{\cdot}H_2O$) was purchased from Oakwood and used as received.

Nickel(II) chloride ethylene glycol dimethyl ether complex ($NiCl_2{\cdot}$glyme) was purchased from Strem and handled in a nitrogen-atmosphere glovebox.

Nickel(II) chloride hexahydrate ($NiCl_2 \cdot 6H_2O$) was purchased from Alfa Aesar and handled in a nitrogen-atmosphere glovebox.
Ozone was generated using a Polymetrics ozone generator and compressed O_2 from Airgas.
Palladium, 5% on activated carbon, 50-70% wetted powder (Pd/C) was purchased from Strem and used as received.
1-*Z*-piperazine was purchased from ChemImpex and used as received.
Piperidine was purchased from Alfa Aesar and used as received.
3-(piperidine-1-carbonyl)benzoic acid was purchased from Enamine and used as received.
Potassium *tert*-butoxide (KO*t*-Bu) was purchased from Strem and handled in a nitrogen-atmosphere glovebox.
Potassium carbonate (K_2CO_3) was purchased from VWR and used as received.
Pyrrolidine was purchased from Oakwood and used as received.
Ruthenium(III) chloride hydrate ($RuCl_3 \cdot xH_2O$) was purchased from Sigma-Aldrich and used as received.
Sodium bicarbonate ($NaHCO_3$) was purchased from Macron and used as received.
Sodium borohydride ($NaBH_4$) was purchased from Oakwood and used as received.
Sodium *tert*-butoxide (NaO*t*-Bu) was purchased from TCI and handled in a nitrogen-atmosphere glovebox.
Sodium Iodide (NaI) was purchased from EMD and used as received.
Sodium metaperiodate ($NaIO_4$) was purchased from Oakwood and used as received.
Tetrabutylammonium fluoride, **1.0 M in THF (TBAF)** was purchased from Sigma Aldrich and used as received.
Tetrapropylammonium perruthenate (TPAP) was purchased from TCI and used as received.
Triethylamine (Et_3N) was purchased from EMD and distilled over CaH_2 under N_2.
Tri-*iso*-propylsilylchloride (TIPSCl) was purchased from Oakwood and used as received.
Tris(dibenzylideneacetone)dipalladium(0) (Pd_2dba_3) was purchased from Sigma Aldrich and handled in a nitrogen-atmosphere glovebox.
Triphenylphosphine (PPh_3) was purchased from Oakwood and used as received.
Zinc(II) chloride ($ZnCl_2$) was purchased from Alfa Aesar, dried with a heat gun under vacuum, and handled in a nitrogen-atmosphere glovebox.

Synthesis of Reagents

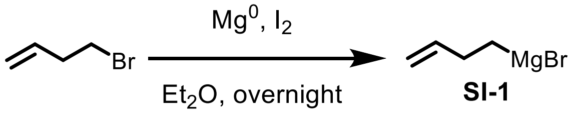

but-3-en-1-ylmagnesium bromide (SI-1): Freshly ground Mg^0 turnings (49 g, 2000 mmol, 5 equiv.) were added to a flame-dried 1000 mL three-neck round-bottom flask with stir bar. The middle neck was fitted with a reflux condenser and a glass stopper and septum on the remaining two necks. The reflux apparatus was flame-dried under vacuum and allowed to cool to room temperature before being refilled with N_2. A crystal of I_2 was added to the flask, and the flask evacuated and refilled with N_2 three times. Another septum was fitted to the top of the reflux condenser, and the whole apparatus was put under N_2. Et_2O (400 mL, 1.0 M) was added via cannula. The Mg^0, I_2, and Et_2O were vigorously stirred until the iodine color disappeared. 4-bromobut-1-ene (40 mL, 400 mmol, 1 equiv.) was added neat dropwise through the

septum on the three-neck flask while keeping a gentle reflux. The reaction was stirred at room temperature for approx. 18 hours and achieved a dark grey to black color. The solution was titrated according to the procedure by Love.[1] Titrations typically produce concentrations between 0.7-0.8 molar. The resulting solution was used immediately in the next reaction.

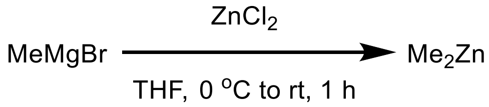

Dimethylzinc: Procedure was adapted from literature.[2] $ZnCl_2$ (0.2730 g, 1.000 equiv., 2.000 mmol) was added to a flame-dried scintillation vial with stir bar in a N_2-atmosphere glovebox. The vial was capped with a septum, sealed with Teflon tape, and removed from the glovebox. Under N_2, THF (4.0 mL, 0.50 molar) was added to the vial, and the resulting solution was cooled to 0 °C in an ice bath. MeMgBr (1.5 mL, 2.7 molar, 2.0 equiv., 4.0 mmol) was added to the mixture, and the resulting solution was stirred for 1 hour. The solids were allowed to settle before the solution was used. The solution was not titrated further.

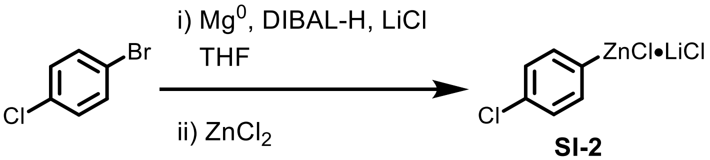

SI-2

ArylZnCl·LiCl (SI-2): Procedure was adapted from literature.[4] Mg^0 turnings (0.2215 g, 1.519 equiv., 9.111 mmol) were added to a scintillation vial with stir bar. The vial was flame-dried under vacuum, refilled with N_2, and brought into a N_2-containing glovebox. LiCl (0.3179 g, 1.250 equiv., 7.500 mmol) was added to the vial. The vial was capped with a septum, sealed with Teflon tape, and removed from the glovebox. Under N_2, THF (3.0 mL, 2.0 molar) was added, and the resulting mixture cooled to 0 °C in an ice bath. DIBAL-H (0.06 mL, 1.0 molar in THF, .01 equiv., 0.06 mmol) was added dropwise at 0 °C to the mixture. 1-bromo-4-chlorobenzene (1.149 g, 1.000 equiv., 6.000 mmol) was added to a separate flame-dried 1-dram vial. The vial was evacuated and refilled with N_2 three times. 0.5 mL of THF was used to transfer the ArBr to the vial containing Mg^0 turnings. After the initial heat evolution, the reaction mixture was removed from the ice bath and allowed to stir for 1 hour at room temperature. Titration of the mixture with I_2 (0.0125 g, 0.05 mmol) and LiCl (0.25 mmol, 0.0105 g) in THF (1 mL) yielded a concentration of 0.74 molar.

$ZnCl_2$ (0.2726 g, 1.000 equiv., 2.000 mmol) was added to a third flame-dried vial with stir bar in the glovebox. The vial was capped with a septum, sealed with Teflon tape, and removed from the glovebox. Under N_2, THF (6.0 mL, 0.33 molar) was added to the vial containing $ZnCl_2$, and the resulting mixture was cooled to 0 °C in an ice bath. The ArMgBr·LiCl solution (2.7 mL, 0.74 molar in THF) was added to the $ZnCl_2$ mixture, and the resulting solution was removed from the ice bath. The ArylZnCl·LiCl solution stirred at room temperature for 15 min. The ArylZnCl·LiCl was not titrated further, and the solids were allowed to settle before the ArylZnCl·LiCl solution was used.

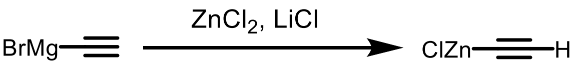

SI-3

Ethynylzinc chloride (SI-3): Procedure was adapted from literature.[3] $ZnCl_2$ (0.1360 g, 1.000 equiv., 1.000 mmol) and LiCl (0.0424 mg, 1.00 equiv., 1.00 mmol) were added to a flame-dried 2-dram vial with stir bar in a N_2-containing glovebox. The vial was capped with a septum, sealed with Teflon tape, and removed from the glovebox. Under N_2, THF (1.0 mL, 1.0 molar) was added followed by dropwise addition of ethynylmagnesium bromide solution (2.2 mL, 0.45 molar in THF, 1.0 equiv., 1.0 mmol) at room temperature. The resulting solution was stirred at room temperature for 30 min. until it became homogeneous. The solution was not titrated further.

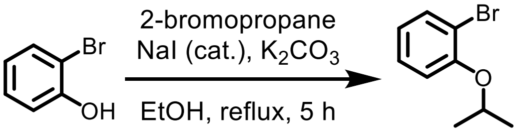

SI-4

1-bromo-2-isopropoxybenzene (SI-4): 1-bromo-2-isopropoxybenzene was prepared according to literature.[5] K_2CO_3 (1.73 g, 1.20 equiv., 12.5 mmol) and NaI (47 mg, 0.03 equiv., 0.31 mmol) were added to a 100 mL two-necked flask with stir bar and fitted with a reflux-condenser were added. The reflux apparatus was evacuated and refilled with N_2 three times. The reflux condenser was fitted with a septum and kept under N_2. Via syringe was added abs. EtOH (26 mL) followed by 2-bromophenol (1.1 mL, 10.4 mmol, 1.00 equiv.) and 2-bromopropane (1.2 mL, 12.5 mmol, 1.20 equiv.). The reaction mixture was refluxed for 5 h. It was cooled to room temperature and the solvent removed under reduced pressure. The mixture was redissolved in EtOAc and washed with 1 M NaOH followed by brine. The organic layer was dried over Na_2SO_4, filtered, and concentrated under reduced pressure to yield a clear, colorless liquid (1.3535 g, 63% yield). It was used without further purification. Characterization data matched that of the literature.[5]

^{1}H NMR (500 MHz, $CDCl_3$) δ 7.53 (d, *J* = 7.9 Hz, 1H), 7.23 (t, *J* = 7.8 Hz, 1H), 6.92 (d, *J* = 8.2 Hz, 1H), 6.82 (t, *J* = 7.6 Hz, 1H), 4.55 (hept, *J* = 6.1 Hz, 1H), 1.38 (d, *J* = 6.1 Hz, 6H).

^{13}C NMR (126 MHz, $CDCl_3$) δ 154.74, 133.64, 128.39, 122.06, 116.00, 113.95, 72.29, 22.22.

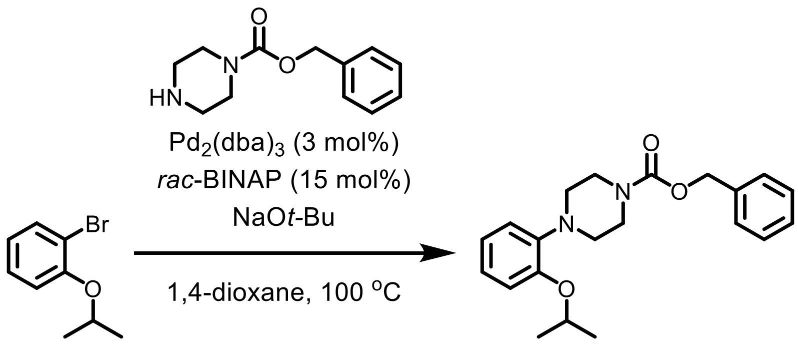

SI-4 SI-5

benzyl 4-(2-isopropoxyphenyl)piperazine-1-carboxylate (SI-5): Procedure was adapted from literature.[6] $Pd_2(dba)_3$ (0.0183 g, 0.02 mmol, 0.003 equiv.), *rac*-BINAP (0.0623 g, 0.100 mmol, 0.015 equiv.), NaO*t*-Bu (0.192 g, 2.00 mmol, 3.00 equiv.) were added to a flame-dried 6 mL screw-cap test tube vial in a N_2-atmosphere glovebox. The vial was capped with a septum and removed from the glovebox. Under N_2, 1,4-dioxane (6.6 mL, 0.1 molar), aryl bromide **SI-4** (0.145 g, 0.674 mmol, 1.00 equiv.), and benzyl piperazine-1-carboxylate (0.3 mL, 1.50 mmol, 2.22 equiv.) were added to the vial. The vial was sealed with a Teflon screwcap and Teflon tape. The resulting solution was stirred at 80 °C in an aluminum block for approx. 18 hours. The reaction mixture was cooled to room temperature, and filtered through a pad of Celite, which was washed twice with CH_2Cl_2 and then twice with EtOAc. The combined rinses were concentrated under reduced pressure and purified by MPLC to yield a clear, colorless oil (0.1621 g, 68% yield).

^{1}H NMR (500 MHz, $CDCl_3$) δ 7.43 – 7.31 (m, 5H), 7.02 – 6.85 (m, 4H), 5.17 (d, *J* = 1.8 Hz, 2H), 4.59 (p, *J* = 6.2 Hz, 1H), 3.67 (t, *J* = 5.0 Hz, 4H), 3.04 (s, 4H), 1.35 (dd, *J* = 6.3, 1.6 Hz, 6H).

^{13}C NMR (126 MHz, $CDCl_3$) δ 155.53, 150.56, 142.44, 136.91, 128.67, 128.18, 128.09, 123.13, 121.44, 118.80, 115.74, 70.45, 67.31, 50.57, 44.32, 22.44.

FTIR: 2973.11 (w), 2814.92 (w), 1697.89 (s), 1593.83 (w), 1494.77 (m), 1426.28 (m), 1233.01 (s), 1218.23 (s), 1120.19 (s), 952.86 (m), 744.65 (s), 696.52 (s).

HRMS (APCI): Calculated for $C_{21}H_{27}O_3N_2$ [M+H$^+$]: 355.2016, Found: 355.2019.

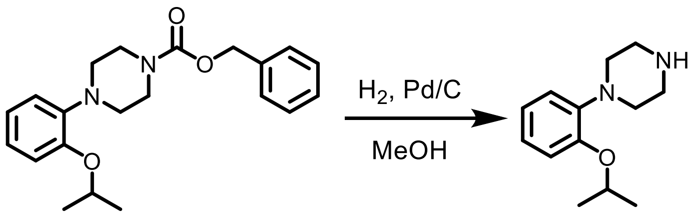

SI-5 SI-6

1-(2-isopropoxyphenyl)piperazine (SI-6): Procedure was adapted from literature.[7] **SI-5** (0.0691 g, 0.195 mmol, 1.00 equiv.) was added to a 1-dram vial with stir bar. MeOH (2.0 mL, 0.1 molar) was added to the vial under N_2 followed by addition of Pd/C (207 mg, 0.0975 mmol, 0.5 equiv.). The solution was sparged with N_2 for 5 minutes, and then sparged with a balloon full of H_2 for 5 minutes. The reaction mixture stirred under H_2 for approx. 18 hours before being filtered through a pad of Celite. The pad of Celite was rinsed

twice with EtOAC and then twice with CH_2Cl_2. The combined rinses were concentrated under reduced pressure to yield a light-yellow oil (0.0280 g, 65% yield). Material was used in the next reaction without further purification. Characterization data matched that from the literature.[7]

^{1}H NMR (500 MHz, CDCl$_3$) δ 6.98 – 6.82 (m, 4H), 4.60 (hept, *J* = 6.0 Hz, 1H), 3.06 (s, 8H), 2.12 (s, 1H), 1.35 (d, *J* = 6.1 Hz, 6H).

^{13}C NMR (126 MHz, CDCl$_3$) δ 150.41, 143.18, 122.51, 121.44, 118.52, 116.09, 70.28, 51.76, 46.33, 22.32.

General Procedures

General Procedure A: Addition of Grignard to Aldehyde Derivatives:

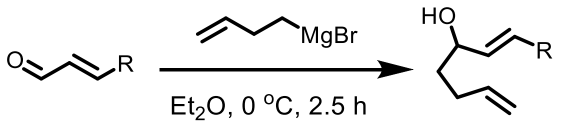

Aldehyde (1 equiv.) was added to a flame-dried round-bottomed flask with stir bar under N_2. The aldehyde was dissolved in Et_2O (0.8 molar) and cooled to 0 °C in an ice bath. **SI-1** (1.2 equiv) was added dropwise via syringe. Vigorous stirring was required so that the stir bar did not get stuck in the yellow precipitate that formed during addition. The precipitate dissolved shortly after addition of all the Grignard reagent. After the reaction was complete by TLC analysis (~2.5 h), the reaction was quenched with sat. aq. NH_4Cl at 0 °C. Additional 2M HCl was added to dissolve white precipitate that formed and to adjust the pH to 1-2. The organic layer was separated, and the aqueous layer was extracted with Et_2O twice more. The combined organic layers were dried over $MgSO_4$, filtered, and concentrated under reduced pressure. The crude residue was used directly in the next step without further purification.

General procedure B: Addition of Grignard to the Cinnamaldehyde Derivatives:

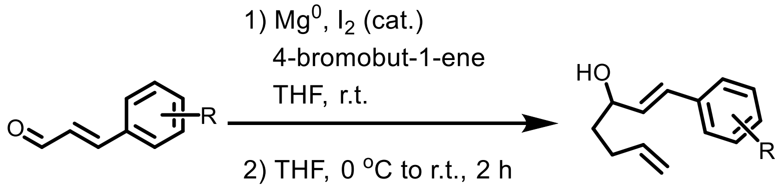

Mg^0 turnings (5 equiv.) and I_2 (0.20 equiv.) were added to a flame-dried 2-necked round bottom flask equipped with a magnetic stirrer under N_2. Then, the flask was equipped with a condenser and a rubber septum on the two necks. Under N_2, Et_2O (0.5 molar) was added to the flask and the resulting mixture was refluxed for 30 minutes. Then, 4-bromobut-1-ene (2.0 equiv.) dissolved in Et_2O (0.5 molar) was added to the reaction mixture while keeping a gentle reflux. The mixture was stirred for an additional hour at reflux and upon completion of the Grignard reagent formation, the heating and stirring were stopped. Cinnamaldehyde derivative (1.0 equiv.) was dissolved in Et_2O (0.5 molar) under a nitrogen atmosphere in a separate flask. The mixture was cooled to -20 °C, and the solution of freshly prepared Grignard was added. A yellow precipitate formed upon addition, which dissolved after complete addition. After 1 hour of stirring, TLC showed completion of the reaction, which was then quenched by sat. aq. NH_4Cl solution.

The organics were extracted with Et_2O thrice, then the combined organic layers were washed with brine and dried over Na_2SO_4. The dried organic layer was filtered and concentrated under reduced pressure. The crude residue was used directly in the next step without further purification.

General procedure C: [2+2] Photocycloaddition using 2-Isopropylthioxanthone:

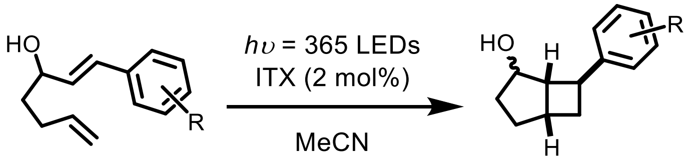

Procedure was adapted from literature.[8] ITX (0.02 equiv.) was weighed out in a flame-dried round-bottomed flask. The flask was capped with a septum and put under a N_2 atmosphere. Crude diene was dissolved in dry MeCN (0.2 molar) under N_2 in a separate flame-dried flask. The solution of diene was degassed with N_2 for 5 minutes and transferred to the flask containing the ITX. The resulting mixture was degassed with N_2 for 15 minutes. Then, the mixture was transferred to a flame-dried Quartz-tube equipped with a septum and a magnetic stirrer. The mixture was irradiated at 365 nm (Evoluchem HCK1012-01-006; 30 W AC200-240V) in an EvoluChem PhotoRedOx Box for 24-48 hours under N_2. Once completed (by TLC analysis), the reaction mixture was concentrated under reduced pressure and used in the next step without further purification.

General procedure D: [2+2] Photocycloaddition using Iridium Photocatalyst:

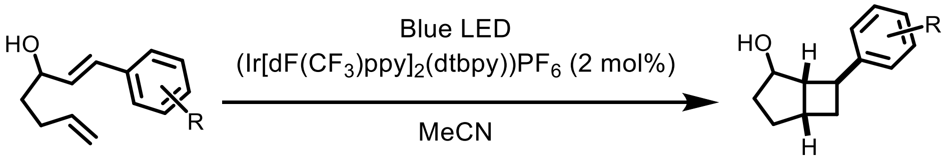

The crude diene was dissolved in dry MeCN (0.30 molar) in a vial and $(Ir[dF(CF_3)ppy]_2(dtbpy))PF_6$ (0.02 equiv.) was added. The resulting solution was degassed with N_2 for 2 minutes before being sealed and irradiated with blue LEDs for 4 hours. TLC analysis showed full conversion and the mixture was evaporated under reduced pressure before being purified by Flash column chromatography (Hexanes:EtOAc 95:5 to 50:50) to yield a mixture of diastereomers as an oil. Crude residue was used directly in the next step without further purification.

General procedure E: [2+2] Photocycloaddition using 2-Isopropylthioxanthone in Flow:

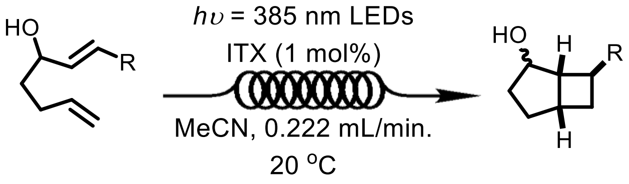

ITX (1 mol%) was added to an oven-dried 2-dram vial in an Ar-atmosphere glovebox. The vial was capped with a septum and removed from the glovebox. The vial was placed under N_2. MeCN (0.1 M) was added to a flame-dried 500 mL round-bottomed flask with stir bar under N_2. Crude diene (1 equiv.) was added to the flask containing MeCN followed by ITX under N_2. Approx. 10 mL more of MeCN was used to help transfer the diene and ITX to the reaction flask. The reaction mixture was sparged with N_2 for 30 minutes while stirring.

The Vapourtec E-Series flow reactor equipped with a UV-150 10 mL photoreactor was pre-equilibrated with anhydrous MeCN. Then, the reaction solution was pumped through the reactor at a rate of 0.222 mL/min (45 min. residence time) while being irradiated at 385 nm using a 60 W LED array. The reaction solution was kept under N_2. The temperature in the photoreactor was kept at 20 °C, and the pressure in the coil was kept between 2-5 bar using a back-pressure regulator during the reaction. The solution of product was collected in a round-bottomed flask open to the air. Once the reaction mixture was consumed, anhydrous MeCN was again pumped through the reactor at the same rate of 0.222 mL/min for 45 min. This solution was collected in the same Erlenmeyer. Upon completion, the combined solutions were concentrated under reduced pressure. The crude yellow oil was used in the next reaction without further purification.

General procedure F: DMP Oxidation of Bicyclo[3.2.0] intermediates:

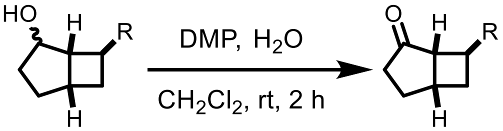

Dess-Martin Periodinane (1.5 equiv.) was added to a round-bottomed flask with stir bar. It was evacuated and refilled with N_2 three times. The flask was capped with a septum and placed under N_2. CH_2Cl_2 (0.1 M) was added via cannula followed by addition of DI H_2O (1.5 equiv.). Crude alcohol was added to the reaction flask, and the reaction stirred for 2 hours at room temperature. The reaction was quenched with a 1:1 sat. aq. $Na_2S_2O_3$: sat. aq. $NaHCO_3$ solution and stirred until CO_2 production ceased. The mixture was transferred to a separatory funnel and the layers were separated. The aqueous layer was extracted twice more with CH_2Cl_2. The organic layers were combined and washed with DI H_2O followed by brine. The combined organic layers were dried over $MgSO_4$, filtered, and concentrated under reduced pressure. Material was used in the next reaction without further purification.

General procedure G: Formation of the Bicyclo[3.2.0] α-Diazoketone:

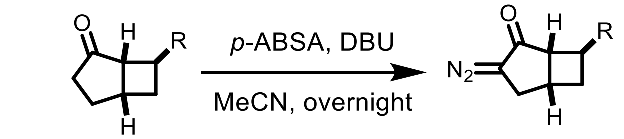

MeCN (0.1 M) was added to a flame-dried round-bottomed flask with stir bar under N_2. Crude ketone (1 equiv.) was added to the flask. The septum was removed, and *p*-ABSA (2.5 equiv.) was quickly added. The septum was replaced, and the reaction was flushed with N_2. DBU (5 equiv.) was added in one portion via syringe. The reaction mixture was stirred at room temperature for approx. 18 hours. The reaction was diluted with a 1:1 EtOAc:Hexanes solution while stirring until a viscous brown oil precipitated from the reaction mixture. The mixture was filtered through a fritted funnel filled with silica gel (wetted with Hexanes) under vacuum. The crude material was purified via flash column chromatography.

General Procedure H: Formation of the Bicyclo[2.2.0] Structure in Flow:

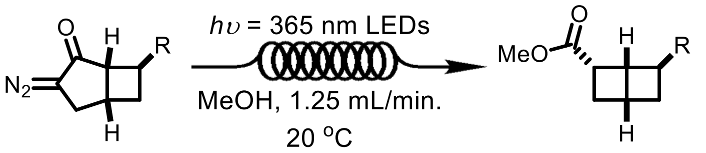

α-Diazoketone (3.6 g, 17 mmol, 1 equiv.) was added to a flame-dried round-bottomed flask with stir bar under N_2. LCMS grade MeOH (0.1 M) was added to the flask, and the resulting solution was sparged with N_2 for 30 minutes while stirring.

The Vapourtec E-Series flow reactor equipped with a UV-150 10 mL photoreactor was pre-equilibrated with LCMS grade MeOH. Then, the reaction solution was pumped through the reactor at a rate of 1.25 mL/min (8 min. residence time) while being irradiated at 365 nm using a 150 W LED array. The reaction solution was kept under N_2. The temperature in the photoreactor was kept at 20 °C, and the pressure in the coil was kept between 2-5 bar using a back-pressure regulator during the reaction. The solution of product was collected in a 500 mL Erlenmeyer flask open to the air. Once the reaction mixture was consumed, LCMS grade MeOH was again pumped through the reactor at the same rate of 1.25 mL/min for 8 min. This solution was collected in the same Erlenmeyer. Upon completion, the combined solutions were concentrated under reduced pressure and purified via MPLC.

General Procedure I: Formation of the [2.2.0] Structure in Batch:

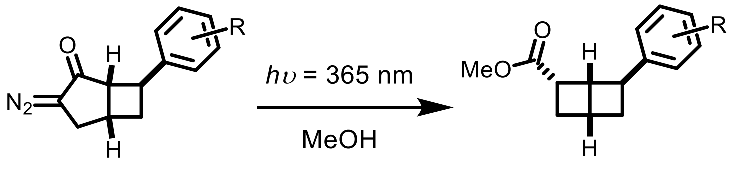

α-Diazoketone (1 equiv.) was added to a round-bottomed flask with stir bar under N_2. Dry MeOH (0.1 molar) was added, and the solution was sparged with N_2 for 60 minutes. The mixture was transferred to a flame dried quartz tube under a N_2 atmosphere. The reaction was irradiated at 365 nm for approx. 18

hours in the EvoluChem PhotoRedOx. The reaction mixture was concentrated under reduced pressure and purified via column chromatography.

Characterization Data of Intermediates and Bicyclo[2.2.0] Final Substrates

1. Precursors to Bicyclo[3.2.0]heptanes

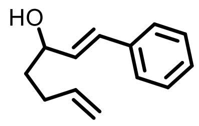

SI-7

(*E*)-1-phenylhepta-1,6-dien-3-ol (SI-7): Following General Procedure A with (*E*)-cinnamaldehyde (15 mL, 120 mmol), **SI-1** and was obtained as a yellow oil (21.9028 g, 97% yield) and used without further purification.

^{1}H NMR (500 MHz, $CDCl_3$) δ 7.35 – 7.30 (m, 2H), 7.26 (dd, *J* = 8.5, 6.8 Hz, 2H), 7.21 – 7.16 (m, 1H), 6.52 (dd, *J* = 16.0, 1.1 Hz, 1H), 6.16 (dd, *J* = 15.9, 6.8 Hz, 1H), 5.80 (ddt, *J* = 16.9, 10.2, 6.6 Hz, 1H), 5.01 (dq, *J* = 17.1, 1.7 Hz, 1H), 4.94 (ddd, *J* = 10.2, 2.2, 1.1 Hz, 1H), 4.29 – 4.23 (m, 1H), 2.14 (dddt, *J* = 11.5, 8.2, 4.9, 1.6 Hz, 2H), 1.75 – 1.60 (m, 3H).

^{13}C NMR (126 MHz, $CDCl_3$) δ 138.35, 136.79, 132.37, 130.57, 128.72, 127.82, 126.60, 115.13, 72.66, 36.46, 29.86.

FTIR: 3333.67 (br), 3025.83 (m), 2922.33 (w), 1639.73 (m), 1493.19 (m), 1448.12 (m), 964.63 (s), 908.99 (s), 746.38 (s), 691.60 (s), 547.98 (w).

HRMS (EI): Calculated for $C_{13}H_{16}O$ [M$^+$]: 188.1196, Found: 188.1199.

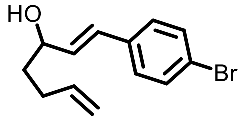

SI-8

(*E*)-1-(4-bromophenyl)hepta-1,6-dien-3-ol (SI-8): Following General Procedure B with (*E*)-3-(4-bromophenyl)acrylaldehyde (350 mg, 1.70 mmol), **SI-8** was obtained as a yellow oil (443 mg, quant.) and used without further purification.

^{1}H NMR (400 MHz, $CDCl_3$) δ 7.48 – 7.38 (m, 2H), 7.29 – 7.20 (m, 2H), 6.59 – 6.44 (m, 1H), 6.21 (dd, J = 15.9, 6.6 Hz, 1H), 5.85 (ddt, J = 16.9, 10.2, 6.7 Hz, 1H), 5.14 – 4.90 (m, 2H), 4.31 (tdd, J = 7.0, 5.9, 1.2 Hz, 1H), 2.19 (dtdd, J = 8.2, 6.6, 3.4, 1.8 Hz, 2H), 1.82 – 1.67 (m, 2H), 1.64 (s, 1H).

^{13}C NMR (101 MHz, CDCl$_3$) δ 138.24, 135.78, 133.18, 131.84, 129.30, 128.13, 121.57, 115.26, 72.49, 36.44, 29.85.

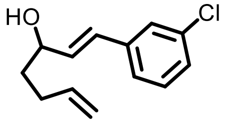

SI-9

(*E*)-1-(3-chlorophenyl)hepta-1,6-dien-3-ol (SI-9): Following General Procedure B with (*E*)-3-(3-chlorophenyl)acrylaldehyde (1.0 g, 6.0 mmol), **SI-9** was obtained as a yellow oil (1.30 g, quant.) and used without further purification.

^{1}H NMR (400 MHz, CDCl$_3$) δ 7.37 (tt, *J* = 1.2, 0.7 Hz, 1H), 7.26 – 7.19 (m, 3H), 6.52 (dd, *J* = 15.9, 1.2 Hz, 1H), 6.23 (dd, *J* = 15.9, 6.5 Hz, 1H), 5.85 (ddt, *J* = 17.0, 10.2, 6.7 Hz, 1H), 5.07 (dq, *J* = 17.1, 1.7 Hz, 1H), 5.03 – 4.98 (m, 1H), 4.32 (tdd, *J* = 7.0, 6.0, 1.3 Hz, 1H), 2.20 (tddd, *J* = 8.3, 6.8, 2.9, 1.4 Hz, 2H), 1.82 – 1.62 (m, 3H).

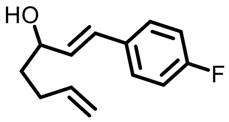

SI-10

(*E*)-1-(4-fluorophenyl)hepta-1,6-dien-3-ol (SI-10): Following General Procedure B with (*E*)-3-(4-fluorophenyl)acrylaldehyde (500 mg, 3.33 mmol), **SI-10** was obtained as a yellow oil (450 mg, 66% yield) and used further without further purification.

^{1}H NMR (400 MHz, CDCl$_3$) δ 7.40 – 7.28 (m, 2H), 7.08 – 6.94 (m, 2H), 6.54 (dd, *J* = 15.9, 1.1 Hz, 1H), 6.14 (dd, *J* = 15.9, 6.7 Hz, 1H), 5.86 (ddt, *J* = 16.9, 10.2, 6.6 Hz, 1H), 5.13 – 4.94 (m, 2H), 4.31 (tdd, *J* = 7.0, 5.8, 1.2 Hz, 1H), 2.19 (dtdt, *J* = 9.7, 6.6, 3.1, 1.5 Hz, 2H), 1.84 – 1.63 (m, 3H).

^{13}C NMR (101 MHz, CDCl$_3$) δ 162.48 (d, J = 246.9 Hz), 138.28, 132.96 (d, J = 3.3 Hz), 132.10 (d, J = 2.2 Hz), 129.38, 128.09 (d, J = 8.0 Hz), 115.62 (d, J = 21.6 Hz), 115.17, 72.57, 36.48, 29.85.

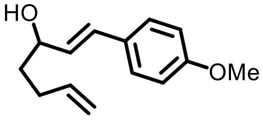

SI-11

(*E*)-1-(4-methoxyphenyl)hepta-1,6-dien-3-ol (SI-11): Following General Procedure B with (*E*)-3-(4-methoxyphenyl)acrylaldehyde (550 mg, 3.39 mmol), **SI-11** was obtained as a yellow oil (740 mg, quant.) and used without further purification.

^{1}H NMR (400 MHz, CDCl$_3$) δ 7.37 – 7.27 (m, 2H), 6.91 – 6.82 (m, 2H), 6.51 (d, *J* = 15.9 Hz, 1H), 6.08 (dd, *J* = 15.9, 7.0 Hz, 1H), 5.84 (dddt, *J* = 17.2, 13.3, 10.2, 6.8 Hz, 1H), 5.06 (d, *J* = 17.1 Hz, 1H), 4.99 (d, *J* = 10.3 Hz, 1H), 4.33 – 4.23 (m, 1H), 3.81 (s, 3H), 2.18 (dtdd, *J* = 9.6, 6.6, 3.0, 1.3 Hz, 2H), 1.85 – 1.62 (m, 3H).

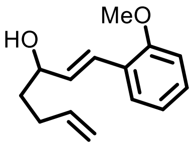

SI-12

(*E*)-1-(2-methoxyphenyl)hepta-1,6-dien-3-ol (SI-12): Following General Procedure B with (*E*)-3-(2-methoxyphenyl)acrylaldehyde (550 mg, 3.39 mmol), **SI-12** was obtained as a yellow oil (710 mg, 96% yield) and used without further purification.

^{1}H NMR (400 MHz, CDCl$_3$) δ 7.44 (d, *J* = 5.9 Hz, 1H), 7.26 – 7.20 (m, 1H), 6.98 – 6.84 (m, 3H), 6.23 (dd, *J* = 16.0, 7.0 Hz, 1H), 5.93 – 5.81 (m, 1H), 5.12 – 4.96 (m, 2H), 4.32 (q, *J* = 5.9 Hz, 1H), 3.85 (s, 3H), 2.27 – 2.12 (m, 2H), 1.75 (d, *J* = 8.4 Hz, 3H).

^{13}C NMR (101 MHz, CDCl$_3$) δ 156.87, 138.47, 133.02, 128.86, 126.99, 125.78, 125.47, 120.77, 114.98, 110.99, 73.14, 55.56, 36.45, 29.91.

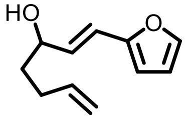

SI-13

(*E*)-1-(furan-2-yl)hepta-1,6-dien-3-ol (SI-13): Following General Procedure B with (*E*)-3-(furan-2-yl)acrylaldehyde (1.00 g, 8.20 mmol), **SI-13** was obtained as a yellow oil (1.50 g, quant.) and used without further purification.

^{1}H NMR (400 MHz, CDCl$_3$) δ 7.34 (d, *J* = 1.9 Hz, 1H), 6.41 (dd, *J* = 15.9, 1.2 Hz, 1H), 6.37 (dd, *J* = 3.3, 1.8 Hz, 1H), 6.24 (d, *J* = 3.3 Hz, 1H), 6.17 (dd, *J* = 15.9, 6.5 Hz, 1H), 5.85 (ddt, *J* = 16.9, 10.2, 6.7 Hz, 1H), 5.06 (dq, *J* = 17.1, 1.7 Hz, 1H), 5.02 – 4.96 (m, 1H), 4.28 (q, *J* = 6.4 Hz, 1H), 2.18 (dtdd, *J* = 8.2, 6.5, 3.3, 1.7 Hz, 2H), 1.78 – 1.68 (m, 3H).

^{13}C NMR (101 MHz, CDCl$_3$) δ 152.48, 142.09, 138.30, 131.01, 118.75, 115.12, 111.42, 108.17, 72.15, 36.48, 29.81.

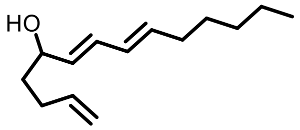

SI-14

(6*E*,8*E*)-tetradeca-1,6,8-trien-5-ol (SI-14): Following General procedure A with (2*E*,4*E*)-deca-2,4-dienal (1.5220 g, 10.00 mmol), **SI-14** was obtained as a yellow oil (2.0128 g, 97% yield) and used without further purification.

^{1}H NMR (500 MHz, CDCl$_3$) δ 6.18 (dd, *J* = 15.3, 10.4 Hz, 1H), 6.02 (dd, *J* = 15.1, 10.5 Hz, 1H), 5.83 (ddt, *J* = 16.9, 10.0, 6.6 Hz, 1H), 5.71 (dt, *J* = 14.7, 7.0 Hz, 1H), 5.57 (dd, *J* = 15.2, 7.0 Hz, 1H), 5.04 (dq, *J* = 17.2, 1.9 Hz, 1H), 4.97 (d, *J* = 10.2 Hz, 1H), 4.15 (q, *J* = 6.7 Hz, 1H), 2.21 – 2.03 (m, 4H), 1.72 – 1.54 (m, 2H), 1.47 (s, 1H), 1.39 (p, *J* = 7.4 Hz, 2H), 1.29 (pd, *J* = 8.4, 3.0 Hz, 4H), 0.89 (t, *J* = 6.8 Hz, 3H).

^{13}C NMR (126 MHz, CDCl$_3$) δ 138.45, 135.95, 133.32, 131.34, 129.48, 114.97, 72.47, 36.45, 32.75, 31.55, 29.88, 29.04, 22.67, 14.18.

FTIR: 3331.28 (br), 2956.47 (m), 2923.92 (m), 2855.16 (m), 1454.06 (w), 1055.59 (w), 985.45 (s), 908.23 (s).

HRMS (EI): Calculated for $C_{14}H_{24}O$ [M$^+$]: 208.1822, Found: 208.1818.

2. [2+2] Cycloaddition to Bicyclo[3.2.0] Systems

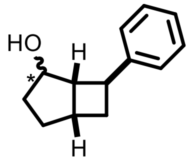

SI-15

7-phenylbicyclo[3.2.0]heptan-2-ol (SI-15): Following General procedure E with (*E*)-1-phenylhepta-1,6-dien-3-ol (30.0 mmol), **SI-15** (2:1 dr) was obtained as a light-yellow oil and used without further purification. The efficiency was 1.5 mmol h^{-1}.

^{1}H NMR (600 MHz, CDCl$_3$) δ 7.34 – 7.29 (m, 3H), 7.26 (d, J = 2.4 Hz, 3H), 7.21 – 7.16 (m, 1.5H), 4.32 (dt, J = 10.2, 6.7 Hz, 0.5H), 4.23 (d, J = 3.9 Hz, 1H), 3.55 – 3.48 (m, 0.5H), 2.93 (td, J = 11.8, 7.4 Hz, 1H), 2.89 – 2.84 (m, 1H), 2.80 (q, J = 6.7 Hz, 0.5H), 2.72 (q, J = 7.1 Hz, 1.5H), 2.47 (dt, J = 12.6, 8.4 Hz, 0.5H), 2.33 (ddd, J = 12.7, 9.7, 7.4 Hz, 1H), 2.26 – 2.16 (m, 1H), 2.12 – 1.90 (m, 4.5H), 1.79 – 1.71 (m, 0.5H), 1.62 (td, J = 13.1, 7.1 Hz, 1.5H), 1.52 (s, 1.5H).

^{13}C NMR (126 MHz, CDCl$_3$) δ 147.19 (*minor*), 146.52, 128.52, 128.46 (*minor*), 126.78 (*minor*), 126.57, 125.95, 125.70 (*minor*), 78.33, 75.43 (*minor*), 54.79, 49.83 (*minor*), 39.61, 34.44 (*minor*), 33.84, 33.48, 33.19 (*minor*), 32.81 (*minor*), 32.33 (*minor*), 31.94, 30.31, 29.68 (*minor*).

FTIR: 3265.79 (br), 2925.64 (m), 1492.57 (m), 1443.98 (m), 1305.95 (m), 1079.94 (m), 986.83 (s), 735.54 (m), 695.39 (s), 525.46 (m).

HRMS (APCI): Calculated for $C_{13}H_{15}O$ [M-H+]: 187.1117, Found: 187.1116.

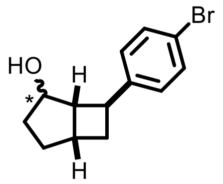

SI-16

7-(4-bromophenyl)bicyclo[3.2.0]heptan-2-ol (SI-16): Following General Procedure C with crude **SI-8** (770 mg, 2.88 mmol), **SI-16** was obtained (770 mg, quant., 1.5:1 dr) and used without further purification.

^{1}H NMR (400 MHz, CDCl$_3$) δ 7.44 – 7.39 (m, 3.3H), 7.18 – 7.10 (m, 3.3H), 4.31 (dt, J = 10.1, 6.6 Hz, 0.66H), 4.21 (d, J = 3.8 Hz, 1H), 3.50 – 3.43 (m, 0.66H), 2.96 – 2.87 (m, 1H), 2.85 – 2.78 (m, 1H), 2.76 – 2.64 (m, 2.3H), 2.46 – 2.37 (m, 0.66H), 2.31 – 2.14 (m, 2.3H), 2.11 – 1.88 (m, 5.3H), 1.80 – 1.68 (m, 0.66H), 1.61 (dt, J = 14.3, 7.5 Hz, 2.66H).

^{13}C NMR (101 MHz, CDCl$_3$) δ 146.18 (*minor*), 145.49, 131.54, 131.44 (*minor*), 128.62 (*minor*), 128.38, 119.57, 119.31 (*minor*), 78.26, 75.33 (*minor*), 54.68, 49.90 (*minor*), 39.10, 34.01 (*minor*), 33.89, 33.46, 33.12 (*minor*), 32.62 (*minor*), 32.37 (*minor*), 31.88, 30.26, 29.66 (*minor*).

FTIR: 3399, 2933, 2082, 1644, 1600, 1508, 1491, 1458, 1437, 1329, 1291, 1241, 1221, 1157, 1114, 1052, 1029 cm^{-1}.

HRMS (ESI): Calculated for $C_{13}H_{15}BrNaO$ [M+Na+]: 289.0198, Found: 289.0205.

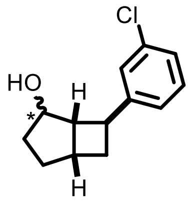

SI-17

7-(3-chlorophenyl)bicyclo[3.2.0]heptan-2-ol (SI-17): Following General Procedure C with crude **SI-9** (1.90 g, 8.53 mmol), **SI-17** was obtained (1.90 g, quant., 2:1 dr) as an oil and used without further purification.

^{1}H NMR (400 MHz, CDCl$_3$) 7.29 – 7.20 (m, 3H), 7.18 – 7.10 (m, 3H), 4.32 (dt, *J* = 10.1, 6.7 Hz, 0.5H), 4.22 (d, *J* = 3.8 Hz, 1H), 3.54 – 3.46 (m, 0.5H), 2.98 – 2.88 (m, 1H), 2.88 – 2.81 (m, 1H), 2.80 – 2.66 (m, 2H), 2.44 (dt, *J* = 12.7, 8.5 Hz, 0.5H), 2.30 (ddd, *J* = 12.7, 9.7, 7.3 Hz, 1H), 2.25 – 2.14 (m, 1H), 2.12 – 1.89 (m, 4.5H), 1.81 – 1.69 (m, 0.5H), 1.61 (dt, *J* = 12.9, 8.1 Hz, 1.5H), 1.45 (s, 1.5H).

^{13}C NMR (101 MHz, CDCl$_3$) δ 148.55, 134.37, 129.79, 129.69 (*minor*), 127.00 (*minor*), 126.83, 126.10, 125.84 (*minor*), 125.05 (*minor*), 124.78, 78.24, 75.28 (*minor*), 54.59, 49.79 (*minor*), 39.30, 34.25 (*minor*), 33.88, 33.50, 33.16 (*minor*), 32.36 (*minor*), 31.71, 30.26, 29.65 (*minor*).

FTIR: 3369, 2933, 2114, 1604, 1508, 1439, 1265, 1222, 1158, 1094, 1054, 1014 cm^{-1}.

LRMS: Calculated for $C_{13}H_{16}ClO$ [M+H$^+$]: 223.081, Found: 223.1.

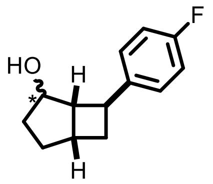

SI-18

7-(4-fluorophenyl)bicyclo[3.2.0]heptan-2-ol (SI-18): Following general procedure C using **SI-10** (200 mg, 0.97 mmol) with a 24 hour reaction time, **SI-18** was obtained (106 mg, 53% yield, 2:1 dr) after purification of the crude product by Flash Column Chromatography (Hexanes:Ethyl Acetate 1:0 to 6:4).

^{1}H NMR (400 MHz, $CDCl_3$) δ 7.26 – 7.17 (m, 3H), 7.03 – 6.95 (m, 3H), 4.32 (dt, *J* = 10.2, 6.7 Hz, 0.5H), 4.22 (d, *J* = 3.8 Hz, 1H), 3.49 (t, *J* = 10.7 Hz, 0.5H), 2.96 – 2.88 (m, 1H), 2.88 – 2.80 (m, 1H), 2.78 – 2.65 (m, 2H), 2.47 – 2.37 (m, 0.5H), 2.33 – 2.13 (m, 2H), 2.12 – 1.89 (m, 4.5H), 1.80 – 1.69 (m, 0.5H), 1.62 (dd, *J* = 12.6, 7.6 Hz, 1.5H), 1.43 (s, 1.5H).

^{13}C NMR (101 MHz, $CDCl_3$) δ 161.28 (d, J = 243.6 Hz), 130.52, 127.94 (d, J = 7.8 Hz), 115.21 (d, J = 21.1 Hz), 78.29, 59.68 (*minor*), 54.97, 38.97, 33.92, 33.39, 32.16, 30.27, 27.07 (*minor*).

FTIR: 3351, 2933, 2114, 1600, 1508, 1491, 1456, 1437, 1305, 1223, 1158, 1106, 1049, 1029 cm^{-1}.

HRMS (ESI): Calculated for $C_{14}H_{16}FO_3$ [M+HCOO$^+$]: 251.1106, Found: 251.2361.

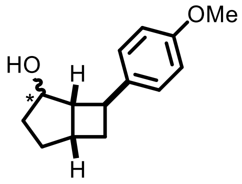

SI-19

7-(4-methoxyphenyl)bicyclo[3.2.0]heptan-2-ol (SI-19): Following general procedure D using crude **SI-11** (200 mg, 0.92 mmol) with a reaction time of 3 hours, **SI-19** was obtained (180 mg, 90%, 2:1 dr) after purification of the crude product by Flash Column Chromatography (Hexanes:Ethyl Acetate 1:0 to 6:4).

^{1}H NMR (400 MHz, $CDCl_3$) δ 7.25 – 7.14 (m, 3H), 6.90 – 6.81 (m, 3H), 4.30 (dt, J = 10.0, 6.7 Hz, 0.5H), 4.20 (d, J = 3.9 Hz, 1H), 3.79 (m, 4.5H), 3.45 (ddd, J = 9.8, 7.6, 4.8 Hz, 0.5H), 2.91 (dtd, J = 13.9, 7.0, 3.9 Hz, 1H), 2.81 (dt, J = 9.5, 6.5 Hz, 1H), 2.76 – 2.64 (m, 2H), 2.47 – 2.37 (m, 0.5H), 2.33 – 2.13 (m, 2.5H), 2.12 – 1.86 (m, 5H), 1.80 – 1.68 (m, 1H), 1.68 – 1.54 (m, 1.5H).

^{13}C NMR (101 MHz, $CDCl_3$) δ 157.87, 157.71 (*minor*), 139.40 (*minor*), 138.74, 127.71 (*minor*), 127.52, 113.93, 113.89 (*minor*), 78.34, 75.50 (*minor*), 55.45, 55.09, 50.06 (*minor*), 38.94, 33.86, 33.75 (*minor*), 33.35, 33.13 (*minor*), 33.08 (*minor*), 32.36 (*minor*), 32.25, 30.31, 29.69 (*minor*), 27.06 (*minor*).

FTIR: 3360, 2929, 1510, 1243, 1176, 1034 cm^{-1}.

HRMS (ESI): Calculated for $C_{14}H_{18}NaO_2$ [M+Na$^+$]: 241.1199, Found: 241.1199.

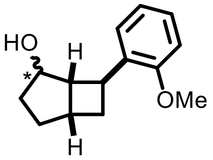

SI-20

7-(2-methoxyphenyl)bicyclo[3.2.0]heptan-2-ol (SI-20): Following general procedure C using **SI-12** (400 mg, 1.83 mmol), **SI-20** was obtained as an oil (400 mg, quant., >20:1 dr) and used in the next step without further purification.

^{1}H NMR (400 MHz, CDCl$_3$) δ 7.33 – 7.29 (m, 1H), 7.18 (td, J = 7.8, 1.7 Hz, 1H), 6.96 (td, J = 7.5, 1.0 Hz, 1H), 6.84 (dd, J = 8.1, 0.9 Hz, 1H), 4.28 (d, J = 3.9 Hz, 1H), 3.81 (m, 4H), 3.17 – 3.07 (m, 1H), 2.91 – 2.82 (m, 1H), 2.68 (t, J = 6.4 Hz, 1H), 2.38 (ddd, J = 12.5, 9.6, 7.9 Hz, 1H), 2.32 – 2.19 (m, 1H), 2.09 – 1.96 (m, 1H), 1.98 – 1.81 (m, 2H), 1.62 (dd, J = 12.7, 7.6 Hz, 1H).

^{13}C NMR (101 MHz, CDCl$_3$) δ 157.11, 133.87, 126.96, 126.56, 120.50, 110.31, 78.60, 55.38, 53.99, 34.24, 33.88, 33.38, 30.42, 29.97.

FTIR: 3354, 2927, 2114, 1598, 1490, 1462, 1436, 1288, 1240, 1174, 1106, 1048, 1029 cm^{-1}.

HRMS (ESI): Calculated for $C_{14}H_{18}NaO_2$ [M+Na$^+$]: 241.1199, Found 241.1194.

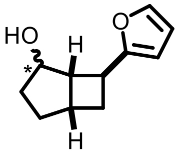

SI-21

7-(furan-2-yl)bicyclo[3.2.0]heptan-2-ol (SI-21): Following general procedure D using **SI-13** (210 mg, 1.18 mmol), **SI-21** was obtained (210 mg, quant., 2:1 dr) after purification by Flash Column Chromatography (Hexanes:Ethyl Acetate 1:0 to 6:4).

^{1}H NMR (400 MHz, CDCl$_3$) δ 7.32 (dd, J = 1.8, 0.8 Hz, 1.5H), 6.29 (dd, J = 3.2, 1.9 Hz, 1.5H), 6.02 (td, J = 2.4, 1.2 Hz, 1.5H), 4.34 – 4.25 (m, 0.5H), 4.19 (d, J = 3.9 Hz, 1H), 3.56 – 3.47 (m, 0.5H), 2.96 – 2.79 (m, 2.5H), 2.76 (t, J = 6.1 Hz, 1H), 2.74 – 2.66 (m, 0.5H), 2.49 (ddd, J = 12.3, 9.4, 5.8 Hz, 0.5H), 2.37 (ddd, J = 12.6, 9.5, 7.2 Hz, 1H), 2.20 – 1.82 (m, 7H), 1.71 (tt, J = 12.5, 7.2 Hz, 1.5H), 1.58 (dd, J = 12.5, 7.2 Hz, 2.5H).

^{13}C NMR (101 MHz, CDCl$_3$) δ 158.96, 141.23, 141.17 (*minor*), 110.22, 110.20 (*minor*), 103.84, 103.72 (*minor*), 77.97, 74.98 (*minor*), 52.60, 47.58 (*minor*), 33.59, 33.46 (*minor*), 32.83, 32.04 (*minor*), 30.78 (*minor*), 30.32, 29.73, 29.67 (*minor*), 27.93 (*minor*), 27.06.

FTIR: 3363.74, 2930, 2112, 1712, 1640, 1593, 1506, 1442, 1330, 1237, 1148, 1075, 1008 cm^{-1}.

HRMS (ESI): Calculated for $C_{11}H_{15}O_2$ [M+H$^+$]: 179.1067, Found 179.1062.

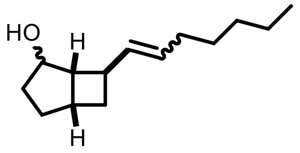

SI-22

7-(hept-1-en-1-yl)bicyclo[3.2.0]heptan-2-ol (SI-22): Following general procedure D using **SI-14** (2.5000 g, 12.00 mmol) which was filtered through a short plug of silica with MeCN immediately before use, the desired product **SI-22** was obtained as a yellow oil and used without further purification.

^{1}H NMR (500 MHz, CDCl$_3$) δ 5.90 – 5.70 (m, 0.13H), 5.56 (dt, *J* = 17.1, 7.0 Hz, 0.6H), 5.43 – 5.29 (m, 0.54H), 5.24 (dq, *J* = 13.4, 7.0 Hz, 0.25H), 5.08 – 4.92 (m, 0.2H), 4.29 – 4.15 (m, 0.29H), 4.09 (dd, *J* = 9.9, 3.9 Hz, 0.41H), 3.18 – 3.06 (m, 0.12H), 2.88 – 2.42 (m, 1.46H), 2.42 – 2.33 (m, 0.56H), 2.22 (tt, *J* = 12.5, 6.7 Hz, 0.45H), 2.16 – 1.60 (m, 6.02H), 1.60 – 1.41 (m, 1.68H), 1.41 – 1.19 (m, 6.24H), 1.09 (s, 0.58H), 0.88 (td, *J* = 7.1, 2.6 Hz, 3H).

3. DMP Oxidations

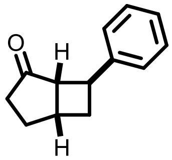

SI-23

7-phenylbicyclo[3.2.0]heptan-2-one (SI-23): Following General procedure F with **SI-15** (83 mmol), **SI-23** was obtained (>20:1 dr) as a yellow oil and used without further purification.

^{1}H NMR (500 MHz, CDCl$_3$) δ 7.35 – 7.31 (m, 2H), 7.30 – 7.27 (m, 2H), 7.25 – 7.18 (m, 1H), 3.52 (dt, J = 8.4, 6.1 Hz, 1H), 3.04 (dp, J = 12.5, 4.3 Hz, 1H), 2.89 (ddt, J = 7.0, 5.8, 1.2 Hz, 1H), 2.78 (dt, J = 17.8, 8.7 Hz, 1H), 2.58 (dddd, J = 12.4, 8.7, 7.1, 1.2 Hz, 1H), 2.47 – 2.22 (m, 3H), 2.01 (dddd, J = 13.1, 9.1, 5.6, 3.3 Hz, 1H).

^{13}C NMR (126 MHz, CDCl$_3$) δ 220.86, 144.26, 128.53, 126.38, 126.33, 52.80, 40.67, 38.07, 33.00, 32.01, 27.88.

FTIR: 2935.17 (w), 1724.36 (s), 1493.29 (w), 1453.80 (w), 1155.22 (m), 746.33 (m), 696.80 (s), 523.29 (w), 443.11 (w).

HRMS (EI): Calculated for $C_{13}H_{14}O$ [M^+]: 186.1038; Found 186.1039.

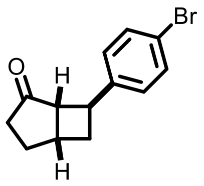

SI-24

7-(4-bromophenyl)bicyclo[3.2.0]heptan-2-one (SI-24): Following General Procedure F using crude **SI-16** (770 mg, 2.88 mmol), **SI-24** was obtained (500 mg, 66% yield, >20:1 dr) as an oil and used without further purification.

^{1}H NMR (300 MHz, CDCl$_3$) δ 7.43 (d, *J* = 8.4 Hz, 2H), 7.14 (dd, *J* = 8.6, 0.6 Hz, 2H), 3.53 – 3.39 (m, 1H), 3.02 (ddt, *J* = 12.3, 8.5, 4.2 Hz, 1H), 2.87 – 2.66 (m, 2H), 2.59 – 2.18 (m, 4H), 2.00 (dddd, *J* = 13.5, 9.4, 5.6, 3.4 Hz, 1H).

^{13}C NMR (75 MHz, CDCl$_3$) δ 220.62, 143.33, 131.67, 128.28, 120.19, 52.77, 40.26, 38.21, 32.99, 32.06, 27.91.

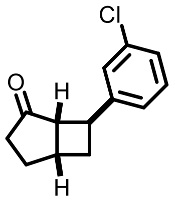

SI-25

7-(3-chlorophenyl)bicyclo[3.2.0]heptan-2-one (SI-25): Following General Procedure F using crude **SI-17** (1.90 g, 8.53 mmol), **SI-25** was obtained (1.88 g, quant., >20:1 dr) as an oil and used without further purification.

^{1}H NMR (300 MHz, CDCl$_3$) δ 7.28 – 7.23 (m, 2H), 7.21 – 7.13 (m, 2H), 3.51 – 3.44 (m, 1H), 3.04 (ddt, *J* = 11.6, 7.6, 3.8 Hz, 1H), 2.86 (dd, *J* = 8.2, 4.6 Hz, 1H), 2.81 – 2.69 (m, 1H), 2.54 (dddd, *J* = 12.4, 8.6, 7.1, 1.2 Hz, 1H), 2.49 – 2.22 (m, 3H), 2.00 (dddd, *J* = 13.3, 9.2, 5.7, 3.5 Hz, 1H).

^{13}C NMR (75 MHz, CDCl$_3$) δ 220.84, 146.34, 134.50, 129.92, 126.64, 124.82, 52.62, 40.41, 38.16, 32.94, 32.10, 27.89.

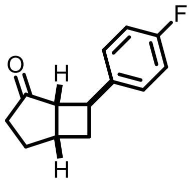

SI-26

7-(4-fluorophenyl)bicyclo[3.2.0]heptan-2-one (SI-26): Following general procedure F using **SI-18** (200 mg, 0.97 mmol), **SI-26** was obtained (198 mg, quant., >20:1 dr) as an oil and used without further purification.

^{1}H NMR (300 MHz, CDCl$_3$) δ 7.27 – 7.17 (m, 2H), 7.00 (t, *J* = 8.7 Hz, 2H), 3.48 (q, *J* = 7.1 Hz, 1H), 3.02 (ddq, *J* = 12.0, 8.3, 4.0 Hz, 1H), 2.87 – 2.80 (m, 1H), 2.80 – 2.67 (m, 1H), 2.58 – 2.20 (m, 4H), 2.00 (dddd, *J* = 13.5, 9.4, 5.6, 3.5 Hz, 1H).

^{19}F NMR (282 MHz, CDCl$_3$) δ -116.83.

^{13}C NMR (75 MHz, CDCl$_3$) δ 220.77, 161.56 (d, *J* = 244.4 Hz), 140.01 (d, *J* = 3.1 Hz), 127.97 (d, *J* = 7.9 Hz), 115.37 (d, *J* = 21.3 Hz), 53.07, 40.16, 38.24, 33.26, 31.97, 27.93.

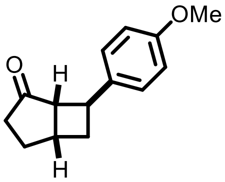

SI-27

7-(4-methoxyphenyl)bicyclo[3.2.0]heptan-2-one (SI-27): Following general procedure F using **SI-19** (180 mg, 0.83 mmol), **SI-19** was obtained (178 mg, quant., >20:1 dr) as an oil and used without further purification.

^{1}H NMR (400 MHz, CDCl$_3$) δ 7.22 – 7.16 (m, 2H), 6.89 – 6.83 (m, 2H), 3.79 (s, 3H), 3.50 – 3.42 (m, 1H), 3.02 (qt, J = 8.1, 3.9 Hz, 1H), 2.86 – 2.81 (m, 1H), 2.78 (m, 1H), 2.53 (dddd, J = 12.3, 8.6, 7.1, 1.2 Hz, 1H), 2.45 – 2.20 (m, 3H), 2.00 (dddd, J = 13.1, 9.1, 5.5, 3.4 Hz, 1H).

^{13}C NMR (101 MHz, CDCl$_3$) δ 221.16, 158.25, 136.50, 127.54, 114.05, 55.45, 53.30, 40.27, 38.20, 33.40, 32.02, 28.02.

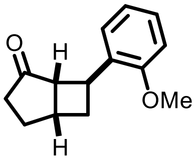

SI-28

7-(2-methoxyphenyl)bicyclo[3.2.0]heptan-2-one (SI-28): Following general procedure F using **SI-20** (500 mg, 2.29 mmol), **SI-28** was obtained (495 mg, quant., >20:1 dr) and used without further purification.

^{1}H NMR (400 MHz, CDCl$_3$) δ 7.30 (d, *J* = 7.5 Hz, 1H), 7.19 (qd, *J* = 8.5, 1.6 Hz, 1H), 6.95 (tdd, *J* = 7.5, 3.5, 1.0 Hz, 1H), 6.84 (d, *J* = 8.1 Hz, 1H), 3.80 (s, 3H), 3.75 (tt, *J* = 10.7, 4.8 Hz, 1H), 3.00 (ddq, *J* = 11.6, 7.7, 3.4 Hz, 1H), 2.92 (t, *J* = 6.4 Hz, 1H), 2.89 – 2.78 (m, 1H), 2.60 – 2.49 (m, 1H), 2.46 – 2.19 (m, 3H), 2.07 – 1.96 (m, 1H).

^{13}C NMR (101 MHz, CDCl$_3$) δ 221.16, 157.21, 131.98, 127.67, 126.73, 120.50, 110.52, 55.41, 51.41, 37.95, 36.10, 32.84, 31.97, 28.01.

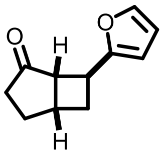

SI-29

7-(furan-2-yl)bicyclo[3.2.0]heptan-2-one (SI-29): Following general procedure F using **SI-21** (550 mg, 3.10 mmol), **SI-29** was obtained (544 mg, quant., >20:1 dr) and used without further purification.

^{1}H NMR (300 MHz, CDCl$_3$) δ 7.35 (dd, *J* = 1.9, 0.8 Hz, 1H), 6.30 (dd, *J* = 3.2, 1.9 Hz, 1H), 6.08 (dt, *J* = 3.2, 0.7 Hz, 1H), 3.47 – 3.39 (m, 1H), 3.11 (ddt, *J* = 9.0, 6.3, 3.5 Hz, 1H), 2.95 – 2.87 (m, 1H), 2.81 – 2.66 (m, 1H), 2.61 (dddd, *J* = 12.4, 8.9, 6.3, 1.7 Hz, 1H), 2.47 – 2.34 (m, 1H), 2.32 – 2.13 (m, 2H), 1.95 (dddd, *J* = 13.8, 9.3, 4.7, 2.8 Hz, 1H).

^{13}C NMR (75 MHz, CDCl$_3$) δ 220.63, 156.79, 141.84, 110.38, 105.15, 50.92, 37.64, 34.21, 32.66, 31.58, 27.76.

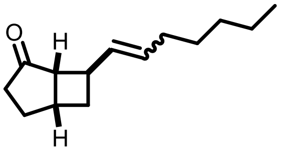

SI-30

7-(hept-1-en-1-yl)bicyclo[3.2.0]heptan-2-one (SI-30): Following general procedure F using **SI-22** (12.00 mmol), **SI-30** was obtained and used without further purification.

^{1}H NMR (500 MHz, CDCl$_3$) δ 5.78 (d, *J* = 17.4 Hz, 0.15H), 5.65 – 5.52 (m, 0.7H), 5.49 – 5.30 (m, 0.9H), 5.25 (td, *J* = 10.8, 5.9 Hz, 0.14H), 5.09 – 4.92 (m, 0.27H), 4.09 (dd, *J* = 10.0, 3.9 Hz, 0.13H), 3.25 – 3.15 (m, 0.1H), 3.15 – 3.07 (m, 0.23H), 3.02 – 2.87 (m, 0.66H), 2.86 – 2.63 (m, 1.24H), 2.57 (t, *J* = 6.1 Hz, 0.38H), 2.55 – 2.26 (m, 1.66H), 2.26 – 2.04 (m, 2.21H), 2.04 – 1.81 (m, 3.17H), 1.81 – 1.41 (m, 1.39H), 1.41 – 1.16 (m, 6.28H), 0.97 – 0.81 (m, 3H).

4. Formation of the α-Diazoketones

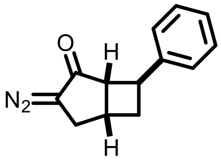

SI-31

3-diazo-7-phenylbicyclo[3.2.0]heptan-2-one (SI-31): Following General Procedure G with crude **SI-23** (30 mmol), **SI-31** was obtained after column chromatography (EtOAc 30% to 40% in Hexanes) as a bright yellow oil (2.7527 g, 43% over 3 steps).

^{1}H NMR (500 MHz, CDCl$_3$) δ 7.36 – 7.27 (m, 4H), 7.24 – 7.19 (m, 1H), 3.69 – 3.61 (m, 1H), 3.35 (dd, *J* = 13.6, 8.2 Hz, 1H), 3.15 – 3.02 (m, 2H), 2.96 (dd, *J* = 13.6, 1.9 Hz, 1H), 2.65 – 2.54 (m, 1H), 2.48 – 2.38 (m, 1H).

^{13}C NMR (126 MHz, CDCl$_3$) δ 201.41, 144.12, 128.70, 126.54, 126.50, 59.67, 54.49, 42.74, 34.25, 31.14, 29.70.

FTIR: 2931.43 (w), 2071.88 (s), 1653.72 (s), 1494.06 (m), 1454.26 (m), 1326.87 (s), 1292.09 (m), 1229.69 (s), 1028.09 (m), 935.64 (m), 748.59 (s), 696.97 (s), 639.48 (m), 526.66 (m).

HRMS (EI): Calculated for $C_{13}H_{12}O$ [M-N_2^+]: 184.0883, Found: 184.0875.

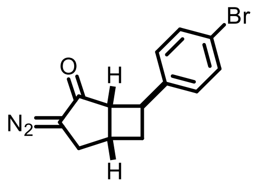

SI-32

7-(4-bromophenyl)bicyclo[3.2.0]heptan-2-one (SI-32): Following General Procedure G with **SI-24** (500 mg, 1.90 mmol), **SI-32** was obtained after column chromatography (Hexanes:Ethyl Acetate 1:0 to 3:2) as a bright yellow oil (180 mg, 33% yield).

^{1}H NMR (300 MHz, CDCl$_3$) δ 7.44 (d, *J* = 8.4 Hz, 2H), 7.16 (dd, *J* = 8.6, 0.5 Hz, 2H), 3.58 (ddd, *J* = 9.1, 6.0, 3.1 Hz, 1H), 3.41 – 3.27 (m, 1H), 3.11 – 3.01 (m, 2H), 2.95 (dd, *J* = 14.2, 1.2 Hz, 1H), 2.59 – 2.47 (m, 1H), 2.47 – 2.36 (m, 1H).

^{13}C NMR (75 MHz, CDCl$_3$) δ 200.97, 143.12, 131.74, 128.33, 120.25, 59.73, 54.31, 42.21, 34.11, 31.06, 29.61.

FTIR: 2930, 2077, 1727, 1657, 1510, 1488, 1457, 1328, 1293, 1231, 1175, 1072, 1009 cm^{-1}.

HRMS (ESI): Calculated for $C_{13}H_{12}BrN_2O$ [M+H$^+$]: 291.0128, Found: 291.0126.

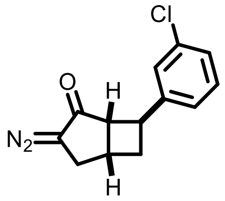

SI-33

7-(3-chlorophenyl)-3-diazobicyclo[3.2.0]heptan-2-one (SI-33): Following General Procedure G with **SI-25** (1.88 g, 8.52 mmol), **SI-33** was obtained after column chromatography (Hexanes:Ethyl Acetate 1:0 to 3:2) as a bright yellow oil (510 mg, 24% yield).

^{1}H NMR (400 MHz, CDCl$_3$) δ 7.32 – 7.26 (m, 2H), 7.25 – 7.17 (m, 2H), 3.63 (ddd, *J* = 9.1, 5.9, 3.1 Hz, 1H), 3.42 – 3.32 (m, 1H), 3.16 – 3.05 (m, 2H), 2.98 (dd, *J* = 14.1, 1.2 Hz, 1H), 2.65 – 2.53 (m, 1H), 2.51 – 2.42 (m, 1H).

FTIR: 2932, 2078, 1727, 1660, 1509, 1468, 1436, 1328, 1292, 1238, 1023 cm^{-1}.

LRMS (ESI): Calculated for $C_{13}H_{11}ClN_2OK$ [M+K$^+$]: 265.056, Found: 265.1.

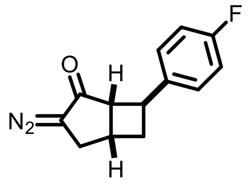

SI-34

7-(3-chlorophenyl)-3-diazobicyclo[3.2.0]heptan-2-one (SI-34): Following General Procedure G with **SI-26** (25 mg, 0.12 mmol), **SI-34** was obtained after purification by Flash column chromatography (Hexanes:Ethyl Acetate 1:0 to 6:4) as a bright yellow oil (15 mg, 53% yield).

^{1}H NMR (300 MHz, CDCl$_3$) δ 7.28 – 7.19 (m, 2H), 7.00 (t, *J* = 8.7 Hz, 2H), 3.60 (ddd, *J* = 9.1, 6.0, 3.1 Hz, 1H), 3.40 – 3.28 (m, 1H), 3.12 – 3.01 (m, 2H), 2.95 (dd, *J* = 14.2, 1.3 Hz, 1H), 2.61 – 2.48 (m, 1H), 2.46 – 2.35 (m, 1H).

^{13}C NMR (75 MHz, CDCl$_3$) δ 201.20, 161.61 (d, *J* = 244.5 Hz), 139.81, 128.06 (d, *J* = 8.0 Hz), 115.48 (d, *J* = 21.3 Hz), 59.76, 54.64, 42.14, 34.41, 31.12, 29.57.

FTIR: 2934, 2078, 1655, 1602, 1508, 1457, 1329, 1293, 1229, 1157, 1058, 1013 cm^{-1}.

HRMS (ESI): Calculated for $C_{13}H_{11}FN_2NaO$ [M+Na$^+$]: 253.0748, Found: 253.0742.

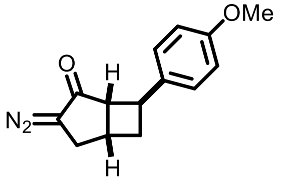

SI-35

3-diazo-7-(4-methoxyphenyl)bicyclo[3.2.0]heptan-2-one (SI-35): Following General Procedure G with **SI-27** (150 mg, 0.69 mmol), **SI-35** was obtained after column chromatography (Hexanes:Ethyl Acetate 1:0 to 6:4) as a bright yellow oil (35 mg, 18% yield).

^{1}H NMR (400 MHz, CDCl$_3$) 7.23 – 7.18 (m, 2H), 6.90 – 6.84 (m, 2H), 3.80 (s, 3H), 3.59 (ddd, J = 9.1, 5.9, 3.1 Hz, 1H), 3.40 – 3.27 (m, 1H), 3.05 (dtt, J = 7.0, 4.6, 1.8 Hz, 2H), 2.95 (dd, J = 14.1, 1.3 Hz, 1H), 2.60 – 2.50 (m, 1H), 2.45 – 2.36 (m, 1H).

^{13}C NMR (101 MHz, CDCl$_3$) δ 201.56, 158.30, 136.33, 127.60, 114.11, 55.46, 54.87, 42.20, 34.53, 31.18, 29.65.

FTIR: 2933, 2080, 1643, 1606, 1508, 1457, 1330, 1294, 1222, 1176, 1157, 1095, 1031 cm^{-1}.

HRMS (ESI): Calculated for $C_{14}H_{14}NaO_2$ [$M+Na^+$]: 265.0947, Found: 265.0942.

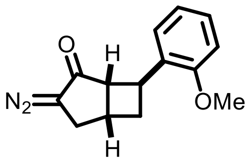

SI-36

3-diazo-7-(2-methoxyphenyl)bicyclo[3.2.0]heptan-2-one (SI-36): Following General Procedure G with crude **SI-28** (495 mg, 2.29 mmol), **SI-36** was obtained after column chromatography (Hexanes:Ethyl Acetate 1:0 to 6:4) as a bright yellow oil (250 mg, 45% yield).

^{1}H NMR (400 MHz, CDCl$_3$) δ 7.34 – 7.27 (m, 1H), 7.24 – 7.17 (m, 1H), 6.95 (tdd, J = 7.5, 4.4, 1.2 Hz, 1H), 6.84 (ddd, J = 8.2, 5.2, 1.2 Hz, 1H), 3.91 – 3.83 (m, 1H), 3.81 (d, J = 3.3 Hz, 3H), 3.34 (dd, J = 13.5, 8.6 Hz, 1H), 3.21 (s, 1H), 3.03 (ddddd, J = 8.7, 7.7, 5.3, 2.2, 1.2 Hz, 1H), 2.97 (dd, J = 13.5, 2.1 Hz, 1H), 2.52 (dddd, J = 12.6, 8.9, 6.3, 1.8 Hz, 1H), 2.38 (dddd, J = 11.9, 10.0, 4.7, 3.8 Hz, 1H).

^{13}C NMR (101 MHz, CDCl$_3$) δ 202.00, 157.24, 131.88, 127.78, 126.78, 120.52, 110.63, 59.71, 55.46, 52.82, 38.21, 34.18, 31.16, 29.60.

FTIR: 2932, 2083, 1644, 1601, 1508, 1492, 1457, 1438, 1329, 1221, 1157, 1094, 1052, 1029 cm^{-1}.

HRMS (ESI): Calculated for $C_{14}H_{15}N_2O_2$ [$M+H^+$]: 243.1128, Found: 243.1127.

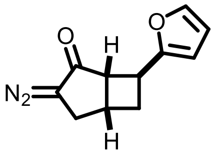

SI-37

3-diazo-7-(furan-2-yl)bicyclo[3.2.0]heptan-2-one (SI-37): Following General Procedure G with crude **SI-29** (100 mg, 0.56 mmol), **SI-29** was obtained after column chromatography (Hexanes:Ethyl Acetate 1:0 to 6:4) as a bright yellow oil (35 mg, 50% yield).

^{1}H NMR (300 MHz, CDCl$_3$) δ 7.34 (dd, *J* = 1.9, 0.8 Hz, 1H), 6.29 (dd, *J* = 3.2, 1.9 Hz, 1H), 6.08 (dt, *J* = 3.2, 0.7 Hz, 1H), 3.57 (ddd, *J* = 9.1, 5.0, 2.5 Hz, 1H), 3.35 – 3.24 (m, 1H), 3.22 – 3.09 (m, 2H), 2.91 – 2.82 (m, 1H), 2.59 (dddd, *J* = 11.2, 7.3, 4.8, 3.5 Hz, 1H), 2.41 – 2.28 (m, 1H).

^{13}C NMR (75 MHz, CDCl$_3$) δ 200.80, 156.75, 141.77, 110.36, 105.14, 59.76, 52.69, 36.06, 32.86, 30.93, 30.36.

FTIR: 2934, 2078, 1657, 1599, 1508, 1491, 1457, 1328, 1292, 1230, 1158, 1054, 1029 cm^{-1}.

HRMS (ESI): Calculated for $C_{11}H_{10}N_2NaO_2$ [$M+Na^+$]: 225.0634, Found: 225.0630.

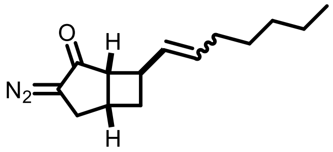

SI-38

3-diazo-7-(hept-1-en-1-yl)bicyclo[3.2.0]heptan-2-one (SI-38): Following General Procedure G with crude **SI-30** (10.00 mmol), an isomeric mixture of products **SI-38** was obtained after removal of sulfonamide byproduct via MPLC (gradient: 0% EtOAc in Hexanes to 5% EtOAc over 1 min., 1 min. hold, 5% to 10% over 1 min., 1 min. hold, 10% to 15% over 1 min., 1 min. hold, 15% to 20% over 1 min., 1 min. hold, 20% to 85% over 5.1 min., 100% EtOAc over 16.5 min.) as a bright yellow oil.

^{1}H NMR (500 MHz, $CDCl_3$) δ 5.62 (dd, *J* = 13.9, 7.5 Hz, 0.8H), 5.47 (dq, *J* = 16.7, 8.2 Hz, 0.49H), 5.42 – 5.33 (m, 0.42H), 3.26 (ddd, *J* = 13.1, 8.3, 3.7 Hz, 1.16H), 3.04 – 2.88 (m, 1.26H), 2.83 (dd, *J* = 13.6, 2.4 Hz, 1.33H), 2.77 (dt, *J* = 6.8, 2.6 Hz, 0.46H), 2.29 – 2.11 (m, 1.86H), 1.99 (p, *J* = 7.0 Hz, 1.9H), 1.42 – 1.21 (m, 6H), 0.88 (q, *J* = 6.5 Hz, 3H).

5. Bicyclo[2.2.0]hexane Formation

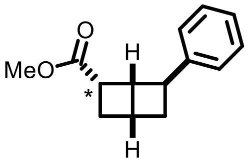

5a

methyl 6-phenylbicyclo[2.2.0]hexane-2-carboxylate (5a): Following General Procedure H with **SI-31** (3.6 g, 17 mmol), **5a** was obtained after MPLC (gradient: 0 to 5% EtOAc in Hexanes over 9 min., 3 min. hold, 5 to 100% EtOAc over 2 min., 6 min. hold) to yield a faint yellow oil (2.6119 g, 71% yield, 4:1 dr) with an efficiency of 5 mmol h^{-1}.

^{1}H NMR (500 MHz, $CDCl_3$) δ 7.34 – 7.28 (m, 2.5H), 7.25 – 7.15 (m, 3.75H), 3.83 (td, J = 8.3, 4.0 Hz, 1H), 3.72 (s, 3H), 3.69 (s, 0.75H), 3.67 – 3.62 (m, 0.25H), 3.56 (ddd, J = 10.1, 9.0, 7.7 Hz, 1H), 3.34 (ddd, J = 8.5, 6.2, 2.5 Hz, 0.25H), 3.15 (dddd, J = 7.8, 5.5, 4.0, 1.3 Hz, 1H), 3.04 (dtt, J = 5.0, 2.5, 1.1 Hz, 0.25H), 2.89 (tdt, J = 8.0, 5.4, 2.7 Hz, 0.25H), 2.80 (dddt, J = 12.6, 7.7, 6.2, 1.3 Hz, 0.25H), 2.76 – 2.69 (m, 1H), 2.69 – 2.63 (m, 2H), 2.63 – 2.48 (m, 1.5H), 2.46 – 2.39 (m, 1.25H).

^{13}C NMR (126 MHz, $CDCl_3$) δ 176.05 (*minor*), 174.43, 146.35 (*minor*), 146.06, 128.59 (*minor*), 128.47, 126.53 (*minor*), 126.50, 126.15 (*minor*), 125.92, 51.91 (*minor*), 51.56, 46.68, 46.41 (*minor*), 45.87 (*minor*), 44.18 (*minor*), 40.59, 39.66, 36.27 (*minor*), 35.76, 30.96 (*minor*), 30.89 (*minor*), 29.70, 28.82.

FTIR: 2949.40 (m), 1728.20 (s), 1601.88 (w), 1493.99 (m), 1434.21 (m), 1338.47 (m), 1196.14 (s), 1071.00 (m), 1047.94 (m), 836.05 (w), 742.36 (s), 697.53 (s), 520.30 (w).

HRMS (APCI): Calculated for $C_{14}H_{17}O_2$ [M+H^+]: 217.1223, Found: 217.1224.

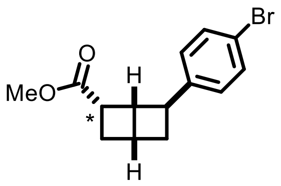

8

methyl 6-(4-bromophenyl)bicyclo[2.2.0]hexane-2-carboxylate (8): Following General Procedure I with **SI-32** (100 mg, 0.343 mmol), **8** was obtained after column chromatography (Hexanes:Ethyl Acetate 1:0 to 9:1) as a faint oil (60 mg, 59% yield, 3:1 dr).

^{1}H NMR (300 MHz, $CDCl_3$) δ 7.45 – 7.37 (m, 2.66H), 7.13 – 7.04 (m, 2.66H), 3.77 (td, *J* = 8.3, 4.0 Hz, 1H), 3.71 (s, 3H), 3.69 (s, 1H), 3.62 – 3.48 (m, 1.33H), 3.32 (ddd, *J* = 8.3, 6.1, 2.4 Hz, 0.33H), 3.09 (ddd, *J* = 8.6, 5.9, 4.1 Hz, 1H), 2.99 (ddt, *J* = 5.2, 2.5, 1.3 Hz, 0.33H), 2.93 – 2.84 (m, 0.33H), 2.82 – 2.36 (m, 6H).

^{13}C NMR (75 MHz, $CDCl_3$) δ 175.88 (*minor*), 174.30, 145.00 (*minor*), 144.15, 131.61 (*minor*), 131.49, 128.32, 120.12 (*minor*), 119.59, 51.96 (*minor*), 51.60, 46.59, 46.19 (*minor*), 45.26 (*minor*), 44.11 (*minor*), 40.13, 39.59, 36.18 (*minor*), 35.63, 30.93 (*minor*), 30.90 (*minor*), 29.77, 28.74.

FTIR: 2950, 1728, 1596, 1570, 1477, 1434, 1335, 1197, 1174, 1078, 1051 cm^{-1}.

HRMS (ESI): Calculated for $C_{14}H_{15}BrNaO_2$ [M+Na$^+$]: 317.0148, Found: 317.0138.

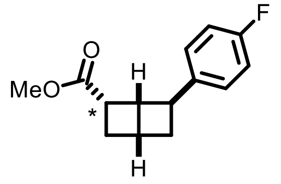

9

methyl 6-(4-fluorophenyl)bicyclo[2.2.0]hexane-2-carboxylate (9): Following General Procedure I with **SI-34** (15 mg, 0.065 mmol), **9** was obtained after column chromatography (Hexanes:Ethyl Acetate 1:0 to 9:1) as a faint oil (15 mg, 49% yield, 3:1 dr).

^{1}H NMR (400 MHz, $CDCl_3$) δ 7.21 – 7.12 (m, 2.66H), 7.02 – 6.94 (m, 2.66H), 3.79 (td, J = 8.4, 4.3 Hz, 1H), 3.72 (s, 3H), 3.69 (s, 1H), 3.65 – 3.50 (m, 1.33H), 3.32 (ddd, J = 8.4, 6.2, 2.4 Hz, 0.33H), 3.12 – 3.06 (m, 1H), 2.99 (ddt, J = 5.1, 2.5, 1.3 Hz, 0.33H), 2.87 (ddq, J = 10.5, 5.3, 2.6 Hz, 0.33H), 2.83 – 2.74 (m, 0.33H), 2.74 – 2.36 (m, 6H).

^{13}C NMR (101 MHz, $CDCl_3$) δ 175.96 (*minor*), 174.37, 161.27 (d, *J* = 243.7 Hz), 141.67 (d, J = 3.2 Hz), 127.89 (d, J = 7.8 Hz), 115.28 (d, J = 21.2 Hz, *minor*), 115.16 (d, J = 21.2 Hz), 51.95 (*minor*), 51.59, 46.79, 46.50 (*minor*), 45.14 (*minor*), 44.12 (*minor*), 39.97, 39.60, 36.43 (*minor*), 35.88, 30.91 (*minor*), 30.83 (*minor*), 29.77, 28.66.

FTIR: 2951, 1727, 1602, 1509, 1435, 1341, 1243, 1220, 1197, 1175, 1158, 1071, 1042 cm^{-1}.

LRMS (ESI): Calculated for $C_{14}H_{15}FO_2$ [M-H$^+$]: 233.106, Found: 233.2.

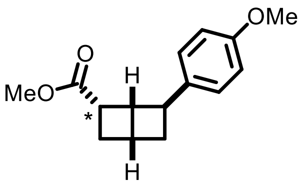

10

methyl 6-(4-methoxyphenyl)bicyclo[2.2.0]hexane-2-carboxylate (10): Following General Procedure I with **SI-35** (35 mg, 0.065 mmol), **10** was obtained after column chromatography (Hexanes:Ethyl Acetate 1:0 to 9:1) as a faint oil (30 mg, 45% yield, 3:1 dr).

^{1}H NMR (400 MHz, $CDCl_3$) δ 7.14 (dd, J = 15.3, 8.7 Hz, 2.66H), 6.85 (dt, J = 9.7, 3.0 Hz, 2.66H), 3.79 (d, J = 1.7 Hz, 4H), 3.78 – 3.72 (m, 1H), 3.71 (s, 3H), 3.68 (s, 1H), 3.61 – 3.50 (m, 1.33H), 3.31 (ddd, J = 8.3, 6.1, 2.4 Hz, 0.33H), 3.12 – 3.05 (m, 1H), 3.00 – 2.96 (m, 0.33H), 2.86 (dt, J = 5.2, 2.6 Hz, 0.33H), 2.82 – 2.75 (m, 0.33H), 2.75 – 2.35 (m, 6H).

^{13}C NMR (101 MHz, $CDCl_3$) δ 176.11 (*minor*), 174.48, 158.00 (*minor*), 157.87, 138.59 (*minor*), 138.26, 127.51 (*minor*), 127.48, 113.98 (*minor*), 113.89, 55.45, 51.90 (*minor*), 51.55, 46.88, 46.72 (*minor*), 45.17 (*minor*), 44.16 (*minor*), 39.96, 39.63, 36.48 (*minor*), 35.99, 30.87 (*minor*), 30.83 (*minor*), 29.71, 28.66.

FTIR: 2950, 1727, 1610, 1510, 1435, 1336, 1294, 1242, 1196, 1175, 1071, 1033 cm^{-1}.

HRMS (ESI): Calculated for $C_{15}H_{18}NaO_3$ [$M+Na^+$]: 269.1148, Found: 269.1149.

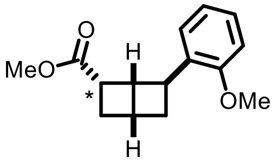

11

methyl 6-(2-methoxyphenyl)bicyclo[2.2.0]hexane-2-carboxylate (11): Following General Procedure I with **SI-36** (260 mg, 1.1 mmol), **11** was obtained after column chromatography (Hexanes:Ethyl Acetate 1:0 to 9:1) as a faint oil (100 mg, 38% yield, 3:1 dr).

^{1}H NMR (400 MHz, $CDCl_3$) δ 7.28 – 7.13 (m, 2.66H), 6.98 – 6.90 (m, 1.33H), 6.87 – 6.78 (m, 1.33H), 4.13 (td, J = 8.4, 4.1 Hz, 1H), 3.94 (td, J = 7.9, 3.1 Hz, 0.33H), 3.81 (s, 1H), 3.77 (s, 3H), 3.70 (d, J = 3.8 Hz, 4H), 3.53 (dt, J = 10.4, 8.3 Hz, 1H), 3.32 (ddd, J = 7.9, 5.6, 2.1 Hz, 0.33H), 3.19 – 3.13 (m, 1H), 3.01 (dt, J = 5.3, 2.5 Hz, 0.33H), 2.88 – 2.43 (m, 5.65H), 2.38 (ddd, J = 12.0, 8.3, 1.2 Hz, 1H).

^{13}C NMR (101 MHz, $CDCl_3$) δ 176.39 (*minor*), 174.22, 157.09, 156.98 (*minor*), 133.77 (*minor*), 133.71, 127.11 (*minor*), 126.99, 126.85, 126.45 (*minor*), 120.51 (*minor*), 120.42, 110.38 (*minor*), 110.34, 55.43 (*minor*), 55.26, 51.83 (*minor*), 51.47, 45.78 (*minor*), 45.75, 44.42 (*minor*), 40.50 (*minor*), 39.70, 35.21, 34.97, 34.34 (*minor*), 31.00 (*minor*), 30.60 (*minor*), 29.25, 29.16.

FTIR: 2949, 1726, 1599, 1491, 1461, 1434, 1348, 1241, 1194, 1172, 1109, 1070, 1043, 1027 cm^{-1}.

HRMS (ESI): Calculated for $C_{15}H_{18}NaO_3$ [$M+Na^+$]: 269.1148, Found: 269.1148.

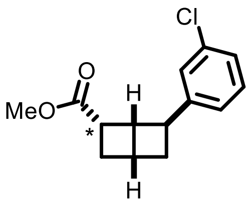

12

methyl 6-(3-chlorophenyl)bicyclo[2.2.0]hexane-2-carboxylate (12): Following General Procedure I with **SI-33** (580 mg, 2.35 mmol), **12** was obtained after column chromatography (Hexanes:Ethyl Acetate 1:0 to 9:1) as a faint oil (200 mg, 34% yield, 4:1 dr).

^{1}H NMR (400 MHz, $CDCl_3$) δ 7.25 – 7.05 (m, 5H), 3.80 (td, *J* = 8.3, 4.0 Hz, 1H), 3.71 (m, 3.75H), 3.56 (m, 1.25H), 3.32 (ddd, *J* = 8.5, 6.2, 2.5 Hz, 0.25H), 3.12 (dddd, *J* = 8.1, 5.3, 4.1, 1.3 Hz, 1H), 3.02 (dtt, *J* = 5.0, 2.5, 1.2 Hz, 0.25H), 2.88 (dtt, *J* = 7.9, 5.3, 2.7 Hz, 0.25H), 2.83 – 2.37 (m, 6H).

^{13}C NMR (101 MHz, $CDCl_3$) δ 174.28, 148.08, 134.34, 129.85 (*minor*), 129.72, 126.76 (*minor*), 126.73, 126.30 (*minor*), 126.08, 124.75 (*minor*), 124.74, 51.99 (*minor*), 51.64, 46.50, 46.11 (*minor*), 45.48 (*minor*), 44.11 (*minor*), 40.33, 39.58, 36.09 (*minor*), 35.57, 30.95 (*minor*), 30.93 (*minor*), 29.87 (*minor*), 29.76, 28.81.

FTIR: 2950, 1727, 1596, 1570, 1477, 1434, 1332, 1197, 1174, 1078, 1051 cm^{-1}.

HRMS (ESI): Calculated for $C_{14}H_{15}ClNaO_2$ [M+Na$^+$]: 273.0653, Found: 273.0653.

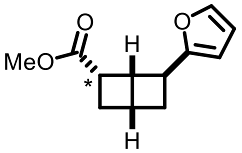

13

methyl 6-(furan-2-yl)bicyclo[2.2.0]hexane-2-carboxylate (13): Following General Procedure I with **SI-37** (30 mg, 0.15 mmol), **13** was obtained after column chromatography (Hexanes:Ethyl Acetate 1:0 to 9:1) as a as a faint oil (10 mg, 33% yield, 2.5:1 dr).

^{1}H NMR (400 MHz, $CDCl_3$) δ 7.39 – 7.31 (m, 1.4H), 6.28 (dd, J = 3.2, 1.9 Hz, 1.4H), 6.14 – 5.98 (m, 1.4H), 3.79 (td, J = 8.1, 3.8 Hz, 1H), 3.72 (s, 3H), 3.69 (s, 1.2H), 3.68 – 3.62 (m, 0.4H), 3.58 – 3.49 (m, 1H), 3.31 (ddd, J = 8.6, 6.3, 2.5 Hz, 0.4H), 3.16 (dddd, J = 6.6, 5.0, 3.8, 1.5 Hz, 1H), 3.03 (ddt, J = 5.2, 2.5, 1.3 Hz, 0.4H), 2.85 (ddq, J = 10.6, 5.3, 2.7 Hz, 0.4H), 2.79 – 2.68 (m, 1.4H), 2.67 – 2.54 (m, 3.4H), 2.47 (ddd, J = 12.6, 8.3, 2.7 Hz, 0.4H), 2.42 – 2.29 (m, 1.4H).

^{13}C NMR (101 MHz, $CDCl_3$) δ 174.17, 158.55, 141.40 (*minor*), 141.38, 110.23 (*minor*), 110.20, 104.20 (*minor*), 104.14, 51.96 (*minor*), 51.63, 44.68 (*minor*), 44.49, 43.72 (*minor*), 39.26, 38.77 (*minor*), 34.20, 34.09, 33.50 (*minor*), 31.23 (*minor*), 30.87 (*minor*), 29.50, 29.25.

FTIR: 2951, 1723, 1512, 1491, 1463, 1435, 1350, 1243, 1200, 1176, 1029 cm^{-1}.

LRMS (ESI): Calculated for $C_{12}H_{15}O_3$ [M+H$^+$]: 207.24, Found: 207.1.

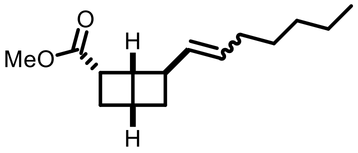

5b

1-(6-(hept-1-en-1-yl)bicyclo[2.2.0]hexan-2-yl)ethan-1-one (5b): Following General Procedure H with **SI-38** (12.00 mmol), an isomeric mixture of products **(5b)** was obtained after removal of byproducts via MPLC (gradient: 0% EtOAc in Hexanes to 0.5% over 1 min., 0.5% to 1% over 1 min., 1% to 1.5% over 1 min., 1.5% to 2% over 1 min., 2% to 5% over 8 min., 5% to 100% over 2.7 min., 6.4 min. hold.) as a faint oil**.**

^{1}H NMR (500 MHz, $CDCl_3$) δ 5.66 – 5.22 (m, 1.81H), 3.69 (d, *J* = 1.7 Hz, 2H), 3.67 – 3.53 (m, 0.64H), 3.53 – 2.78 (m, 3.2H), 2.78 – 2.49 (m, 3H), 2.49 – 2.08 (m, 2.32H), 1.97 (qq, *J* = 13.4, 7.2 Hz, 2.25H), 1.40 – 1.20 (m, 6H), 0.88 (t, *J* = 6.8 Hz, 3H).

Functional Group Manipulations

1. Transformations of Cinnamaldehyde-Derived Products

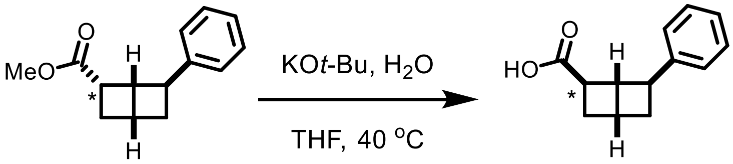

5a 14

methyl 6-phenylbicyclo[2.2.0]hexane-2-carboxylate (14): KO*t*-Bu (1.992 g, 17.8 mmol, 2 equiv.) was added to an oven-dried scintillation vial in a N_2-atmosphere glovebox. The vial was capped and removed from the glovebox. Ester **5a** (1.9196 g, 8.9 mmol, 1 equiv., 4:1 dr) was added to a 200 mL pear-shaped flask with stir bar under N_2. THF (90 mL, 0.1 M) was added to the pear-shaped flask. The septum of the flask was briefly removed to pour the KO*t*-Bu into the reaction mixture. The flask was flushed with N_2 before being sealed with a Teflon cap and Teflon tape. The reaction mixture stirred at 40 °C for approx. 18 hours. The reaction was quenched with sat. aq. NH_4Cl and transferred to a separatory funnel. The aqueous layer was extracted with CH_2Cl_2 three times. The combined organic layers were dried over $MgSO_4$, filtered, and concentrated under reduced pressure. The product was used without further purification to yield a clear, colorless oil (1.5409 g, 78% yield, 6:1 dr).

^{1}H NMR (500 MHz, $CDCl_3$) δ 7.35 – 7.29 (m, 2.3H), 7.27 – 7.17 (m, 3.45H), 3.94 (td, *J* = 8.3, 3.8 Hz, 0.15H), 3.68 (td, *J* = 7.6, 2.6 Hz, 1H), 3.65 – 3.59 (m, 0.15H), 3.37 (ddd, *J* = 8.5, 6.0, 2.3 Hz, 1H), 3.22 – 3.17 (m, 0.15H), 3.12 – 3.07 (m, 1H), 2.90 (dtt, *J* = 8.2, 5.5, 2.7 Hz, 1H), 2.86 – 2.78 (m, 1H), 2.73 (q, *J* = 6.2 Hz, 0.15H), 2.70 – 2.64 (m, 0.3H), 2.64 – 2.42 (m, 3.3H).

^{13}C NMR (126 MHz, $CDCl_3$) δ 181.64, 181.56 (*minor*), 146.13, 145.84 (*minor*), 128.61, 128.47 (*minor*), 126.52, 126.20, 125.94 (*minor*), 46.58 (*minor*), 46.25, 45.77, 44.10, 40.45 (*minor*), 39.58 (*minor*), 36.23, 35.63 (*minor*), 30.95, 30.86, 29.64 (*minor*), 28.77 (*minor*).

FTIR: 3022.14 (w), 2984.46 (w), 2927.37 (m), 1685.88 (s), 1419.03 (m), 1220.87 (m), 923.17 (m), 696.29 (s), 470.42 (m).

HRMS (ESI): Calculated for $C_{13}H_{13}O_2$ [M-H$^+$]: 201.0921, Found: 201.0919.

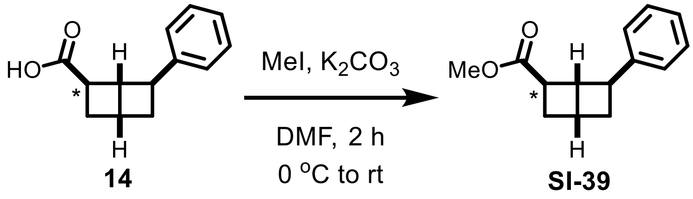

methyl 6-phenylbicyclo[2.2.0]hexane-2-carboxylate (SI-39): Carboxylic acid **14** (0.6068 g, 1.000 equiv., 3.000 mmol, 6:1 dr) was added to a flame-dried 50 mL round-bottomed flask under N_2. DMF (6 mL, 0.5 M) was added, and the solution was cooled to 0 °C in an ice water bath. The septum was removed and K_2CO_3 (0.6219 g, 1.500 equiv., 4.500 mmol,) was added in one portion. The septum was quickly replaced, and the reaction was flushed with N_2. The reaction mixture stirred at 0 °C for 15 minutes before MeI (0.56 mL, 3.0 equiv., 9.0 mmol) was added via syringe. After stirring for 2 hours at room temperature, the reaction was quenched with DI H_2O. The reaction mixture was transferred to a separatory funnel and extracted with EtOAc three times. The combined organic layers were dried over $MgSO_4$, filtered, and concentrated under reduced pressure. Purification by MPLC (gradient: 0 to 5% EtOAc in Hexanes over 9 min., 3 min. hold, 5 to 100% EtOAc over 2 min., 6 min. hold) yielded a clear, colorless liquid (0.5454 g, 84% yield, 6:1 dr).

^{1}H NMR (500 MHz, Acetone-*d$_6$*) δ 7.36 – 7.14 (m, 5.75H), 3.78 (td, *J* = 8.1, 3.8 Hz, 0.15H), 3.73 – 3.66 (m, 1.45H), 3.64 (s, 3H), 3.63 – 3.57 (m, 0.15H), 3.37 (ddd, *J* = 8.5, 6.1, 2.5 Hz, 1H), 3.08 (ddq, *J* = 9.4, 5.4, 2.0 Hz, 0.15H), 2.95 (ddq, *J* = 5.3, 2.5, 1.4 Hz, 1H), 2.85 (tdt, *J* = 8.1, 5.4, 2.7 Hz, 1H), 2.74 (m, 1H), 2.59 (m, 1.30H), 2.58 – 2.35 (m, 2.45H).

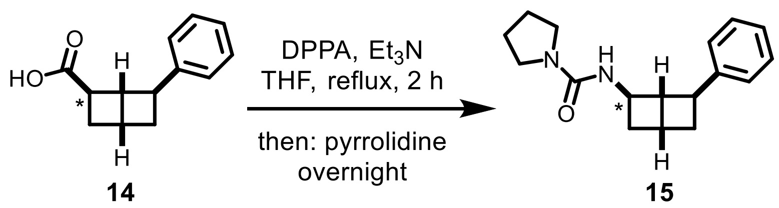

***N*-(6-phenylbicyclo[2.2.0]hexan-2-yl)pyrrolidine-1-carboxamide (15):** Procedure was adapted from literature.[9] Carboxylic acid **14** (101 mg, 1 equiv., 0.500 mmol, 6:1 dr) was added to a flame-dried 2 dram vial with stir bar. The vial was evacuated and refilled with N_2 three times. THF (3 mL, 0.15 molar) was added under N_2 before diphenylphosphoryl azide (0.16 mL, 1.5 equiv., 0.75 mmol) was added followed by Et_3N (0.10 mL, 1.4 equiv., 0.70 mmol). After the reaction was refluxed for 2 hours, the solution was allowed to cool to room temperature and pyrrolidine (0.12 mL, 3.0 equiv., 1.5 mmol) was added. The resulting solution was refluxed for approx. 18 hours. The mixture was cooled to room temperature and concentrated under reduced pressure. Purification via MPLC (0% EtOAc for 0.5 min., 0% to 0.2% EtOAc over 0.5 min., 0.2 to 0.5% EtOAc over 0.5 min., 0.5% to 1% EtOAc over 0.5 min., 1% EtOAc to 2% EtOAc over 0.5 min., 2% EtOAc to 3% over 0.5 min., 3% EtOAc to 5% over 0.5 min., % EtOAc for 5 min., 5% to 10% over 0.5 min.) yielded a white solid (0.0620 g, 46% yield, 6:1 dr).

^{1}H NMR (500 MHz, $CDCl_3$) δ 7.34 – 7.13 (m, 5.75H), 4.62 (q, J = 7.4 Hz, 0.3H), 4.56 – 4.48 (m, 1H), 4.39 (d, J = 6.7 Hz, 1H), 3.87 (td, J = 8.4, 4.3 Hz, 0.15H), 3.63 (ddd, J = 8.3, 6.2, 2.4 Hz, 1H), 3.33 (m, J = 5.6 Hz, 4.6H), 2.94 – 2.86 (m, 0.15H), 2.82 (tdt, J = 8.2, 5.5, 2.8 Hz, 1H), 2.74 – 2.44 (m, 4.3H), 2.35 (dd, J = 11.7, 8.0 Hz, 0.15H), 2.23 (ddd, J = 13.4, 7.4, 5.4 Hz, 1H), 2.11 – 2.05 (m, 0.15H), 1.89 (h, J = 3.7 Hz, 4.6H).

^{13}C NMR (126 MHz, $CDCl_3$) δ 156.16, 146.69, 128.50, 128.48 (*minor*), 126.62 (*minor*), 126.59, 125.87, 125.81 (*minor*), 52.82, 52.27, 49.83 (*minor*), 45.65, 44.38 (*minor*), 43.79, 37.59, 36.82 (*minor*), 36.53 (*minor*), 36.28 (*minor*), 35.38, 28.99, 25.74.

FTIR: 3273.74 (w), 2969.29 (w), 2872.44 (w), 1615.58 (s), 1515.10 (s), 1405.03 (m), 1322.14 (m), 1201.64 (m), 746.39 (m), 696.92 (s), 602.61 (m), 579.14 (m).

HRMS (ESI): Calculated for $C_{17}H_{22}ON_2Na$ [M+Na$^+$]: 293.1624, Found: 293.1625.

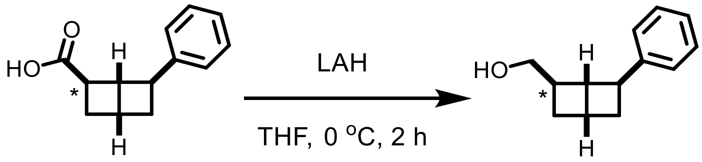

14 **16**

(6-phenylbicyclo[2.2.0]hexan-2-yl)methanol (16): LAH (0.7506 g, 4.000 equiv., 19.78 mmol) was added to a flame-dried scintillation vial with stir bar. The vial was evacuated and backfilled with N_2 three times. THF (4 mL) was added slowly to the vial, and the vial was cooled to 0 °C. Carboxylic acid **14** (1.000 g, 1 equiv., 4.944 mmol, 6:1 dr) was added to a separate flame-dried 1-dram vial. The vial was evacuated and backfilled with N_2 three times. THF (1 mL) was used to transfer carboxylic acid **14** to the vial containing LAH dropwise. The resulting mixture stirred at 0 °C in an ice bath for 2 h. The reaction was quenched with DI H_2O at 0 °C. The layers were separated, and the aqueous layer was extracted twice more with EtOAc. The combined organic layers were dried over $MgSO_4$, filtered, and concentrated. Purification via MPLC yielded a colorless oil (0.8916 g, 96% yield, 6:1 dr).

^{1}H NMR (500 MHz, $CDCl_3$) δ 7.36 – 7.15 (m, 5.75H), 3.99 – 3.90 (m, 0.3H), 3.81 (dd, *J* = 10.8, 6.5 Hz, 0.15H), 3.65 (ddt, *J* = 17.9, 7.6, 3.3 Hz, 3H), 2.97 (dddt, *J* = 7.8, 5.8, 4.2, 2.0 Hz, 0.15H), 2.95 – 2.87 (m, 0.15H), 2.84 – 2.76 (m, 1H), 2.74 – 2.68 (m, 0.15H), 2.64 (t, *J* = 5.5 Hz, 2H), 2.59 – 2.48 (m, 2.3H), 2.36 (ddd, *J* = 12.1, 8.1, 1.6 Hz, 0.15H), 2.30 (ddd, *J* = 12.4, 7.5, 2.9 Hz, 1H), 2.13 (ddd, *J* = 13.0, 7.0, 4.7 Hz, 1H), 1.93 (ddd, *J* = 12.5, 8.5, 4.1 Hz, 0.15H), 1.59 (s, 0.15H), 1.37 (s, 1H).

^{13}C NMR (126 MHz, $CDCl_3$) δ 147.15, 146.90 (*minor*), 128.52, 128.50 (*minor*), 126.61 (*minor*), 126.59, 125.93, 125.81 (*minor*), 67.03, 63.29 (*minor*), 45.90, 45.59 (*minor*), 45.34, 43.07, 37.36 (*minor*), 37.25 (*minor*), 36.78 (*minor*), 36.69, 30.48, 30.39, 28.74 (*minor*).

FTIR: 3353.28 (br), 2923.55 (s), 2853.09 (m), 1454.73 (m), 1031.05 (m), 746.41 (m), 698.95 (s).

HRMS (EI): Calculated for $C_{13}H_{14}$ [M-H_2O^+]: 170.1096, Found: 170.1090.

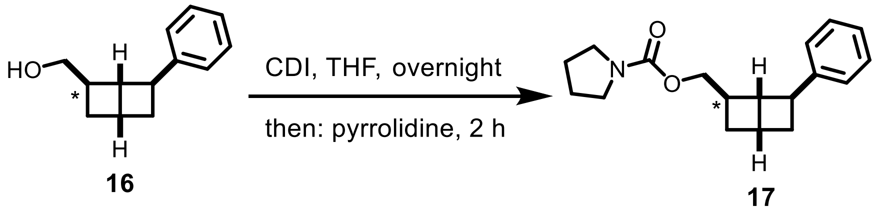

(6-phenylbicyclo[2.2.0]hexan-2-yl)methyl pyrrolidine-1-carboxylate (17): Procedure was adapted from literature.[10] CDI (2.264 g, 3.000 equiv., 13.96 mmol) was added to a flame-dried 100 mL pear-shaped flask with stir bar. The flask was evacuated and refilled with N_2 three times. THF (40 mL) was added followed by alcohol **16** (0.8762 g, 1 equiv., 4.654 mmol, 6:1 dr) at 0 °C. After approx. 18 hours, pyrrolidine (2.3 mL, 6.0 equiv., 28 mmol) was added and stirred for 5 hours. The reaction was concentrated under reduced pressure. Purification via MPLC (0% EtOAc in Hexanes for 0.5 min., 0% to 100% EtOAc over 9 min., 100% EtOAc for 6.2 min.) yielded a clear, colorless oil (1.2183 g, 92% yield, 6:1 dr).

^{1}H NMR (500 MHz, $CDCl_3$) δ 7.34 – 7.13 (m, 6H), 4.38 – 4.29 (m, 0.16H), 4.18 – 4.08 (m, 2H), 3.96 (td, J = 8.0, 3.9 Hz, 0.16H), 3.65 (td, J = 7.6, 2.8 Hz, 1H), 3.44 – 3.19 (m, 4.6H), 3.08 – 2.98 (m, 0.16H), 2.95 – 2.90 (m, 0.16H), 2.82 – 2.72 (m, 2H), 2.69 (dt, J = 6.4, 2.2 Hz, 1.16H), 2.62 – 2.46 (m, 2.32H), 2.40 – 2.27 (m, 1.16H), 2.24 – 2.15 (m, 1H), 1.99 (ddd, J = 12.6, 8.6, 4.2 Hz, 0.16H), 1.84 (tt, J = 10.3, 5.2 Hz, 4.6H).

^{13}C NMR (126 MHz, $CDCl_3$) δ 155.52, 155.36 (*minor*), 147.09, 146.99 (*minor*), 128.50, 128.36 (*minor*), 126.61 (*minor*), 126.54, 125.87, 125.70 (*minor*), 68.36, 65.01 (*minor*), 46.25 (*minor*), 45.94, 45.83 (*minor*), 45.22, 40.09, 37.54 (*minor*), 36.49 (*minor)*, 36.46, 34.21 (*minor*), 30.48 (*minor*), 30.44, 30.36, 28.89 (*minor*), 25.86, 25.10.

FTIR: 2949.00 (m), 1695.85 (s), 1416.03 (s), 1363.61 (m), 1097.74 (s), 745.30 (m), 697.99 (s).

HRMS (ESI): Calculated for $C_{18}H_{23}O_2NNa$ [M+Na$^+$]: 308.1621, Found: 308.1622.

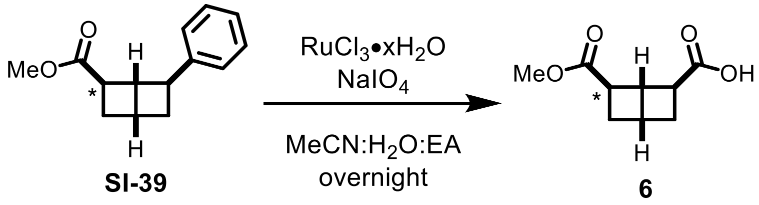

6-(methoxycarbonyl)bicyclo[2.2.0]hexane-2-carboxylic acid (6): Procedure was adapted from literature.[11] Ester **SI-39** (0.5407 g, 2.5 mmol, 1 equiv., 6:1 dr) was added to a 50 mL round-bottomed flask with stir bar. EtOAc (5 mL, 0.5 M), MeCN (5 mL, 0.5 M), and DI H_2O (7.6 mL, 0.33 M) were added to the flask in that order. Next, $NaIO_4$ (7.75 g, 36.3 mmol, 14.5 equiv.) was added to the reaction mixture, and the resulting heterogenous mixture was vigorously stirred while $RuCl_3 \cdot xH_2O$ (0.0114 g, 0.055 mmol, 2.2 mol%) was added. The flask was fitted with a reflux condenser open to air. After approx. 15 min., the reaction mixture starts refluxing and turns to a solid mass. After stirring for approx. 18 hours at room temperature, the reaction was diluted with CH_2Cl_2 and quenched with 1 M aq. HCl. The layers were separated, and the aqueous layer was extracted four more times with CH_2Cl_2. The combined organic layers were dried over $MgSO_4$, filtered, and concentrated under reduced pressure to yield a viscous oil (0.2756 g, 60% yield, 5:1 dr).

^{1}H NMR (500 MHz, Acetone-d$_6$) δ 3.65 (d, J = 2.5 Hz, 3.6H), 3.40 – 3.28 (m, 2.2H), 3.27 – 3.20 (m, 0.2H), 3.09 (dtt, J = 5.0, 2.5, 1.2 Hz, 1H), 2.78 – 2.52 (m, 3.8H), 2.45 (td, J = 8.5, 4.1 Hz, 0.2H), 2.35 (ddt, J = 12.5, 8.5, 2.1 Hz, 2H), 2.17 (ddd, J = 12.0, 8.4, 1.3 Hz, 0.2H).

^{13}C NMR (126 MHz, Acetone-d$_6$) δ 176.04 (*minor*), 176.00, 175.58, 173.91 (*minor*), 51.92, 51.58 (*minor*), 43.82, 43.76, 42.94, 42.42 (*minor*), 39.33 (*minor*), 39.21 (*minor*), 32.34 (*minor*), 32.12, 31.30, 31.26, 30.34 (*minor*), 29.93 (*minor*).

FTIR: 3088.75 (br), 2940.27 (m), 2856.44 (w), 1727.53 (s), 1697.90 (s), 1435.29 (m), 1352.60 (m), 1254.06 (m), 1159.16 (s), 1059.14 (m), 930.62 (w), 791.04 (w), 675.13 (w).

HRMS (APCI): Calculated for $C_9H_{13}O_4$ [M+H$^+$]: 185.0808, Found: 185.0808.

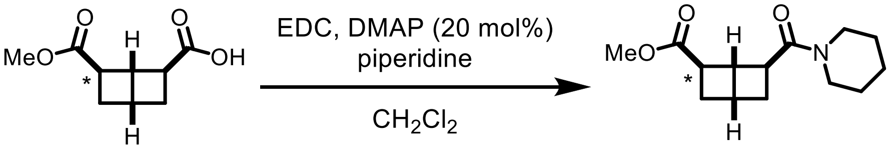

6 **SI-40**

methyl 6-(piperidine-1-carbonyl)bicyclo[2.2.0]hexane-2-carboxylate (SI-40): EDC (0.8972 g, 1.200 equiv., 5.779 mmol) and DMAP (0.1177 g, 0.2000 equiv., 0.9631 mmol) were added to a flame-dried 100 mL round bottom flask with stir bar. The flask was evacuated and refilled with N_2 three times. Carboxylic acid **6** (.8870 g, 1.000 equiv., 4.816 mmol, 5:1 dr) was transferred to the flask using CH_2Cl_2 (50 mL, 0.10 molar), and the resulting solution was cooled to 0 °C in an ice bath. Piperidine (0.60 mL, 1.2 equiv., 5.8 mmol) was added to the solution under N_2, and the reaction was brought to room temperature over approx. 18 hours. The reaction mixture was concentrated and purified by MPLC to yield a clear, colorless oil (0.6991 g, 58% yield, 6:1 dr).

^{1}H NMR (500 MHz, CDCl$_3$) δ 3.69 (d, J = 6.8 Hz, 3.45H), 3.65 – 3.52 (m, 1.3H), 3.48 (ddd, J = 12.5, 6.9, 4.5 Hz, 1.15H), 3.32 (td, J = 7.8, 2.8 Hz, 1.15H), 3.20 (td, J = 11.9, 6.6 Hz, 3.3H), 3.12 – 3.07 (m, 1H), 2.86 (dt, J = 13.3, 7.5 Hz, 1H), 2.80 – 2.71 (m, 2.15H), 2.68 (s, 0.15H), 2.57 (q, J = 10.7 Hz, 0.15H), 2.49 (ddd, J = 12.7, 8.2, 3.9 Hz, 0.15H), 2.32 (dd, J = 10.4, 8.5 Hz, 1H), 2.24 (ddd, J = 12.0, 8.7, 2.0 Hz, 1H), 2.20 – 2.14 (m, 0.15H), 1.69 – 1.45 (m, 7H).

^{13}C NMR (126 MHz, CDCl$_3$) δ 175.47, 174.18 (*minor*), 172.39 (*minor*), 171.88, 52.01, 51.57 (*minor*), 46.14, 45.95 (*minor*), 43.70, 43.18 (*minor*), 43.07, 43.00, 42.41, 41.16 (*minor*), 38.78 (*minor*), 37.32 (*minor*), 31.99 (*minor*), 30.65, 30.42, 30.34, 29.66 (*minor*), 29.48 (*minor*), 26.60 (*minor*), 26.47, 25.70, 24.76.

FTIR: 2933.22 (m), 2853.25 (m), 1728.01 (s), 1633.22 (s), 1432.16 (s), 1349.22 (m), 1252.68 (s), 1217.10 (s), 1016.14 (m), 852.20 (m).

HRMS (ESI): Calculated for $C_{14}H_{21}O_3NNa$ [M+Na$^+$]: 274.1414, Found: 274.1415.

2. Transformations of (*E,E*)-2,4-decadienal-Derived Products

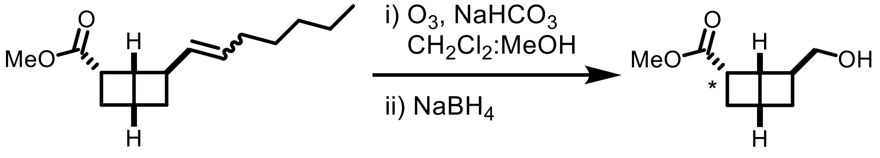

methyl 6-(hydroxymethyl)bicyclo[2.2.0]hexane-2-carboxylate (SI-41): Procedure was adapted from literature.[12] Crude ester **5b** (10.00 mmol, isomeric mixture), methanol (200 mL, 0.05 molar), CH_2Cl_2 (200.0 mL, 0.05 molar), and $NaHCO_3$ (0.0840 g, 0.100 equiv., 1.00 mmol) were added to a flame-dried 1000 mL round-bottomed flask with stir bar. The resulting mixture was sparged with Ozone at -78 °C until a blue color persisted (approx. 30 min.). Next, the reaction mixture was sparged with N_2 while warming to room temperature. Once the blue color disappeared, $NaBH_4$ (2.2700 g, 6.000 equiv., 60.00 mmol) was added to the reaction. This resulting mixture was stirred at room temperature under air for 2 hours. The reaction was quenched with DI H_2O, and the layers were separated. The aqueous layer was extracted with CH_2Cl_2 twice more. The combined organic layers were dried over $MgSO_4$, filtered, and concentrated under reduced pressure. Purification via MPLC (50% EtOAc in Hexanes to 70% EtOAc over 1 min., 70% to 100% EtOAc over 8.4 min., 100% EtOAc for 2 min.) yielded a clear, colorless oil (0.4822 g, 28% yield over 5 steps, 3:1 dr).

^{1}H NMR (500 MHz, $CDCl_3$) δ 3.73 – 3.67 (m, 4H), 3.64 (dt, J = 12.6, 4.5 Hz, 1.66H), 3.57 (dd, J = 10.7, 7.9 Hz, 1H), 3.49 (dt, J = 10.3, 8.1 Hz, 1H), 3.27 – 3.21 (m, 0.33H), 2.83 (pd, J = 4.3, 2.6 Hz, 1H), 2.77 – 2.54 (m, 4.32H), 2.54 – 2.44 (m, 1H), 2.28 (t, J = 9.3 Hz, 0.33H), 2.22 – 2.15 (m, 0.33H), 2.12 – 1.97 (m, 2.33H), 1.43 (s, 1.33H).

^{13}C NMR (126 MHz, $CDCl_3$) δ 176.18 (*minor*), 174.60, 66.93, 66.69 (*minor*), 51.92 (*minor*), 51.65, 43.18 (*minor*), 42.52 (*minor*), 41.51 (*minor*), 41.20, 39.07, 38.40, 31.26 (*minor*), 31.23 (*minor*), 30.93, 30.18 (*minor*), 30.12, 29.40.

FTIR: 3399.81 (br), 2948.68 (m), 2854.62 (m), 1727.83 (s), 1435.22 (m), 1344.24 (m), 1197.39 (s), 1174.32 (s), 1047.80 (s), 967.65 (m), 762.38 (w), 478.93 (w).

HRMS (ESI): Calculated for $C_9H_{14}O_3Na$ [M+Na$^+$]: 193.0835, Found: 193.0835.

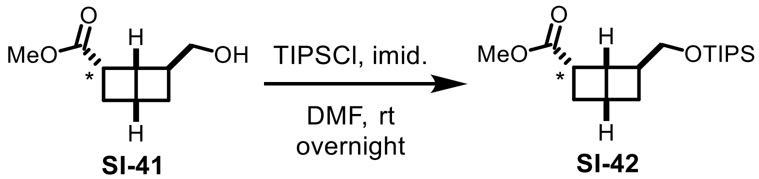

methyl 6-(((triisopropylsilyl)oxy)methyl)bicyclo[2.2.0]hexane-2-carboxylate (SI-42): Alcohol **SI-41** (0.3667 g, 1.000 equiv., 2.154 mmol, 3:1 dr) was added to a flame-dried 1-dram vial with stir bar. The vial was evacuated and refilled with N_2 three times. DMF (1.0 mL, 2.0 molar) was added to the vial under N_2. The septum was removed and imidazole (0.1540 g, 1.050 equiv., 2.262 mmol) was added to the solution. The septum was quickly replaced, and the reaction mixture was flushed with N_2 for 5 min. before TIPS-Cl (0.50 mL, 1.1 equiv., 2.3 mmol) was added. The reaction mixture stirred at room temperature for approx.

18 hours. The reaction was quenched with DI H_2O and extracted with Et_2O. The layers were separated, and the aqueous layer extracted with Et_2O once more. The combined organic layers were dried over $MgSO_4$, filtered, and concentrated under reduced pressure. Purification via MPLC (0% to 10% EtOAc in Hexanes over 9 min., 10% to 100% EtOAc over 3 min., 100% EtOAc for 3 min.) yielded a clear, colorless oil (0.5720 g, 81% yield, 3:1 dr).

^{1}H NMR (500 MHz, $CDCl_3$) δ 3.66 (h, J = 6.7 Hz, 6.5H), 3.52 – 3.43 (m, 1H), 3.25 – 3.19 (m, 0.33H), 2.88 (dq, J = 8.7, 2.7 Hz, 1H), 2.76 – 2.44 (m, 5.33H), 2.24 (dd, J = 10.7, 8.4 Hz, 0.33H), 2.19 – 2.08 (m, 1.66H), 1.97 (ddd, J = 11.9, 7.8, 1.9 Hz, 1H), 1.05 (q, J = 4.9 Hz, 28H).

^{13}C NMR (126 MHz, $CDCl_3$) δ 176.43 (*minor*), 174.66, 67.06, 66.85 (*minor*), 51.81 (*minor*), 51.45, 43.29 (*minor*), 42.97 (*minor*), 41.97 (*minor*), 41.13, 39.17, 38.35, 31.23 (*minor*), 31.08 (*minor*), 30.90, 30.10 (*minor*), 29.69, 29.62, 18.16, 12.20 (*minor*), 12.16.

FTIR: 2941.70 (s), 2864.66 (s), 1734.57 (s), 1461.98 (m), 1434.63 (m), 1381.74 (m), 1195.99 (s), 1175.54 (s), 1096.49 (s), 1066.99 (s), 1013.23 (m), 881.23 (s), 679.65 (s), 657.34 (s).

HRMS (ESI): Calculated for $C_{18}H_{34}O_3NaSi$ [M+Na$^+$]: 349.2169, Found: 349.2170.

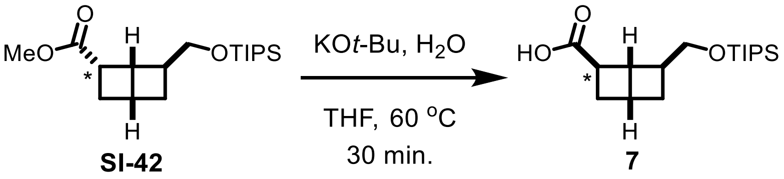

6-(((triisopropylsilyl)oxy)methyl)bicyclo[2.2.0]hexane-2-carboxylic acid (7): KO*t*-Bu (0.6872 g, 2.000 equiv., 6.125 mmol) was added to a flame-dried scintillation vial in a N_2-atmosphere glovebox. The vial was called and removed from the glovebox. Ester **SI-42** (1.000 g, 1.000 equiv., 3.062 mmol, 3:1 dr) was added to a 100 mL round bottom flask. The flask was evacuated and refilled with N_2 three times and capped with a septum. THF (30 mL, 0.1 molar) was added to the flask under N_2. The septum was removed, and the KO*t*-Bu was added in one portion. The septum was quickly replaced, and the flask was flushed with N_2 for 5 min. while DI H_2O (55 µL, 1.0 equiv., 3.1 mmol) was added. The reaction stirred N_2 at 60 °C for 30 min. The reaction mixture was cooled to room temperature and was quenched with 1M HCl. The layers were separated, and the aqueous layer was extracted twice more with EtOAc. The combined organic layers were dried over $MgSO_4$, filtered, and concentrated under reduced pressure. Purification via MPLC (0-15% EtOAc in Hexanes over 28.1 min.,15% EtOAc to 16.6% EtOAc over 0.8 min., 16.6% EtOAc to 100% EtOAc over 7.7 min., 100% EtOAc over 8.5 min.) yielded a clear, colorless oil (0.8139 g, 85% yield, 10:1 dr).

^{1}H NMR (500 MHz, $CDCl_3$) δ 3.75 – 3.64 (m, 2.2H), 3.58 – 3.51 (m, 0.1H), 3.25 (ddd, J = 8.8, 6.0, 2.5 Hz, 1H), 2.92 (d, J = 6.9 Hz, 0.1H), 2.83 – 2.67 (m, 3.2H), 2.56 (h, J = 7.8 Hz, 1.2H), 2.50 – 2.44 (m, 0.1H), 2.28 (dd, J = 11.1, 8.5 Hz, 1H), 2.24 – 2.08 (m, 2H), 1.97 (dd, J = 12.1, 7.9 Hz, 0.1H), 1.05 (d, J = 5.8 Hz, 23H).

^{13}C NMR (126 MHz, $CDCl_3$) δ 181.62, 66.91 (*minor*), 66.78, 43.21, 42.96, 41.84, 41.06 (*minor*), 39.06 (*minor*), 38.34 (*minor*), 31.26, 31.09, 30.82 (*minor*), 30.12, 29.62 (*minor*), 29.44 (*minor*), 29.39 (*minor*), 18.18, 17.84 (*minor*), 12.42 (minor), 12.19.

FTIR: 2940.66 (s), 2864.56 (s), 1700.77 (s), 1462.00 (m), 1248.42 (m), 1232.81 (m), 1098.19 (s), 881.05 (s), 679.65 (s), 658.08 (s).

HRMS (ESI): Calculated for $C_{17}H_{32}O_3NaSi$ [M+Na$^+$]: 335.2013, Found: 335.2015.

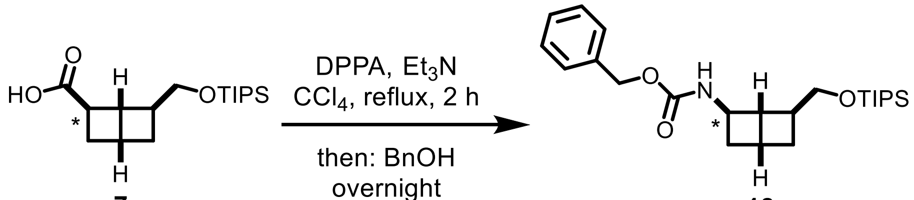

benzyl (6-(((triisopropylsilyl)oxy)methyl)bicyclo[2.2.0]hexan-2-yl)carbamate (18): Procedure was adapted from literature.[9] Carboxylic acid **7** (0.0151 mg, 1.00 equiv., 0.0483 mmol, 10:1 dr) was added to a flame-dried 1-dram vial. CCl_4 (0.3 mL, .20 molar) was added followed by DPPA (11 µL, 1.0 equiv., 0.048 mmol) and Et_3N (7 µL, 1.05 equiv., 0.051 mmol). The resulting solution was refluxed for 2 h under N_2. Benzyl alcohol (10 µL, 2 equiv., 0.0966 mmol) was added in one portion, and the reaction continued stirring at 80 °C for approx. 18 hours. After cooling to room temperature, EtOAc and DI H_2O were added to the reaction mixture, and layers were separated. The organic layer was washed with brine, dried over Na_2SO_4, and concentrated under reduced pressure. Purification via MPLC (100% Hexanes for 0.7 min., increasing to 100% EtOAc over 13.3 min., 100% EtOAC for 8.2 min.) yielded a yellowish oil (0.0142 g, 70% yield, 10:1 dr).

^{1}H NMR (500 MHz, $CDCl_3$, 40 °C) δ 7.40 – 7.30 (m, 5.5H), 5.12 (s, 2.3H), 4.94 (s, 1H), 4.29 (s, 1H), 3.78 – 3.64 (m, 2.3H), 3.28 (s, 0.1H), 2.92 – 2.70 (m, 0.4H), 2.65 (dtq, J = 8.1, 5.4, 2.7 Hz, 1H), 2.62 – 2.45 (m, 3H), 2.34 – 2.26 (m, 0.1H), 2.19 (dp, J = 12.9, 6.8 Hz, 2.1H), 2.11 – 2.04 (m, 1H), 1.08 (d, J = 4.7 Hz, 23H).

^{13}C NMR (126 MHz, $CDCl_3$) δ 155.38, 136.76, 128.66, 128.30, 128.24, 66.66, 52.31, 46.93, 42.97, 40.87, 37.46, 29.72, 29.18, 18.20, 12.19.

FTIR: 3324.99 (br), 2940.65 (s), 2864.24 (s), 1707.41 (s), 1522.76 (m), 1462.04 (m), 1248.34 (s), 1096.70 (s), 881.99 (s), 775.20 (m), 681.29 (s).

HRMS (ESI): Calculated for $C_{24}H_{39}O_3NNaSi$ [M+Na$^+$]: 440.2591, Found: 440.2592.

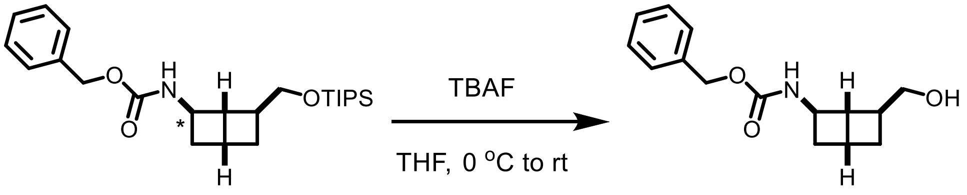

benzyl (6-(hydroxymethyl)bicyclo[2.2.0]hexan-2-yl)carbamate (SI-43): Carbamate **18** (0.0393 g, 1.00 equiv., 0.0941 mmol, 10:1 dr) and THF (0.2 mL, 0.5 molar) were added to a flame-dried scintillation vial equipped with a stir bar. The resulting solution was cooled to 0 °C in an ice bath and TBAF (0.2 mL, 1 molar in THF, 2 equiv., 0.2 mmol) was added. The reaction mixture was allowed to warm to room temperature over 18 hours. The reaction was quenched with sat. aq. NH_4Cl and extracted with EtOAc. The layers were separated, and the aqueous layer was extracted twice more with EtOAc. The combined organic layers were dried over $MgSO_4$, filtered, and concentrated under reduced pressure. Purification via MPLC (0%

EtOAc in Hexanes for 0.5 min., 0% to 100% EtOAc over 6 min., 100% EtOAc over 4.6 min.) yielded a yellowish oil (0.0209 g, 85% yield, >20:1 dr).

^{1}H NMR (500 MHz, $CDCl_3$) δ 7.40 – 7.30 (m, 5H), 5.09 (s, 2H), 4.97 (s, 1H), 4.29 (s, 1H), 3.65 (d, J = 9.6 Hz, 2H), 2.72 – 2.63 (m, 1H), 2.63 – 2.34 (m, 3H), 2.26 – 2.00 (m, 3H), 1.39 (s, 1H).

^{13}C NMR (126 MHz, $CDCl_3$) δ 155.44, 136.65, 128.68, 128.53, 128.28, 66.78, 66.58, 52.23, 46.82, 40.60, 37.10, 29.73, 29.12.

FTIR: 3317.20 (br), 3033.39 (w), 2925.41 (w), 2853.54 (w), 1693.66 (s), 1530.68 (m), 1260.97 (s), 1039.69 (m), 696.97 (m).

HRMS (ESI): Calculated for $C_{15}H_{19}O_3NNa$ [M+Na$^+$]: 284.1257, Found: 284.1258.

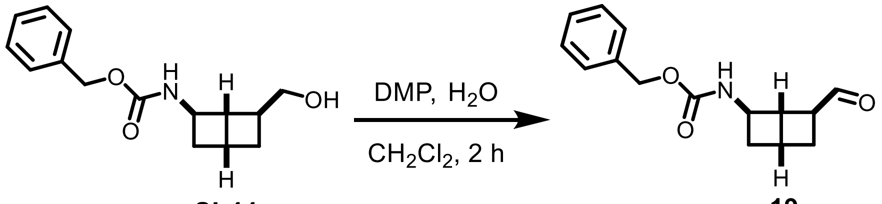

SI-44 → **19**

benzyl (6-formylbicyclo[2.2.0]hexan-2-yl)carbamate (19): Alcohol **SI-44** (0.0143 g, 1.00 equiv., 0.0547 mmol, >20:1 dr) was added to a 1-dram vial with stir bar. The vial was evacuated and refilled with N_2 three times. CH_2Cl_2 (0.5 mL, 0.1 molar) was added to the vial under N_2. The septum was removed and DMP (27.9 mg, 1.20 Eq, 0.0657 mmol) was added to the solution. The septum was quickly replaced, and the vial was flushed with N_2 for 30 seconds DI H_2O (2 µL, 1.2 equiv., 0.0657 mmol). The reaction mixture stirred at room temperature for 3 hours. The reaction was quenched with a 1:1 soln of sat. aq. $NaHCO_3$ and $Na_2S_2O_3$. Once gas formation ceased, the layers were separated, and the aqueous layer extracted once more with CH_2Cl_2. The combined organic layers were dried over $MgSO_4$, filtered, and concentrated under reduced pressure. Purification via MPLC (gradient: 0% EtOAc in Hexanes for 0.9 min., 0% to 100% EtOAc over 9.9 min., 2.5 min. hold) yielded a clear, colorless oil (0.0119 g, 84% yield, >20:1).

^{1}H NMR (500 MHz, $CDCl_3$, 40 °C) δ 9.75 (s, 1H), 7.34 (q, J = 6.4 Hz, 5H), 5.09 (s, 2H), 5.01 (s, 1H), 4.41 (s, 1H), 3.32 (s, 1H), 2.88 (s, 1H), 2.77 – 2.64 (m, 2H), 2.58 (t, J = 10.9 Hz, 1H), 2.29 – 2.21 (m, 1H), 2.16 (t, J = 10.7 Hz, 1H).

^{13}C NMR (126 MHz, $CDCl_3$) δ 200.27, 155.38, 136.49, 128.72, 128.37, 128.32, 66.96, 52.58, 49.40, 44.53, 36.87, 29.29, 26.79.

FTIR: 3324.93 (br), 3033.36 (w), 2930.08 (m), 1698.49 (s), 1525.46 (m), 1342.97 (m), 1254.48 (s), 1072.06 (m), 1015.45 (m), 738.89 (w), 697.61 (m).

HRMS (APCI): Calculated for $C_{15}H_{18}O_3N$ [M+H$^+$]: 260.1281, Found: 260.1281.

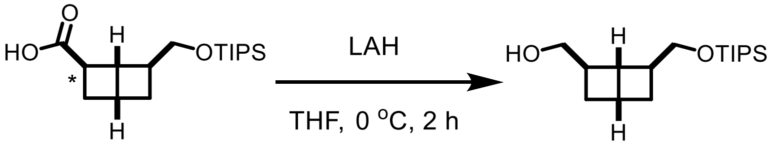

7 **20**

(6-(((triisopropylsilyl)oxy)methyl)bicyclo[2.2.0]hexan-2-yl)methanol (20): LAH (0.0051 g, 4.0 equiv., 0.13 mmol) was added to a flame-dried 1-dram vial with stir bar. The vial was evacuated and backfilled with N_2 three times. THF (0.3 mL, 0.1 molar) was added slowly to the vial, and the resulting mixture was cooled to 0 °C in an ice bath. Carboxylic acid **7** (0.0105 g, 1.00 equiv., 0.336 mmol, 10:1 dr) was added to a separate flame-dried 0.5-dram vial. The vial containing carboxylic acid **7** was evacuated and backfilled with N_2 three times. Another portion of THF (0.3 mL, 0.1 molar) was used to transfer carboxylic acid **7** to the vial containing LAH dropwise. This resulting mixture was stirred at 0 °C for 2 h. The reaction was quenched with DI H_2O at 0 °C. After gas formation ceased, the aqueous layer was extracted with EtOAc. The organic layer was separated, and the aqueous layer was extracted twice more with EtOAc. The combined organic layers were dried over $MgSO_4$, filtered, and concentrated under reduced pressure. Purification via MPLC (30% EtOAc in Hexanes to 100% EtOAc over 7.5 min., 100% EtOAc over 7.7 min.) yielded a clear, colorless oil (0.0086 g, 86% yield, >20:1).

^{1}H NMR (500 MHz, $CDCl_3$) δ 3.74 – 3.57 (m, 4H), 2.61 (tdq, *J* = 7.8, 5.1, 2.4 Hz, 1H), 2.57 – 2.48 (m, 2H), 2.31 (d, *J* = 4.9 Hz, 1H), 2.18 – 1.99 (m, 4H), 1.36 – 1.26 (m, 1H), 1.14 – 0.99 (m, 21H).

^{13}C NMR (126 MHz, $CDCl_3$) δ 67.38, 67.36, 42.43, 42.18, 40.92, 30.83, 30.75, 30.64, 18.20, 12.22.

FTIR: 3333.77 (br), 2923.78 (s), 2865.18 (s), 1463.48 (m), 1098.54 (m), 882.38 (m), 681.21 (m).

HRMS (ESI): Calculated for $C_{17}H_{34}O_2NaSi$ [M+Na$^+$]: 321.2220, Found: 321.2220.

3. Decarboxylative Cross Coupling Scope

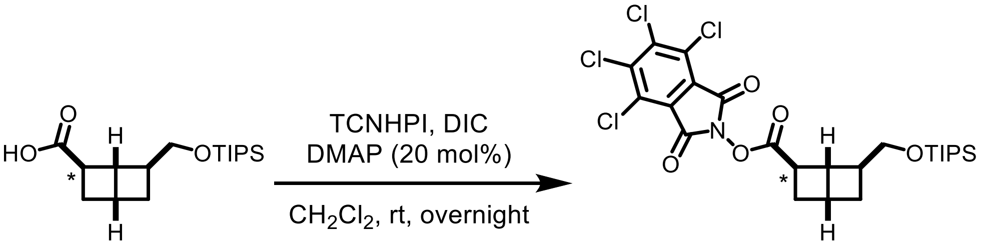

7 **21**

4,5,6,7-tetrachloro-1,3-dioxoisoindolin-2-yl 6-(((triisopropylsilyl)oxy)methyl)bicyclo[2.2.0]hexane-2-carboxylate (21): Procedure was adapted from literature.[4] Carboxylic acid **7** (0.0974 g, 1 equiv., 0.312 mmol, 10:1 dr) was added to a flame-dried 2-dram vial with stir bar. The vial was evacuated and refilled with N_2 three times, capped with a septum, and placed under N_2. CH_2Cl_2 (3 mL, 0.1 molar) was added to the vial containing carboxylic acid **7**. The septum was removed, and TCNHPI (0.0985 g, 1.05 equiv., 0.327 mmol) and DMAP (0.0076 g, 0.20 equiv., 0.062 mmol) were added to the The septum was quickly replaced, and the reaction mixture was flushed with N_2. DIC (52 µL, 1.05 equiv., 0.327 mmol) was added, and the reaction mixture stirred at room temperature for approx. 18 hours. The reaction mixture was filtered through a pad of Celite, and the pad of Celite was washed with CH_2Cl_2. The combined filtrates were

concentrated under reduced pressure. The crude residue was purified via MPLC (0% EtOAc in Hexanes for 0.5 min., 0% to 100% EtOAc over 5.5 min., 100% EtOAc for 7.6 min.) to yield a white solid (0.1242 g, 67% yield, 10:1 dr).

^{1}H NMR (500 MHz, $CDCl_3$) δ 3.80 – 3.71 (m, 2.3H), 3.59 (t, *J* = 7.3 Hz, 1H), 3.14 (m, 0.1H), 3.01 – 2.86 (m, 2.1H), 2.84 – 2.63 (m, 2.2H), 2.58 (t, *J* = 10.0 Hz, 0.1H), 2.50 – 2.44 (m, 1H), 2.44 – 2.36 (m, 0.1H), 2.29 – 2.14 (m, 2H), 2.06 – 1.97 (m, 0.1H), 1.06 (d, *J* = 5.8 Hz, 23.1H).

^{13}C NMR (126 MHz, $CDCl_3$) δ 171.42, 157.85, 141.10, 130.58, 124.95, 66.58, 43.04, 41.83, 40.79 (*minor*), 40.28, 38.09 (*minor*), 36.49 (*minor*), 34.28 (*minor*), 31.64, 31.45, 30.31 (*minor*), 30.14, 29.87 (*minor*), 22.49 (*minor*), 18.19, 14.22 (*minor*), 12.19.

FTIR: 2941.96 (m), 2865.18 (m), 1791.44 (m), 1750.72 (s), 1378.58 (m), 1099.41 (m), 1040.35 (m), 882.14 (m), 732.65 (m).

HRMS (ESI): Calculated for $C_{25}H_{31}Cl_4NO_5SiNa$ [M+Na$^+$]: 616.0618, Found: 616.0618.

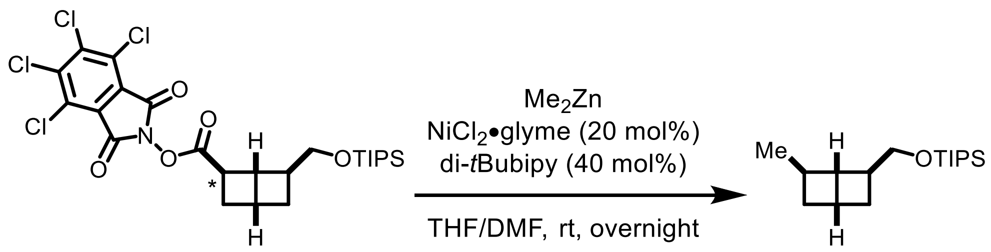

21 **22**

triisopropyl((6-methylbicyclo[2.2.0]hexan-2-yl)methoxy)silane (22): Procedure was adapted from literature.[2] 4,4'-di-*tert*-butyl-2,2'-bipyridine (0.0104 g, 0.400 equiv., 0.0388 mmol) was added to a flame-dried 2-dram vial with stir bar. The vial was evacuated and refilled with N_2 three times. It was capped loosely and brought into a N_2-atmosphere glovebox. In the glovebox, $NiCl_2$·glyme (0.0043 g, 0.20 equiv., 0.019 mmol) was added. The vial was capped with a septum, sealed with Teflon tape, and removed from the glovebox. Redox-active ester **21** (0.0578 g, 1.00 equiv., 0.0971 mmol, 10:1 dr) was added to a separate 1-dram vial. DMF (1.0 mL, 0.10 molar) was used to transfer redox-active ester **21** to the 2-dram vial containing $NiCl_2$·glyme. This light blue mixture stirred for 10 min before freshly-made dimethylzinc (0.6 mL, 0.364 molar in THF, 2 equiv., 0.2 mmol) was added. The reaction mixture turned dark red, to dark green, to navy blue. After approx. 18 hours, the reaction was quenched with 1M HCl and extracted twice with Et_2O. The combined organic layers were washed with DI H_2O followed by brine. The organic layer was dried over $MgSO_4$, filtered, and concentrated under reduced pressure. The crude material was purified via MPLC (0% EtOAc in Hexanes for 3 min., 0% to 5% over 2 min., 5% to 100% EtOAc over 2.5 min., 100% EtOAc for 4 min.) to yield a clear, colorless oil (0.0228 g, 83% yield, >20:1 dr).

^{1}H NMR (500 MHz, $CDCl_3$) δ 3.65 (dq, *J* = 10.1, 8.1 Hz, 2H), 2.57 (tdq, *J* = 7.9, 5.2, 2.6 Hz, 1H), 2.48 – 2.35 (m, 2H), 2.18 (ddt, *J* = 12.1, 7.5, 2.3 Hz, 1H), 2.11 (dd, *J* = 4.9, 2.4 Hz, 1H), 2.09 – 1.98 (m, 2H), 1.86 (dt, *J* = 12.9, 6.8 Hz, 1H), 1.12 – 1.03 (m, 24H).

^{13}C NMR (126 MHz, $CDCl_3$) δ 67.62, 45.93, 42.62, 36.24, 35.00, 30.46, 29.67, 22.75, 18.20, 12.26.

FTIR: 2921.76 (s), 2864.53 (s), 1462.58 (m), 1379.13 (w), 1247.21 (w), 1099.94 (m), 882.35 (m), 680.81 (m).

HRMS (EI): Calculated for $C_{14}H_{27}OSi$ [M-$C_3H_7^+$]: 239.1831, Found: 239.1827.

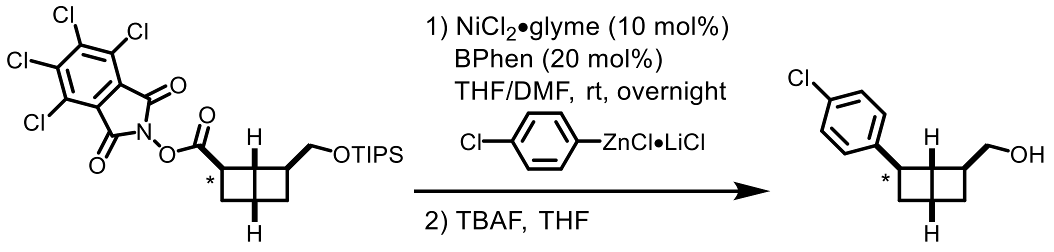

21 **23**

(6-(4-chlorophenyl)bicyclo[2.2.0]hexan-2-yl)methanol (23): Procedure was adapted from literature.[4] BPhen (19.9 mg, 0.200 equiv., 0.0600 mmol) was added to a flame-dried 2-dram vial. The vial was evacuated and refilled with N_2 three times. The vial was capped loosely with a screw cap and brought into a N_2-atmosphere glovebox. $NiCl_2$·glyme (6.6 mg, 0.10 equiv., 0.030 mmol) was added in the glovebox. The vial was capped with a septum, sealed with Teflon tape, and removed from the glovebox. Redox-active ester **21** (0.1790 g, 1.000 equiv., 0.3000 mmol, 10:1 dr) was added to a separate flame-dried 1-dram vial. The vial was evacuated and refilled with N_2 three times. DMF (2.3 mL, 0.13 molar) was used to transfer redox-active ester **21** to the 2-dram vial containing $NiCl_2$·glyme and BPhen. The resulting light blue solution was stirred for 10 min before ArZnCl·LiCl **SI-2** (2.6 mL, 0.23 molar in THF, 2.0 equiv., 0.60 mmol) was added. The reaction quickly turned a dark red, to dark green, to navy blue. The reaction stirred for approx. 18 hours before being quenched with 1M HCl and extracted twice with Et_2O. The combined organic layers were washed with DI H_2O and then brine. It was dried over $MgSO_4$, filtered, and concentrated under reduced pressure.

THF (0.6 mL, 0.5 molar) was added to the flask containing crude material and stir bar. TBAF (0.45 mL, 1.0 molar, 1.5 equiv., 0.45 mmol) was added to the solution, and the reaction mixture stirred at room temperature for approx. 18 hours. The reaction mixture was quenched with sat. aq. NH_4Cl and extracted with EtOAc. The organic layers were separated, and the aqueous layer was extracted twice more with EtOAc. The combined organic layers were dried over $MgSO_4$, filtered, and concentrated under reduced pressure. It was purified via MPLC (0% EtOAc in Hexanes for 0.6 min., 0% to 5% EtOAc over 4.4 min., 5% to 10% over 2 min., 10% to 100% EtOAc over 1.5 min., 12.5 min hold) to yield a white solid (0.0279 g, 42% yield over 2 steps, 8:1 dr).

^{1}H NMR (500 MHz, $CDCl_3$) δ 7.26 (q, *J* = 3.8 Hz, 2.26H), 7.17 (d, *J* = 8.0 Hz, 2H), 7.05 (d, *J* = 7.8 Hz, 0.26H), 3.94 (q, *J* = 9.3 Hz, 0.13H), 3.66 (q, *J* = 9.1 Hz, 2H), 3.60 (t, *J* = 7.6 Hz, 1H), 3.54 (d, *J* = 8.2 Hz, 0.13H), 2.98 – 2.82 (m, 0.26H), 2.78 (ddp, *J* = 7.7, 4.9, 2.9 Hz, 1H), 2.72 (m, 0.13H), 2.67 – 2.58 (m, 2H), 2.54 (ddd, *J* = 12.3, 8.2, 2.3 Hz, 1H), 2.46 (dt, *J* = 13.5, 7.4 Hz, 1H), 2.38 – 2.32 (m, 0.13H), 2.28 (ddd, *J* = 12.6, 7.6, 2.8 Hz, 1H), 2.12 (dt, *J* = 13.0, 6.5 Hz, 1H), 2.04 (dt, *J* = 13.8, 7.4 Hz, 0.13H), 1.87 (dd, *J* = 12.2, 8.0 Hz, 0.13H), 1.37 – 1.23 (m, 1.13H).

^{13}C NMR (126 MHz, $CDCl_3$) δ 145.60, 141.86 (*minor*), 131.50 (*minor*), 128.57, 128.40, 127.98, 67.30 (*minor*), 66.96, 45.88, 44.75, 43.01, 42.86 (*minor*), 38.40 (*minor*), 36.69, 36.20 (*minor*), 32.73 (*minor*), 31.66 (*minor*), 30.43, 30.34, 28.45 (*minor*).

FTIR: 3332.81 (br), 2924.36 (s), 2852.83 (m), 1491.44 (s), 1091.79 (m), 1037.53 (m), 1013.84 (m), 819.68 (m), 526.36 (m).

HRMS (EI): Calculated for $C_{13}H_{15}ClO$ [M^+]: 222.0809, Found: 222.0804.

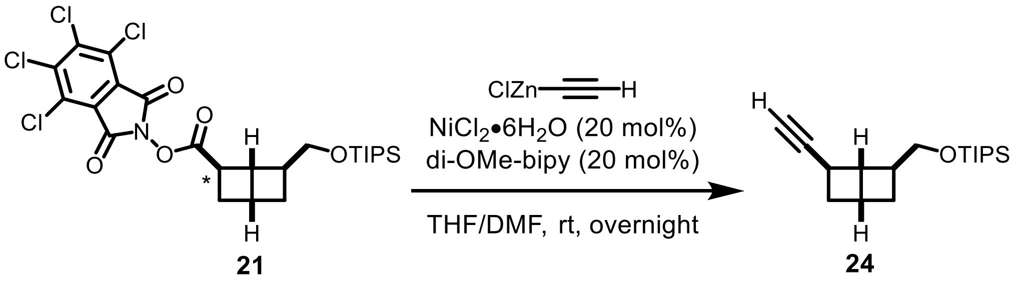

((6-ethynylbicyclo[2.2.0]hexan-2-yl)methoxy)triisopropylsilane (24): Procedure was adapted from literature.[3] A flame-dried 2-dram vial with stir bar was added redox-active ester **21** (0.0599 g, 1.00 equiv., 0.101 mmol, 10:1 dr). The vial was evacuated and backfilled with N_2 three times. A solution of $NiCl_2{\cdot}6H_2O$ and di-OMe-bipy (1 mL, 0.02 M in DMF, 20 mol% $NiCl_2{\cdot}6H_2O$, 20 mol% di-OMe-bipy) and ethynylzinc chloride solution **SI-3** (0.80 mL, 0.31 molar in THF, 2.5 equiv., 0.25 mmol) were added in quick succession. The reaction mixture was stirred at room temperature for approx. 18 hours. The reaction was quenched with 1 M HCl, and the aqueous layer was extracted with Et_2O three times. The combined organic layers were dried over $MgSO_4$, filtered, and concentrated under reduced pressure. The crude material was purified via MPLC (0% EtOAc in Hexanes for 1 min., 0% to 5% EtOAc over 1 min., 5% for 1 min., 5% to 10% over 1 min., 1 min. hold, 10% to 20% over 1 min., 1 min. hold, 20% to 100% EtOAc over 0.5 min., 8 min. hold) to yield a light-yellow oil (0.0138 g, 47% yield, >20:1 dr).

^{1}H NMR (500 MHz, CDCl$_3$) δ 3.73 – 3.64 (m, 2H), 3.18 – 3.11 (m, 1H), 2.73 (tdt, *J* = 7.8, 5.0, 2.7 Hz, 1H), 2.63 (t, *J* = 4.1 Hz, 1H), 2.48 (tt, *J* = 14.0, 6.9 Hz, 2H), 2.39 (ddt, *J* = 12.1, 8.3, 2.0 Hz, 1H), 2.29 (s, *J* = 2.0 Hz, 1H), 2.16 (dt, *J* = 13.0, 6.4 Hz, 1H), 2.12 – 2.03 (m, 1H), 1.06 (m, 21H).

^{13}C NMR (126 MHz, CDCl$_3$) δ 88.98, 70.21, 66.78, 45.49, 43.02, 35.66, 31.11, 30.03, 29.55, 18.20, 12.21.

FTIR: 3312.00 (m), 2925.37 (s), 2864.94 (s), 1462.70 (m), 1381.57 (m), 1248.40 (m), 1101.05 (m), 882.09 (m), 681.28 (m), 623.58 (m).

HRMS (EI): Calculated for $C_{15}H_{25}OSi$ [$M-C_3H_7^+$]: 249.1675, Found: 249.1669.

4. Synthesis of Ladder-Mazapertine

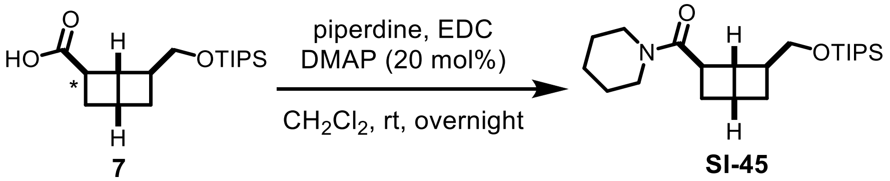

piperidin-1-yl(6-(((triisopropylsilyl)oxy)methyl)bicyclo[2.2.0]hexan-2-yl)methanone (SI-45): Carboxylic acid **7** (0.5761 g, 1.000 equiv., 1.843 mmol, 10:1 dr) was added to a flame-dried 50 mL round bottom flask with stir bar. The flask was evacuated and refilled with N_2 three times. CH_2Cl_2 (20 mL, 0.10 molar) was added to the flask followed by addition of EDC (0.3434 mg, 1.200 equiv., 2.212 mmol) and DMAP (0.0450

mg, 0.200 equiv., 0.369 mmol) in quick succession. The reaction mixture was flushed with N_2 before piperidine (0.22 mL, 1.2 equiv., 2.2 mmol) was added. After stirring at room temperature for approx. 18 hours, the reaction mixture was concentrated under reduced pressure. The crude mixture was purified via MPLC (10% EtOAc in Hexanes to 30% over 10 min., 30% to 100% EtOAc over 2.2 min., 8 min. hold) to yield a yellowish oil (0.3982 g, 57% yield, >20:1 dr).

^{1}H NMR (500 MHz, CDCl$_3$) δ 3.71 (dd, *J* = 9.9, 5.9 Hz, 1H), 3.69 – 3.62 (m, 2H), 3.43 (ddd, *J* = 12.6, 7.0, 4.6 Hz, 1H), 3.31 – 3.19 (m, 2H), 3.14 (ddd, *J* = 13.0, 7.5, 4.2 Hz, 1H), 2.94 (dt, *J* = 12.2, 7.5 Hz, 1H), 2.69 – 2.59 (m, 2H), 2.50 (p, *J* = 7.0 Hz, 1H), 2.19 – 1.99 (m, 3H), 1.68 – 1.42 (m, 6H), 1.05 (d, *J* = 5.4 Hz, 21H).

^{13}C NMR (126 MHz, CDCl$_3$) δ 172.68, 67.13, 45.94, 43.01, 42.95, 42.37, 42.34, 30.22, 29.90, 29.72, 26.40, 25.61, 24.71, 18.05, 12.02.

FTIR: 2935.07 (m), 2862.63 (m), 1644.32 (s), 1429.56 (m), 1253.32 (m), 1219.18 (m), 1092.81 (m), 881.34 (m), 679.52 (m).

HRMS (EI): Calculated for $C_{19}H_{34}NO_2Si$ [M-$C_3H_7^+$]: 336.2359, Found: 336.2359.

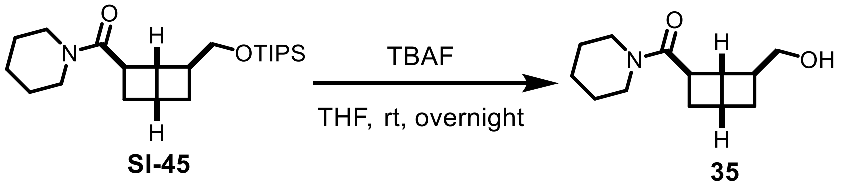

(6-(hydroxymethyl)bicyclo[2.2.0]hexan-2-yl)(piperidin-1-yl)methanone (35): Amide **SI-45** (0.3936 g, 1.000 equiv., 1.037 mmol, >20:1 dr) was added to a flame-dried scintillation vial equipped with a stir bar. THF (5 mL, 0.2 molar) was added to the flask followed by addition of TBAF (1.3 mL, 1.0 molar in THF, 1.3 equiv., 1.3 mmol). The reaction mixture stirred at room temperature for approx. 18 hours. The reaction mixture was quenched with sat. aq. NH_4Cl and the layers were separated. The aqueous layer was extracted four more times with CH_2Cl_2. The combined organic layers were dried over $MgSO_4$, filtered, and concentrated under reduced pressure. The crude mixture was purified via MPLC (50% EtOAc in Hexanes hold for 0.5 min., 50% to 100% EtOAc over 6 min., hold 8 min.) to yield a colorless oil (0.2287 g, 99% yield, >20:1 dr).

^{1}H NMR (500 MHz, CDCl$_3$) δ 3.72 – 3.56 (m, 3H), 3.49 (dt, *J* = 12.7, 5.9 Hz, 1H), 3.31 (td, *J* = 7.8, 2.9 Hz, 1H), 3.21 (q, *J* = 4.9 Hz, 2H), 2.86 (dt, *J* = 12.1, 7.6 Hz, 1H), 2.72 (d, *J* = 4.8 Hz, 1H), 2.66 (dtt, *J* = 7.9, 5.5, 2.5 Hz, 1H), 2.52 (p, *J* = 7.1 Hz, 1H), 2.19 (dddd, *J* = 14.3, 12.2, 8.0, 2.5 Hz, 2H), 2.05 (ddd, *J* = 13.0, 8.2, 5.6 Hz, 1H), 1.67 – 1.48 (m, 6H), 1.41 (q, *J* = 4.8 Hz, 1H).

^{13}C NMR (126 MHz, CDCl$_3$) δ 172.67, 66.86, 46.05, 43.03, 42.61, 42.39, 41.51, 30.73, 30.21, 30.02, 26.59, 25.74, 24.80.

FTIR: 3386.63 (br), 2923.64 (m), 2852.55 (m), 1612.84 (s), 1438.48 (m), 1252.18 (m), 1221.89 (m), 1045.59 (m), 852.12 (m), 543.70 (w).

HRMS (ESI): Calculated for $C_{13}H_{21}O_2NNa$ [M+Na^+]: 246.1465, Found: 246.1466.

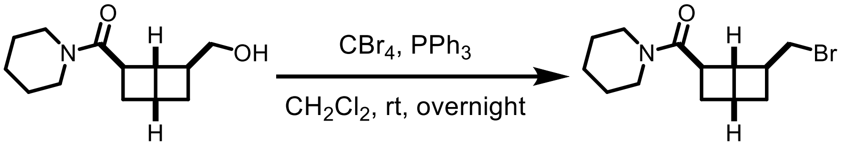

35 **SI-46**

(6-(bromomethyl)bicyclo[2.2.0]hexan-2-yl)(piperidin-1-yl)methanone (SI-46): Alcohol **35** (0.2500 g, 1.000 equiv., 1.120 mmol, >20:1 dr) was added to a flame-dried scintillation vial with stir bar. The vial was evacuated and refilled with N_2 three times. The vial was fitted with a septum and place under N_2. CH_2Cl_2 (5.6 mL, 0.20 molar) was added under N_2. The septum was removed, PPh_3 (0.3520 g, 1.200 equiv., 1.340 mmol) and CBr_4 (0.4450 mg, 1.200 equiv., 1.340 mmol) were added, and the septum quickly replaced. The vial was flushed with N_2 before it was stirred at room temperature for approx. 18 hours. The reaction mixture was concentrated and purified via MPLC (0% EtOAc in Hexanes over 0.5 min., 0% to 20% EtOAc over 0.5 min., 2 min. hold, 20% to 100% EtOAc over 3.5 min., 7 min. hold) to yield a white solid (0.2499 g, 78% yield, >20:1 dr).

^{1}H NMR (500 MHz, $CDCl_3$) δ 3.65 (dt, *J* = 12.8, 5.0 Hz, 1H), 3.52 (dd, *J* = 9.8, 7.0 Hz, 1H), 3.49 – 3.39 (m, 2H), 3.29 – 3.21 (m, 2H), 3.21 – 3.14 (m, 1H), 2.99 – 2.90 (m, 1H), 2.81 – 2.73 (m, 1H), 2.67 (dt, *J* = 6.4, 3.4 Hz, 2H), 2.29 (ddd, *J* = 13.1, 7.8, 2.3 Hz, 1H), 2.15 (ddd, *J* = 12.6, 8.5, 1.9 Hz, 1H), 2.05 (ddd, *J* = 14.5, 7.2, 4.3 Hz, 1H), 1.70 – 1.47 (m, 6H).

^{13}C NMR (126 MHz, $CDCl_3$) δ 172.16, 46.14, 43.93, 43.21, 43.06, 42.33, 38.48, 33.28, 29.96, 29.00, 26.80, 25.72, 24.77.

FTIR: 2938.87 (m), 2924.55 (m), 2852.23 (m), 1627.39 (s), 1431.17 (s), 1278.52 (m), 1251.92 (m), 597.06 (m).

HRMS (ESI): Calculated for $C_{13}H_{21}ONBr$ [M+H$^+$]: 286.0801, Found: 286.0802.

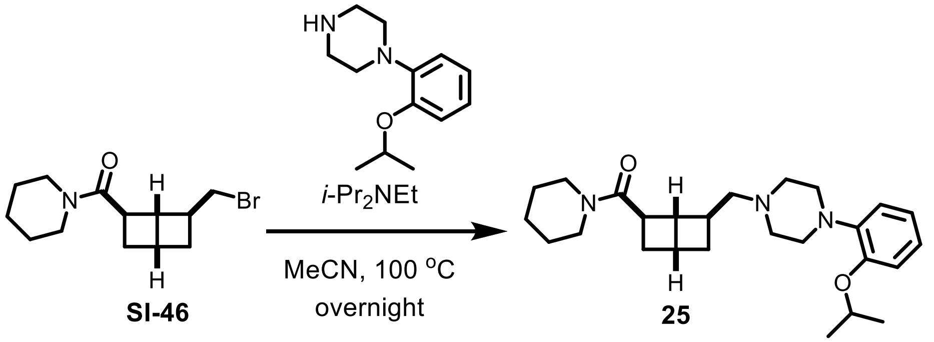

SI-46 **25**

Ladderane-Mazapertine (25): Bromide **SI-46** (0.0098 g, 1.0 equiv., 0.034 mmol, >20:1 dr) and piperazine **SI-6** (0.0220 mg, 2.9 equiv., 0.10 mmol) were added to a flame-dried screw cap test tube vial with stir bar. The vial was capped with a septum, and the vial was evacuated and refilled with N_2 three times. The vial was placed under N_2, and MeCN (0.34 mL, 0.10 molar) and *i*-Pr_2NEt (0.06 mL, 10 equiv., 0.34 mmol) were added. The septum was quickly replaced with a Teflon screwcap and sealed with Teflon tape. The reaction was heated to 100 °C for approx. 18 hours in an aluminum block. The reaction mixture was cooled to room temperature, diluted with 1 M KOH, and extracted with EtOAc. The layers were separated, and the

aqueous layer was extracted twice more with EtOAc. The combined organic layers were dried over $MgSO_4$, filtered, and concentrated under reduced pressure. The crude material was purified via MPLC (50% EtOAc in Hexanes to 100% EtOAc over 7.5 min., 7.5 min. hold) to yield a yellowish oil (0.0128 g, 88% yield, >20:1 dr).

^{1}H NMR (500 MHz, $CDCl_3$) δ 6.98 – 6.83 (m, 4H), 4.59 (hept, *J* = 6.1 Hz, 1H), 3.63 – 3.46 (m, 2H), 3.27 – 2.98 (m, 7H), 2.91 (dt, *J* = 13.8, 7.5 Hz, 1H), 2.71 – 2.46 (m, 8H), 2.27 – 2.12 (m, 2H), 2.04 (dt, *J* = 13.0, 6.5 Hz, 1H), 1.66 – 1.48 (m, 7H), 1.34 (d, *J* = 6.1 Hz, 6H).

^{13}C NMR (126 MHz, $CDCl_3$) δ 172.77, 150.45, 143.02, 122.50, 121.59, 118.46, 116.35, 70.41, 64.80, 54.04, 50.55, 46.03, 43.76, 43.00, 42.70, 38.41, 32.55, 30.19, 30.14, 29.85, 26.72, 25.71, 24.80, 22.47, 22.45.

FTIR: 2925.10 (s), 2852.52 (m), 2810.54 (m), 1641.78 (s), 1495.47 (m), 1435.02 (m), 1236.90 (s), 1138.43 (m), 747.49 (m).

HRMS (ESI): Calculated for $C_{26}H_{40}O_2N_3$ [M+H$^+$]: 426.3115, Found: 426.3121.

Functional Group Manipulations of *endo*-[2]-Ladderane

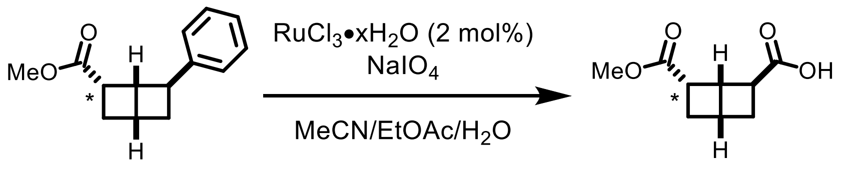

5a **27**

6-(methoxycarbonyl)bicyclo[2.2.0]hexane-2-carboxylic acid (27): Procedure was adapted from literature.[11] Ester **5a** (817 mg, 3.78 mmol, 1 equiv., 4:1 dr) was added to a 100 ml round bottom flask equipped with a stir-bar. MeCN (7.6 ml), EtOAc (7.6 ml), and DI H_2O (11.5 ml) were added to the flask. While the mixture stirred vigorously, $NaIO_4$ (11.67 g, 54.81 mmol, 14.5 equiv.) was added followed by addition of $RuCl_3{\cdot}xH_2O$ (16 mg, 0.076 mmol, 0.02 equiv.). The flask was fitted with a reflux condenser, and the reaction stirred vigorously at room temperature open to air. The reaction started refluxing after approx. 15 min., and the reaction occasionally needed the use of spatula to aid stirring due to the amount of white precipitate formed. After approx. 18 hours, the reaction was quenched with water (10 ml). The aqueous layer was extracted with EtOAc five times and TLC of the aqueous layer was taken to ensure compete extraction. The combined organic layers were dried over Na_2SO_4, filtered, and concentrated under reduced pressure. The crude material was purified via flash column chromatography (50-60% EtOAc in Hexane) to yield a viscous oil (497 mg, 54% yield, 6:1 dr).

^{1}H NMR (400 MHz, CD_3OD) δ 3.69 (s, 3.5H), 3.60 (dt, *J* = 10.3, 8.1 Hz, 1H), 3.39 – 3.33 (m, 1H), 3.24 (dd, *J* = 7.6, 4.3 Hz, 1.15H), 3.07 (m, 0.15H), 2.76 (m, 0.15H), 2.72 – 2.55 (m, 3.3H), 2.47 (ddd, *J* = 12.6, 8.4, 3.8 Hz, 1H), 2.36 (t, *J* = 10.5 Hz, 0.3H), 2.24 – 2.14 (m, 1H).

^{13}C NMR (126 MHz, CD_3OD) δ 178.60, 175.37, 52.35 (*minor*), 51.99, 44.62 (*minor*), 44.49 (*minor*), 43.51 (*minor*), 43.04, 40.06, 39.93, 32.80, 32.65 (*minor*), 31.77 (*minor*), 31.66 (*minor*), 30.78, 30.26.

FTIR: 3320 (br), 2983 (m), 2950 (m), 1728 (s), 1702 (s), 1473 (m), 1354 (w), 1203 (s).

HRMS (ESI): Calculated for $C_9H_{12}O_4Na$ [M+Na$^+$]: 207.0628, Found: 207.0629.

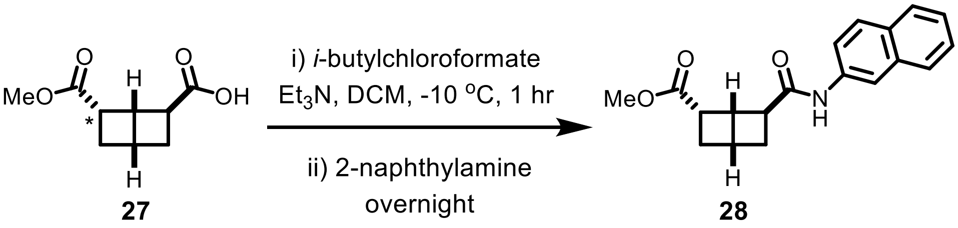

methyl 6-(naphthalen-2-ylcarbamoyl)bicyclo[2.2.0]hexane-2-carboxylate (28): Procedure was adapted from literature.[17] Carboxylic acid **27** (36 mg, 0.2 mmol, 1 equiv., 6:1 dr) was added to a flame-dried 2-dram vial equipped with a stir bar and capped with a septum. CH_2Cl_2 (2 ml) was added to the vial, and the resulting solution was cooled to –10 °C. Et_3N (0.36 ml, 0.2 mmol, 1.3 equiv.) was added to the solution followed by addition of *i*-butylchloroformate (0.031 ml, 0.24 mmol, 1.2 equiv.). After stirring at -10 °C for 1.5 hrs., 2-naphthylamine (37 mg, 0.26 mmol, 1.3 equiv.) was added to the reaction mixture. The septum was replaced with a screw cap, and the reaction warmed to room temperature over approx. 18 hours. The reaction mixture was quenched with sat. aq. NH_4Cl, and the aqueous layer was extracted with CH_2Cl_2 three times. The combined organic layers were dried over Na_2SO_4, filtered, and concentrated under reduced pressure. The crude material was purified via flash column chromatography (30-40% EtOAc in hexane) to yield amide **32** (22 mg, 67% yield, >20:1 dr) as a reddish solid.

^{1}H NMR (500 MHz, CDCl$_3$) δ 8.26 (d, *J* = 2.1 Hz, 1H), 8.16 (s, 1H), 7.82 – 7.74 (m, 3H), 7.50 (dd, *J* = 8.8, 2.2 Hz, 1H), 7.44 (t, *J* = 7.5 Hz, 1H), 7.41 – 7.34 (m, 1H), 3.82 (s, 3H), 3.66 – 3.53 (m, 2H), 3.22 – 3.15 (m, 1H), 2.94 (dt, *J* = 12.1, 8.3 Hz, 1H), 2.81 (q, *J* = 11.0 Hz, 1H), 2.70 (p, *J* = 6.3 Hz, 1H), 2.54 (ddd, *J* = 12.9, 8.9, 4.5 Hz, 1H), 2.01 (dd, *J* = 12.1, 7.8 Hz, 1H).

^{13}C NMR (126 MHz, CDCl$_3$) δ 175.35, 172.37, 136.19, 134.14, 130.47, 128.77, 127.73, 127.65, 126.51, 124.76, 119.77, 115.75, 52.10, 43.42, 41.77, 39.08, 30.90, 29.04, 29.01.

FTIR: 3324 (br), 2926 (m), 1727 (s), 1695 (s), 1604 (w), 1585 (s), 1433 (m), 1224 (m), 858 (m).

HRMS (ESI): Calculated for $C_{19}H_{19}O_3NNa$ [M+Na$^+$]: 332.1257, Found: 332.1259.

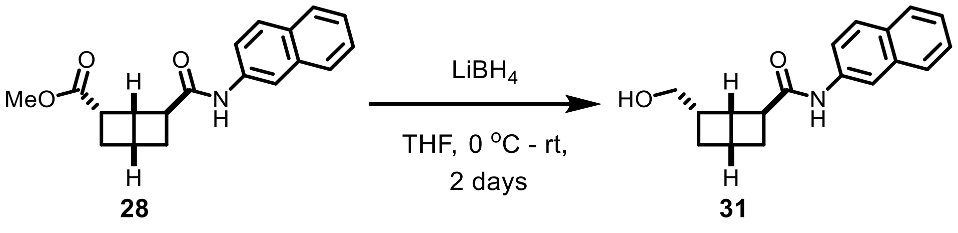

6-(hydroxymethyl)-N-(naphthalen-2-yl)bicyclo[2.2.0]hexane-2-carboxamide (31): Ester **28** (31 mg, 0.1 mmol, 1 equiv., >20:1 dr) was added to a flame dried 2-dram vial equipped with a stir bar and capped with a septum. THF (0.3 ml) was added, and the reaction mixture was cooled to 0 °C. The septum was removed, $LiBH_4$ (8.5 mg, 0.4 mmol, 4 equiv.) was added, and the septum quickly replaced. The reaction was brought

to room temperature and the septum was replaced with a screw cap. The reaction stirred for 2 days at room temperature. After the consumption of the starting material, the reaction mixture was quenched with DI H_2O, and the aqueous layer was extracted with ethyl acetate three times. The combined organic layers were dried over Na_2SO_4, filtered, concentrated under reduced pressure. The crude material was purified via flash column chromatography (40-50% EtOAc in Hexane) to yield a clear, colorless oil (16 mg, 57% yield, >20:1 dr).

^{1}H NMR (500 MHz, CDCl$_3$) δ 8.22 – 8.12 (m, 2H), 7.77 – 7.70 (m, 3H), 7.41 (dd, *J* = 8.7, 2.4 Hz, 2H), 7.36 (t, *J* = 7.5 Hz, 1H), 3.96 – 3.83 (m, 2H), 3.65 (td, *J* = 8.2, 4.3 Hz, 1H), 3.10 (qd, *J* = 5.8, 1.9 Hz, 1H), 3.02 – 2.87 (m, 2H), 2.65 (tt, *J* = 7.7, 4.6 Hz, 1H), 2.51 – 2.30 (m, 2H), 1.99 (dd, *J* = 12.2, 7.9 Hz, 1H), 1.80 (ddd, *J* = 12.6, 8.3, 4.3 Hz, 1H).

^{13}C NMR (126 MHz, CDCl$_3$) δ 174.03, 136.17, 134.07, 130.46, 128.72, 127.68, 127.64, 126.51, 124.80, 119.82, 115.95, 62.90, 43.11, 38.09, 35.48, 30.06, 29.97, 29.06.

FTIR: 3293 (br), 3055 (s), 2925 (m), 1662 (s), 1604 (s), 1549 (s), 1470 (w), 1393(w), 887 (m).

HRMS (ESI): Calculated for $C_{18}H_{19}O_2NNa$ [M+Na$^+$]: 304.1308, Found: 304.1310.

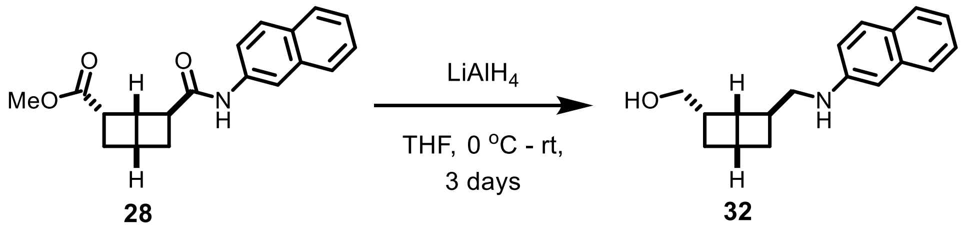

(6-((naphthalen-2-ylamino)methyl)bicyclo[2.2.0]hexan-2-yl)methanol (32): Ester **28** (31 mg, 0.1 mmol, 1 equiv., >20:1 dr) was added to a flame-dried 2-dram vial equipped with a stir bar. The vial was capped with a septum, and THF (0.3 ml) was added. This resulting solution was cooled down to 0 °C followed by addition of $LiAlH_4$ (12 mg, 0.3 mmol, 3 equiv.). The reaction mixture was brought to room temperature, and the septum was replaced with a screw cap. The reaction was stirred for approx. 18 hours at room temperature. The reaction was quenched with saturated NaOH solution and diluted with ethyl acetate. The organic layer was passed through a pad of celite and concentrated under reduced pressure. The crude material was purified via flash column chromatography (40-70% EtOAc in Hexane) to yield a clear, colorless oil (10 mg, 37% yield, >20:1 dr).

^{1}H NMR (500 MHz, CDCl$_3$) δ 7.70 – 7.57 (m, 3H), 7.35 (t, J = 7.6 Hz, 1H), 7.18 (t, J = 7.6 Hz, 1H), 6.89 – 6.78 (m, 2H), 3.83 (t, J = 10.3 Hz, 1H), 3.72 (ddd, J = 10.6, 6.5, 1.6 Hz, 1H), 3.35 (dd, J = 11.5, 6.2 Hz, 1H), 3.24 – 3.14 (m, 1H), 3.03 (q, J = 7.8 Hz, 1H), 2.86 (hept, J = 7.8 Hz, 1H), 2.75 – 2.64 (m, 2H), 2.55 – 2.45 (m, 1H), 2.21 – 2.03 (m, 3H), 1.78 (ddd, J = 12.4, 8.5, 3.1 Hz, 1H).

^{13}C NMR (126 MHz, CDCl$_3$) δ 146.33, 135.42, 129.00, 127.78, 127.56, 126.41, 125.99, 121.94, 118.13, 104.34, 63.09, 49.94, 41.62, 36.11, 33.20, 32.08, 30.53, 29.28.

FTIR: 3355 (br), 2920 (s), 2848 (w), 1628 (s), 1602 (m), 1468 (m), 1398 (w), 1259 (m), 825 (m).

HRMS (ESI): Calculated for $C_{18}H_{22}ON$ [M+H$^+$]: 268.1696, Found: 268.1697.

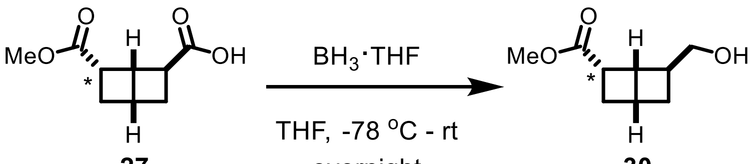

methyl 6-(hydroxymethyl)bicyclo[2.2.0]hexane-2-carboxylate (30): Carboxylic acid **27** (18.4 mg, 0.1 mmol, 1 equiv., 6:1 dr) was added to a flame-dried 2-dram vial equipped with a stir bar and capped with a septum. THF (0.1 ml) was added, and the resulting solution was cooled to 0 °C in an ice bath. BH_3·THF (0.1 ml, 1 M in THF, 1 equiv.) was added dropwise to the reaction mixture. The reaction was brought to room temperature, the septum replaced with a screw cap, and the reaction stirred for approx. 18 hours at room temperature. The reaction mixture was quenched with DI H_2O, and the aqueous layer was extracted with EtOAc three times. The combined organic layers were dried over Na_2SO_4, filtered, and concentrated under reduced vacuum. The crude material was purified via flash column chromatography (30-40% EtOAc in Hexane) to yield a clear, colorless oil (14 mg, 82% yield, 6:1 dr).

^{1}H NMR (400 MHz, $CDCl_3$) δ 3.69 (d, *J* = 4.6 Hz, 3.45H), 3.67 – 3.53 (m, 2.3H), 3.49 (dt, *J* = 10.8, 8.0 Hz, 1H), 3.24 (t, *J* = 8.4 Hz, 0.15H), 2.87 – 2.79 (m, 1H), 2.76 – 2.54 (m, 3.45H), 2.54 – 2.45 (m, 1H), 2.28 (t, *J* = 9.4 Hz, 0.15H), 2.22 – 2.14 (m, 0.15H), 2.12 – 1.98 (m, 2.3H), 1.44 (s, 1H).

^{13}C NMR (126 MHz, $CDCl_3$) δ 174.59, 66.91, 51.90 (*minor*), 51.63, 43.19 (*minor*), 41.20, 39.07, 38.38, 31.26 (*minor*), 31.22 (*minor*), 30.93, 30.18 (*minor*), 30.12, 29.40.

FTIR: 3400 (br), 2949 (m), 2854 (w), 1728 (s), 1436 (m), 1344 (w), 1199 (s), 1052 (m).

HRMS (ESI): Calculated for $C_9H_{14}O_3Na$ [M+Na$^+$]: 193.0835, Found: 193.0835.

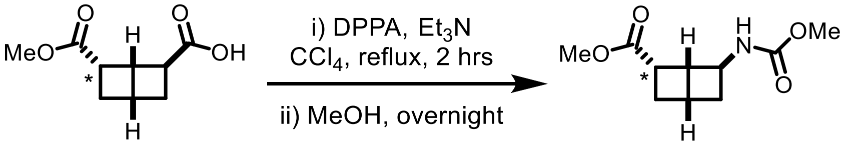

methyl 6-((methoxycarbonyl)amino)bicyclo[2.2.0]hexane-2-carboxylate (29): Procedure was adapted from literature.[9] Carboxylic acid **27** (100 mg, 0.54 mmol, 1 equiv., 6:1 dr) was added to a flame-dried 2-dram vial equipped with a stir bar and capped with a septum. CCl_4 (1 ml) was added to the flask followed by Et_3N (0.08 ml, 0.57 mmol, 1.05 equiv.). The solution was warmed just under reflux and DPPA (0.12 ml, 0.54 mmol, 1 equiv.) was added dropwise. The septum was quickly replaced with a screw cap, and the reaction was refluxed for 2 hrs. The disappearance of the acid was observed by TLC, and the reaction was cooled to room temperature. MeOH (0.026 ml, 0.65 mmol, 1.2 equiv.) was added under N_2, and the reaction was stirred at room temperature for approx. 18 hours. The reaction mixture was evaporated to dryness and diluted with sat. aq. $NaHCO_3$. The aqueous layer was extracted with Et_2O three times. The combined organic layers were dried over Na_2SO_4, filtered, and concentrated under reduced vacuum. The crude material was purified via flash column chromatography (7-10% EtOAc in Hexane) to yield a clear, colorless oil. (61 mg, 53% yield, 4:1 dr).

^{1}H NMR (500 MHz, C_6D_6, 47 °C) δ 4.50 (s, 1H), 4.37 (d, *J* = 2.9 Hz, 0.25H), 4.32 – 4.16 (s, 1H), 4.10 (s, 0.25H), 3.54 (s, 3H), 3.44 (d, *J* = 1.6 Hz, 0.75H), 3.42 (d, *J* = 1.7 Hz, 3.25H), 3.36 (d, *J* = 2.1 Hz, 0.75H), 3.12 (dd, *J* = 10.3, 8.0 Hz, 1H), 2.93 (d, *J* = 7.5 Hz, 0.25H), 2.66 (s, 0.25H), 2.58 (s, 1H), 2.42 (dd, *J* = 11.2, 7.4 Hz, 1.25H), 2.19 – 2.01 (m, 3.25H), 1.86 (t, *J* = 10.6 Hz, 0.25H), 1.69 (s, 1H).

^{13}C NMR (126 MHz, C_6D_6, 47 °C) δ 172.92, 155.73, 51.58, 51.19, 48.40, 48.10, 47.78 (*minor*), 42.10 (*minor*), 38.22, 37.61, 36.70 (*minor*), 30.58 (*minor*), 29.73 (*minor*), 29.03, 27.39.

FTIR: 3329 (w), 2955 (w), 1727 (s), 1546 (m), 1438 (w), 1204 (m).

HRMS (ESI) Calculated for $C_{10}H_{15}O_4NNa$ [M+Na$^+$]: 236.0893, Found: 236.0895.

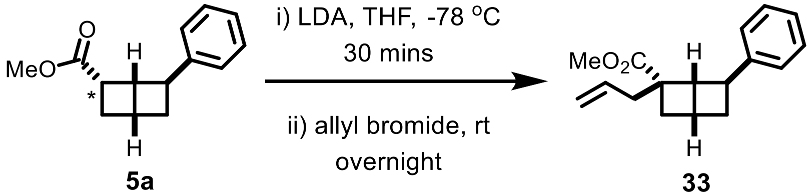

5a 33

methyl 2-allyl-6-phenylbicyclo[2.2.0]hexane-2-carboxylate (33): Ester **5a** (21.6 mg, 0.1 mmol, 1 equiv., 4:1 dr) was added to a flame-dried 2-dram vial equipped with a stir bar and capped with a septum. THF (0.3 ml) was added, and the resulting solution was cooled to -78 °C in a dry ice/acetone bath. Freshly prepared LDA (0.16 ml, 0.75 M in THF, 1.2 equiv.) was added dropwise to the ester **5a** solution. The reaction was stirred at -78 °C. After 1 h, allyl bromide (0.013 ml, 0.15 mmol, 1.5 equiv.) was added dropwise to the reaction mixture, and the septum was quickly replaced with a screw cap. The reaction stirred for approx. 18 hours while warming to room temperature. The reaction was quenched with DI H_2O, and the aqueous layer was extracted with EtOAc three times. The combined organic layers were dried over Na_2SO_4, filtered, and concentrated under reduced vacuum. The crude material was purified via flash column chromatography (2% Ether in Pentane) to yield a clear, colorless oil (22 mg, 85% yield, >20:1 dr).

^{1}H NMR (400 MHz, $CDCl_3$) δ 7.31 (t, *J* = 7.6 Hz, 2H), 7.24 – 7.15 (m, 3H), 5.74 (ddt, *J* = 17.3, 10.2, 7.1 Hz, 1H), 5.15 – 5.05 (m, 2H), 3.71 (s, 3H), 3.66 (td, *J* = 8.3, 4.1 Hz, 1H), 2.82 – 2.70 (m, 2H), 2.68 – 2.49 (m, 3H), 2.46 – 2.30 (m, 3H).

^{13}C NMR (126 MHz, $CDCl_3$) δ 175.98, 145.97, 133.22, 128.48, 126.52, 125.95, 118.18, 52.01, 51.63, 51.58, 49.78, 42.43, 40.92, 34.68, 34.56, 26.50.

FTIR: 3026 (m), 2952 (m), 1730 (s), 1603 (w), 1447 (w), 1283 (m), 748 (m).

HRMS (ESI) Calculated for $C_{17}H_{20}O_2Na$ [M+Na$^+$]: 279.1356, Found: 279.1357.

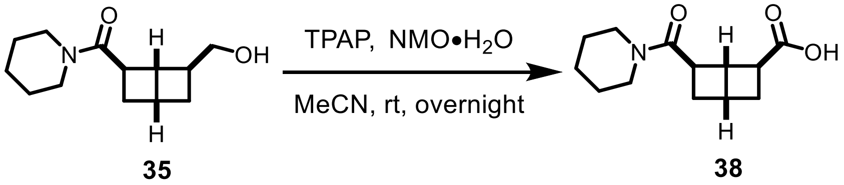

6-(piperidine-1-carbonyl)bicyclo[2.2.0]hexane-2-carboxylic acid (38): Procedure was adapted from literature.[13] Alcohol **35** (0.0860 g, 1.00 equiv., 0.385 mmol) and NMO·H_2O (0.4510 g, 10.00 equiv., 3.850 mmol) were added to a 1-dram vial with stir bar. The 1-dram vial was evacuated and backfilled with N_2 three times. The vial was fitted with a septum and placed under N_2. MeCN (2.0 mL, 0.20 molar) was added, and the solution was allowed to stir for 5 min. The septum was removed and TPAP (0.0135 mg, 0.100 equiv., 0.0385 mmol) was added, the septum replaced, and the vial flushed with N_2 for 2 min. The reaction mixture stirred at room temperature for approx. 18 hours. *i*-PrOH (0.25 mL) was added to the reaction mixture, and the solution was allowed to stir for 30 minutes. The reaction mixture was concentrated on the rotovap to half volume before adding 1 M HCl and EtOAc. The layers were separated, and the aqueous layer was extracted four more times with EtOAc. The combined organic layers were washed with 1 M HCl until the organic layer was colorless (approx. 5 washes). The organic layer was then washed with brine, dried over $MgSO_4$, filtered, and concentrated under reduced pressure. The crude material was purified via MPLC (0% EtOAc in Hexanes for 0.7 min., 0% to 100% EtOAc over 5.3 min., 9.2 min. hold) to yield a white solid (0.0566 g, 62% yield, >20:1 dr).

^{1}H NMR (500 MHz, $CDCl_3$) δ 3.63 – 3.48 (m, 2H), 3.38 – 3.32 (m, 1H), 3.29 – 3.20 (m, 3H), 3.14 (t, *J* = 3.5 Hz, 1H), 2.88 (dt, *J* = 12.5, 6.8 Hz, 1H), 2.77 (t, *J* = 6.5 Hz, 2H), 2.35 (t, *J* = 9.4 Hz, 1H), 2.29 – 2.22 (m, 1H), 1.68 – 1.49 (m, 6H).

^{13}C NMR (126 MHz, $CDCl_3$) δ 179.33, 171.92, 46.19, 43.53, 43.16, 43.00, 42.39, 30.65, 30.40, 30.37, 26.47, 25.70, 24.73.

FTIR: 2921.15 (s), 2852.23 (m), 1720.32 (s), 1588.22 (s), 1448.23 (m), 1227.70 (m), 1175.56 (s), 855.84 (w), 687.43 (m).

HRMS (ESI): Calculated for $C_{13}H_{19}O_3NNa$ [M+Na$^+$]: 260.1257, Found: 260.1258.

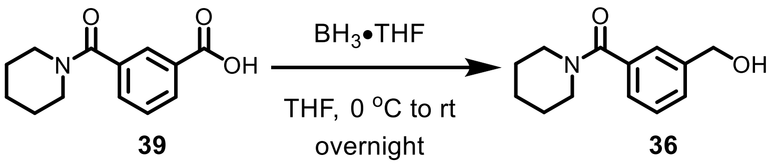

(3-(hydroxymethyl)phenyl)(piperidin-1-yl)methanone (36): 3-(piperidine-1-carbonyl)benzoic acid **39** (0.1000 g, 1.000 equiv., 0.4290 mmol) was added to a flame-dried 1-dram vial with stir bar. The vial was evacuated and refilled with N_2 three times. THF (0.5 mL, 1.0 molar) was added, and the solution was cooled to 0 °C. BH_3·THF (0.90 mL, 1.0 molar, 2.0 equiv., 0.90 mmol) was added was added dropwise via syringe, and the reaction mixture was warmed to room temperature over approx. 18 hours. The solution

was cooled back down to 0 °C before being quenched with DI H_2O. The reaction mixture was diluted with EtOAc, and the layers were separated. The aqueous layer was extracted with EtOAc twice more. The combined organic layers were dried over $MgSO_4$, filtered, and concentrated under reduced pressure. The crude material was purified via MPLC (50% EtOAc in Hexanes to 100% EtOAc over 5 min., 10.8 min. hold) to yield a colorless oil (0.0159 g, 17% yield).

^{1}H NMR (500 MHz, $CDCl_3$) δ 7.43 – 7.35 (m, 3H), 7.29 (dd, *J* = 6.9, 1.7 Hz, 1H), 4.72 (d, *J* = 5.9 Hz, 2H), 3.71 (s, 2H), 3.34 (s, 2H), 1.79 (t, *J* = 5.9 Hz, 1H), 1.68 (d, *J* = 4.8 Hz, 4H), 1.58 – 1.48 (m, 2H).

^{13}C NMR (126 MHz, $CDCl_3$) δ 170.30, 141.50, 136.99, 128.73, 127.92, 126.02, 125.41, 65.08, 48.93, 43.28, 26.72, 25.78, 24.74.

FTIR: 3385.86 (br), 2929.04 (m), 2855.14 (m), 1613.34 (s), 1445.11 (m), 1286.36 (m), 1208.39 (m), 1027.11 (m), 745.86 (w).

HRMS (ESI): Calculated for $C_{13}H_{18}O_2N$ [M+H$^+$]: 220.1332, Found: 220.1332.

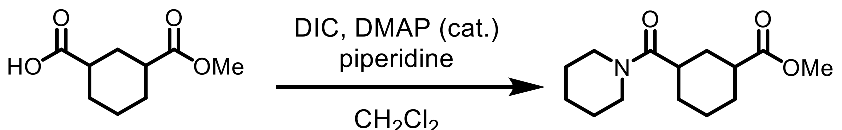

SI-47

methyl 3-(piperidine-1-carbonyl)cyclohexane-1-carboxylate (SI-47): 3-(methoxycarbonyl)cyclohexane-1-carboxylic acid (0.5000 g, 2.685 mmol, 1.000 equiv.) and DMAP (65 mg, 0.54 mmol, 0.20 equiv.) were added to a flame-dried 50 mL round bottom flask with stir bar. It was evacuated and refilled with N_2 three times. Under N_2, CH_2Cl_2 (30 mL, 0.1 M) was added followed by piperidine (0.30 mL, 3.2 mmol, 1.2 equiv.) and stirred at room temperature for approx. 18 hours. The reaction mixture was concentrated under reduced pressure. The crude material was purified via MPLC (50% EtOAc in Hexanes to 100% EtOAc over 6.5 min., 7 min. hold) to yield a clear, colorless oil (0.2010 g, 30% yield).

^{1}H NMR (500 MHz, $CDCl_3$) δ 3.66 (s, 3H), 3.54 (t, J = 5.6 Hz, 2H), 3.41 (t, J = 5.4 Hz, 2H), 2.52 (tt, J = 11.9, 3.4 Hz, 1H), 2.34 (tt, J = 12.4, 3.5 Hz, 1H), 2.01 – 1.94 (m, 2H), 1.90 (dp, J = 13.5, 3.2 Hz, 1H), 1.76 – 1.68 (m, 2H), 1.68 – 1.48 (m, 7H), 1.48 – 1.28 (m, 2H).

^{13}C NMR (126 MHz, $CDCl_3$) δ 175.79, 173.32, 51.78, 46.61, 43.05, 42.94, 39.75, 31.73, 28.80, 28.47, 27.01, 25.77, 25.19, 24.81.

FTIR: 2936.96 (m), 2858.44 (m), 1732.35 (s), 1631.30 (s), 1441.38 (s), 1254.02 (s), 1209.24 (m), 1171.05 (w), 984.93 (w).

HRMS (ESI): Calculated for $C_{14}H_{23}O_3NNa$ [M+Na$^+$]: 276.1570, Found: 276.1572.

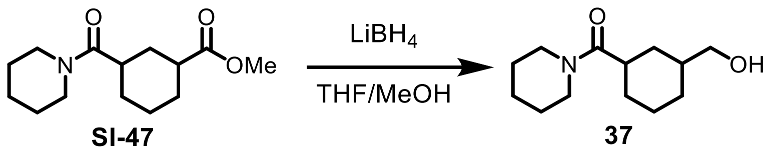

(3-(hydroxymethyl)cyclohexyl)(piperidin-1-yl)methanone (37) and scheme labels are below.

(3-(hydroxymethyl)cyclohexyl)(piperidin-1-yl)methanone (37): $LiBH_4$ (28.4 mg, 1.30 mmol, 4.00 equiv.) was added to a flame-dried 2 dram vial with stir bar. The vial was evacuated and backfilled with N_2 three times. The $LiBH_4$ was suspended in 1.5 mL THF and cooled to 0 °C in an ice bath. 2 mL of THF were used to aid transfer of ester **SI-47** (0.0825 g, 0.326 mmol, 1.00 equiv.) to the vial containing $LiBH_4$. Anhydrous methanol (70 µL, 5 M) was added to the reaction vial and was allowed to room temperature for approx. 18 hours. The reaction mixture was quenched with DI H_2O at 0 °C and extracted with EtOAc five times. The combined organic layers were dried over $MgSO_4$, filtered, and concentrated under reduced pressure. The crude material was purified via MPLC (100% Hexanes over 0.7 min., 0% to 100% EtOAc in Hexanes over 8.1 min., 17 min. hold) to afford a clear, colorless oil (0.0160 g, 22% yield).

^{1}H NMR (500 MHz, $CDCl_3$) δ 3.58 – 3.38 (m, 6H), 2.53 (tt, J = 11.8, 3.4 Hz, 1H), 1.86 (dt, J = 13.2, 3.3 Hz, 1H), 1.83 – 1.69 (m, 3H), 1.68 – 1.40 (m, 9H), 1.37 – 1.20 (m, 3H), 1.00 – 0.90 (m, 1H).

^{13}C NMR (126 MHz, $CDCl_3$) δ 174.17, 68.58, 46.60, 42.89, 40.08, 39.94, 32.38, 29.57, 28.97, 27.01, 25.80, 25.38, 24.84.

FTIR: 3395.63 (br), 2928.66 (m), 2853.84 (m), 2362.14 (w), 2160.86 (w), 2017.79 (w), 1614.68 (s), 1443.33 (m), 1226.76 (w), 1015.75 (w).

HRMS (ESI): Calculated for $C_{13}H_{23}O_2NNa$ [M+Na$^+$]: 248.1621, Found: 248.1621.

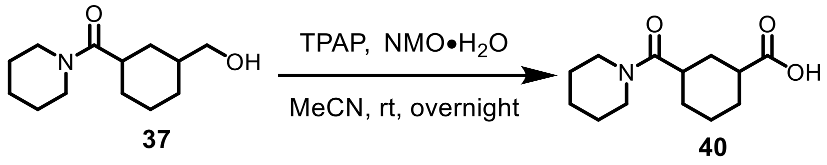

3-(piperidine-1-carbonyl)cyclohexane-1-carboxylic acid (40): A 1-dram vial equipped with a stir bar was flame-dried under vacuum. The 1-dram vial was allowed to cool under vacuum and backfilled with N_2. Alcohol **37** (0.016 g, 0.071 mmol, 1.0 equiv.) and NMO (0.083 g, 0.71 mmol, 10 equiv.) were added to the 1-dram vial. The 1-dram vial was evacuated and backfilled with N_2 three times. MeCN (0.4 mL, 0.2 molar) was added under N_2, and the reaction mixture was allowed to stir for approximately 5 minutes. TPAP (0.0051 mg, 0.014 mmol, 0.2 equiv.) was added to the vial, and the resulting green-black solution was allowed to stir at room temperature for approx. 18 hours. *I*-PrOH was added to the reaction, and the solution was allowed to stir for 30 minutes. The crude reaction was quenched upon addition of 1 M HCl and EtOAc. The aqueous layer was extracted with EtOAc three times. The combined organic layers were washed with 1 M HCl five times (until the organic layer ran clear), then with brine. The combined organic layers were dried over $MgSO_4$, filtered, and concentrated under reduced pressure to afford **40** as an off-white solid (0.0105 g, 62% yield).

^{1}H NMR (500 MHz, $CDCl_3$) δ 3.55 (t, *J* = 5.6 Hz, 2H), 3.42 (t, *J* = 5.4 Hz, 2H), 2.54 (tt, *J* = 11.9, 3.4 Hz, 1H), 2.36 (tt, *J* = 12.4, 3.5 Hz, 1H), 2.05 – 1.98 (m, 2H), 1.91 (dp, *J* = 13.3, 3.2 Hz, 1H), 1.77 – 1.69 (m, 2H), 1.67 – 1.61 (m, 2H), 1.61 – 1.49 (m, 4H), 1.49 – 1.30 (m, 2H).

^{13}C NMR (126 MHz, $CDCl_3$) δ 180.18, 173.40, 46.67, 43.05, 42.69, 39.72, 31.49, 28.72, 28.23, 27.00, 25.76, 25.10, 24.78.

FTIR: 3435.28 (w, br), 2926.55 (s), 2855.84 (m), 1721.66 (s), 1596.65 (s), 1445.29 (s), 1254.25 (m), 1219.00 (m), 1176.72 (m), 1023.45 (w), 853.69 (w), 730.41 (w).

HRMS (ESI): Calculated for $C_{13}H_{21}O_3NNa$ [M+Na$^+$]: 262.1414, Found: 262.1415.

ADME Procedures

Microsomal Clearance:

The following procedure was adapted from the literature.[14] The experiment was performed in 96-well plate format with shaking incubation at 37 °C and 750 rpm. The compounds in 10 mM DMSO were diluted 1:1000 in 100 mM potassium phosphate (Kpi) buffer to 10 µM. 30 µL of the compound solution was added to 120 µL of pretreated microsomal protein for 150 µL enzyme-substrate mixture. Reactions were initiated by addition of 150 µL of cofactor solution (2 mM NADPH, 4 mM $MgCl_2$ in 100 mM KPi). At specific reaction time points (0, 5, 15 and 30 minutes), reaction aliquots (25 µL) were removed and terminated by addition to acetonitrile (150 µL) containing mass spectrometry internal standard (0.4 µM glyburide). The samples were then centrifuged at 500g, and the supernatants analyzed by LC-MS/MS for quantitation of remaining target compound. The percentage of target compound remaining, relative to 0 minutes, was used to estimate *in vitro* elimination-rate constant (k_{mic}).

High Throughput Equilibrium Solubility:

The following procedure was adapted from the literature.[15] Equilibrium solubility was determined using a miniaturized shake flask approach as described in Zhou et al. Aliquots of 10 mM DMSO compound solution were dispensed in triplicate (2.5 µL/sample) in 96- well polypropylene plates using a Labcyte Echo 525 acoustic dispenser. The DMSO was removed using a GeneVac HT4X evaporator for 15 minutes, 35 °C and full vacuum. Media (pH 6.8 Di-Sodium Hydrogen Phosphate/Potassium Hydrogen Phosphate) was added to each well to achieve a target concentration of 1 mM. The plate was sealed and shaken for 16 hours, then centrifuged at 1000g for phase separation. An aliquot of supernatant was transferred to a new plate, centrifuged a second time at 100g, and then the final supernatant was further diluted for subsequent analysis. Quantification of solubility was performed using a Sound Analytics LS1 and Sciex 6500+ MS/MS and an 8-point calibration curve.

Permeability (MDCK)

The following procedure was adapted from the literature.[16] To determine the apparent permeability (Papp), MDCK knockout (MDCK-KO) cells were seeded on Transwell 96-well plate inserts (Corning, Tewksbury, MA,) at a density of 1.5 × 10^5 cells/cm^2 in high glucose DMEM with GlutaMAX containing 10%

v/v heat deactivated FBS and 1% v/v penicillin-streptomycin (all from Gibco) and grown for 4 days at 37 °C in an atmosphere of 5% CO_2 and 95% relative humidity. Stock solutions of the compounds were prepared in dimethyl sulfoxide (DMSO) (10 mM), and each compound was dosed in triplicate at a final concentration of 10 µM in Hanks' balanced salt solution (HBSS) at pH 7.4 containing 10 mM 4-(2-hydroxyethyl)-1-piperazineethanesulfonic acid (HEPES) and 0.02% w/v bovine serum albumin (BSA). A solution without the compounds and containing 5% BSA was added to the acceptor. Bafilomycin (100 nM) was added to both compartments. Cells were incubated with the compounds for 2 h at 37 °C, and flux was measured in the apical-to-basolateral direction. Aliquots from the apical, basal and compound dilution plate were sampled and diluted in 90% Methanol 10% Water with 0.1% Formic acid and 0.4 µM glyburide as an analytical standard to precipitate the protein. The solutions as well as the calibration solutions were centrifuged for 30 min at 3000 rpm for protein precipitation. Drug concentrations in the donor and acceptor compartments were measured by liquid chromatography–mass spectrometry (LC-MS/MS).

Log *P*

The Log *P* was measured using a Pion SiriusT3 instrument.

Supplementary Discussion

Stability Experiments

To determine the stability of the bicyclo[2.2.0] system, the following experiments were performed:

<u>Aqueous stability at two different pH:</u>

SI-48 was first saponified to avoid the possibility of acid- or base-mediated hydrolysis:

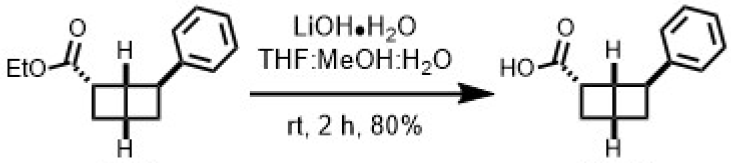

SI-48 **SI-49**

Two solutions of pH = 2 and pH = 10 were prepared with 1M solutions of HCl and NaOH. Then, **SI-49** was stirred in two separate solutions (1 mL of each of the aqueous solution and 0.80 mL of MeCN for each). The stability was tested after 3 hours and after 6 days and the substrate showed complete stability on LC/MS for both durations.

Supplementary Figure 1. ^{1}H NMR spectrum of **SI-4** (500 MHz, $CDCl_3$)

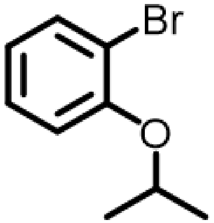

SI-4

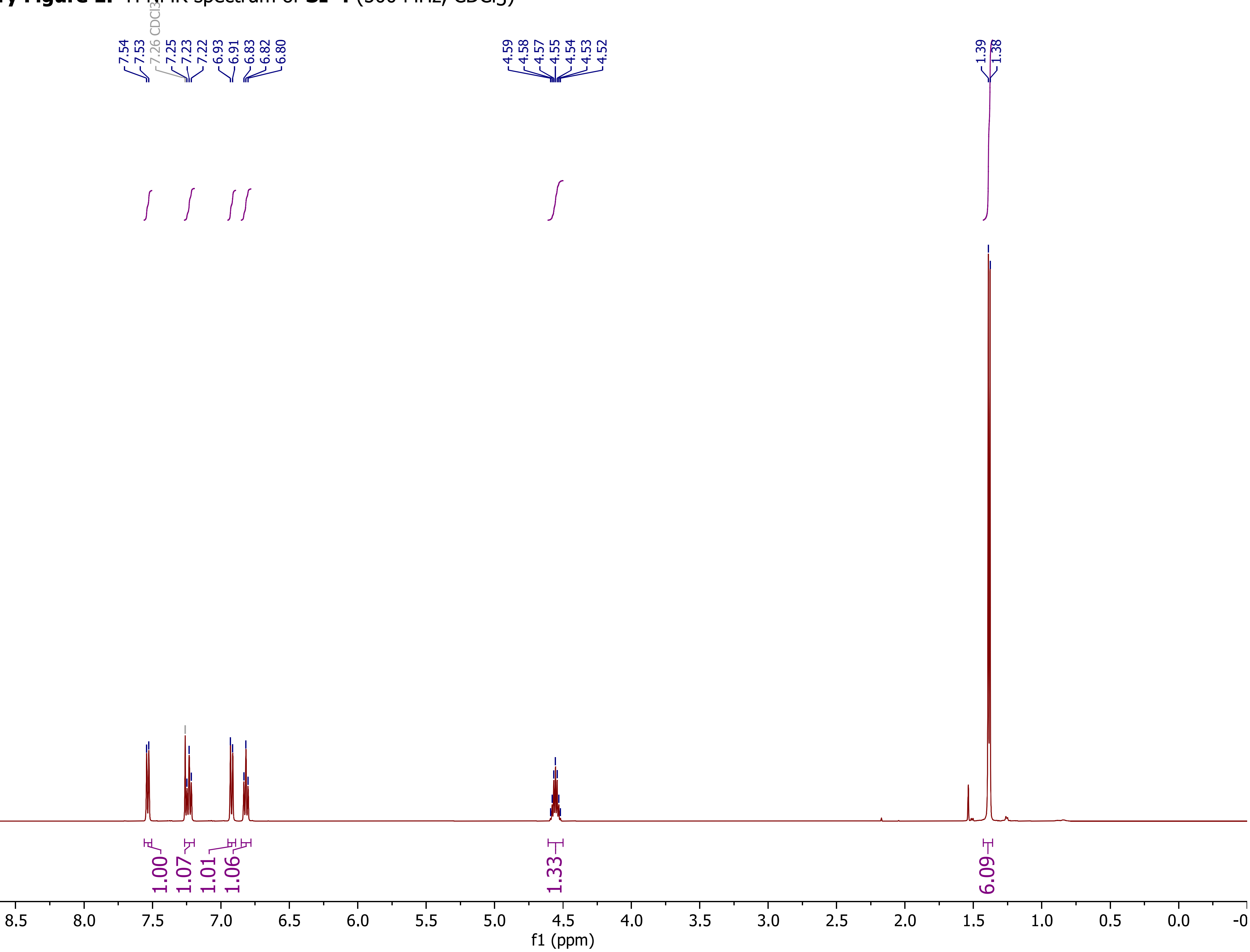

Supplementary Figure 2. ^{13}C NMR spectrum of **SI-4** (126 MHz, $CDCl_3$)

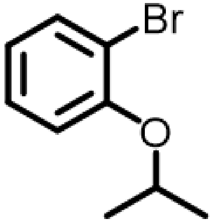

SI-4

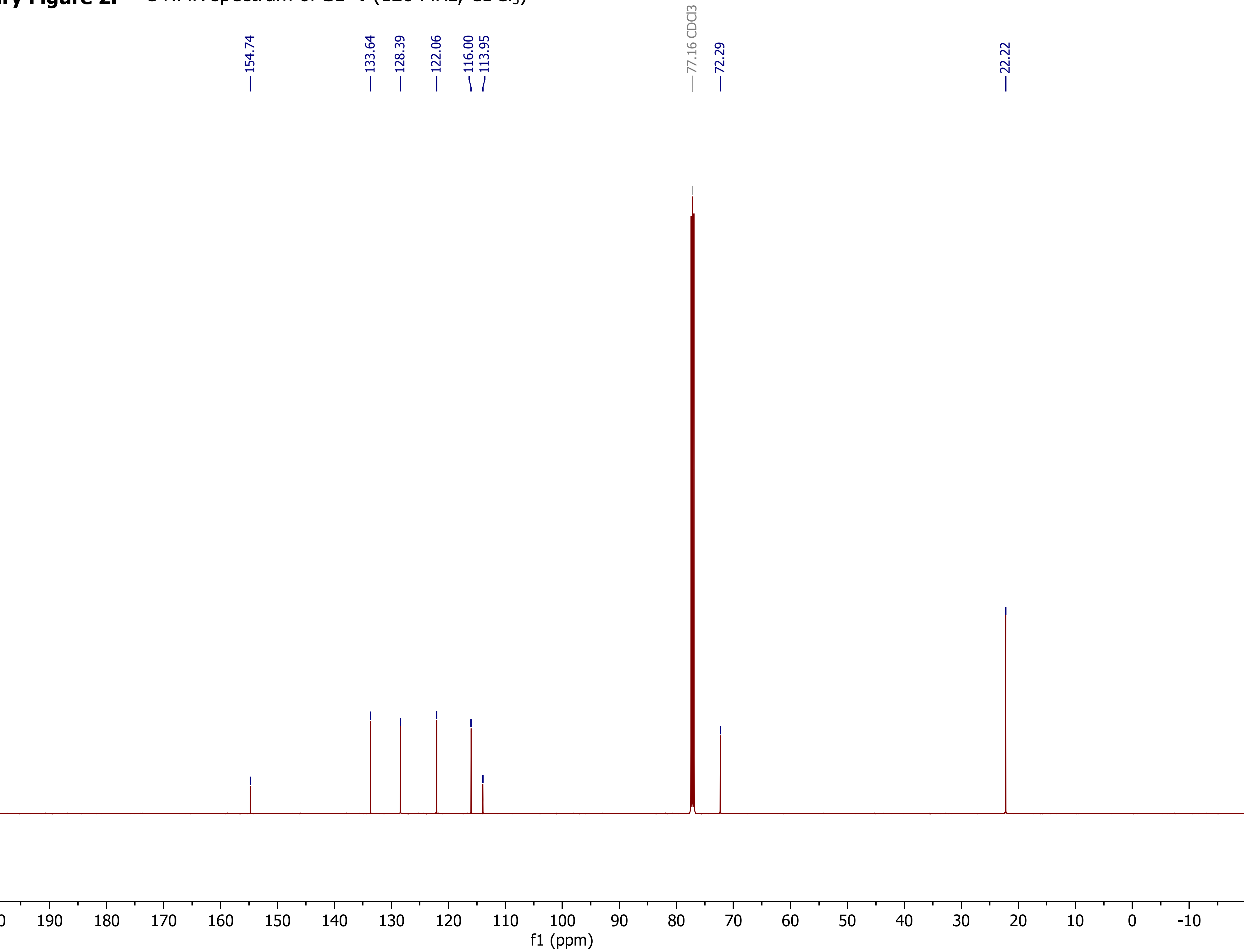

Supplementary Figure 3. ^{1}H NMR spectrum of **SI-5** (500 MHz, $CDCl_3$)

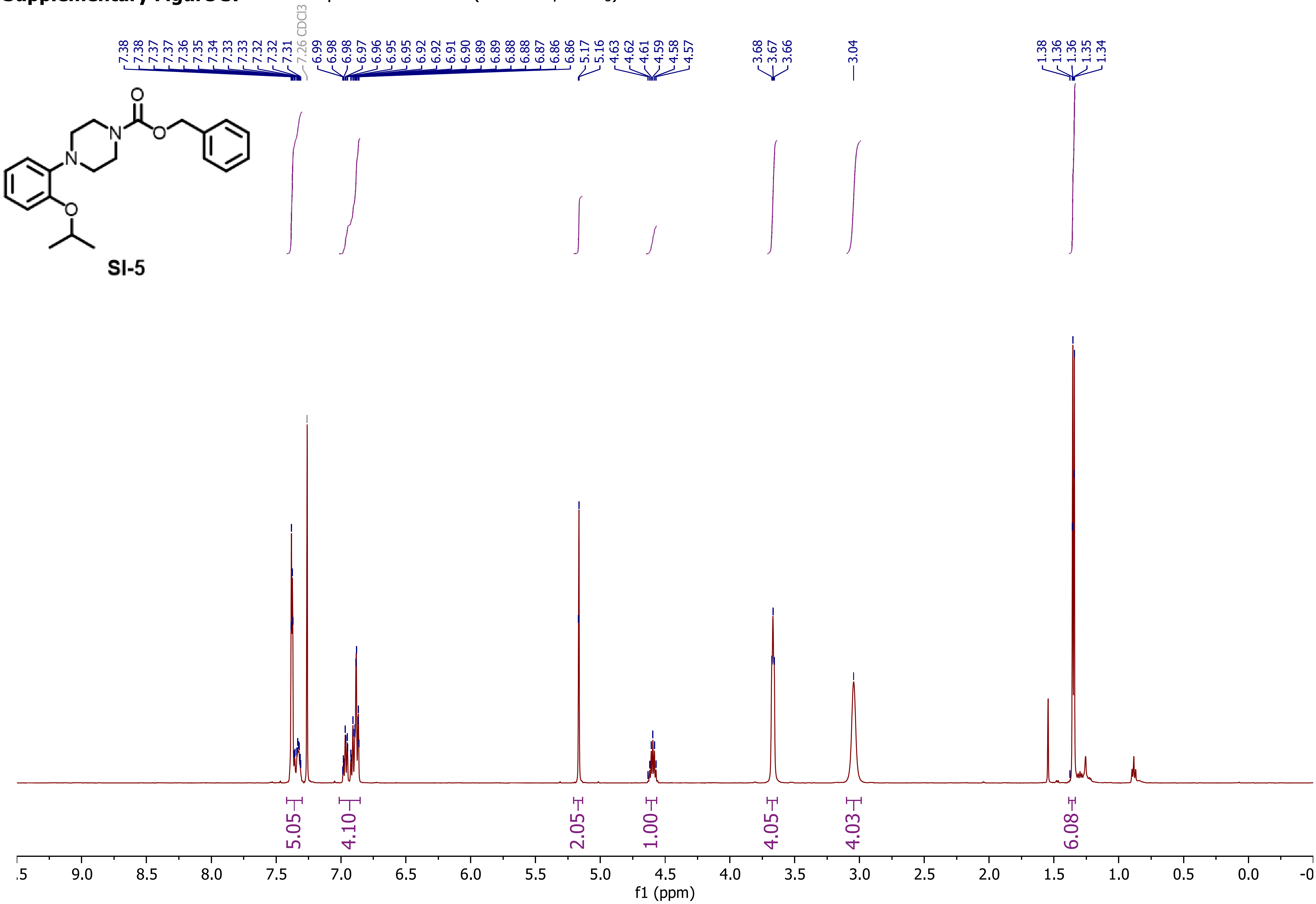

Supplementary Figure 4. ^{13}C NMR spectrum of **SI-5** (126 MHz, $CDCl_3$)

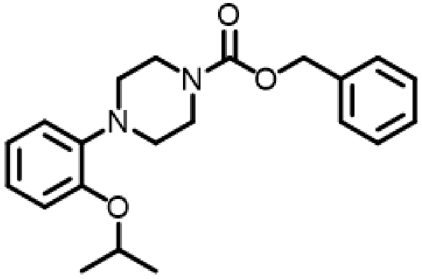

SI-5

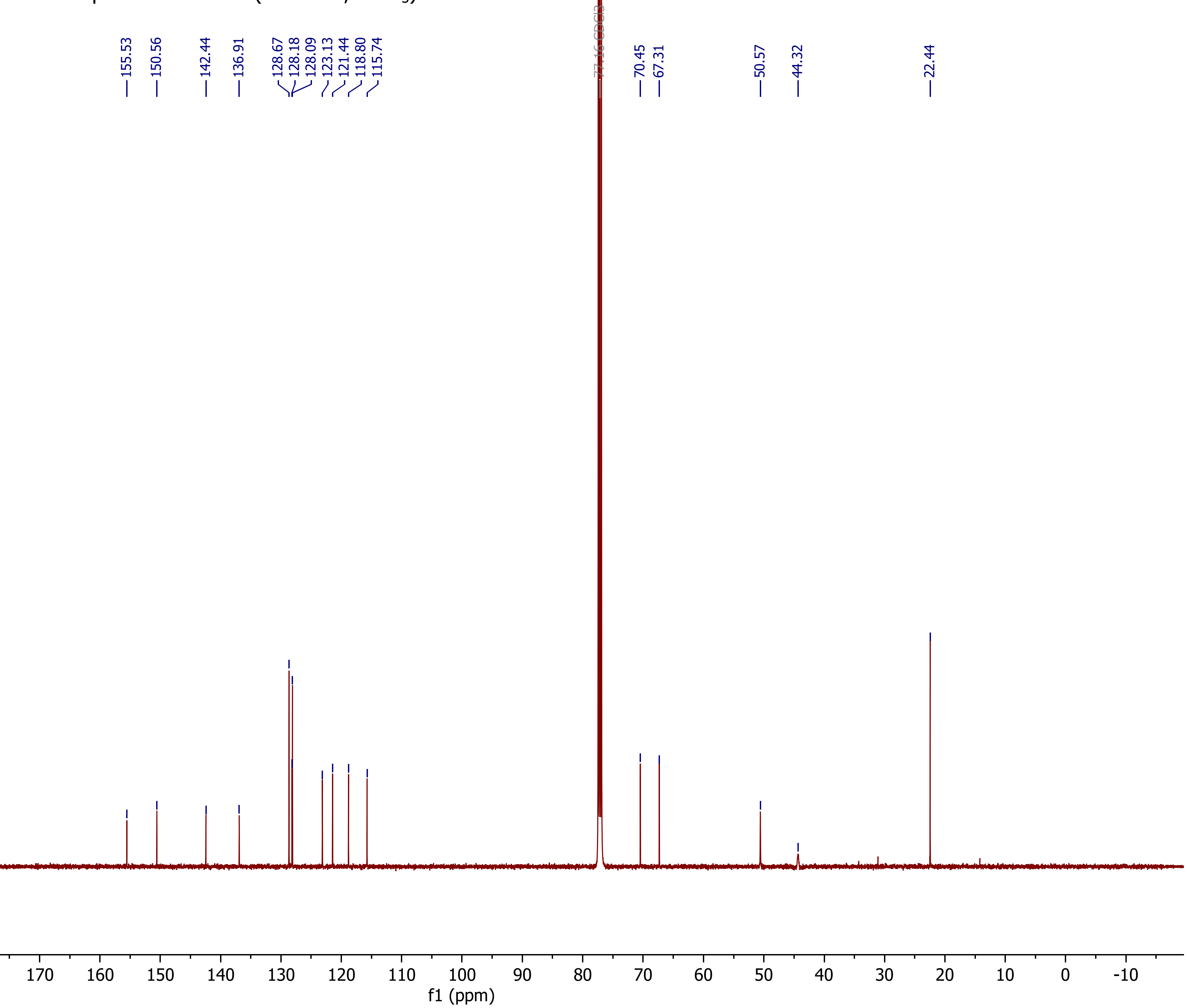

Supplementary Figure 5. ^{1}H NMR spectrum of **SI-6** (500 MHz, $CDCl_3$)

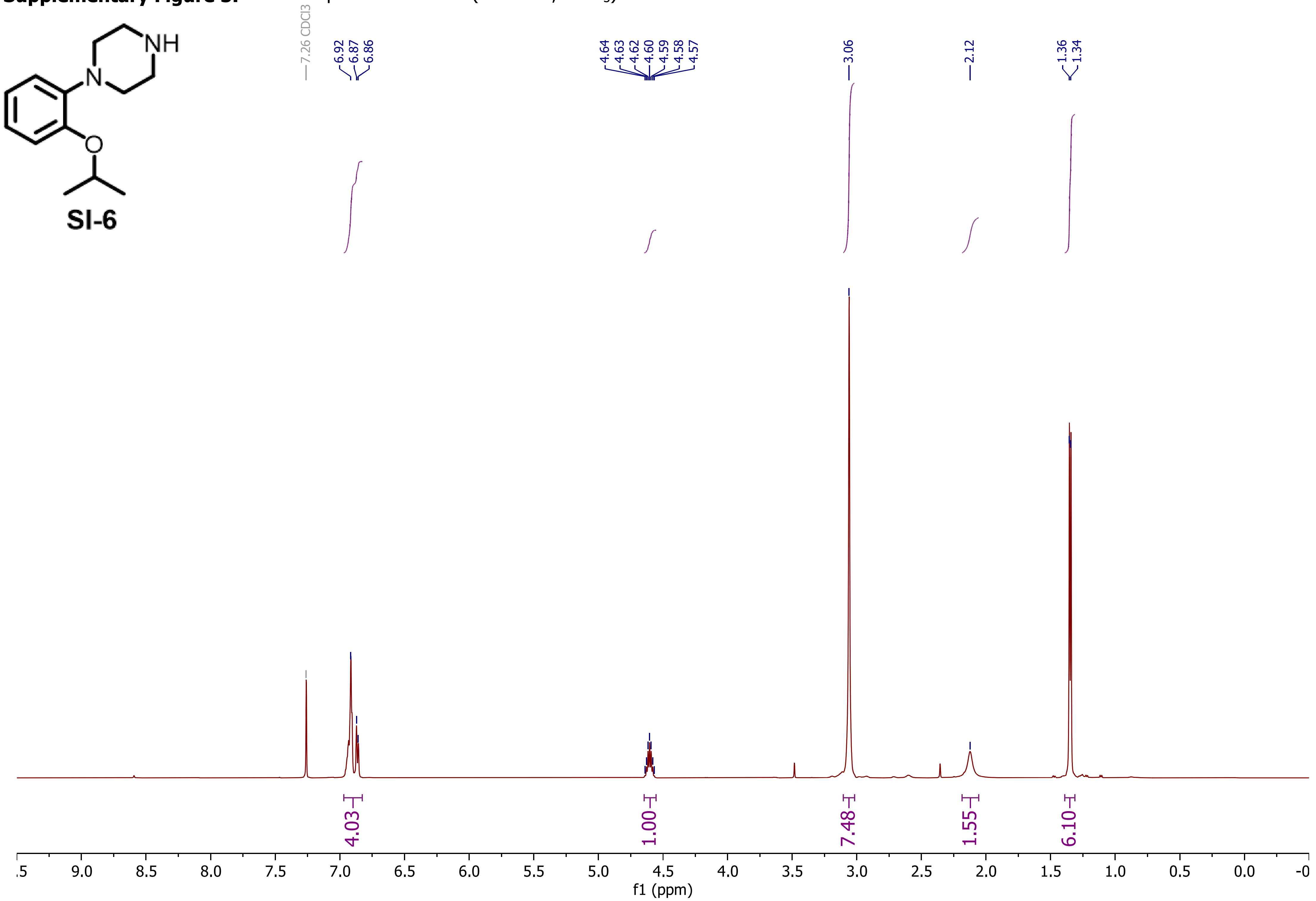

Supplementary Figure 6. ^{13}C NMR spectrum of **SI-6** (126 MHz, $CDCl_3$)

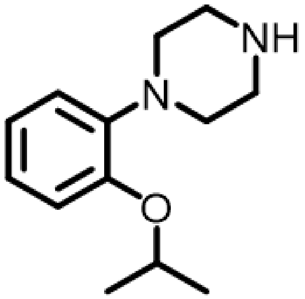

SI-6

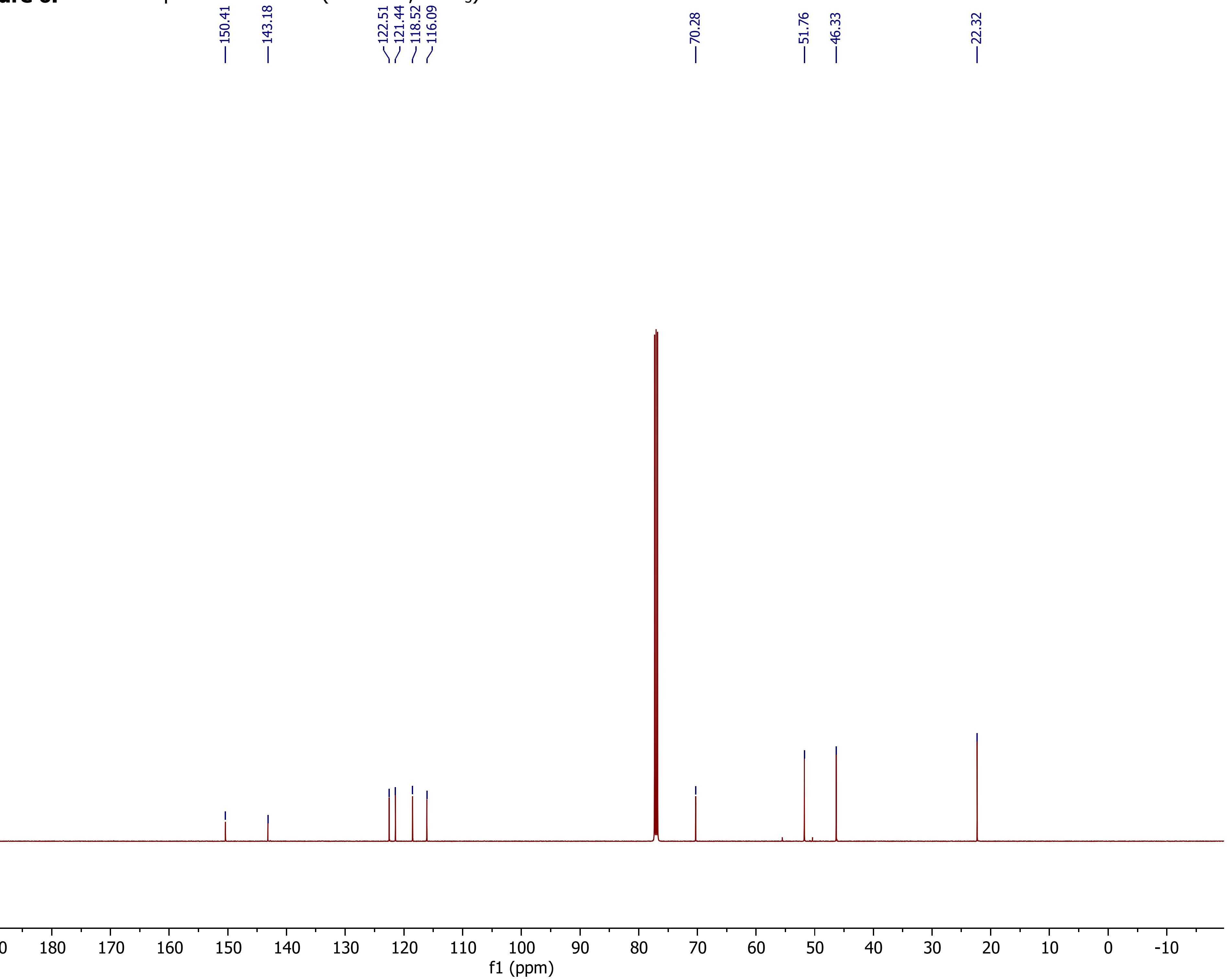

Supplementary Figure 7. ^{1}H NMR spectrum of **SI-7** (500 MHz, $CDCl_3$)

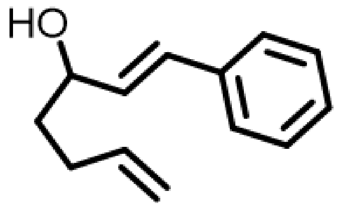

SI-7

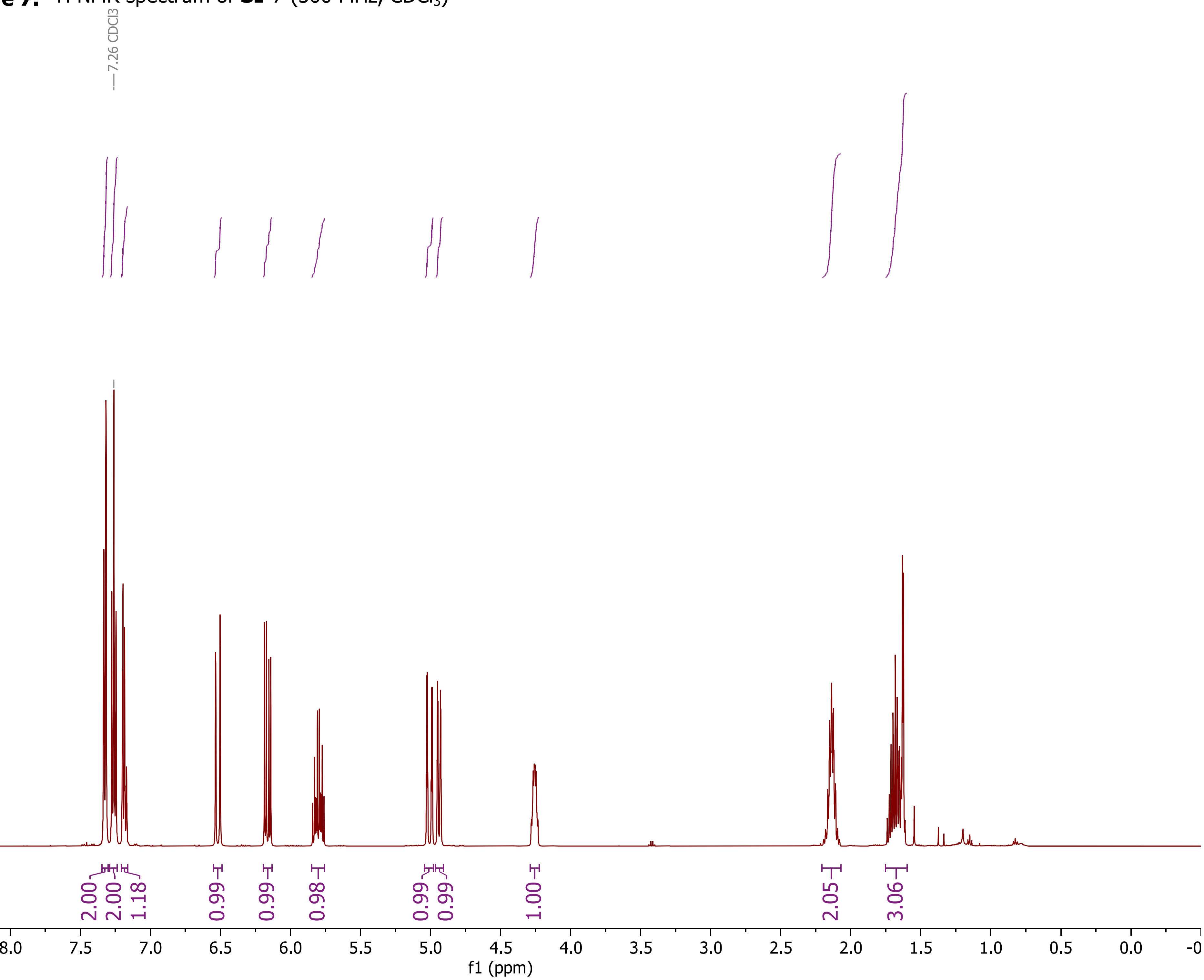

Supplementary Figure 8. ^{13}C NMR spectrum of **SI-7** (126 MHz, $CDCl_3$)

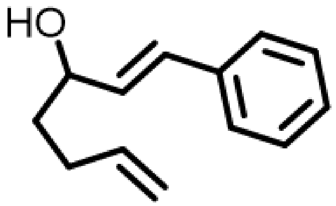

SI-7

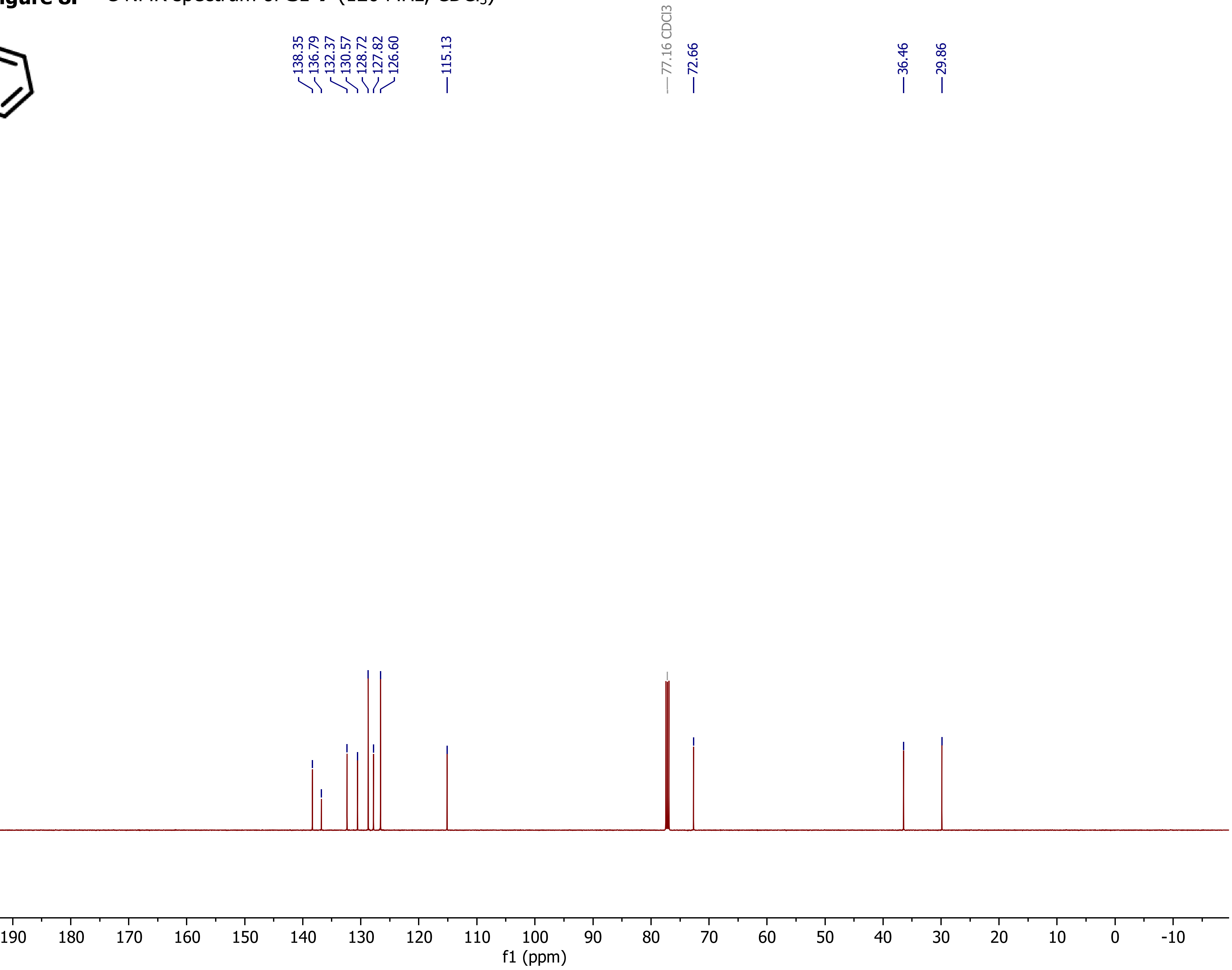

Supplementary Figure 9. ^{1}H NMR spectrum of **SI-8** (400 MHz, $CDCl_3$)

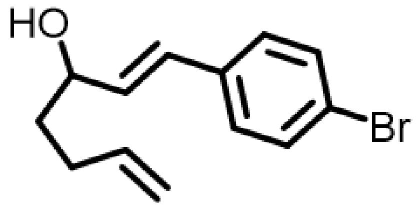

SI-8

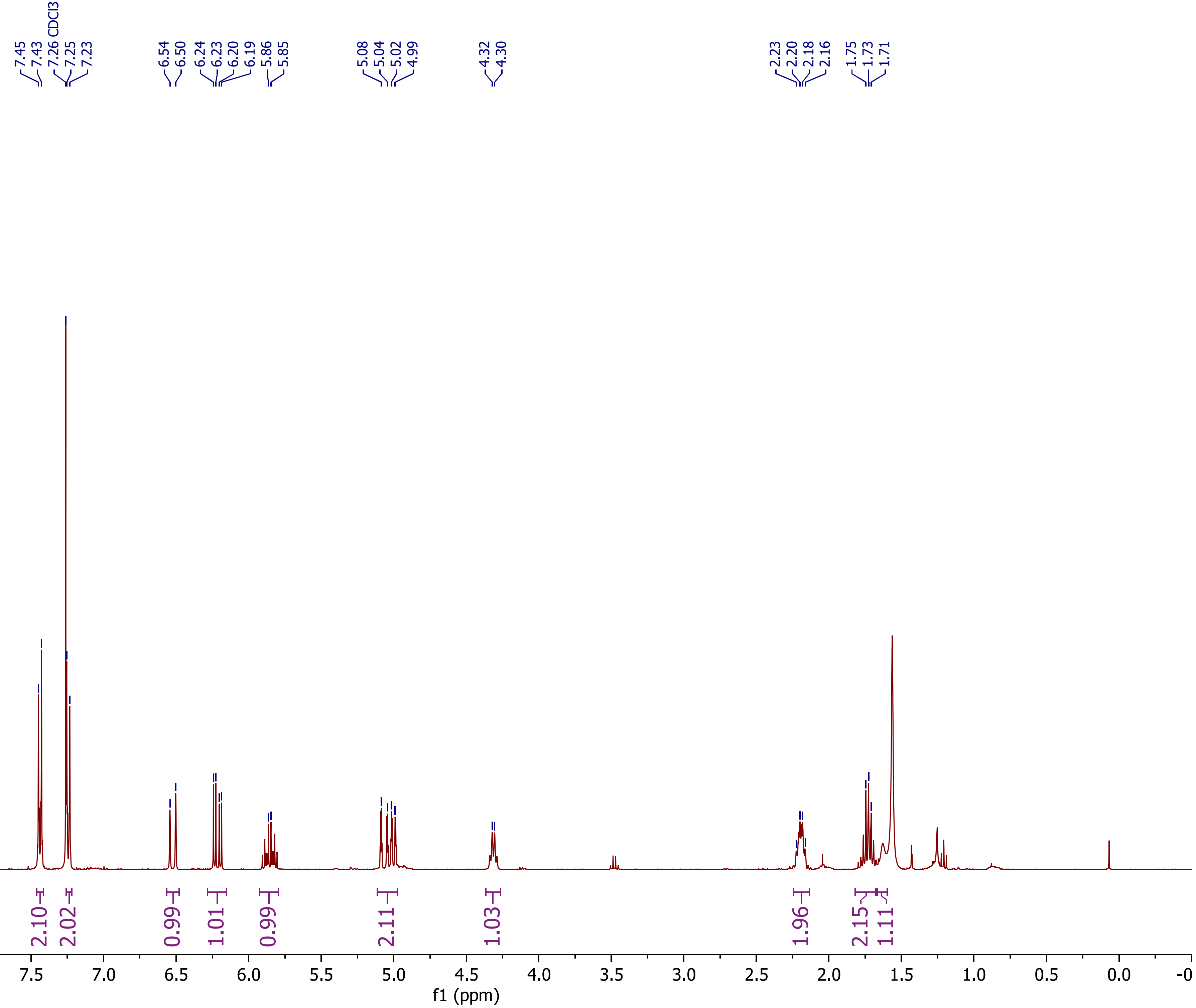

Supplementary Figure 10. ^{13}C NMR spectrum of **SI-8** (101 MHz, $CDCl_3$)

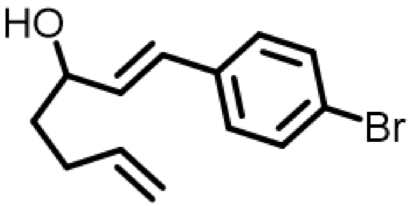

SI-8

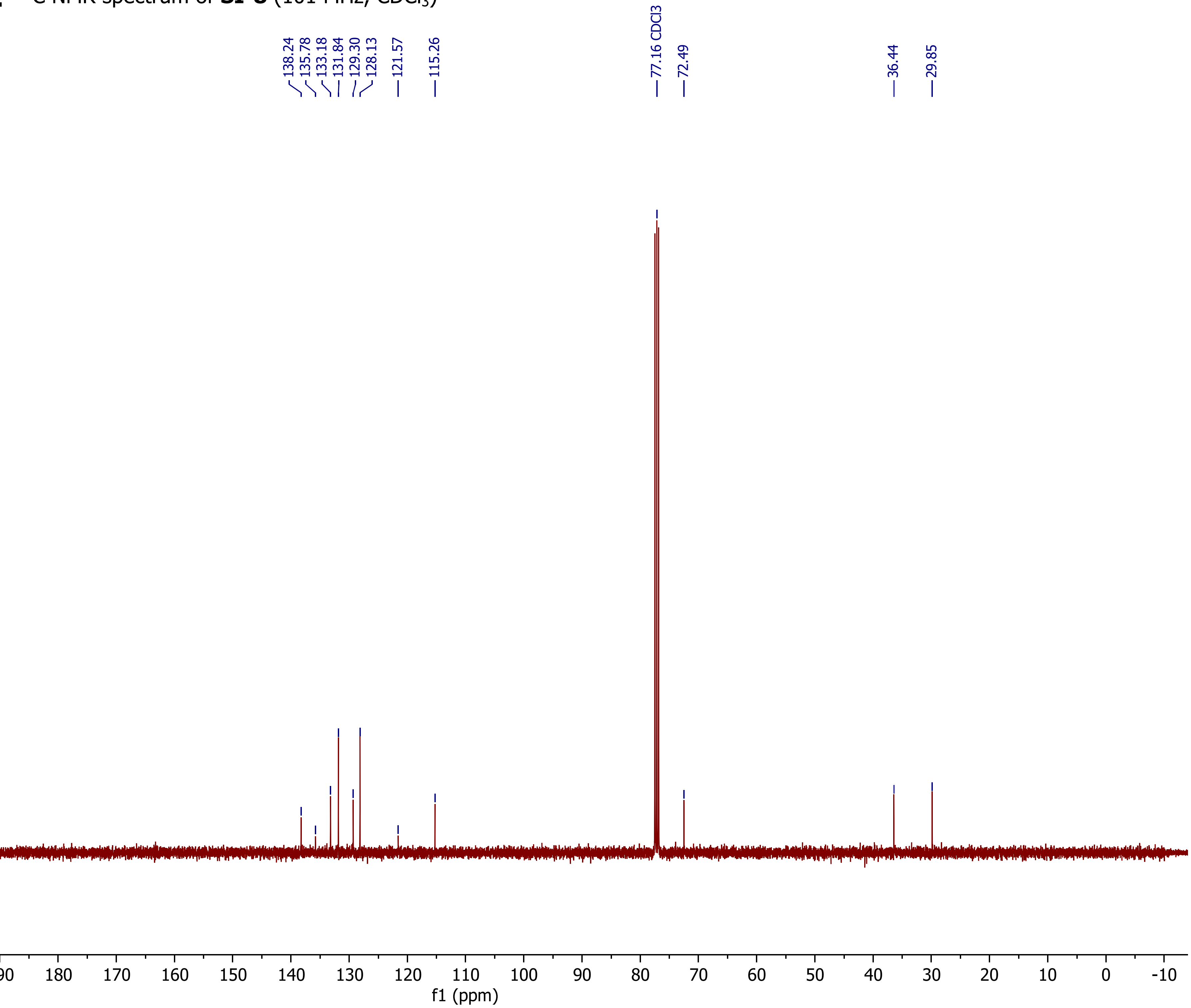

Supplementary Figure 11. ^{1}H NMR spectrum of **SI-9** (400 MHz, $CDCl_3$)

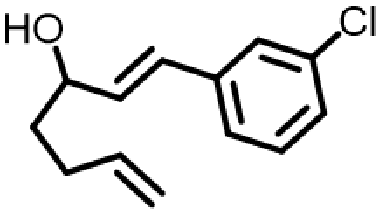

SI-9

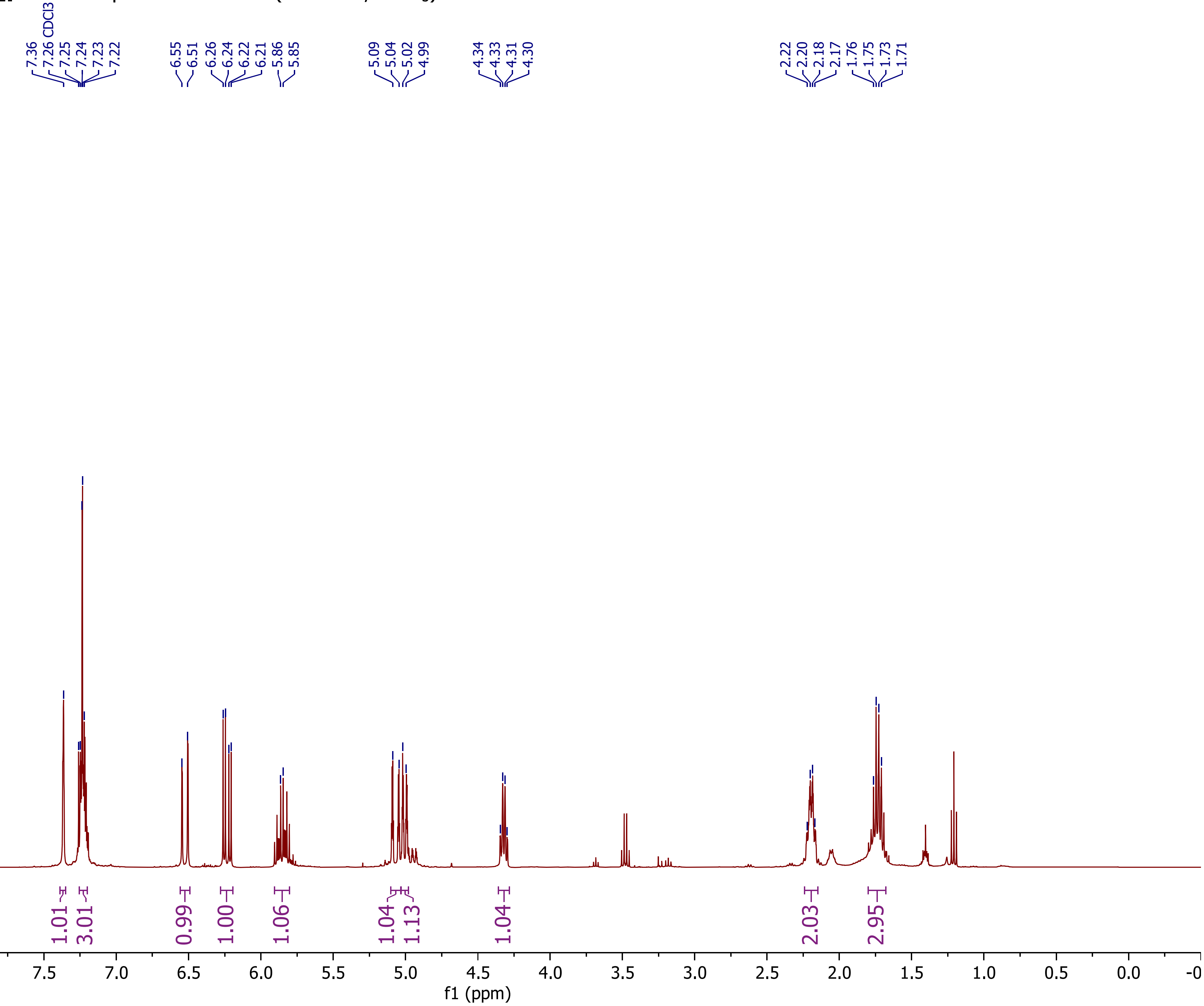

Supplementary Figure 12. ^{1}H NMR spectrum of **SI-10** (400 MHz, $CDCl_3$)

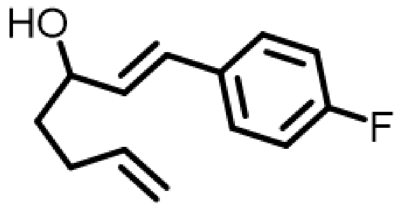

SI-10

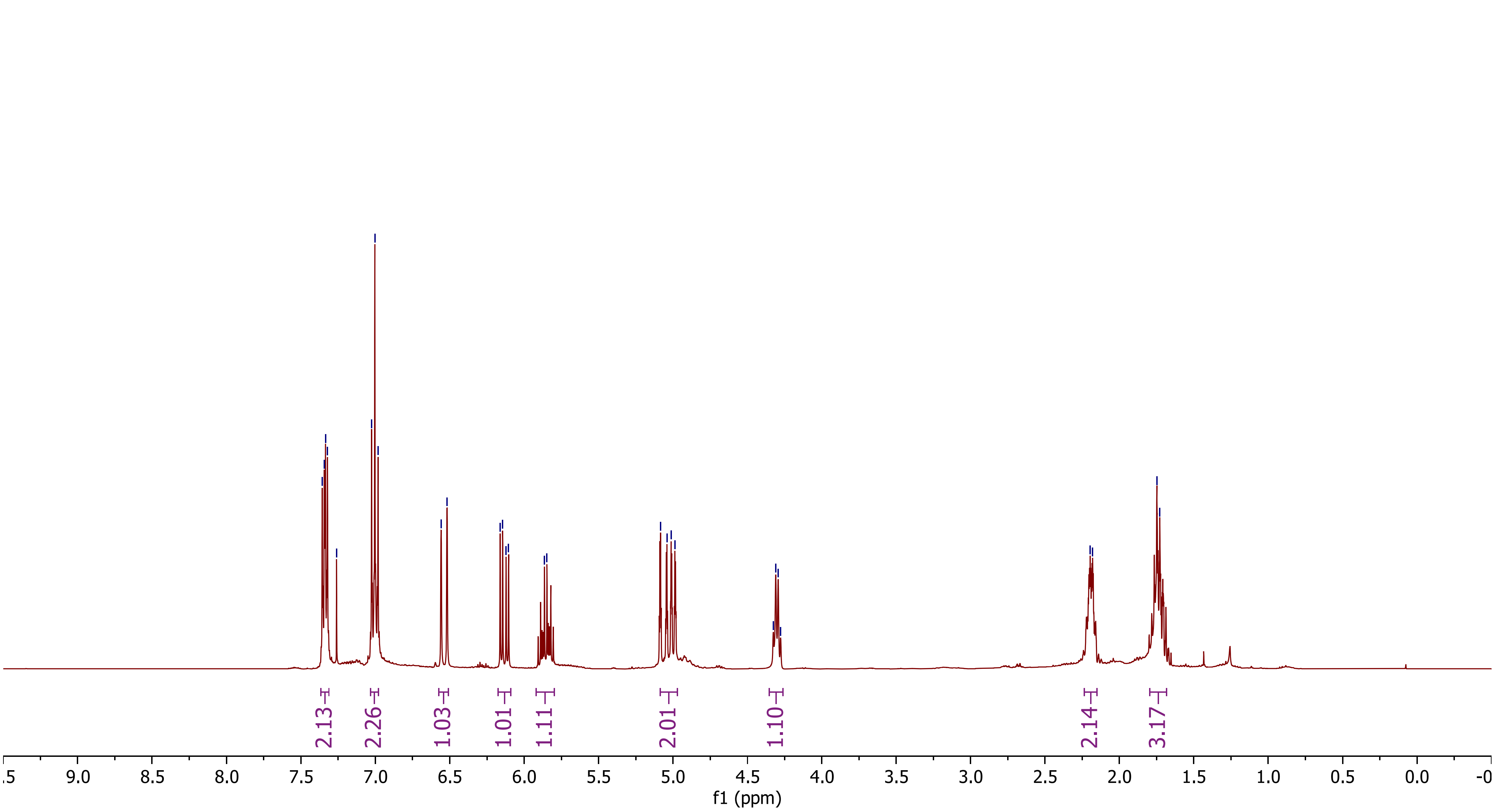

Supplementary Figure 13. ^{13}C NMR spectrum of **SI-10** (101 MHz, $CDCl_3$)

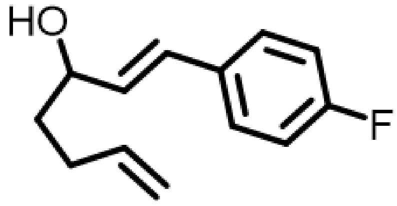

SI-10

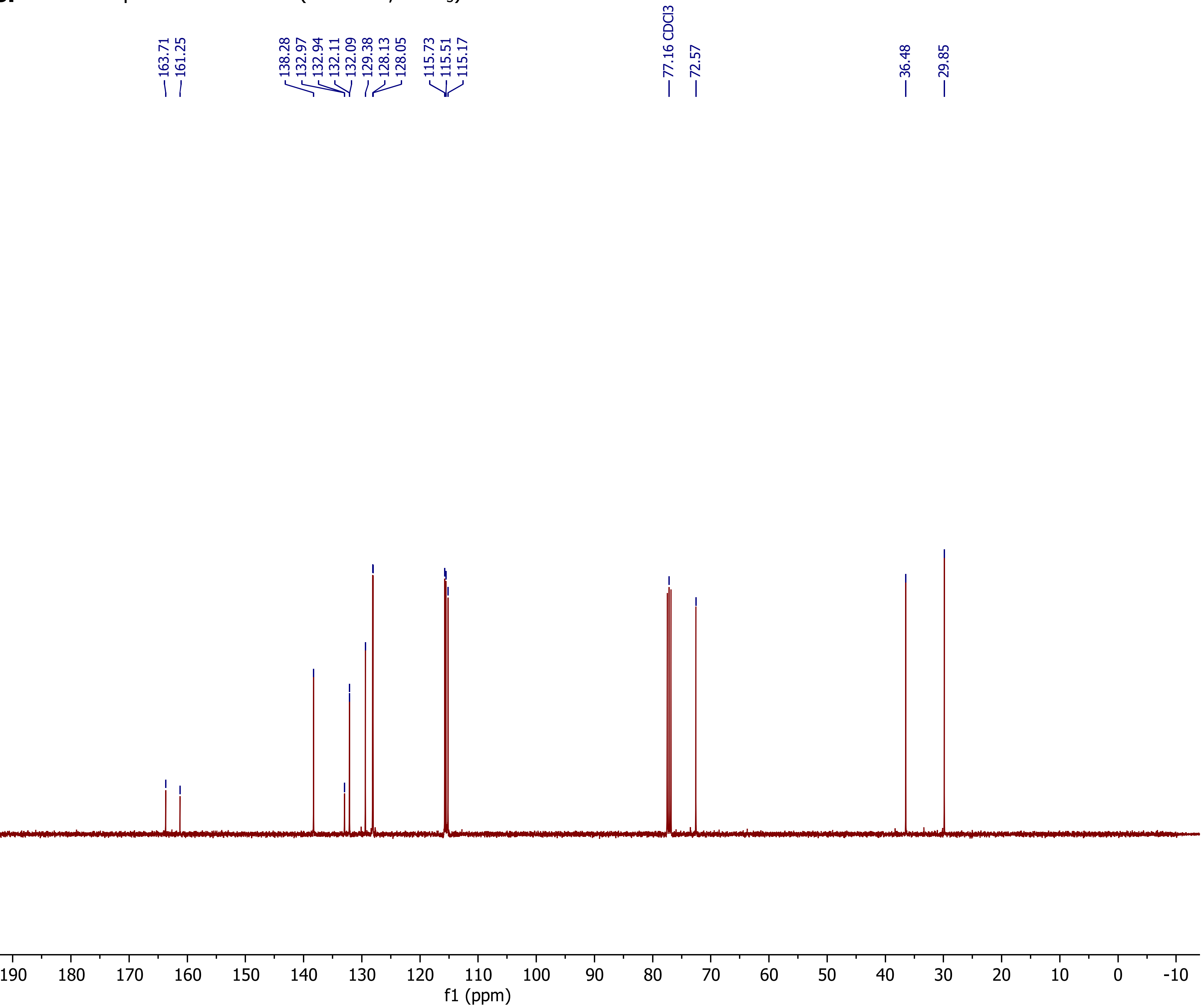

Supplementary Figure 14. ^{1}H NMR spectrum of **SI-11** (400 MHz, $CDCl_3$)

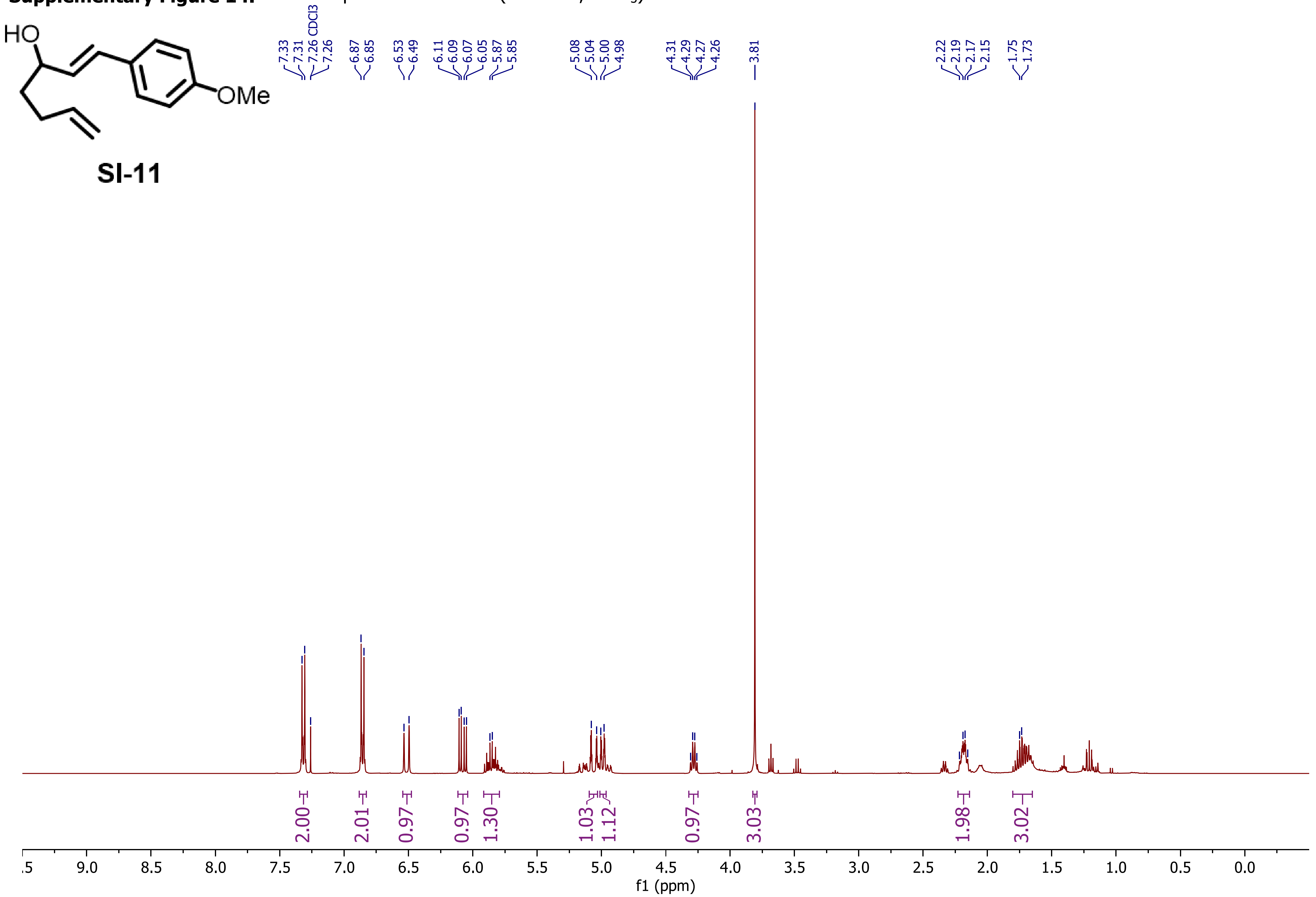

Supplementary Figure 15. ^{1}H NMR spectrum of **SI-12** (400 MHz, $CDCl_3$)

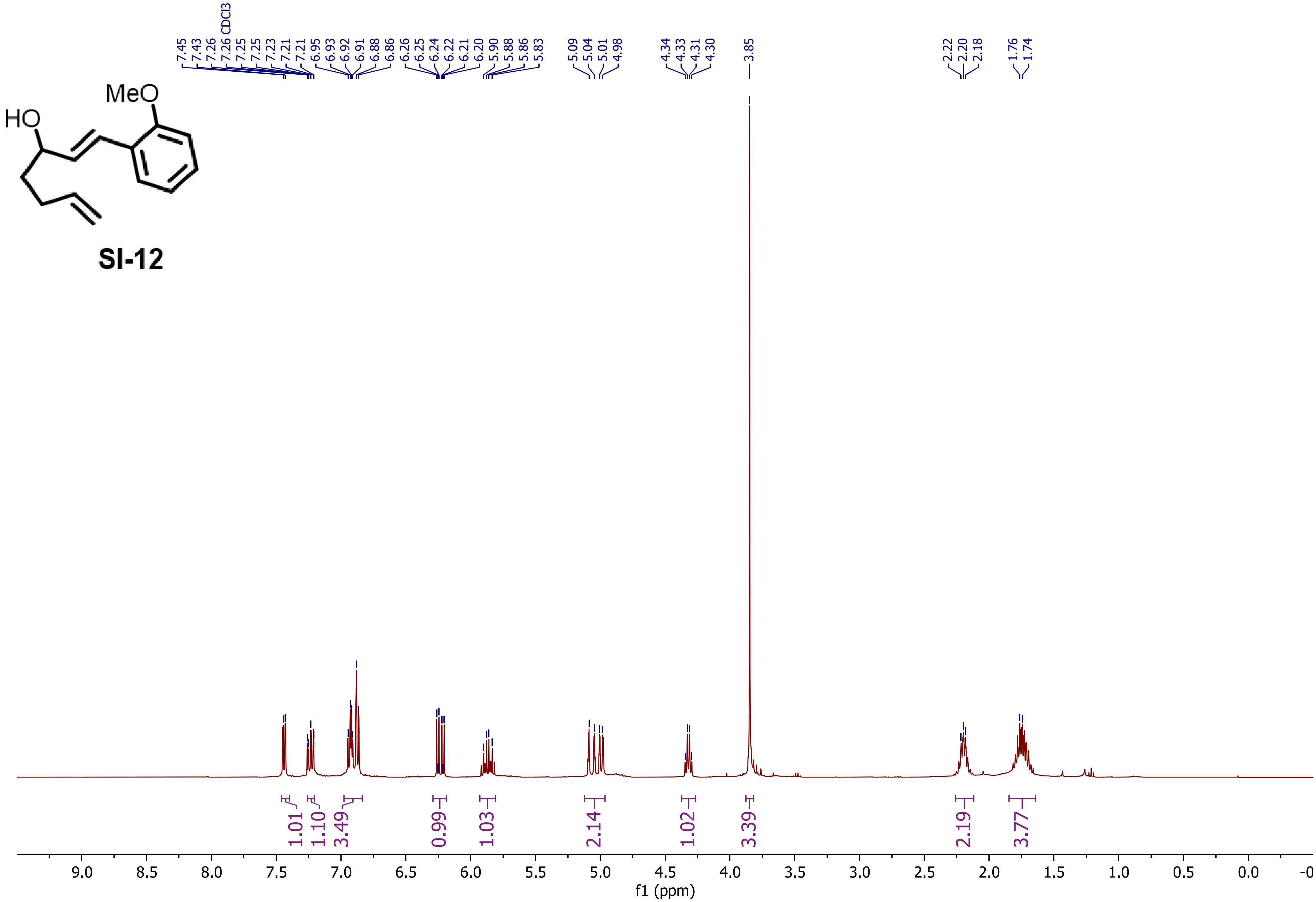

Supplementary Figure 16. ^{13}C NMR spectrum of **SI-12** (101 MHz, $CDCl_3$)

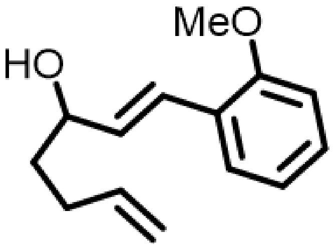

SI-12

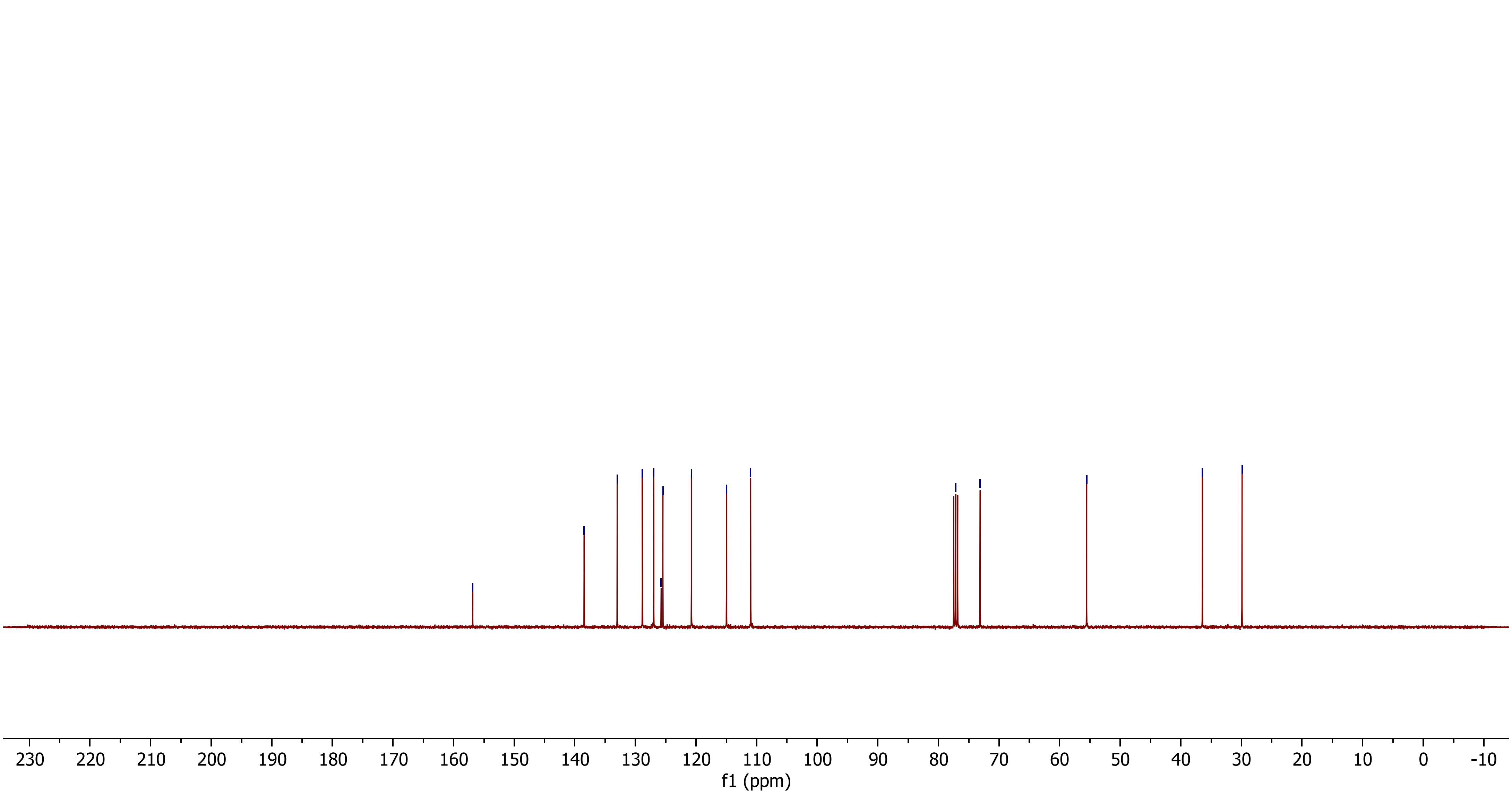

Supplementary Figure 17. ^{1}H NMR spectrum of **SI-13** (400 MHz, $CDCl_3$)

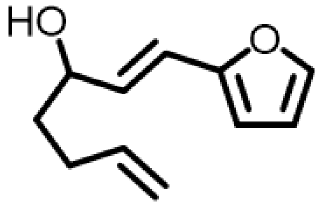

SI-13

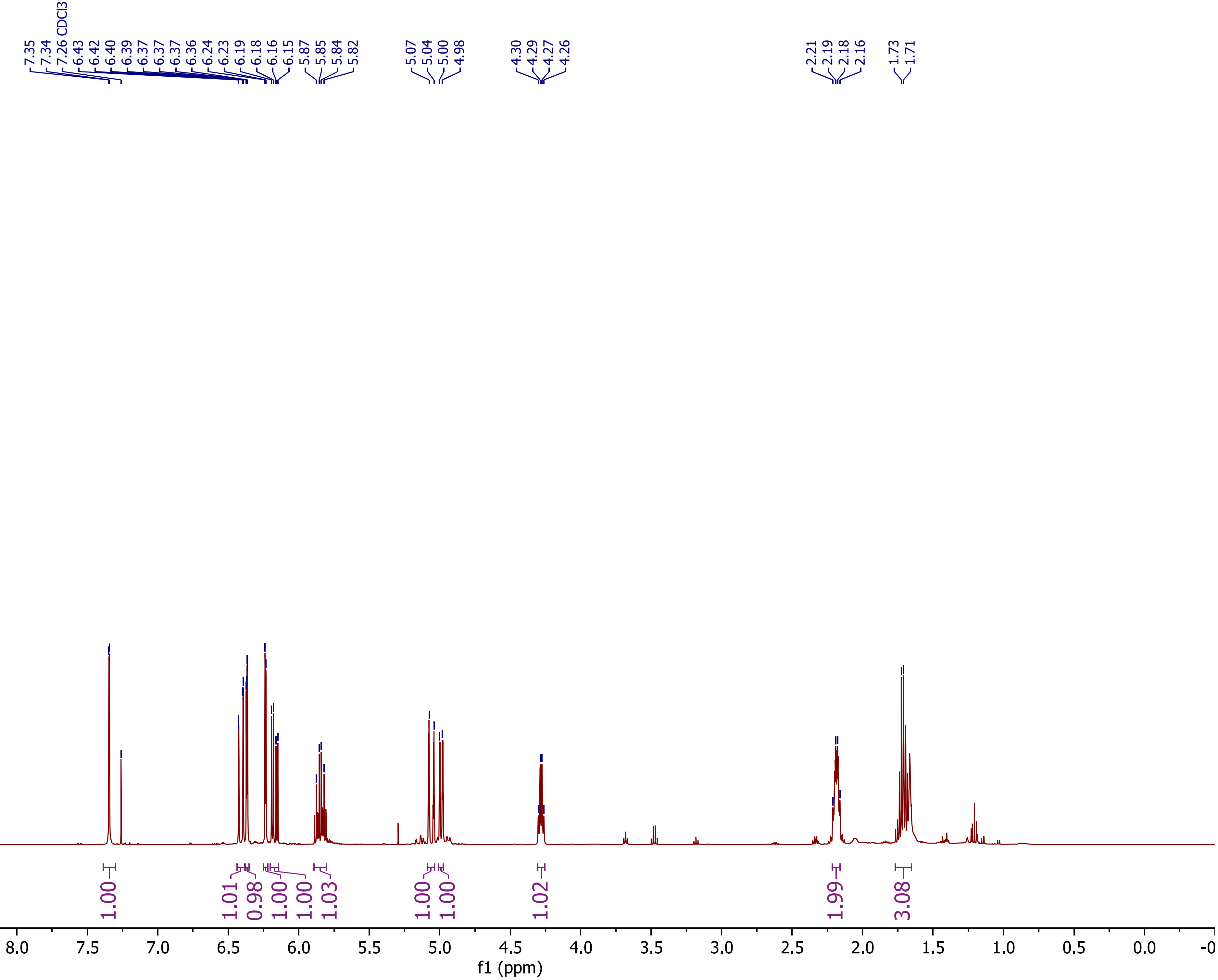

Supplementary Figure 18. ^{13}C NMR spectrum of **SI-13** (101 MHz, $CDCl_3$)

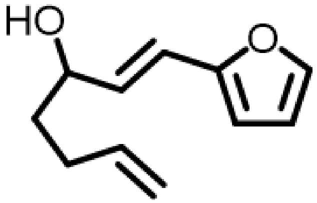

SI-13

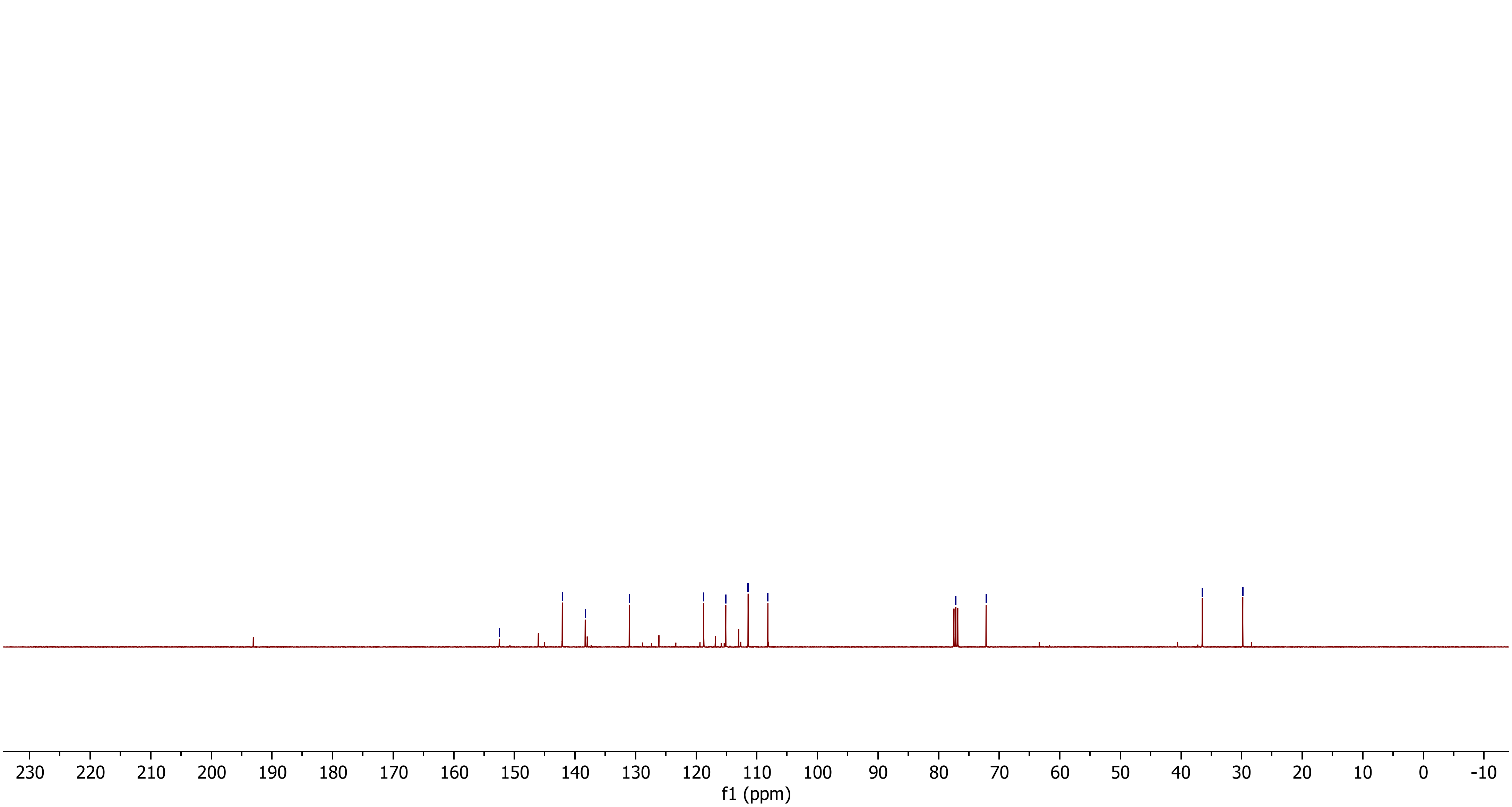

Supplementary Figure 19. ^{1}H NMR spectrum of **SI-14** (500 MHz, $CDCl_3$)

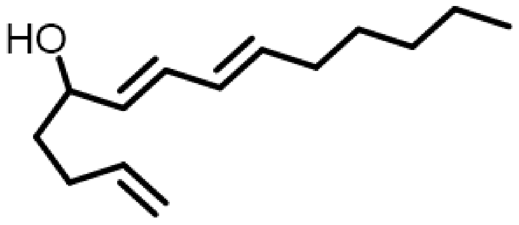

SI-14

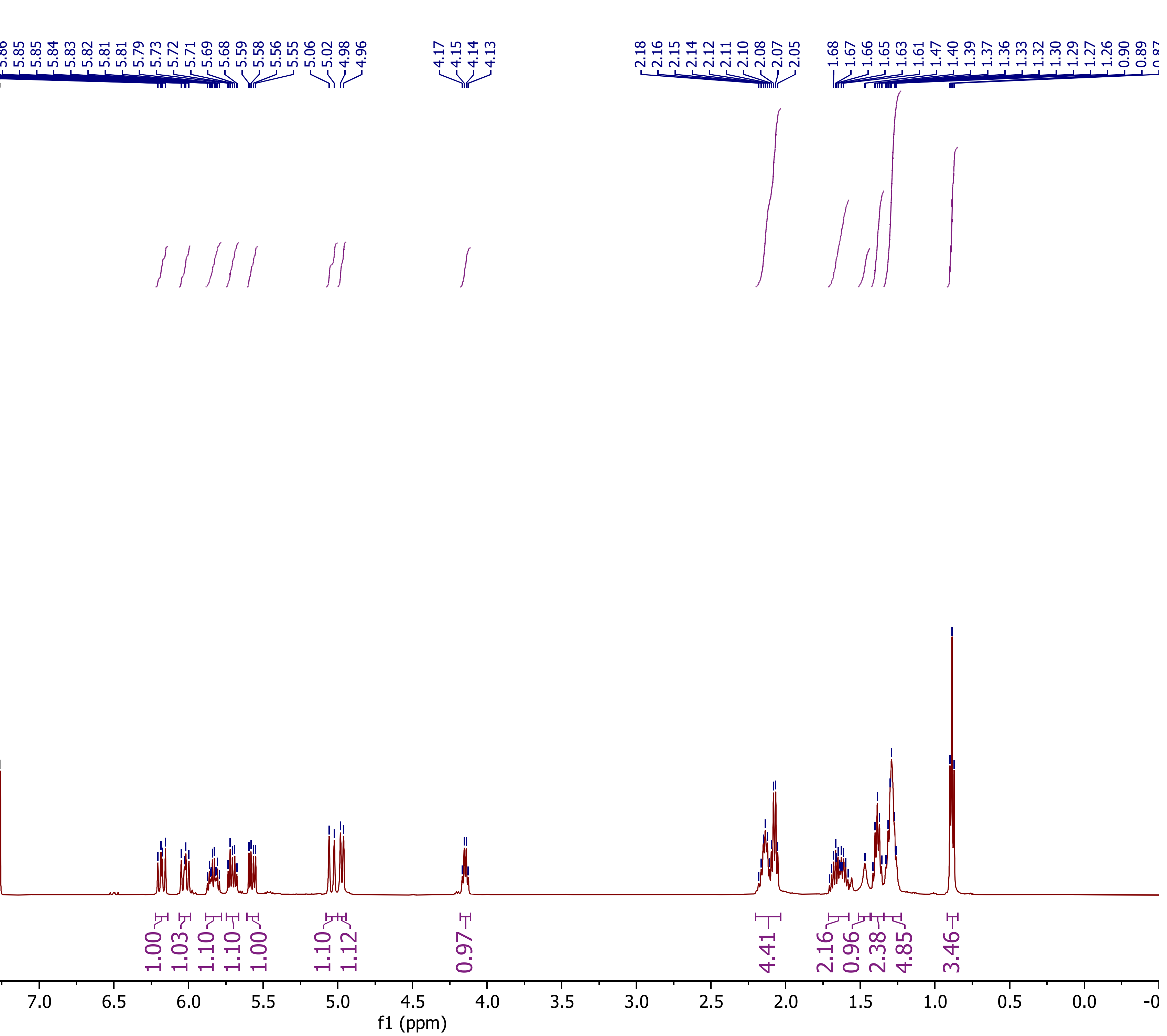

Supplementary Figure 20. ^{13}C NMR spectrum of **SI-14** (126 MHz, $CDCl_3$)

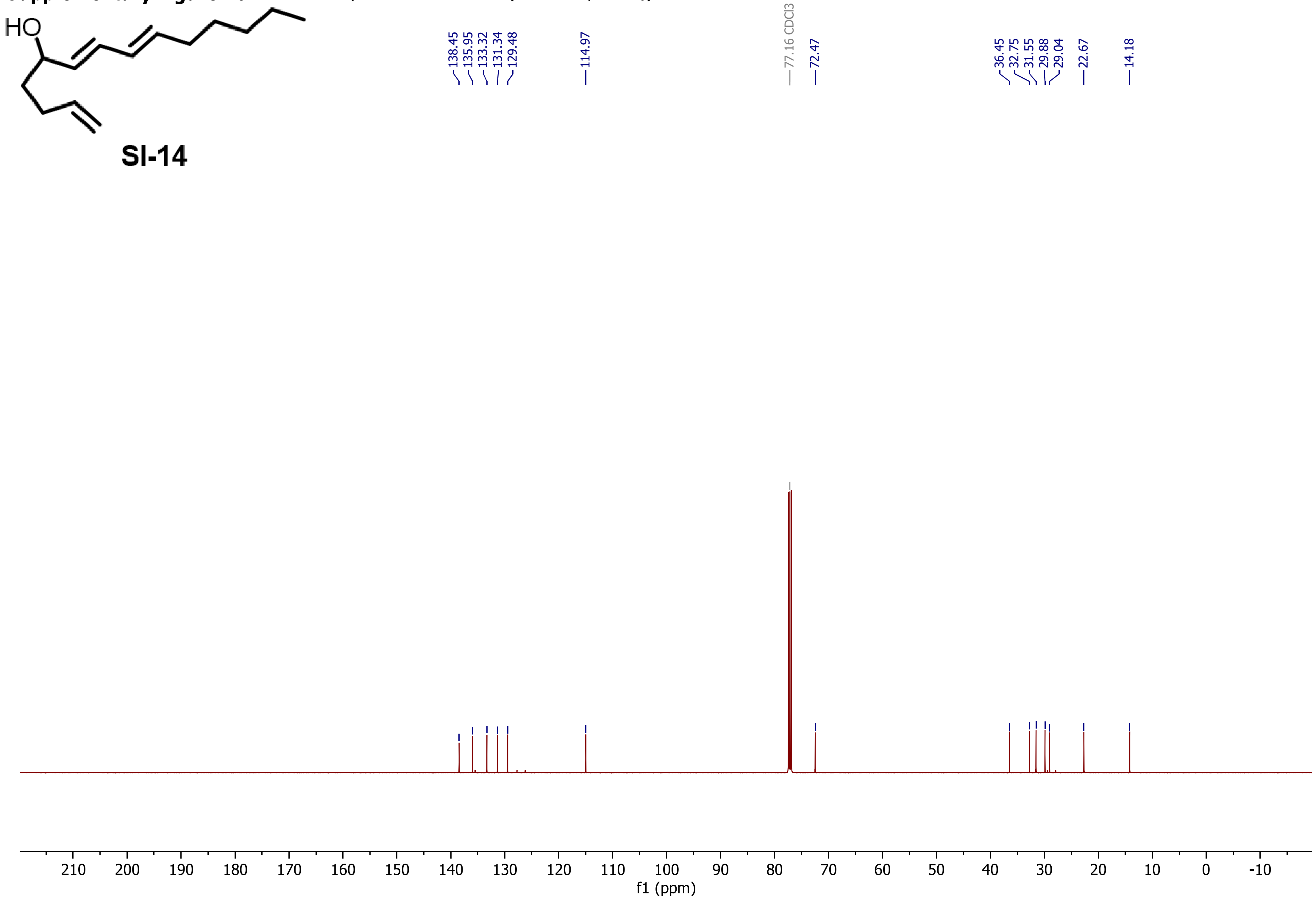

Supplementary Figure 21. ^{1}H NMR spectrum of **SI-15** (500 MHz, $CDCl_3$)

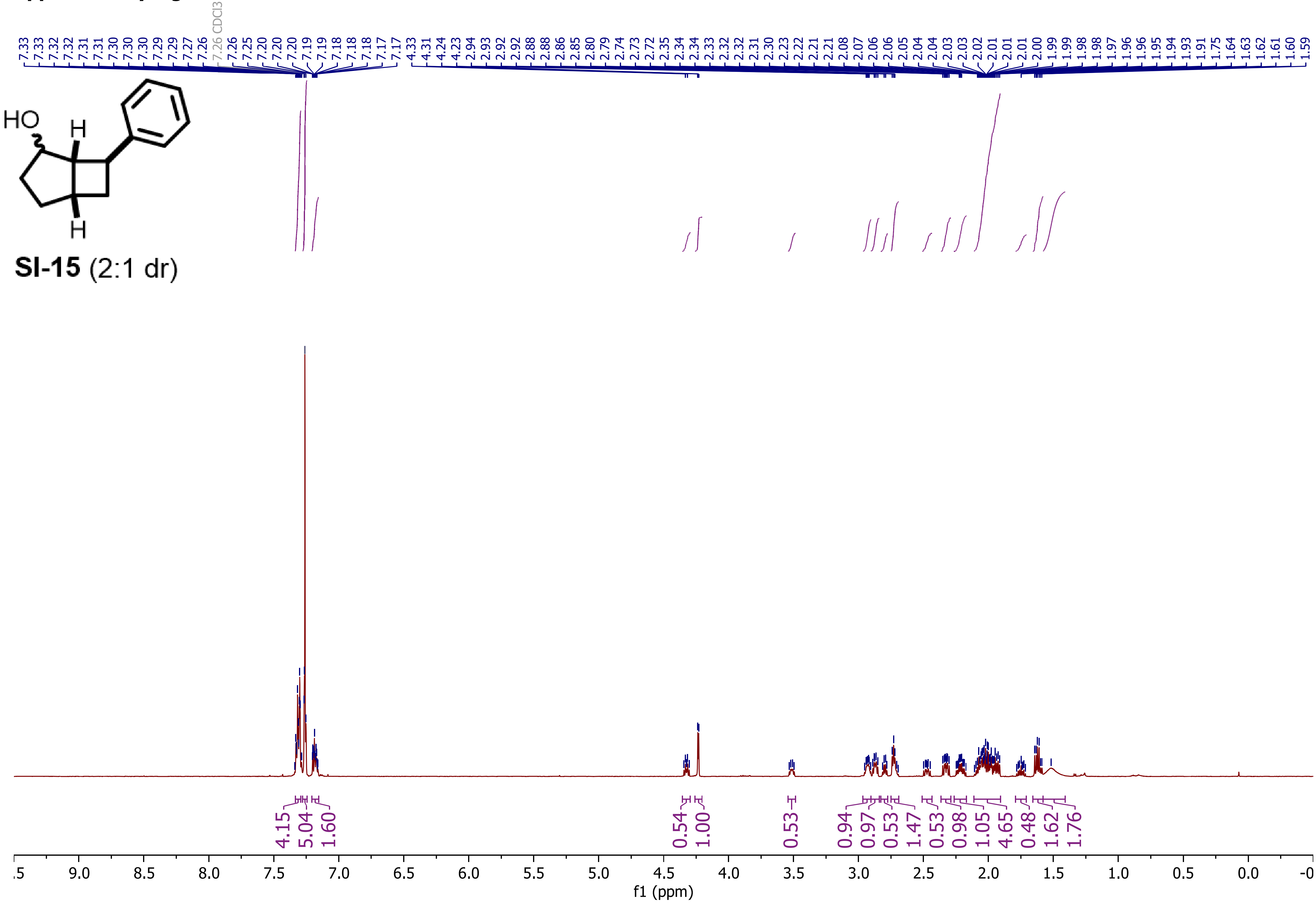

Supplementary Figure 22. ^{13}C NMR spectrum of **SI-15** (126 MHz, $CDCl_3$)

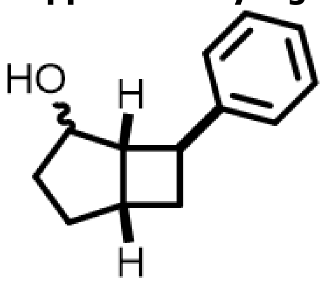

SI-15 (2:1 dr)

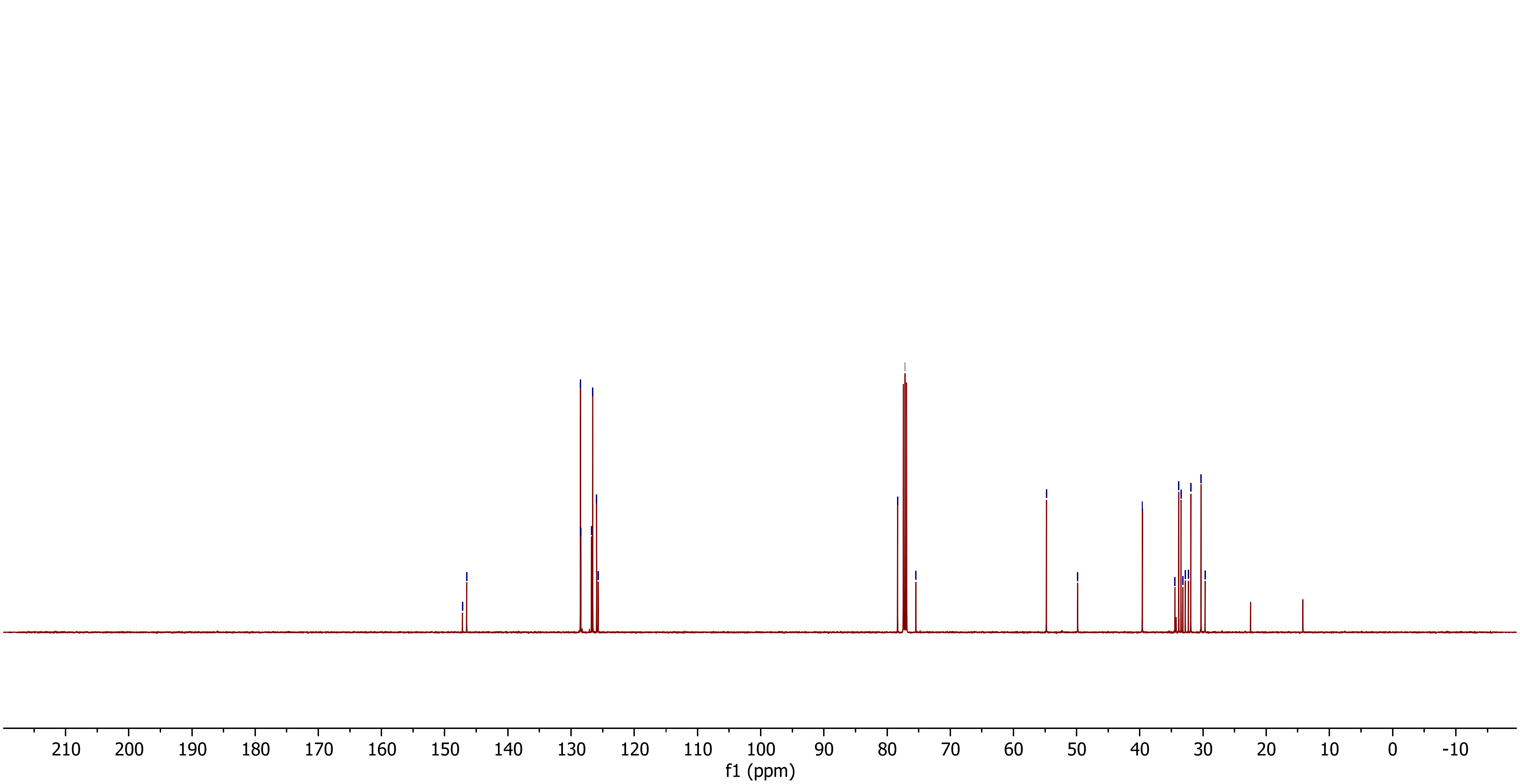

Supplementary Figure 23. ^{1}H NMR spectrum of **SI-16** (400 MHz, $CDCl_3$)

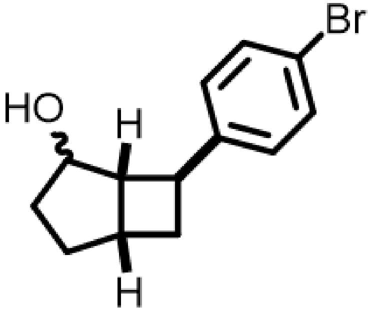

SI-16 (1.5:1 dr)

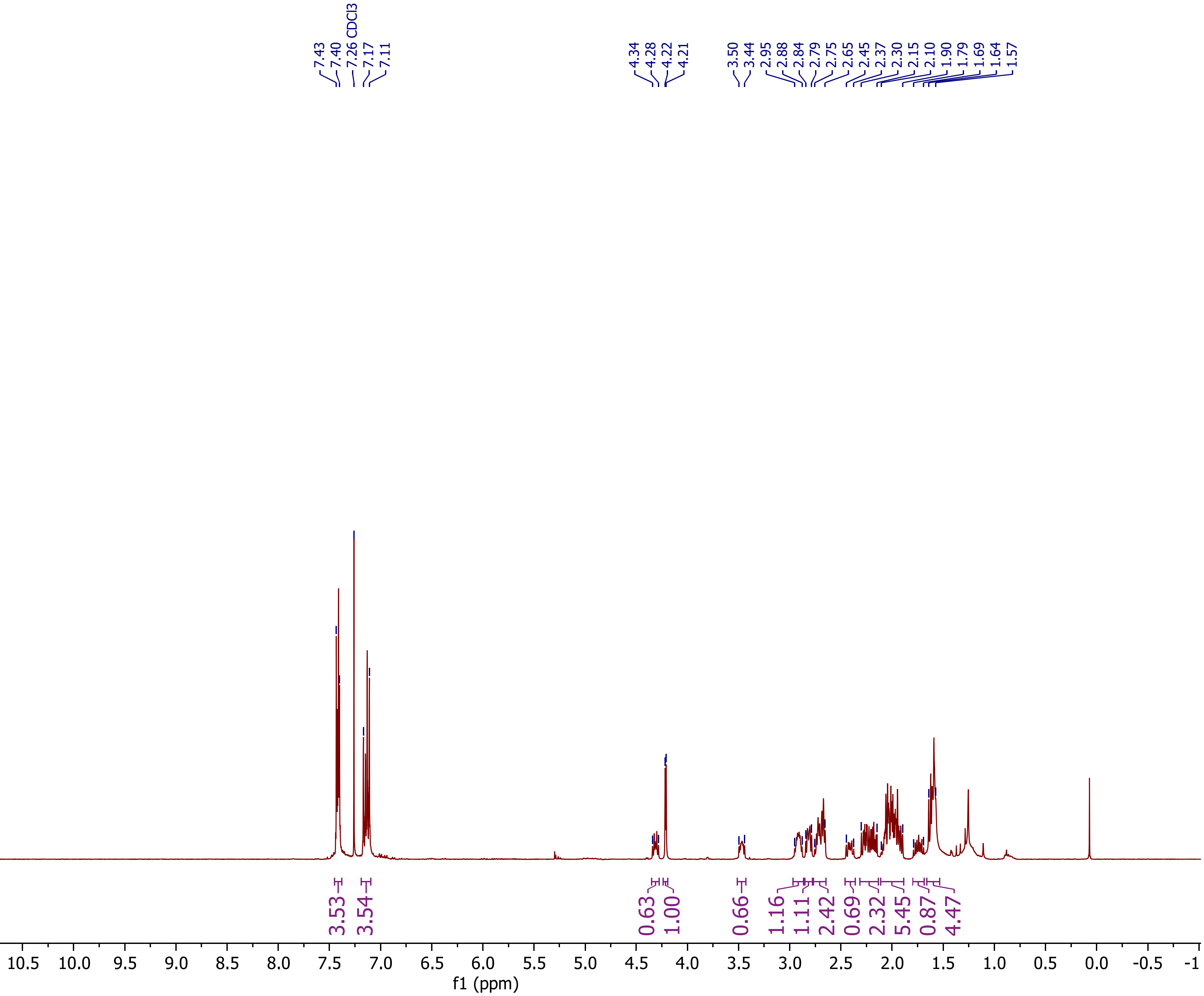

Supplementary Figure 24. ^{13}C NMR spectrum of **SI-16** (101 MHz, $CDCl_3$)

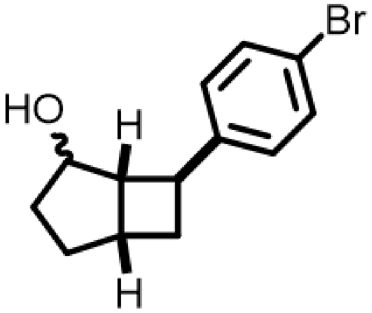

SI-16 (1.5:1 dr)

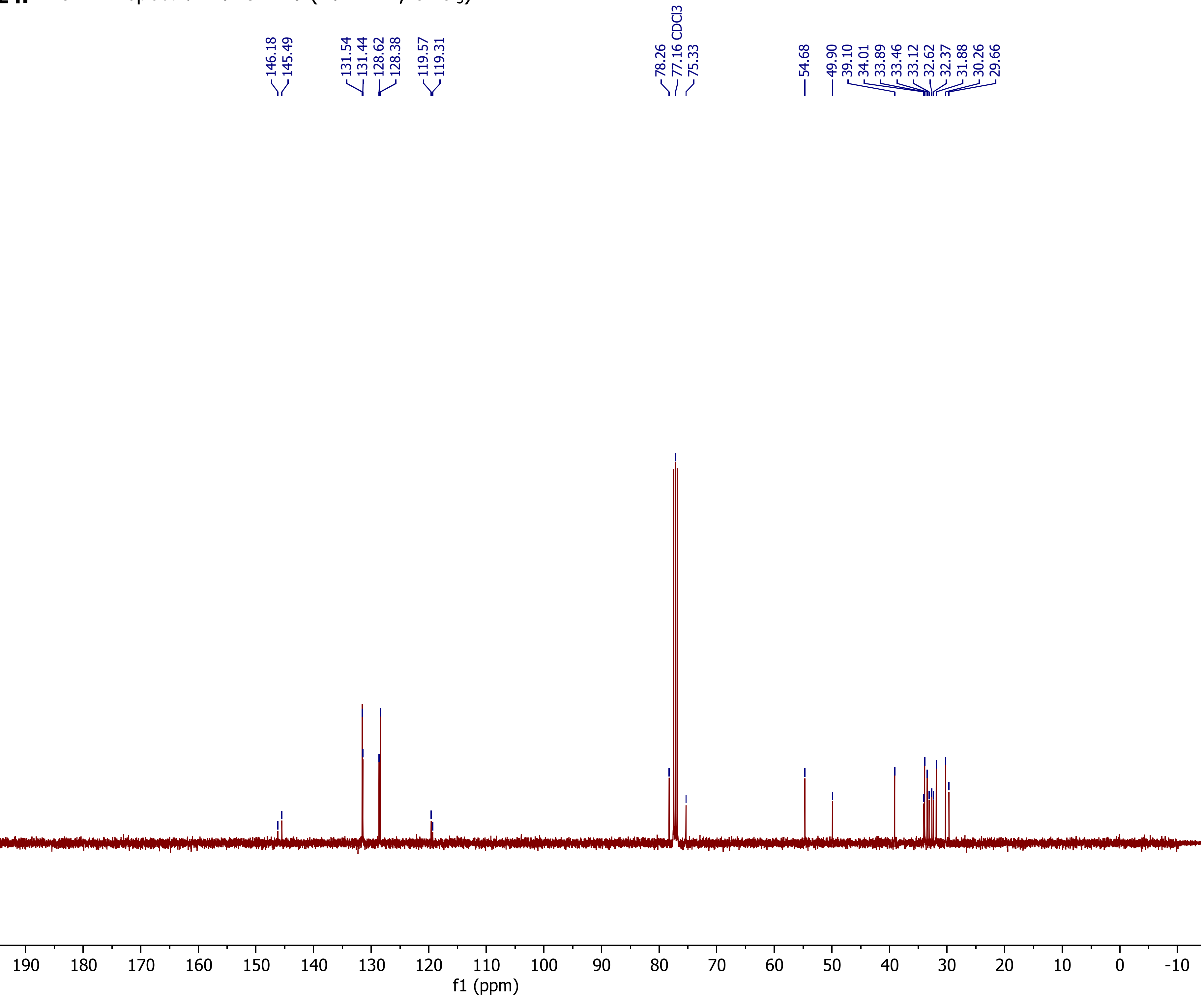

Supplementary Figure 25. ^{1}H NMR spectrum of **SI-17** (400 MHz, $CDCl_3$)

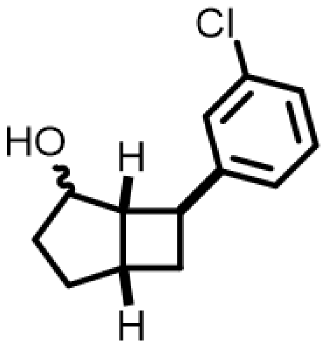

SI-17 (2:1 dr)

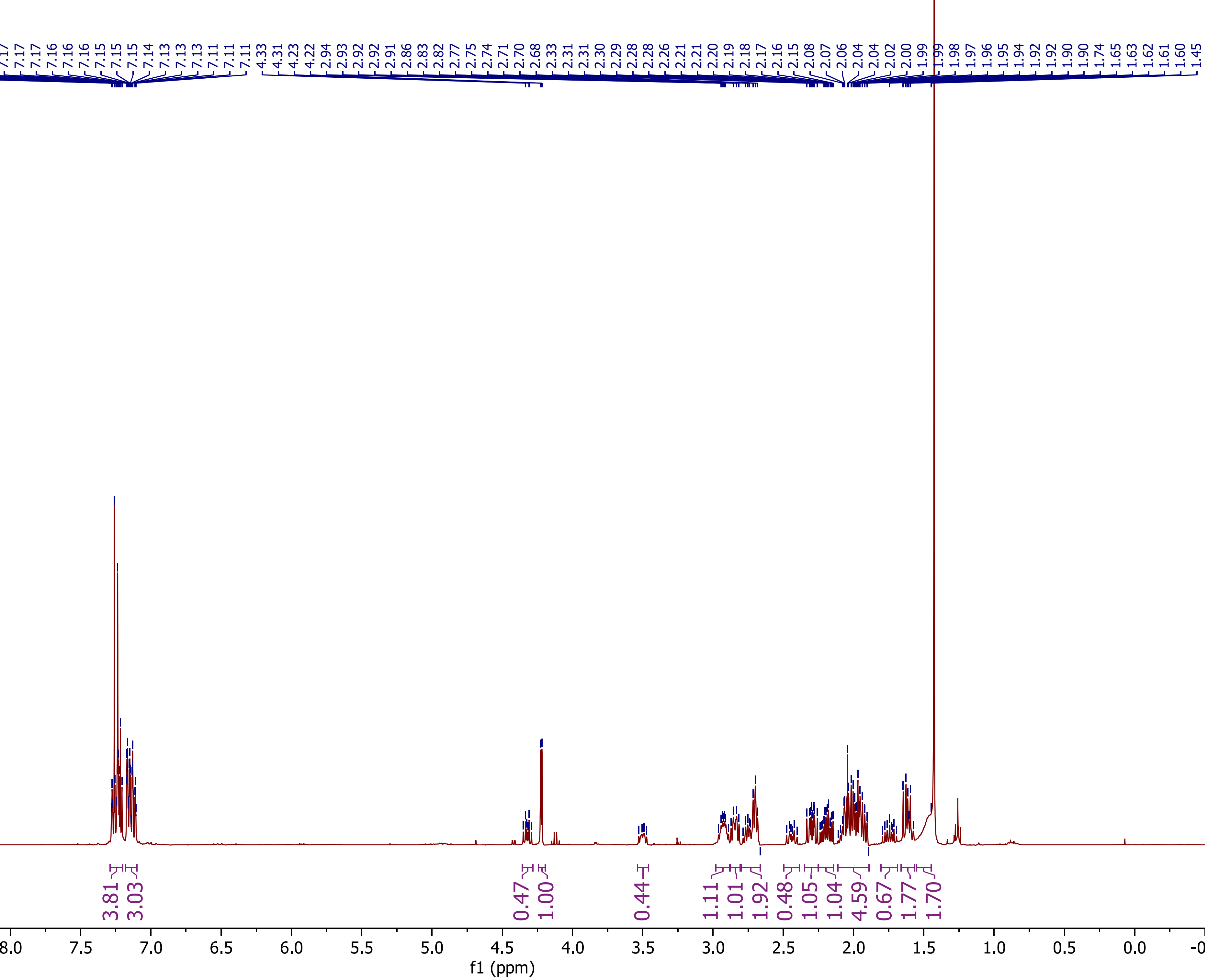

Supplementary Figure 26. ^{13}C NMR spectrum of **SI-17** (101 MHz, $CDCl_3$)

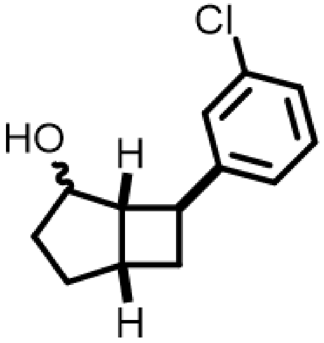

SI-17 (2:1 dr)

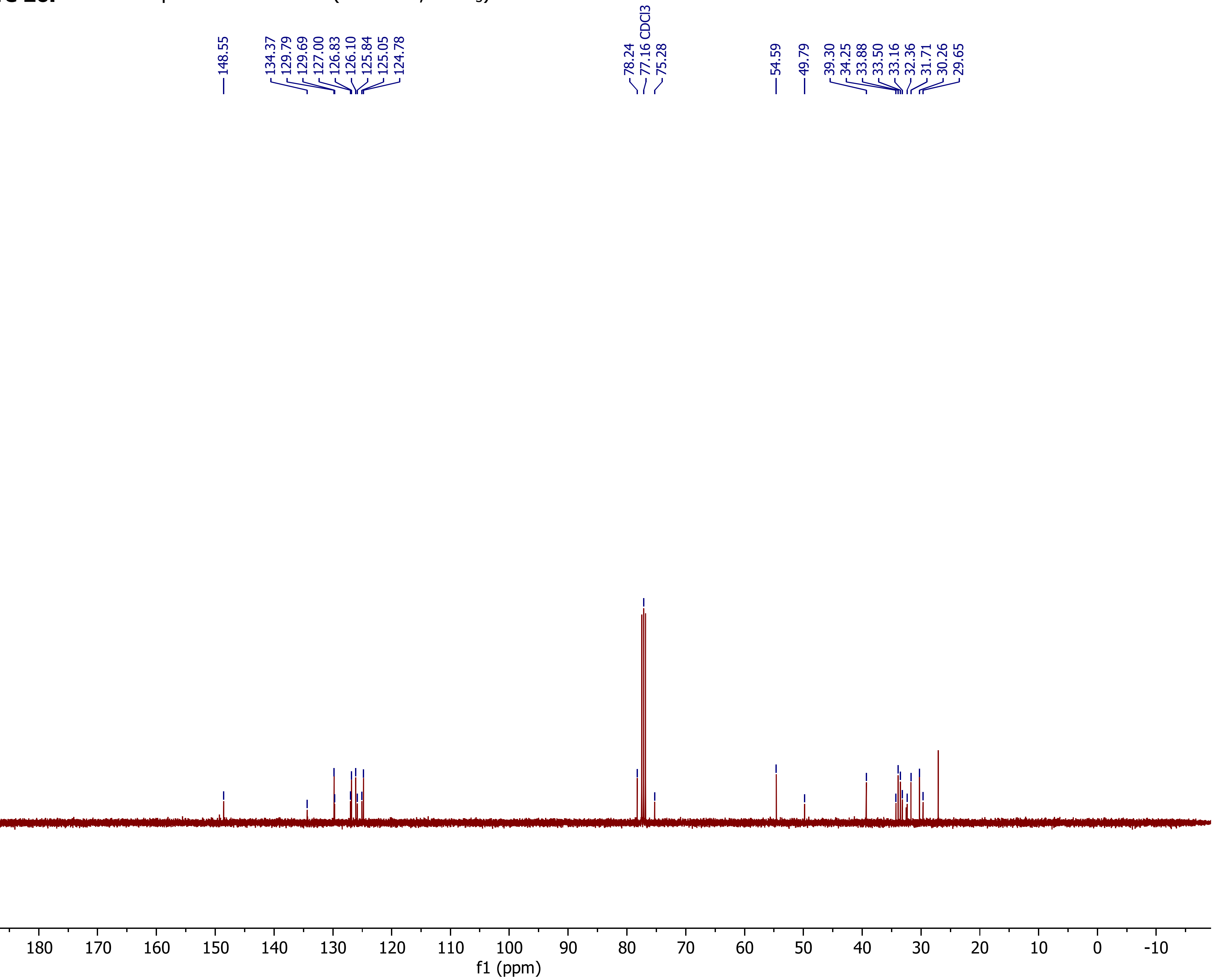

Supplementary Figure 27. ^1H NMR spectrum of **SI-18** (400 MHz, $CDCl_3$)

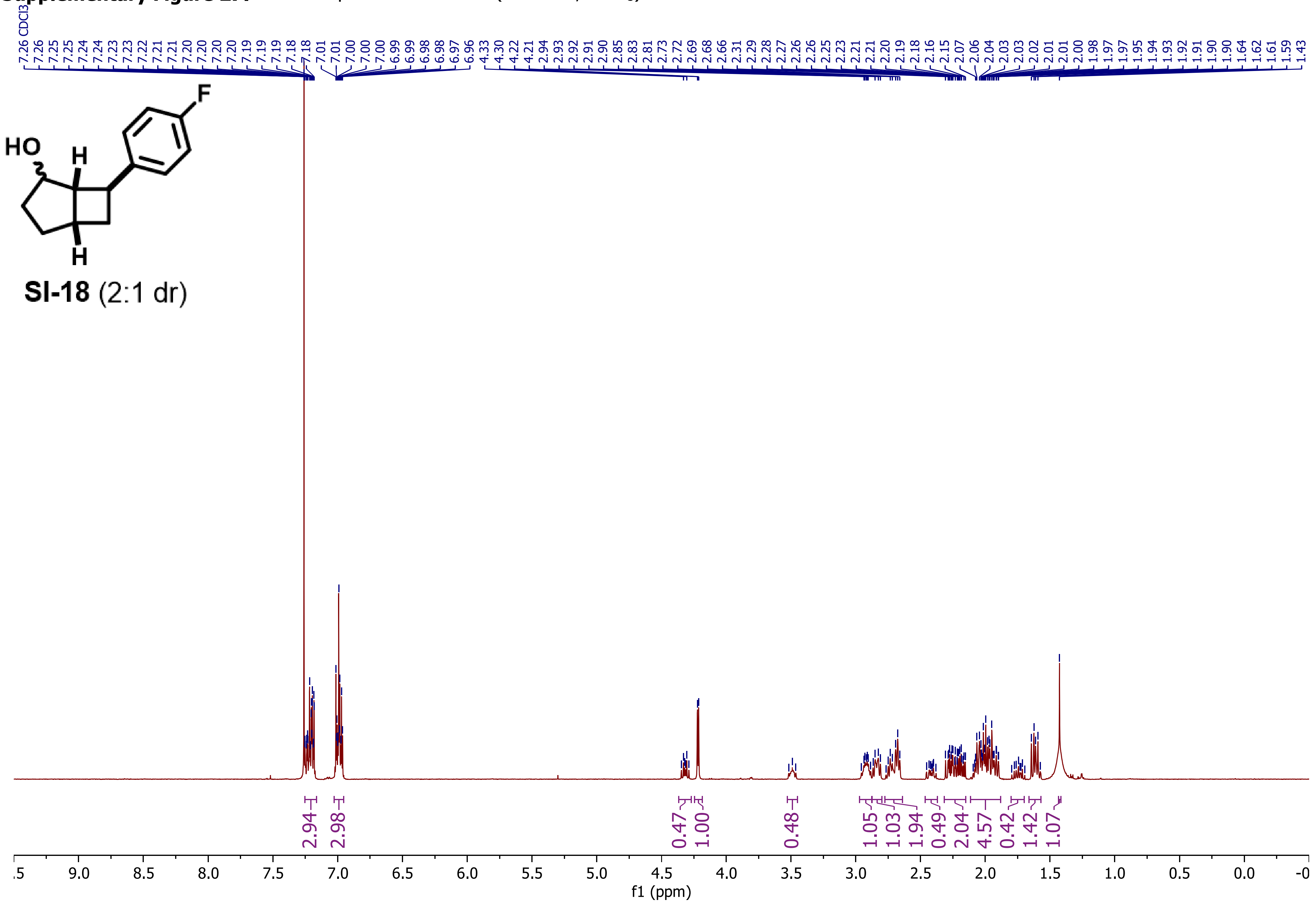

Supplementary Figure 28. ^{13}C NMR spectrum of **SI-18** (101 MHz, $CDCl_3$)

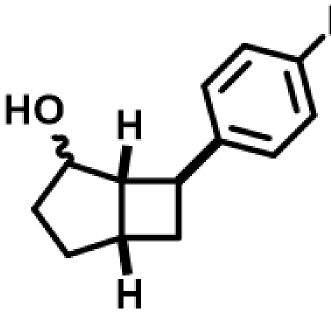

SI-18 (2:1 dr)

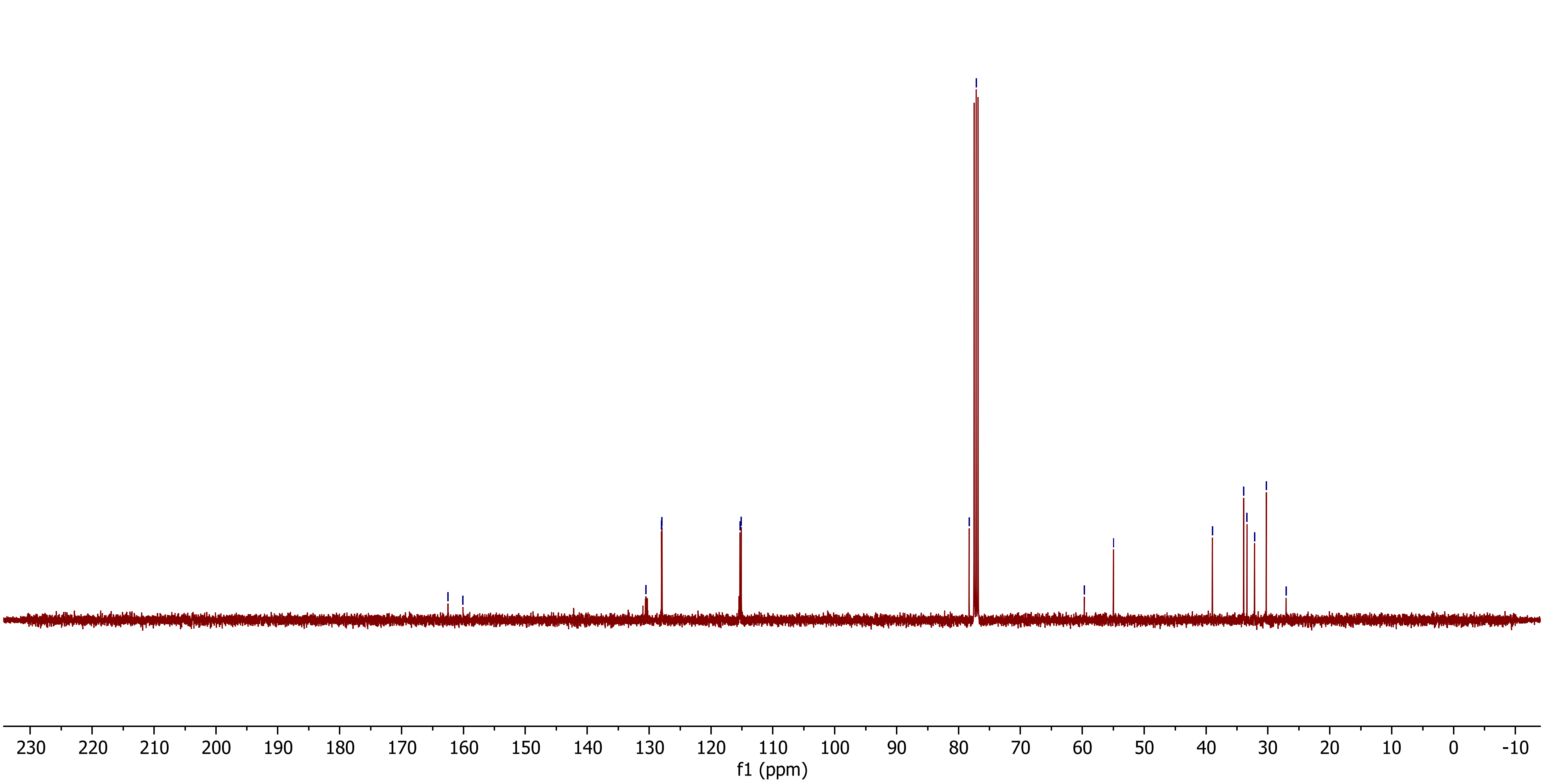

Supplementary Figure 29. ^{1}H NMR spectrum of **SI-19** (400 MHz, $CDCl_3$)

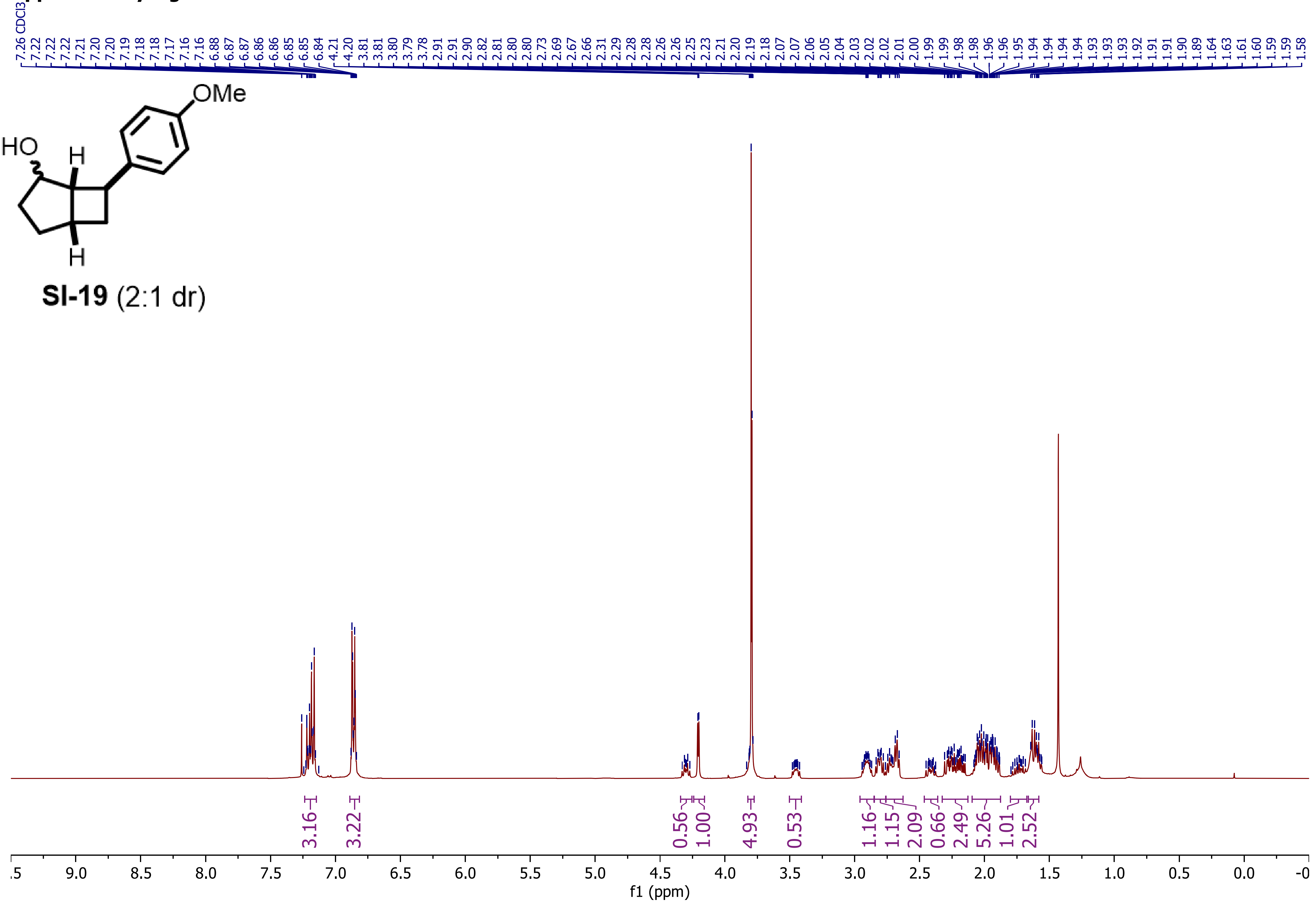

Supplementary Figure 30. ^{13}C NMR spectrum of **SI-19** (101 MHz, $CDCl_3$)

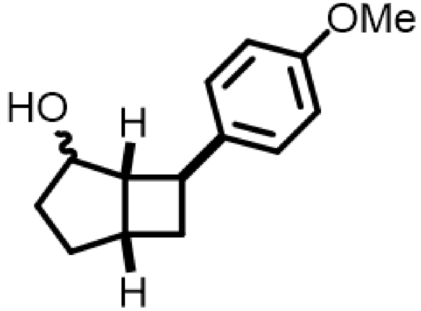

SI-19 (2:1 dr)

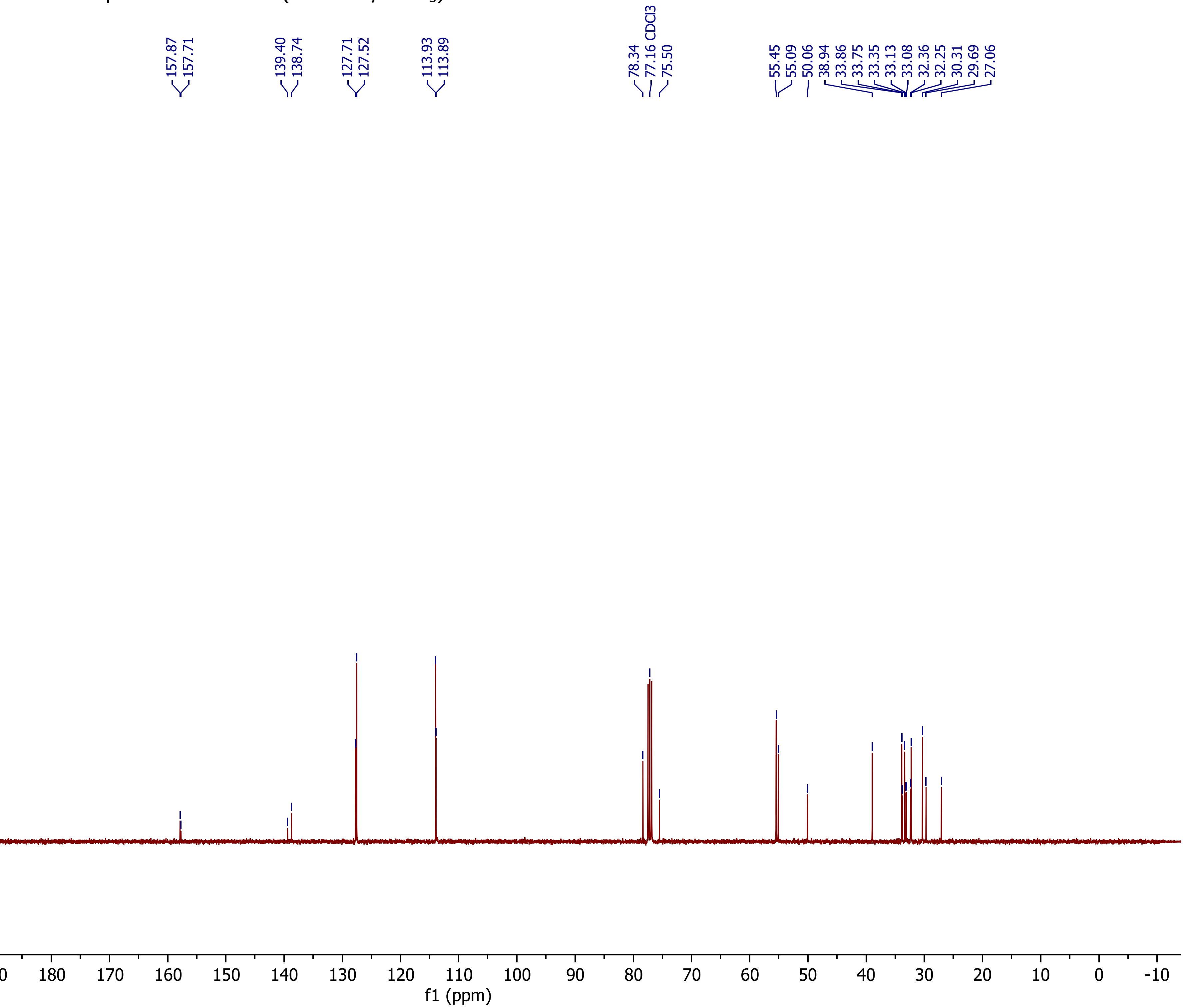

Supplementary Figure 31. ¹H NMR spectrum of **SI-20** (400 MHz, $CDCl_3$)

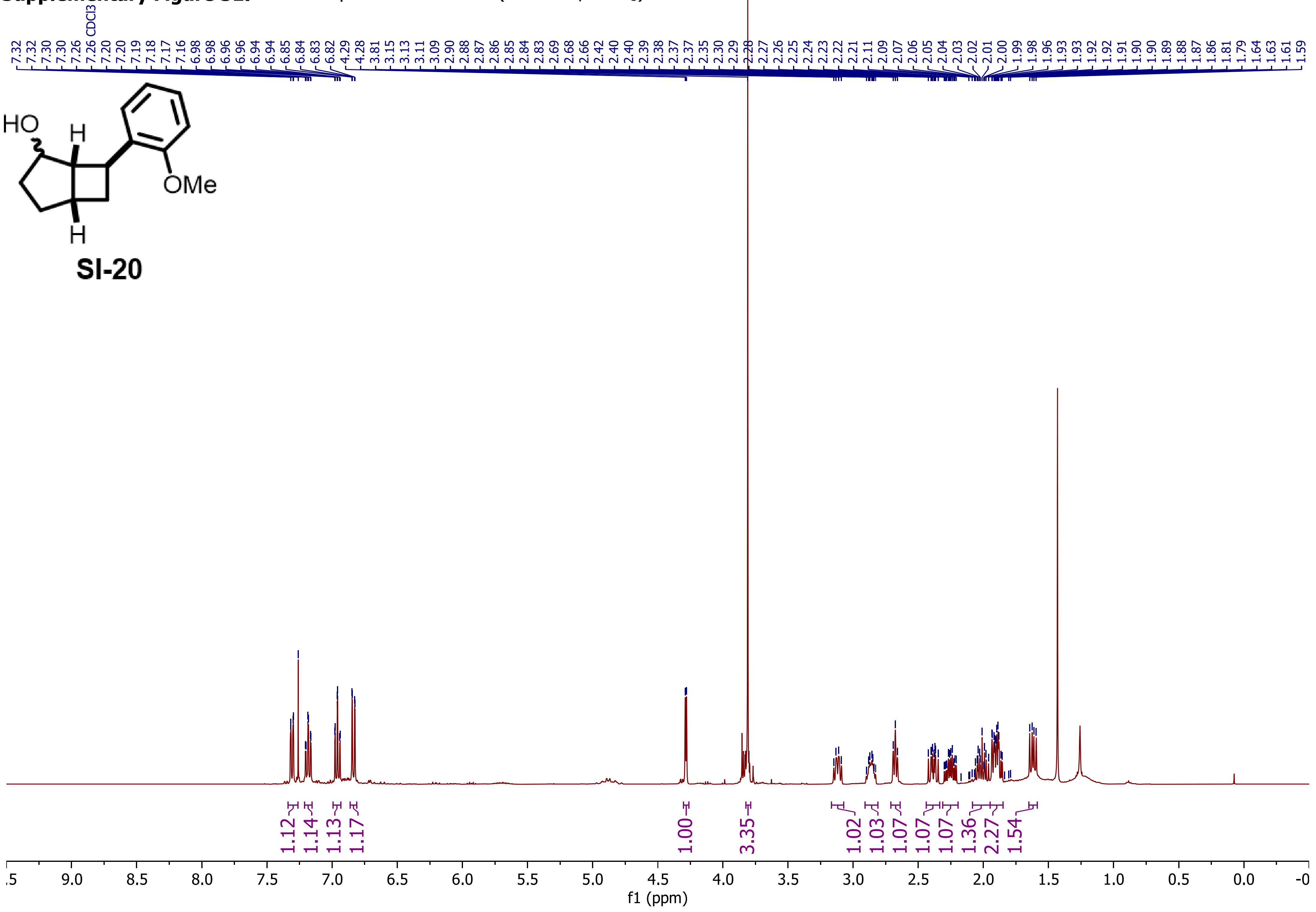

Supplementary Figure 32. ^{13}C NMR spectrum of **SI-20** (101 MHz, $CDCl_3$)

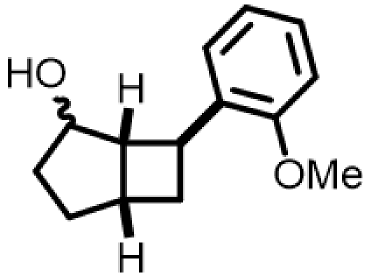

SI-20

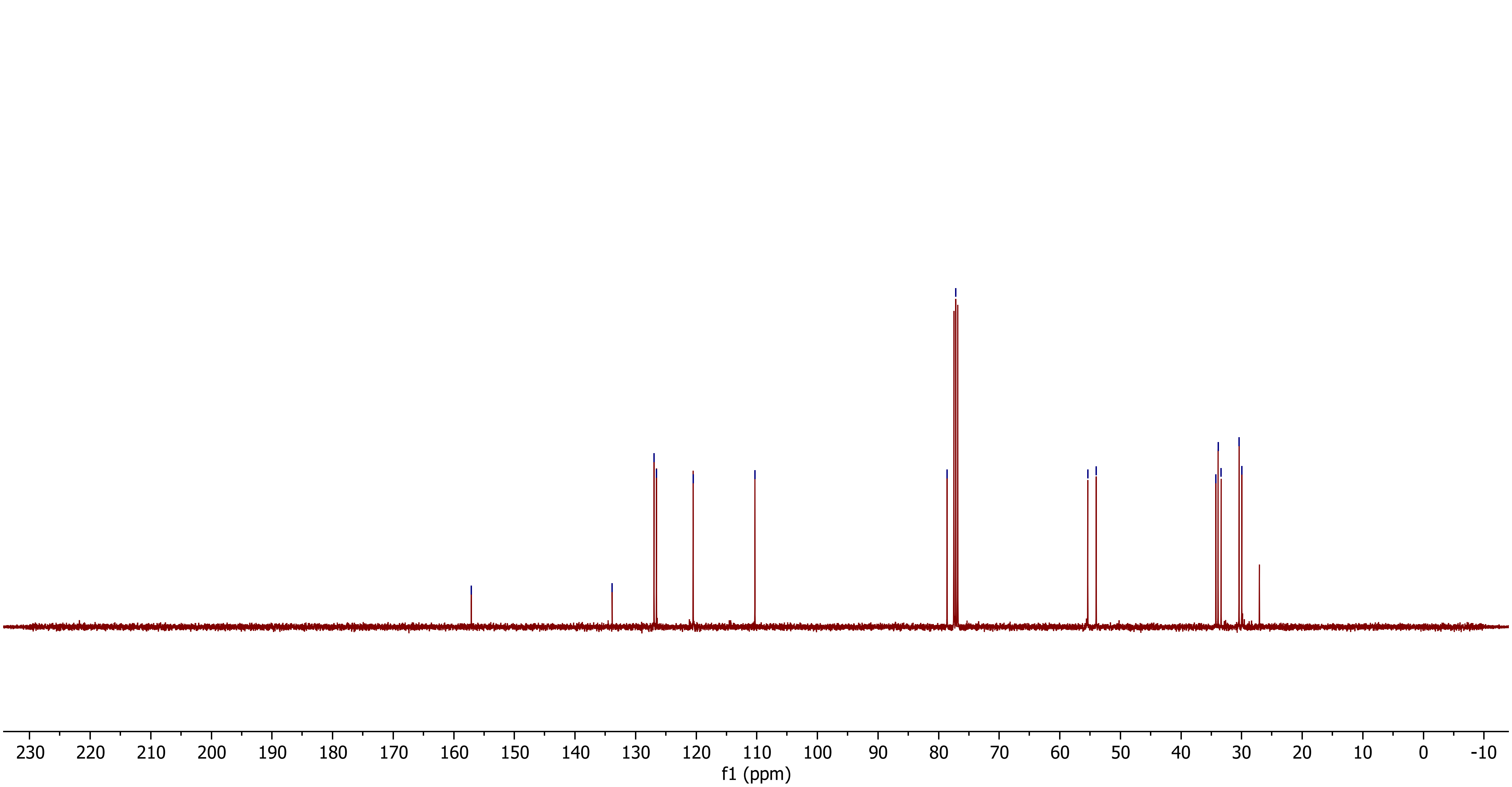

Supplementary Figure 33. ^{1}H NMR spectrum of **SI-21** (400 MHz, $CDCl_3$)

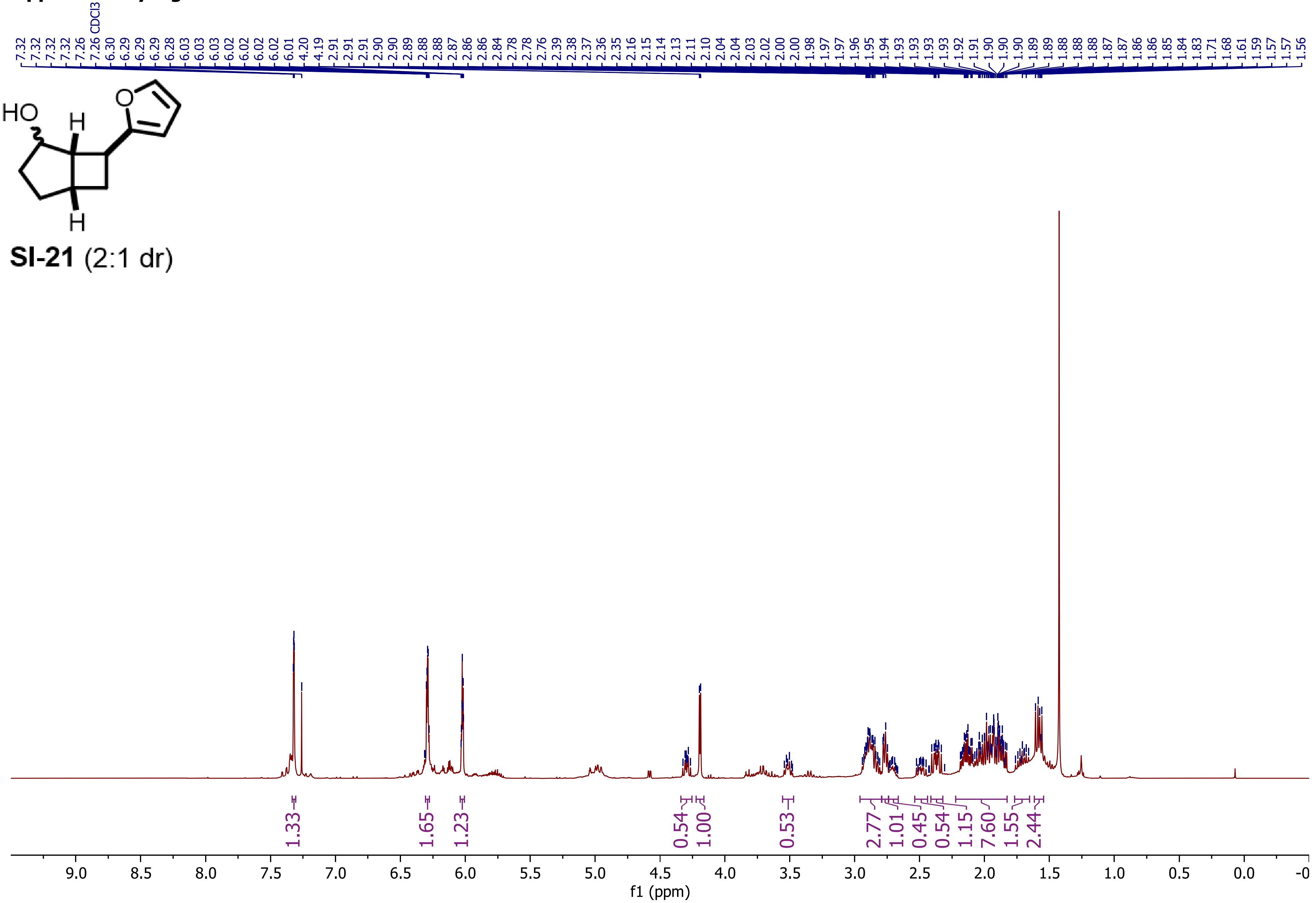

Supplementary Figure 34. ^{13}C NMR spectrum of **SI-21** (101 MHz, $CDCl_3$)

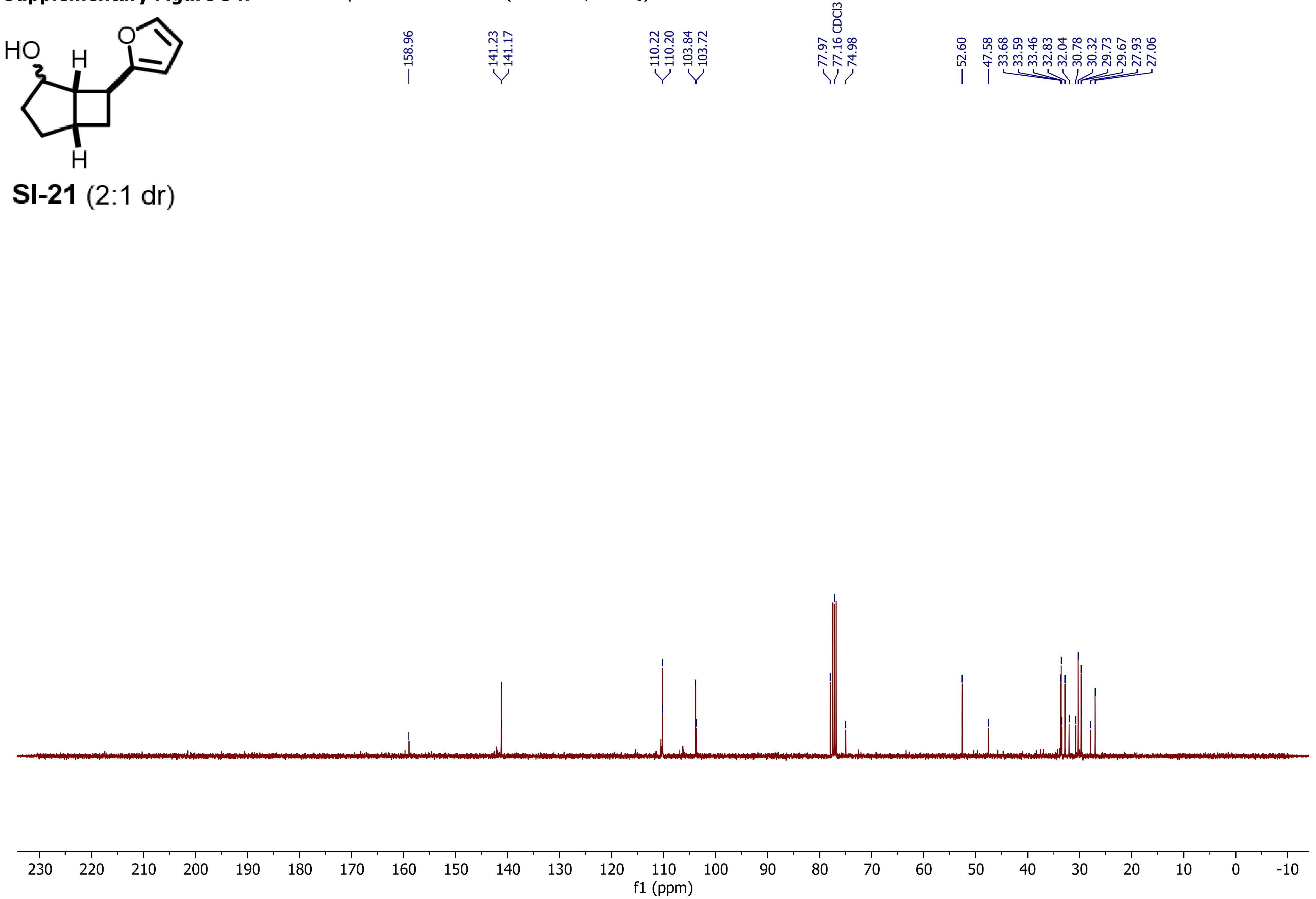

Supplementary Figure 35. ^{1}H NMR spectrum of **SI-22** (500 MHz, $CDCl_3$)

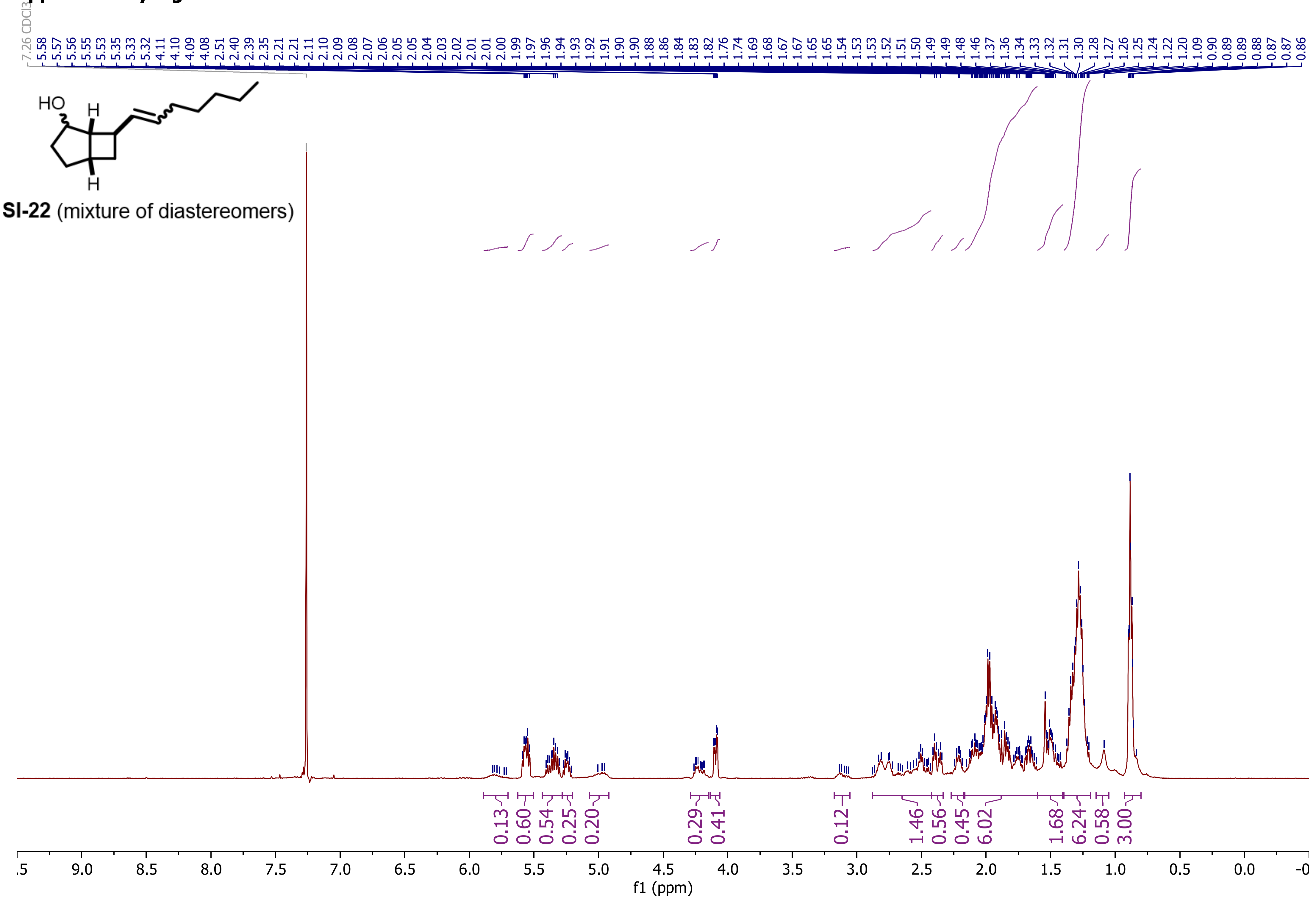

Supplementary Figure 36. ^{1}H NMR spectrum of **SI-23** (500 MHz, $CDCl_3$)

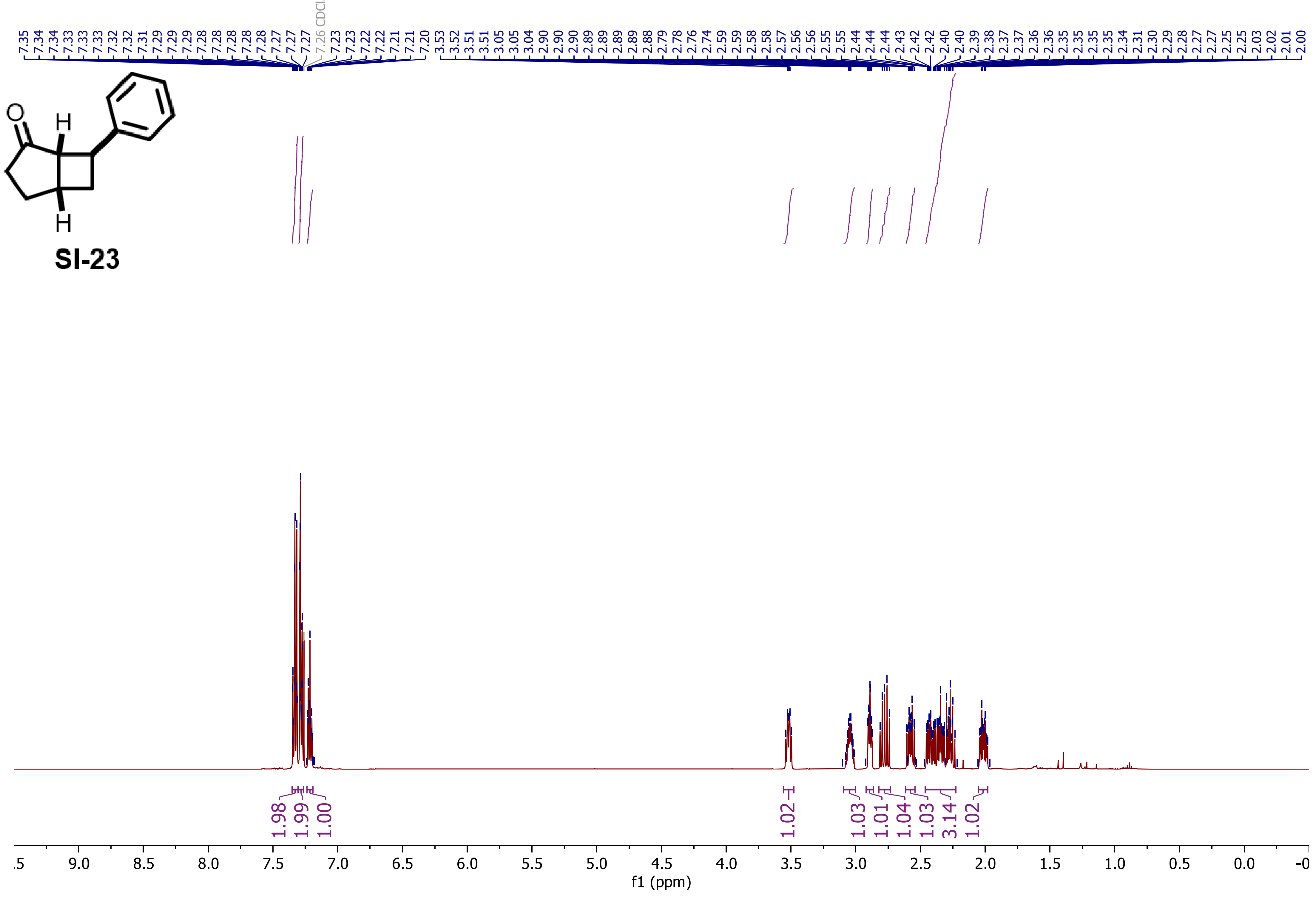

Supplementary Figure 37. ^{13}C NMR spectrum of **SI-23** (126 MHz, $CDCl_3$)

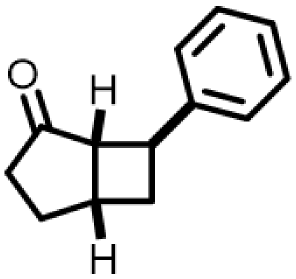

SI-23

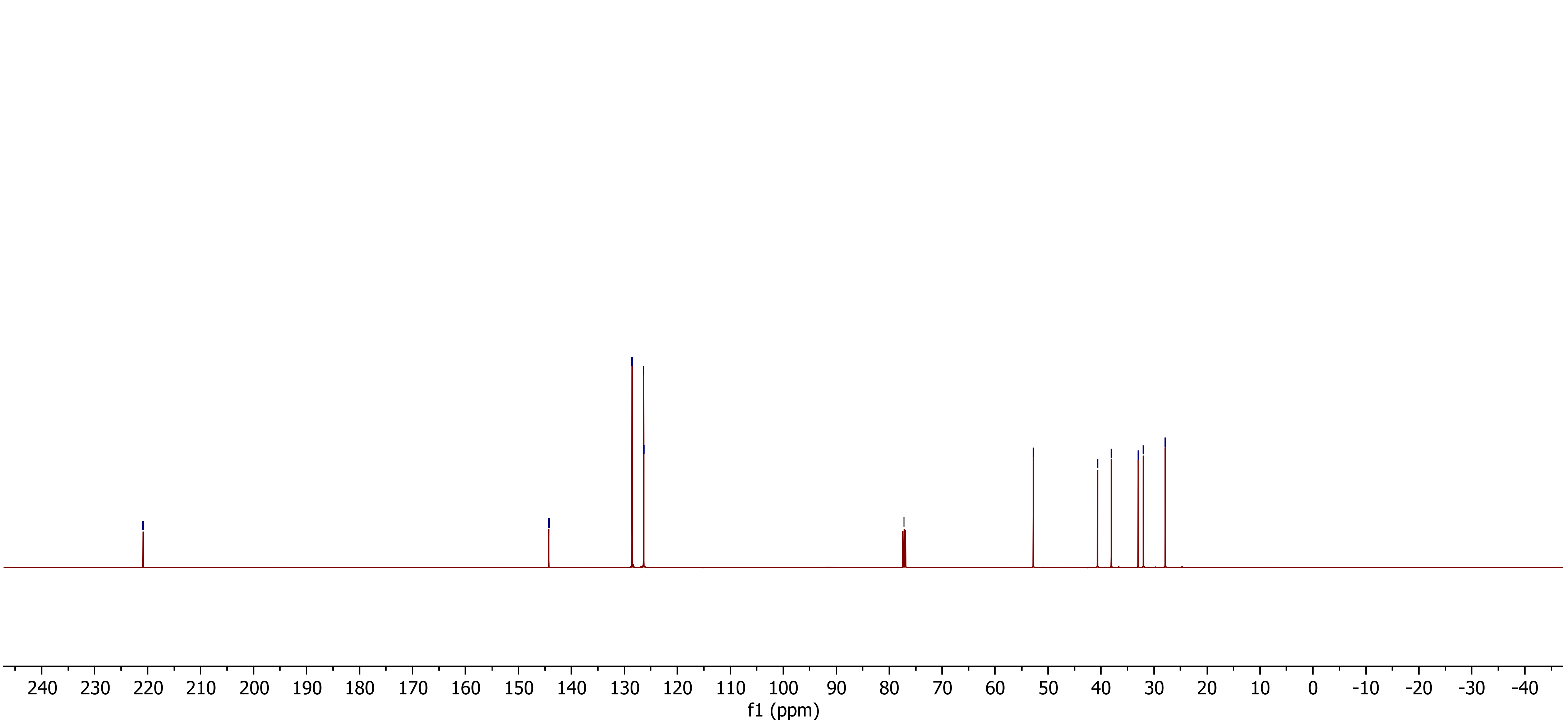

Supplementary Figure 38. ^{1}H NMR spectrum of **SI-24** (300 MHz, $CDCl_3$)

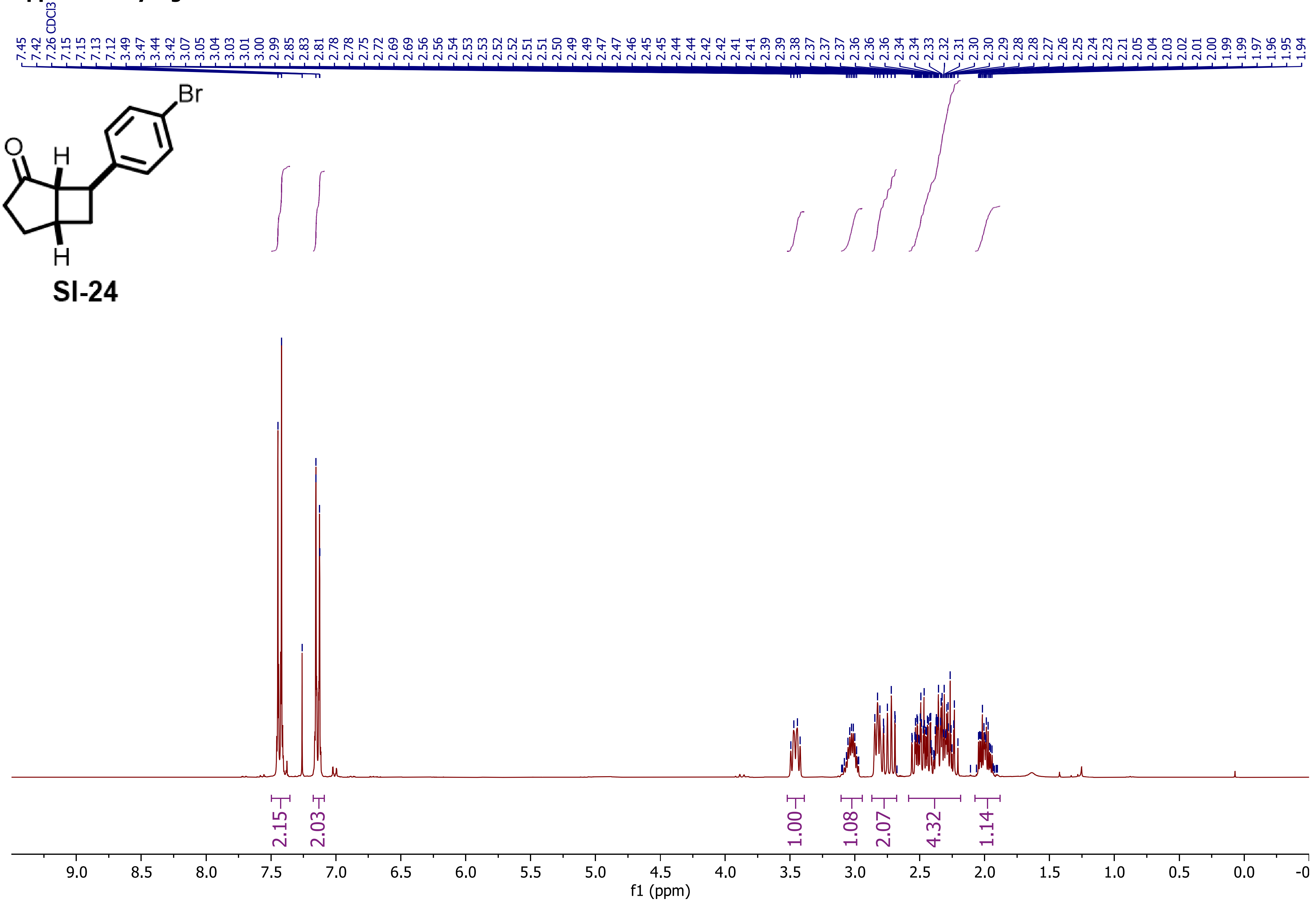

Supplementary Figure 39. ^{13}C NMR spectrum of **SI-24** (75 MHz, $CDCl_3$)

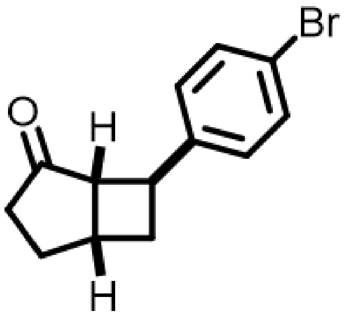

SI-24

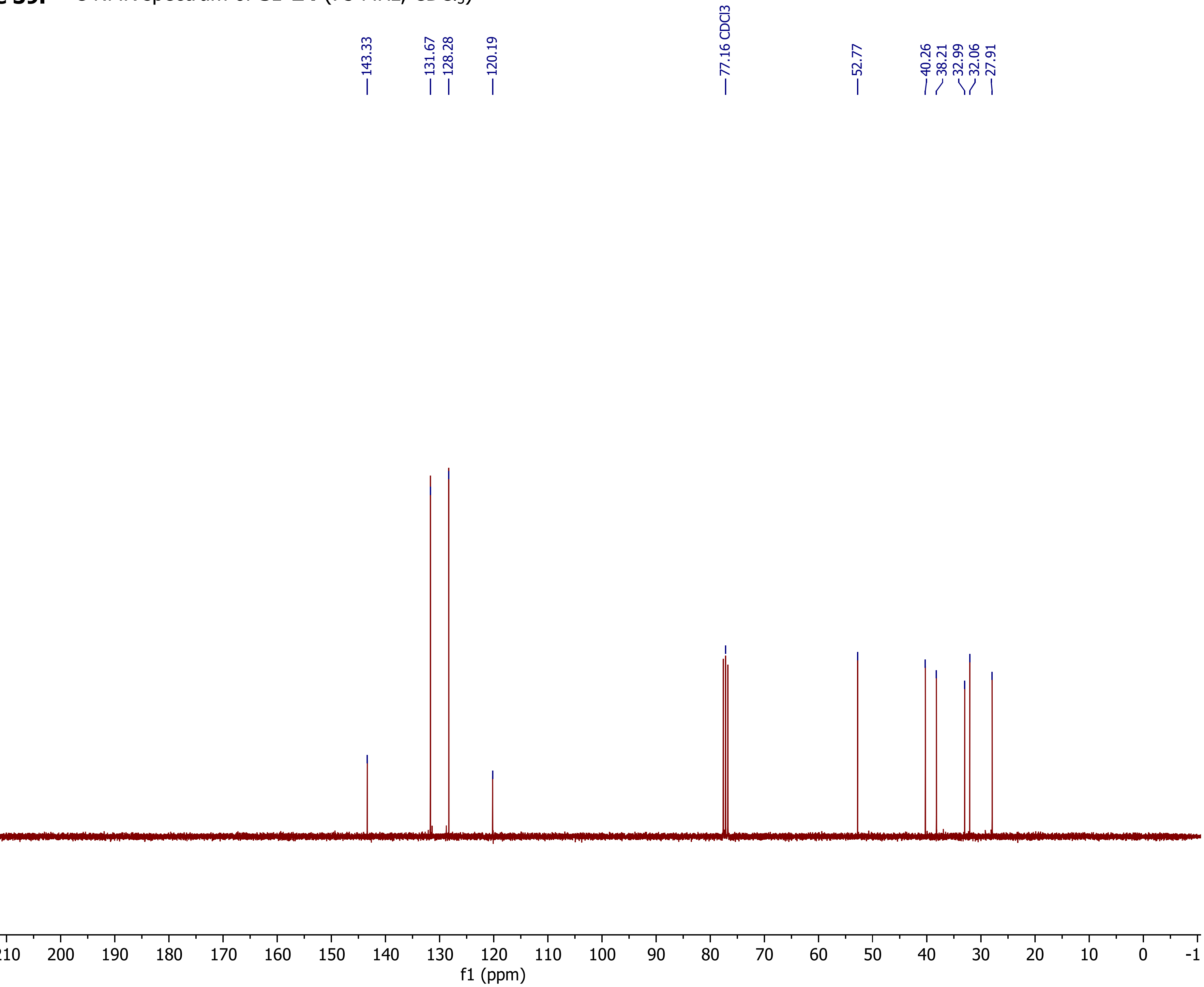

Supplementary Figure 40. ^{1}H NMR spectrum of **SI-25** (300 MHz, $CDCl_3$)

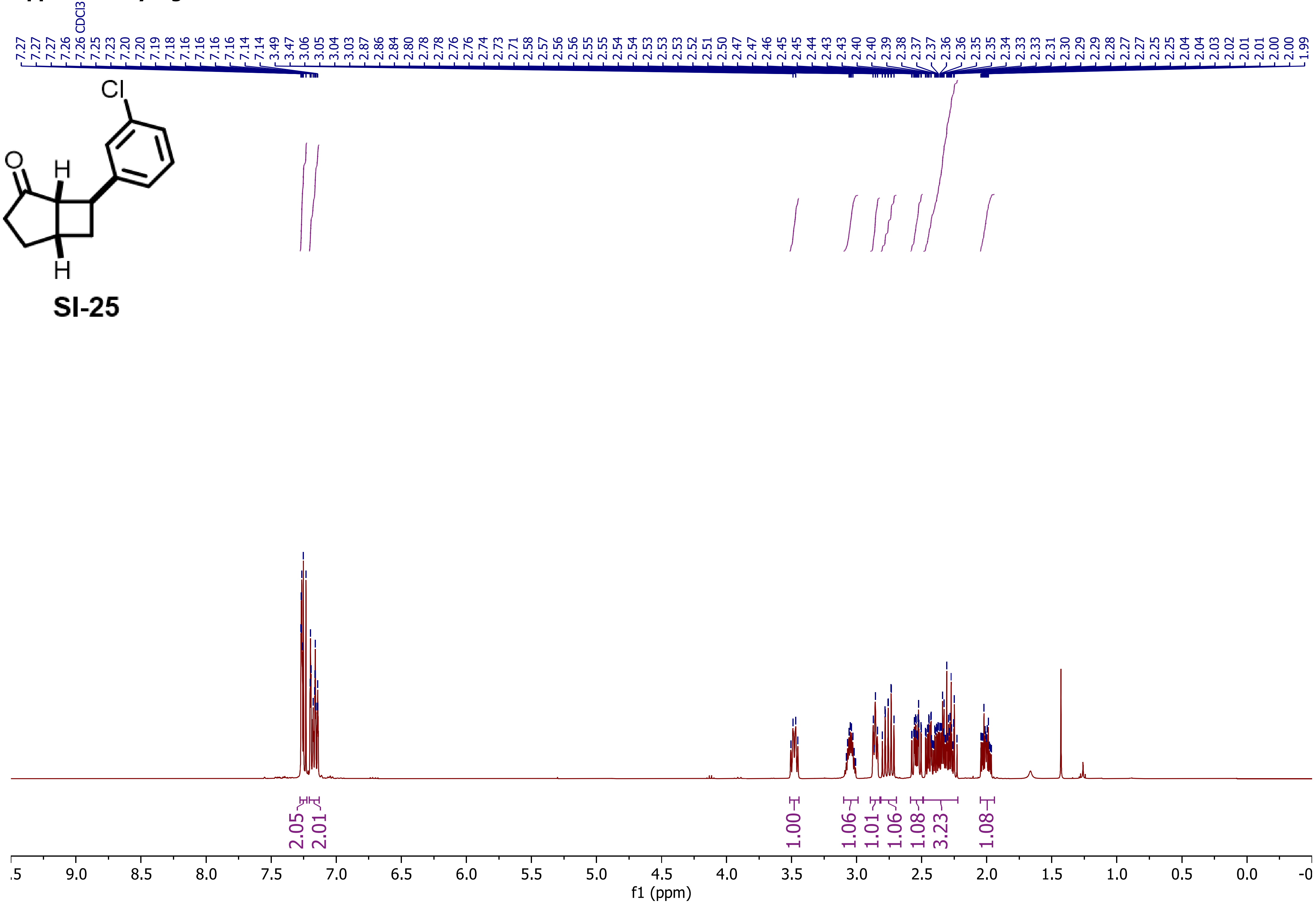

SI-25

Supplementary Figure 41. ^{13}C NMR spectrum of **SI-25** (75 MHz, $CDCl_3$)

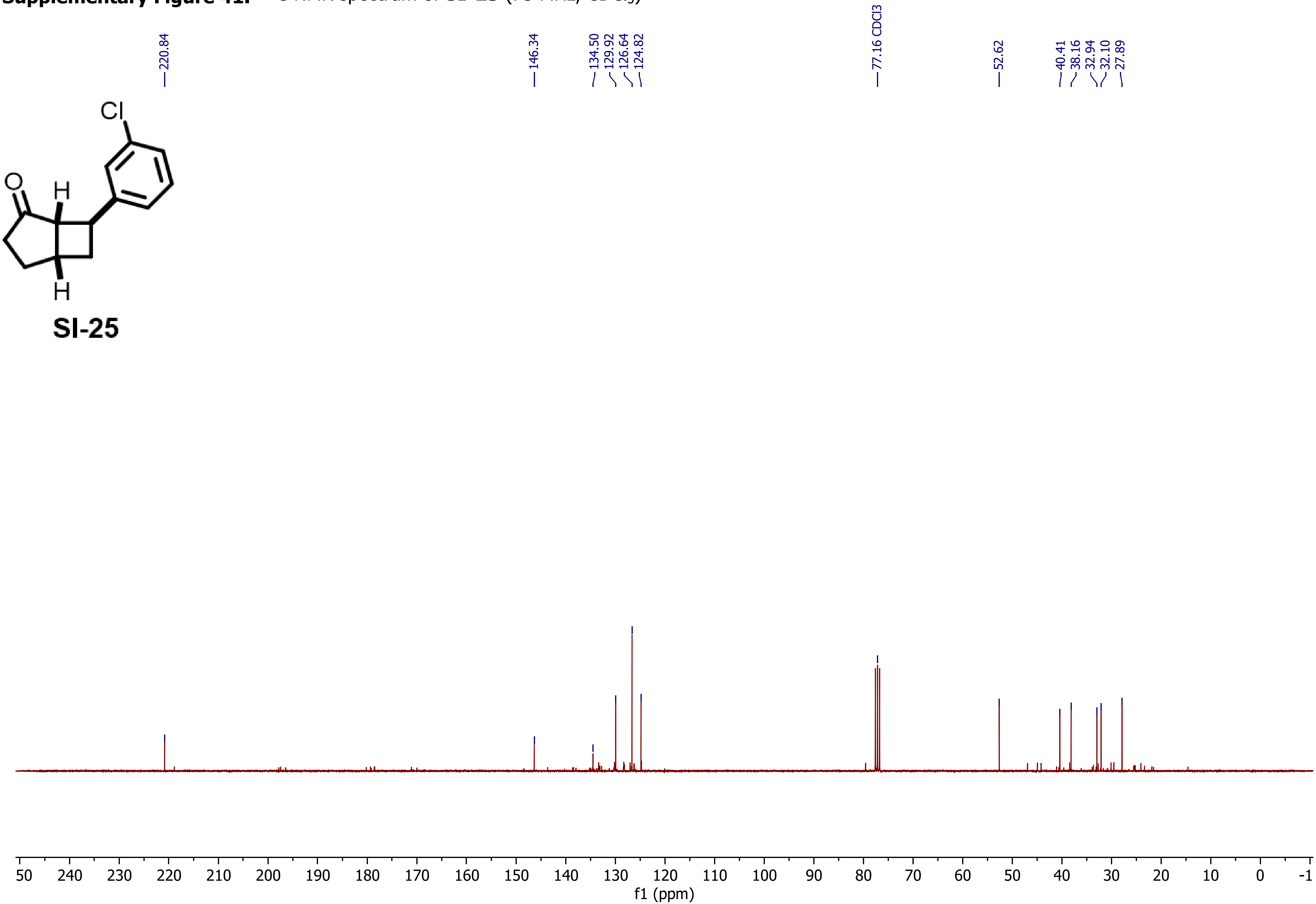

Supplementary Figure 42. ^{1}H NMR spectrum of **SI-26** (300 MHz, $CDCl_3$)

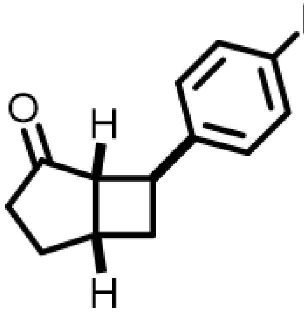

SI-26

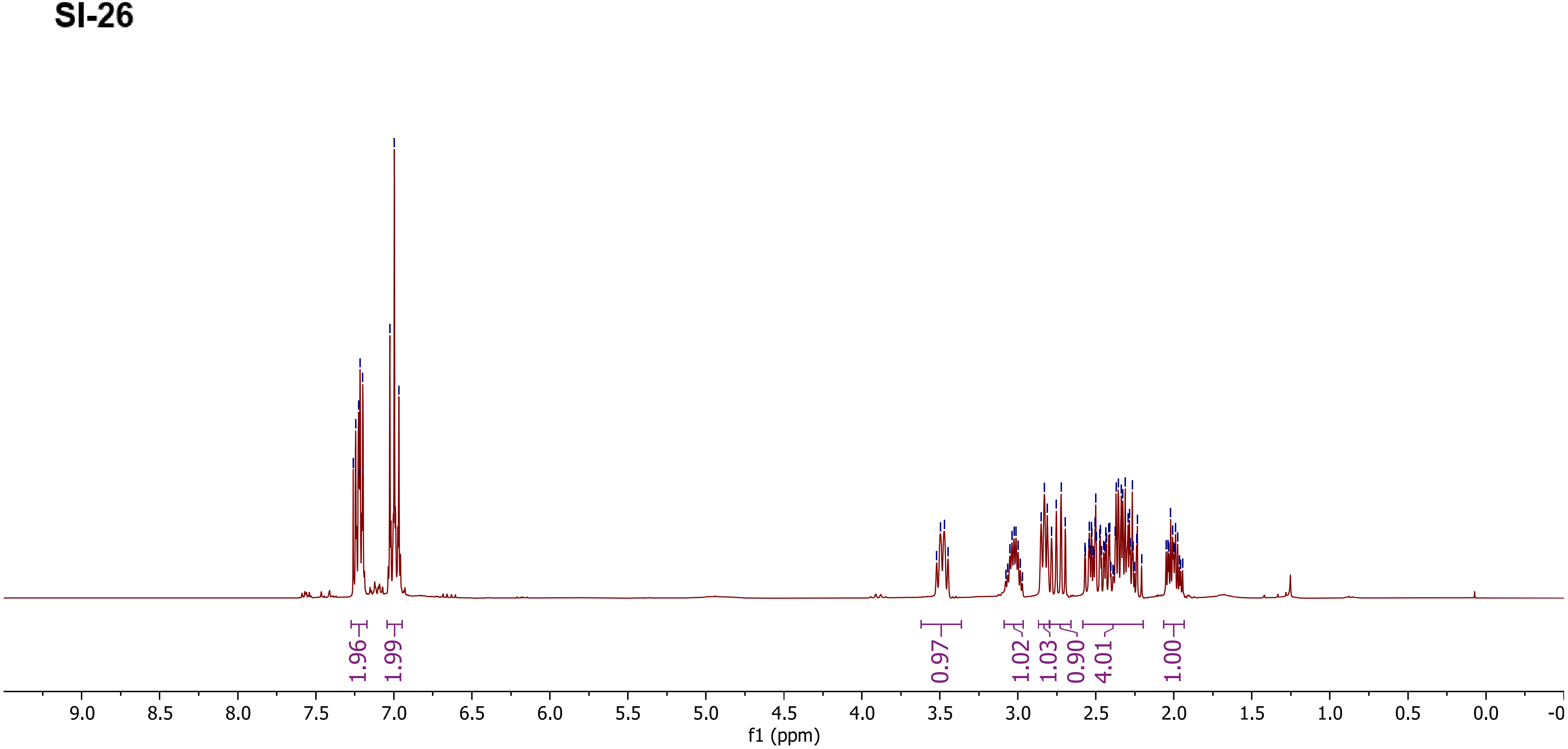

Supplementary Figure 43. ^{19}F NMR spectrum of **SI-26** (282 MHz, $CDCl_3$)

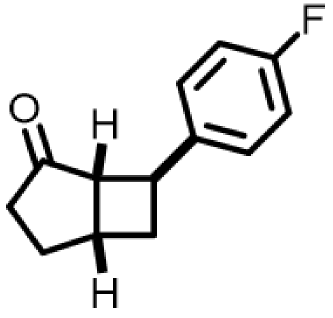

SI-26

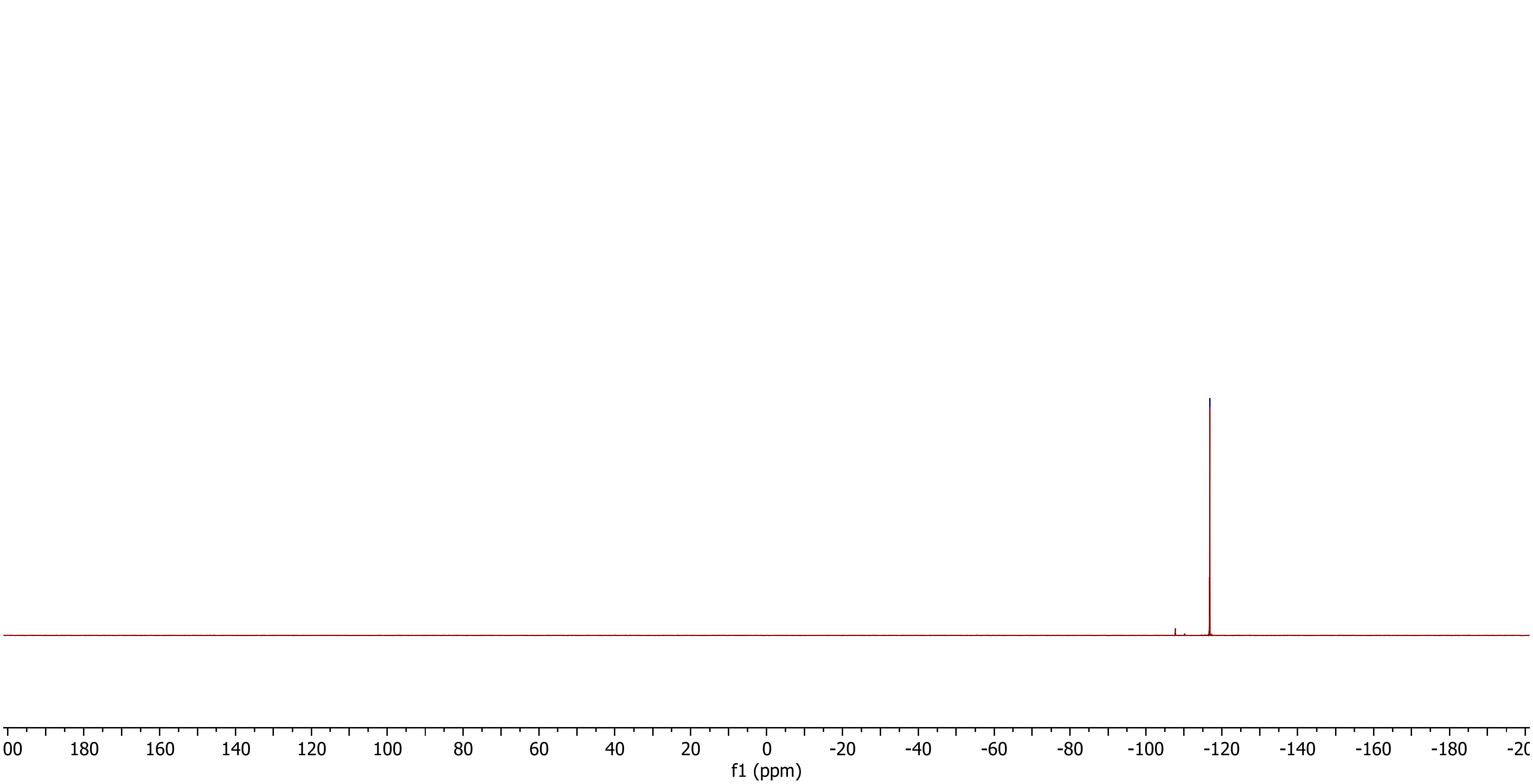

Supplementary Figure 44. ^{13}C NMR spectrum of **SI-26** (75 MHz, $CDCl_3$)

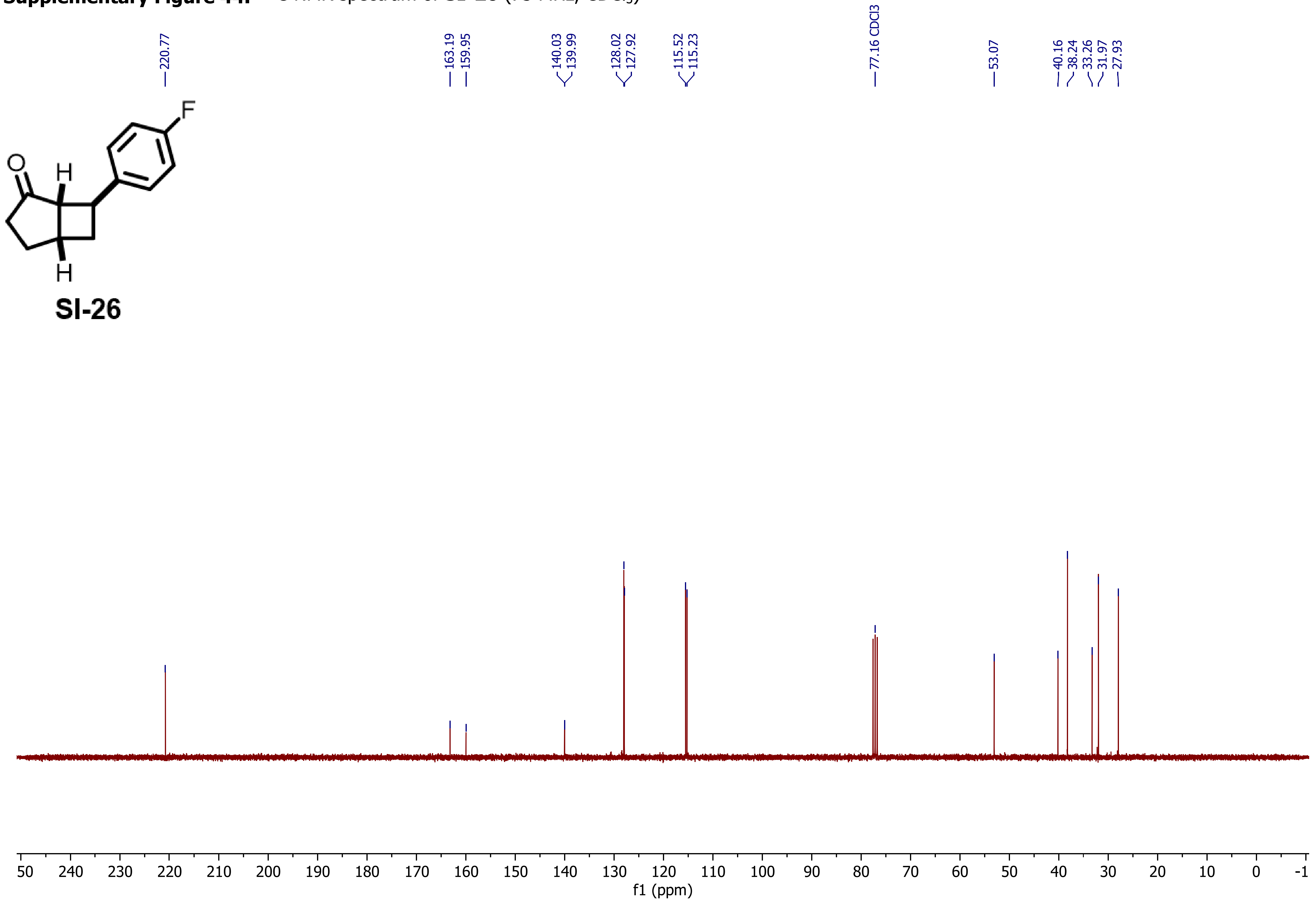

Supplementary Figure 45. ^{1}H NMR spectrum of **SI-27** (400 MHz, $CDCl_3$)

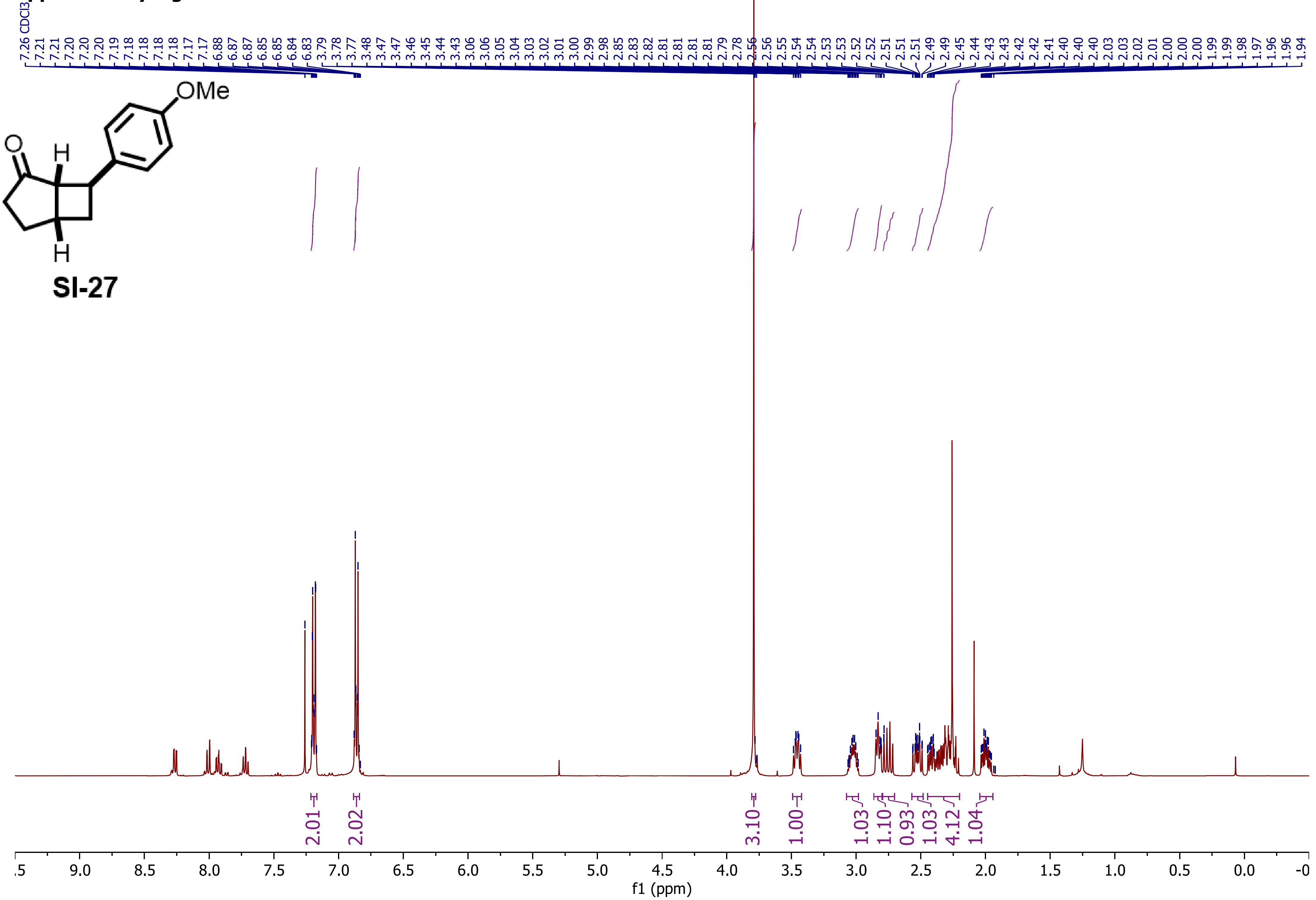

Supplementary Figure 46. ^{13}C NMR spectrum of **SI-27** (101 MHz, $CDCl_3$)

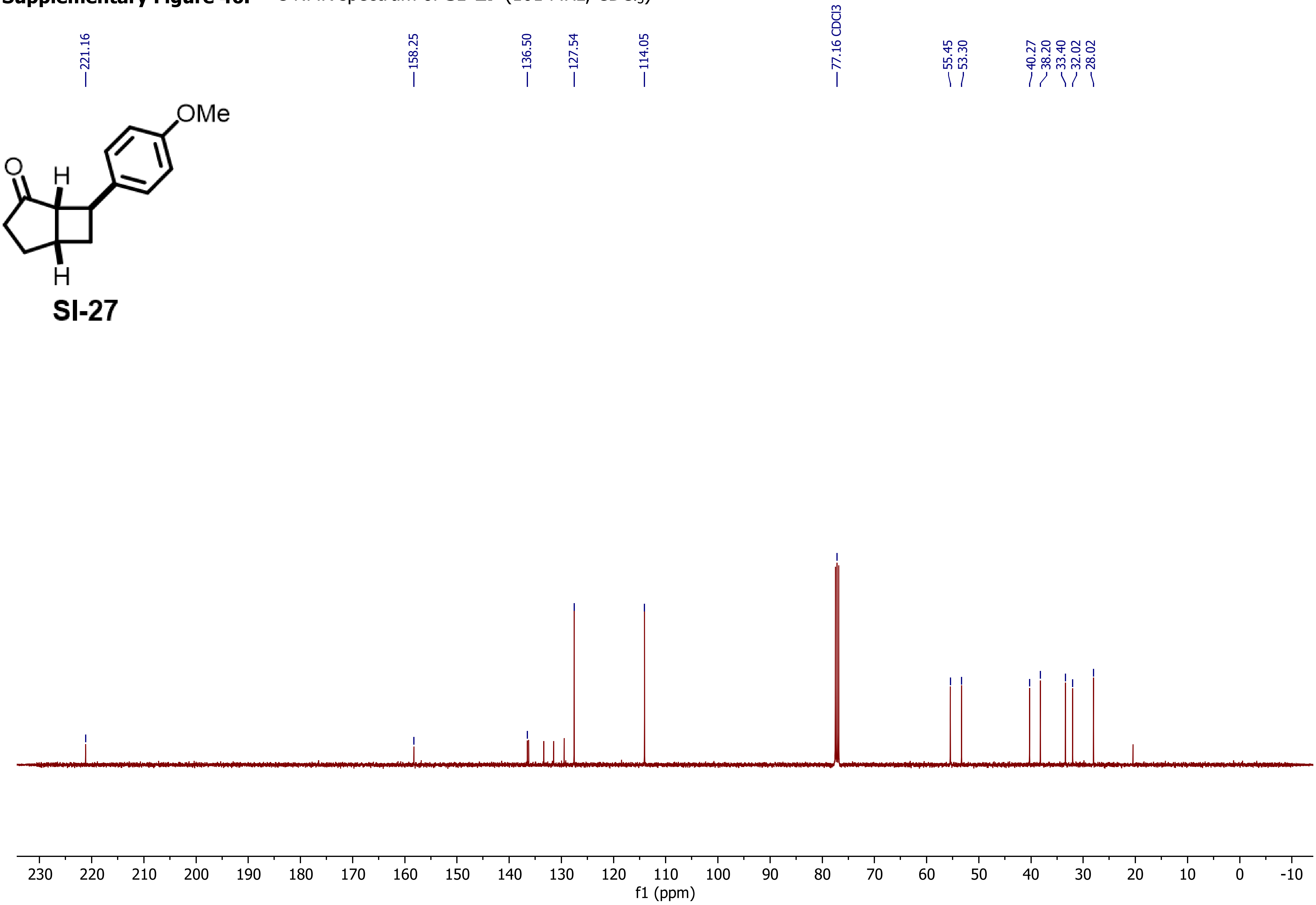

Supplementary Figure 47. ^{1}H NMR spectrum of **SI-28** (400 MHz, $CDCl_3$)

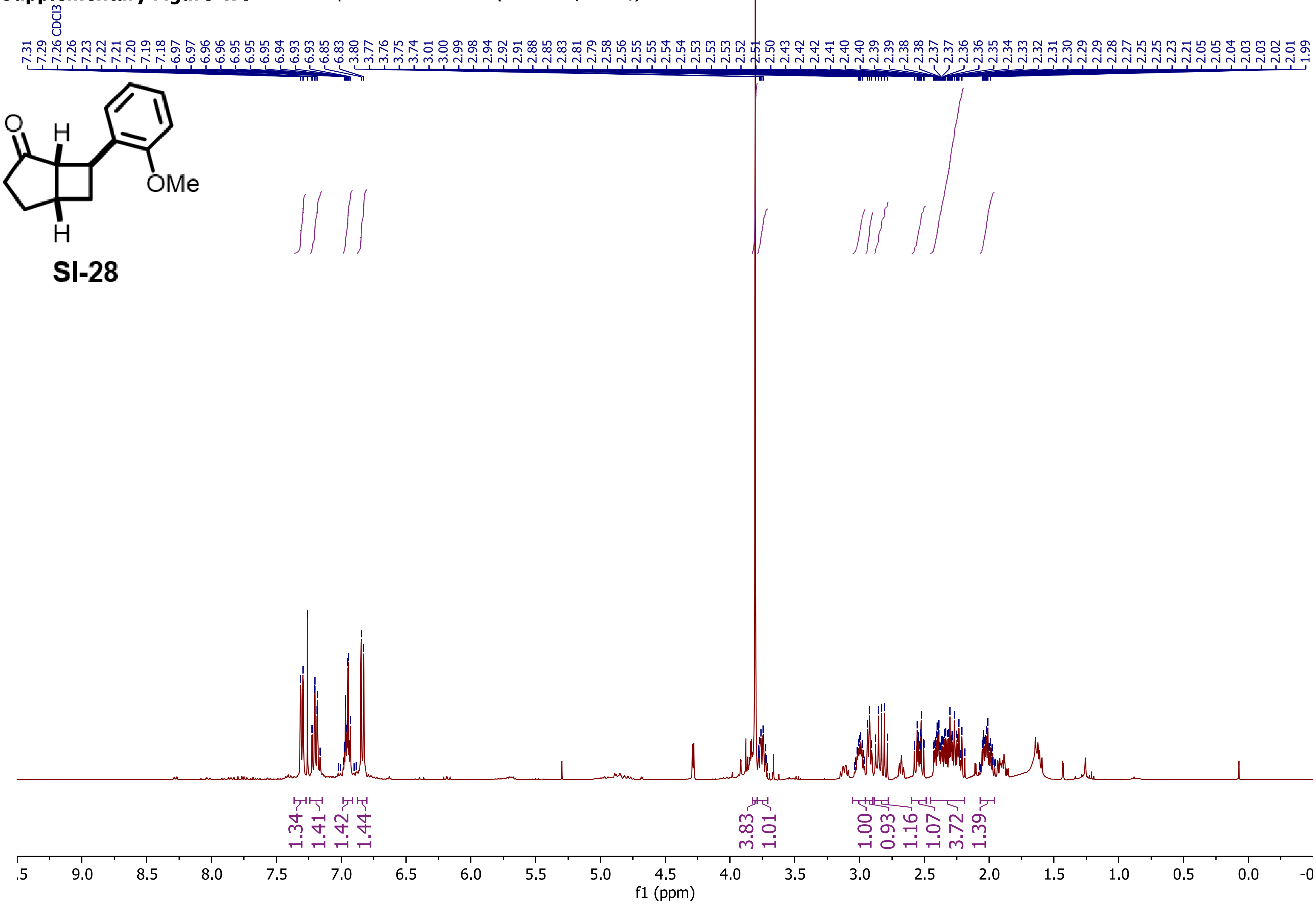

Supplementary Figure 48. ^{13}C NMR spectrum of **SI-28** (101 MHz, $CDCl_3$)

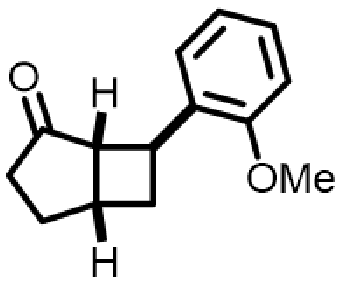

SI-28

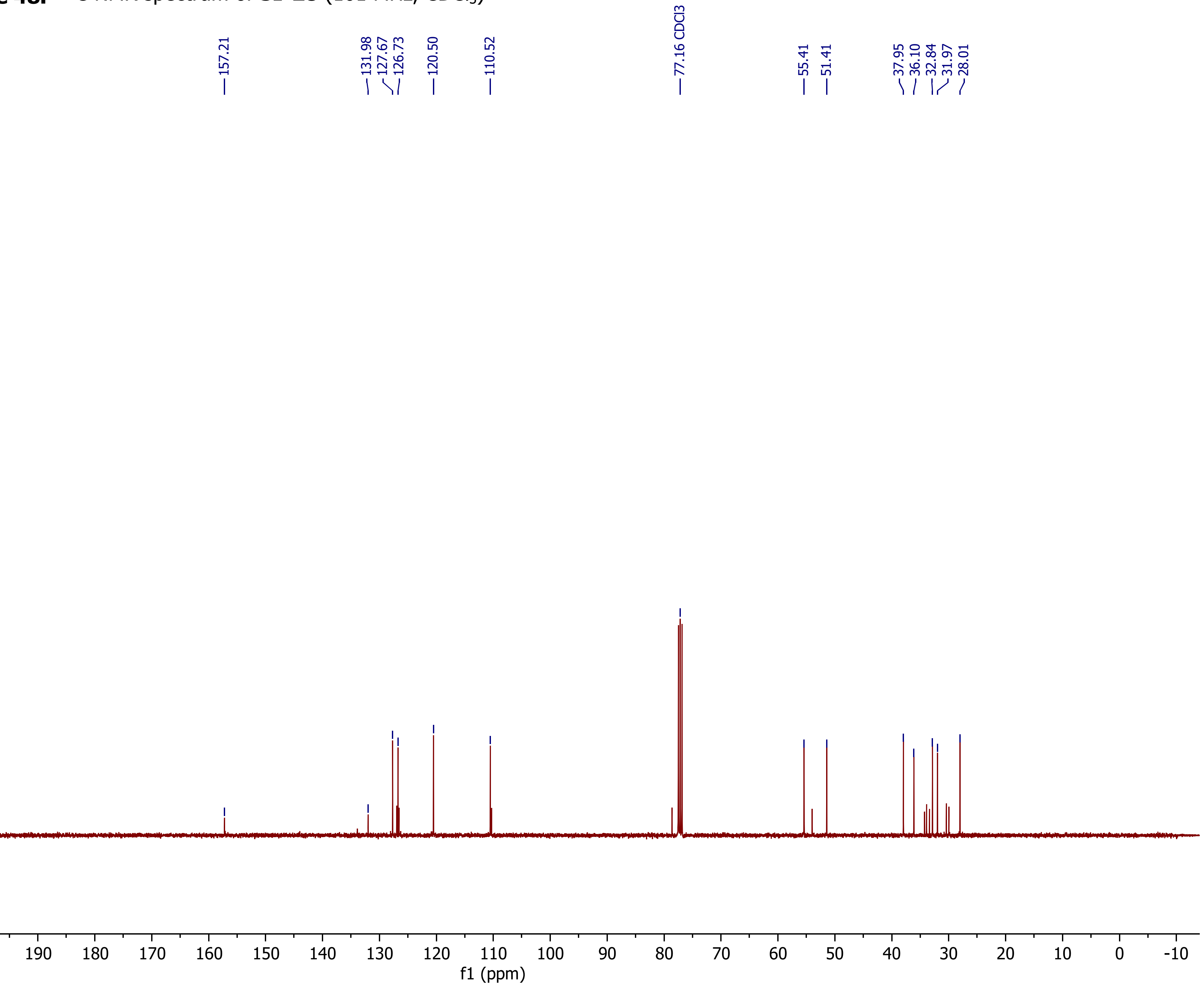

Supplementary Figure 49. ^{1}H NMR spectrum of **SI-29** (300 MHz, $CDCl_3$)

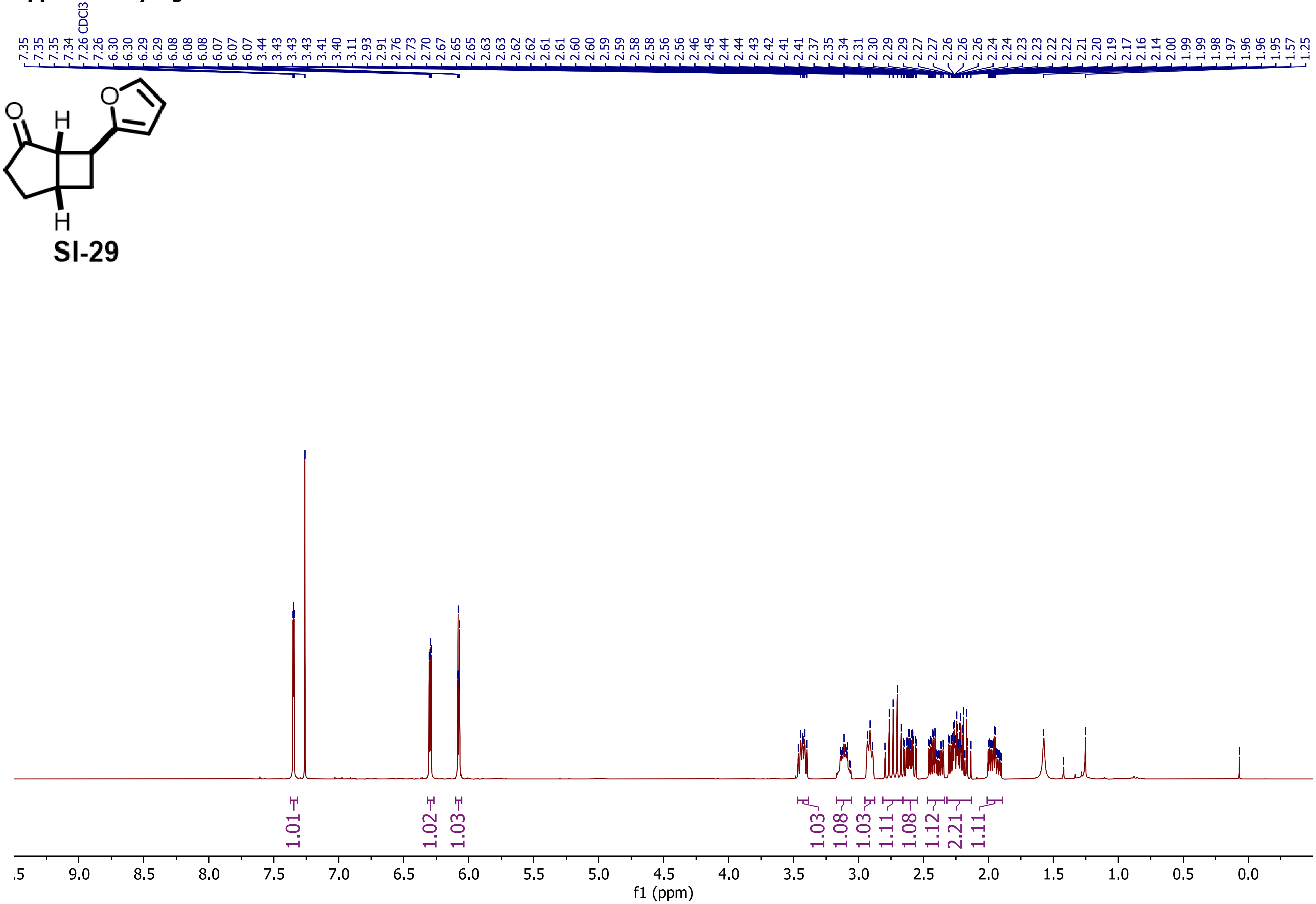

Supplementary Figure 50. ^{13}C NMR spectrum of **SI-29** (75 MHz, $CDCl_3$)

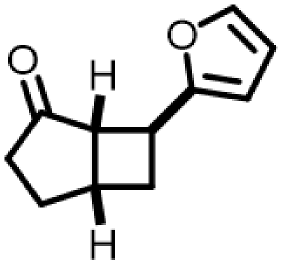

SI-29

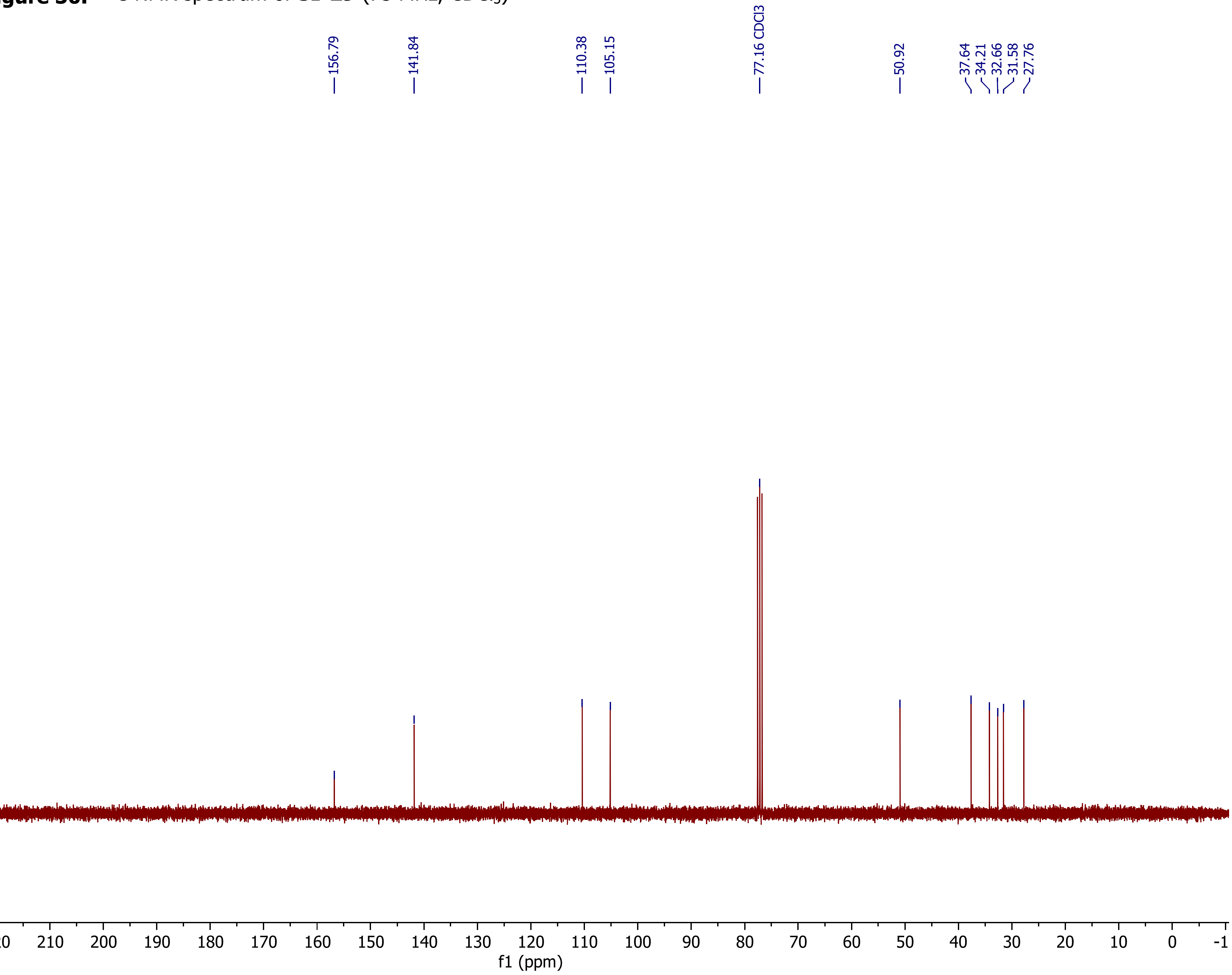

Supplementary Figure 51. ^{1}H NMR spectrum of **SI-30** (500 MHz, $CDCl_3$)

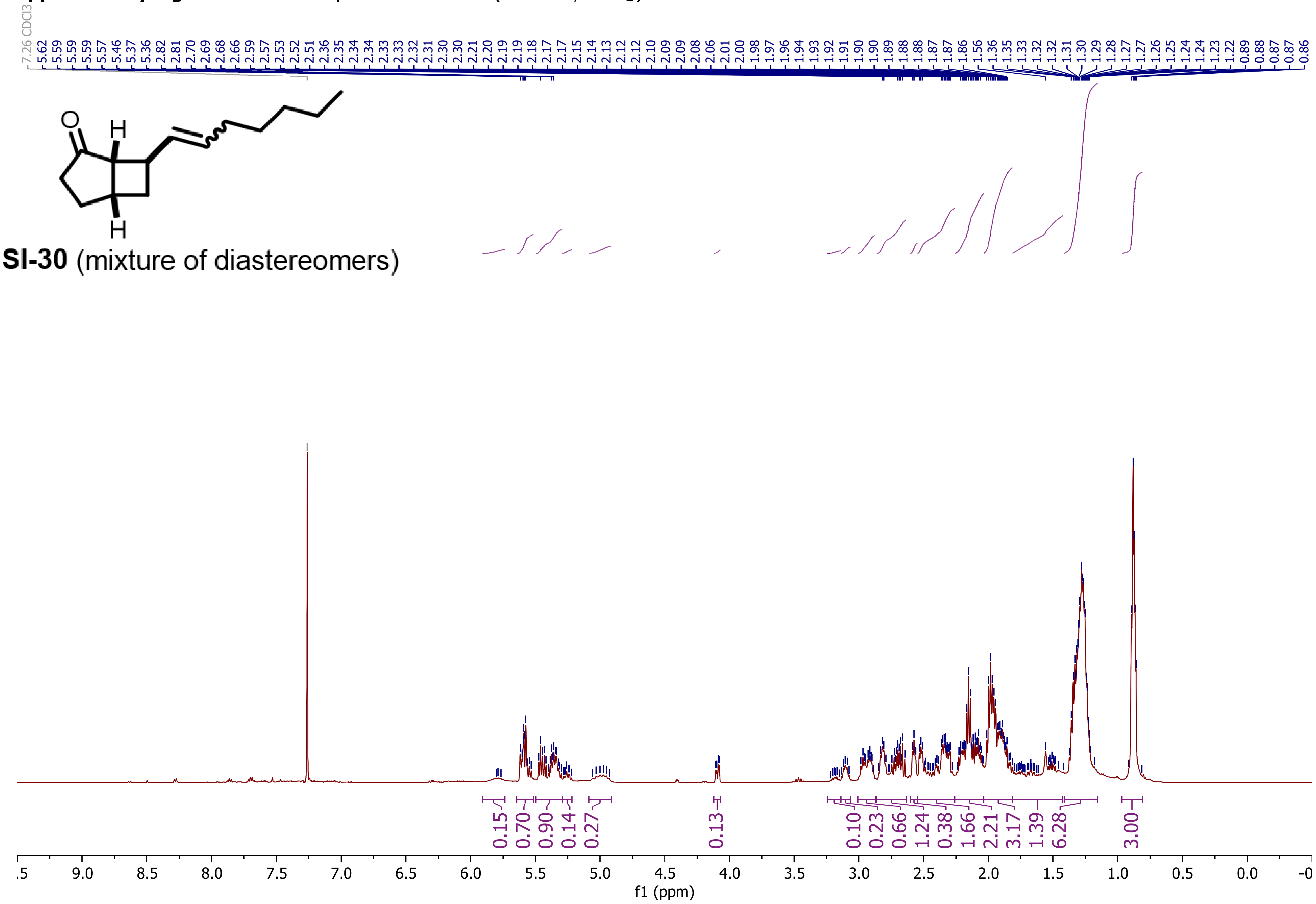

Supplementary Figure 52. ^{1}H NMR spectrum of **SI-31** (500 MHz, $CDCl_3$)

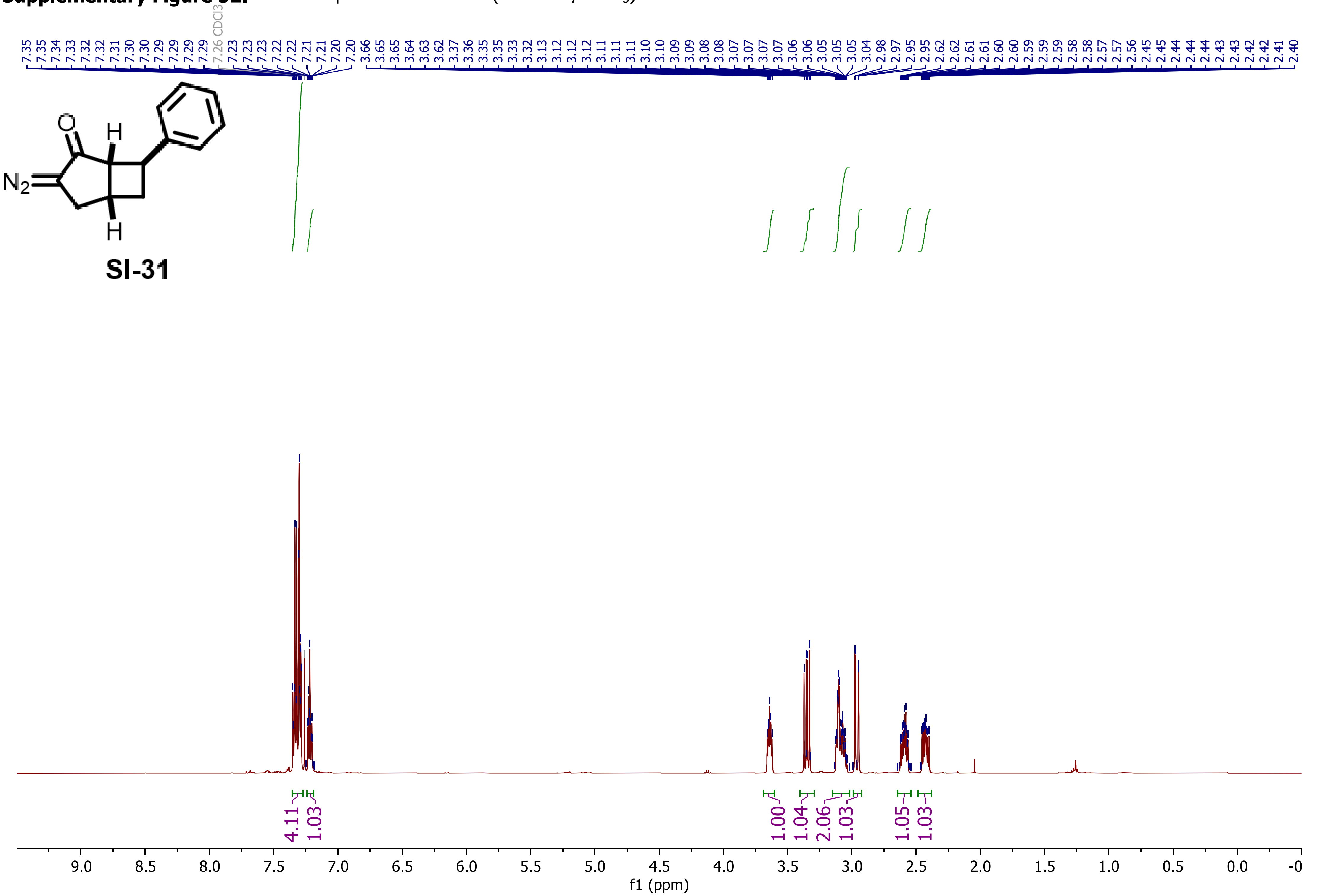

Supplementary Figure 53. ^{13}C NMR spectrum of **SI-31** (126 MHz, $CDCl_3$)

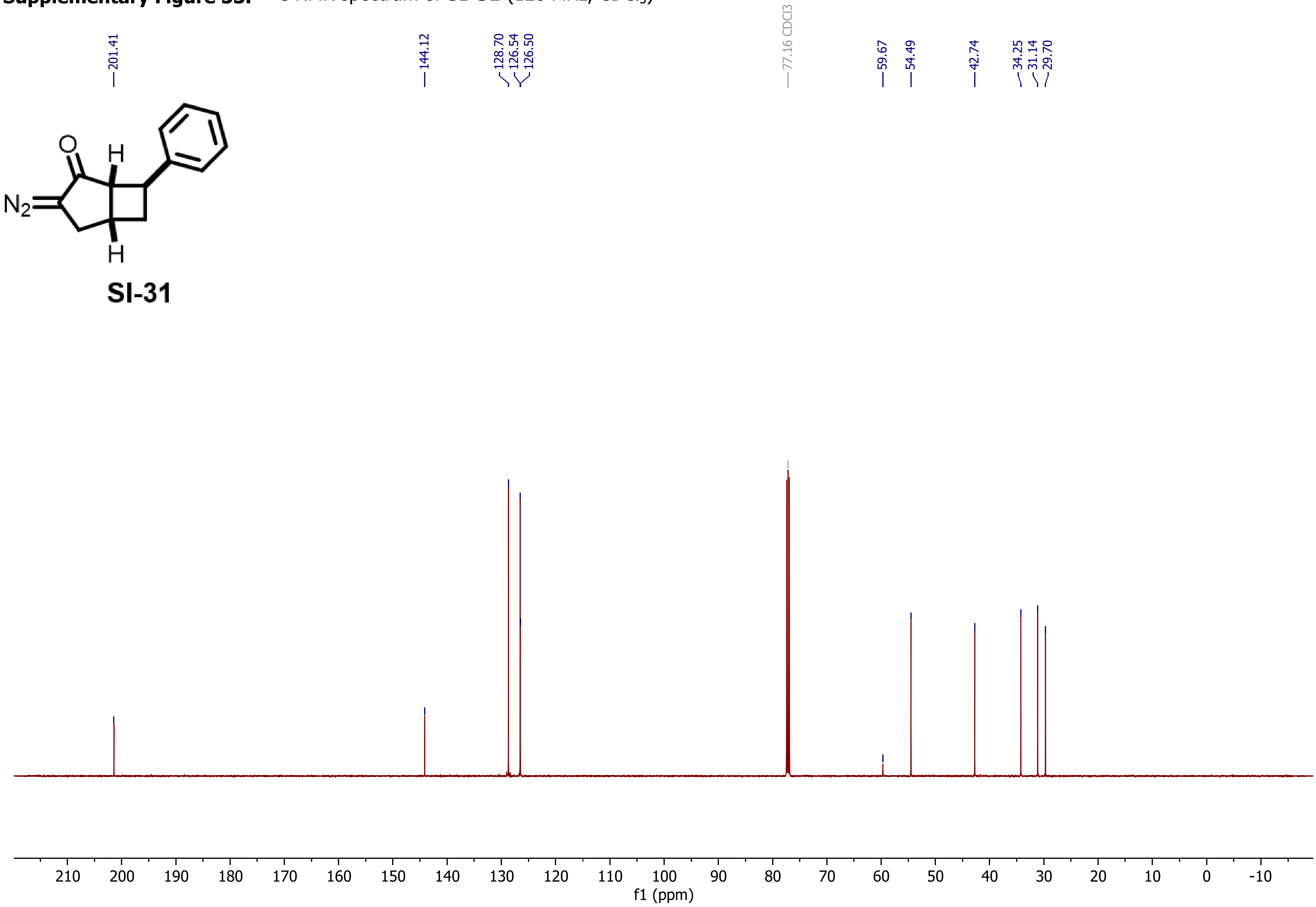

Supplementary Figure 54. ^{1}H NMR spectrum of **SI-32** (300 MHz, $CDCl_3$)

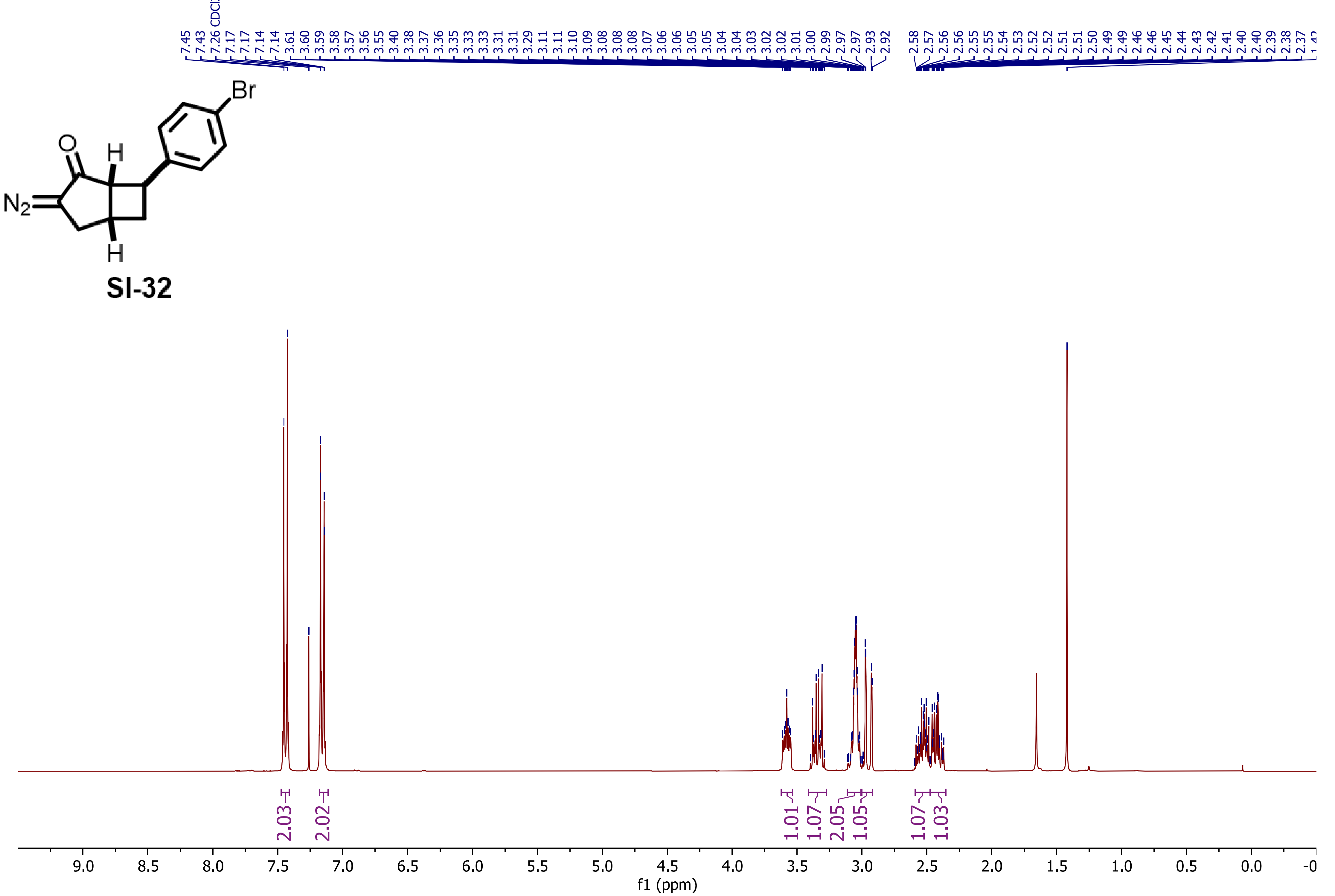

Supplementary Figure 55. ^{13}C NMR spectrum of **SI-32** (75 MHz, $CDCl_3$)

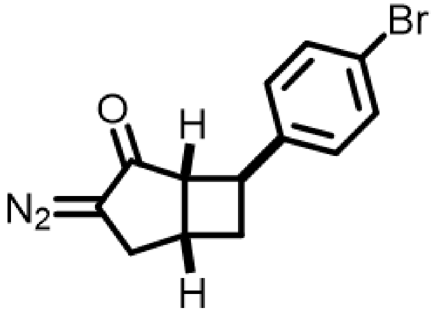

SI-32

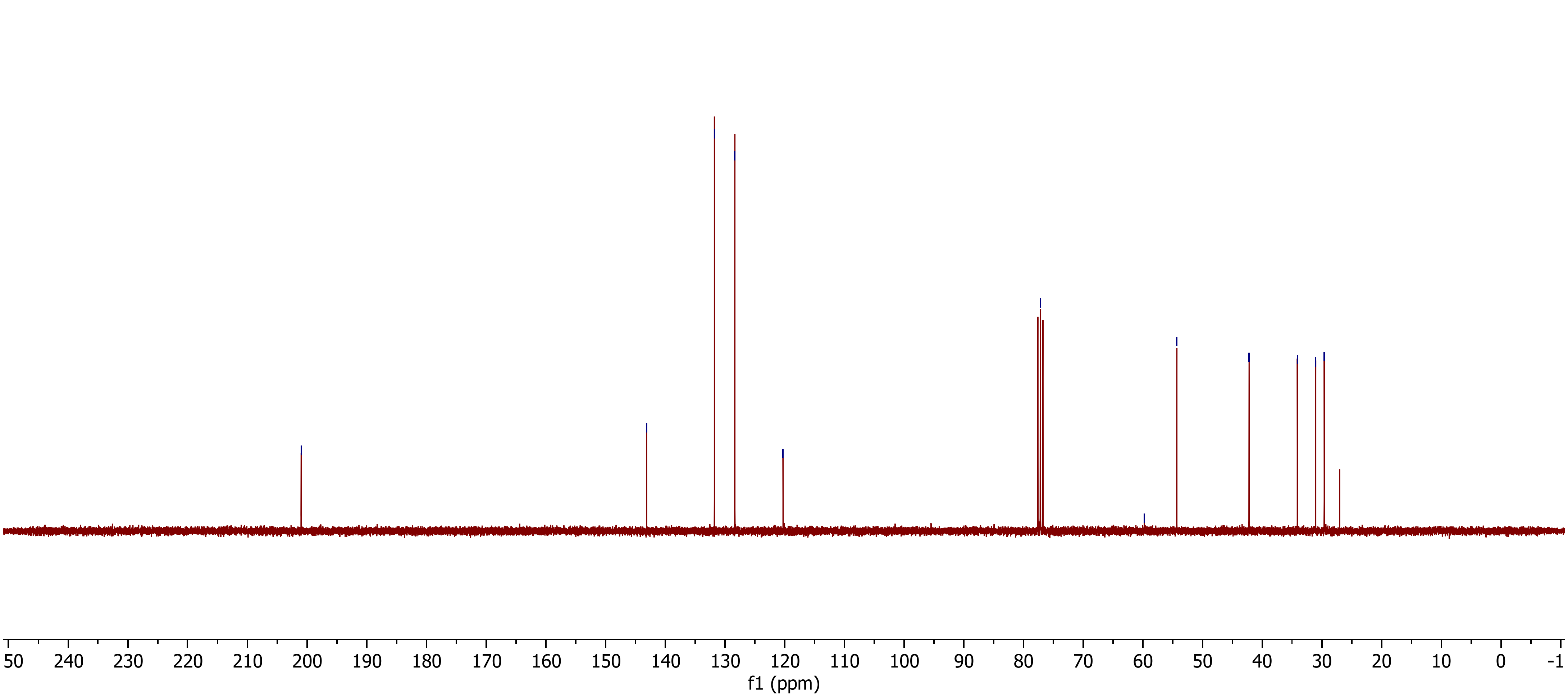

Supplementary Figure 56. ^{1}H NMR spectrum of **SI-33** (400 MHz, $CDCl_3$)

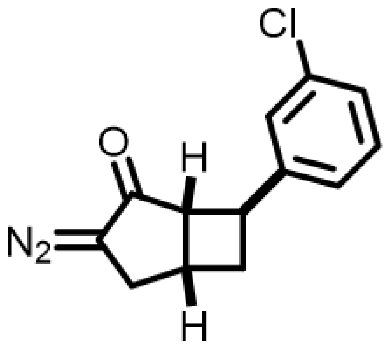

SI-33

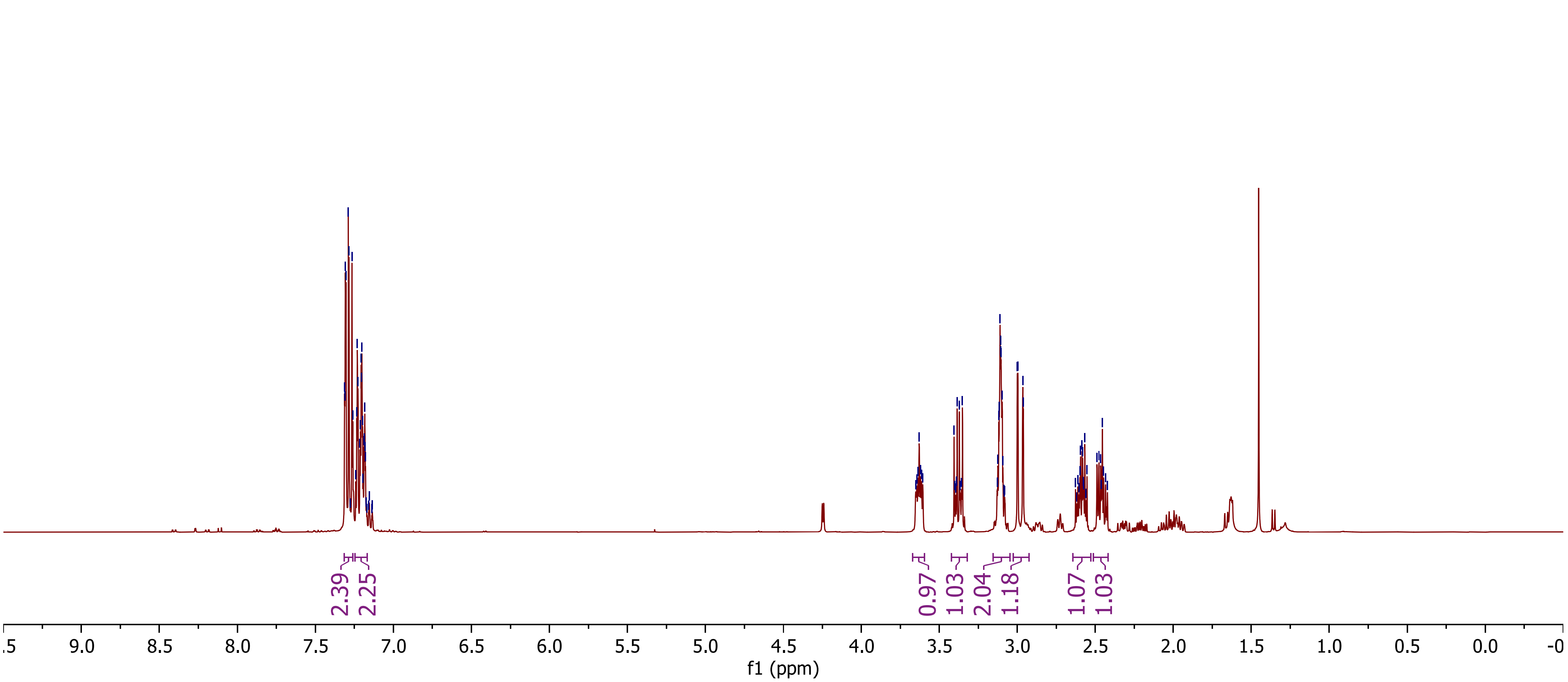

Supplementary Figure 57. ^{1}H NMR spectrum of **SI-34** (300 MHz, $CDCl_3$)

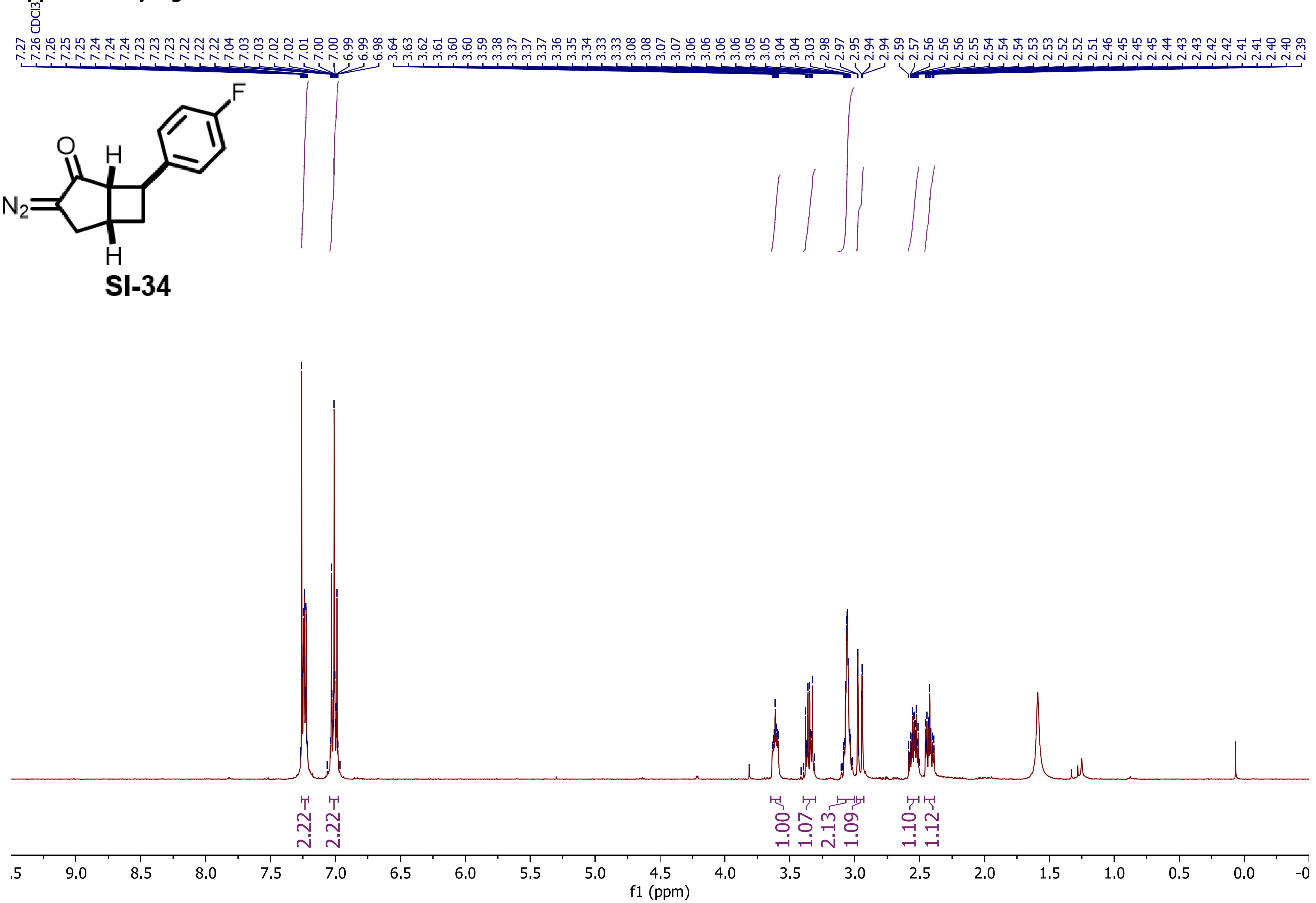

Supplementary Figure 58. ^{13}C NMR spectrum of **SI-34** (75 MHz, $CDCl_3$)

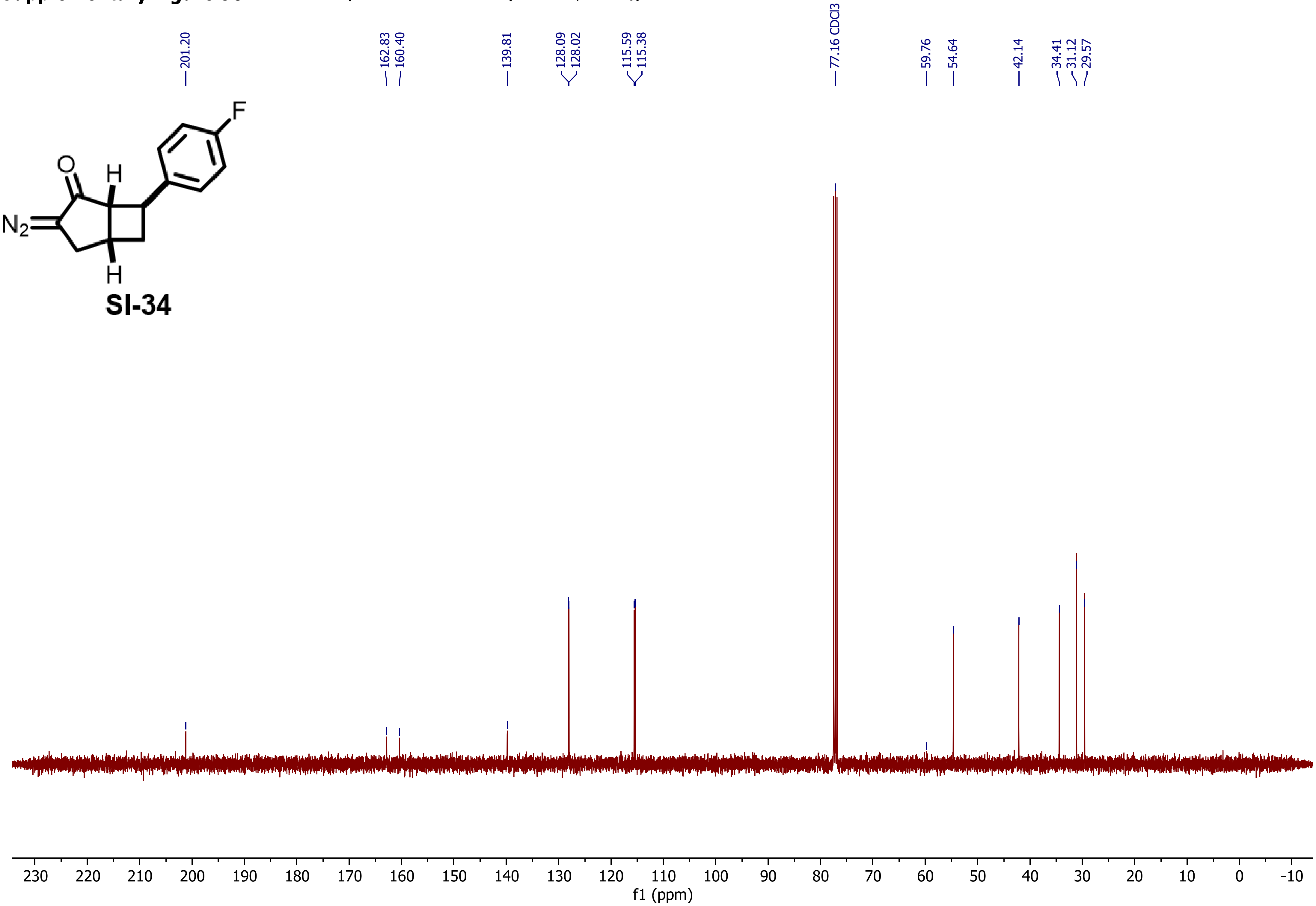

SI-34

Supplementary Figure 59. ^{1}H NMR spectrum of **SI-35** (400 MHz, $CDCl_3$)

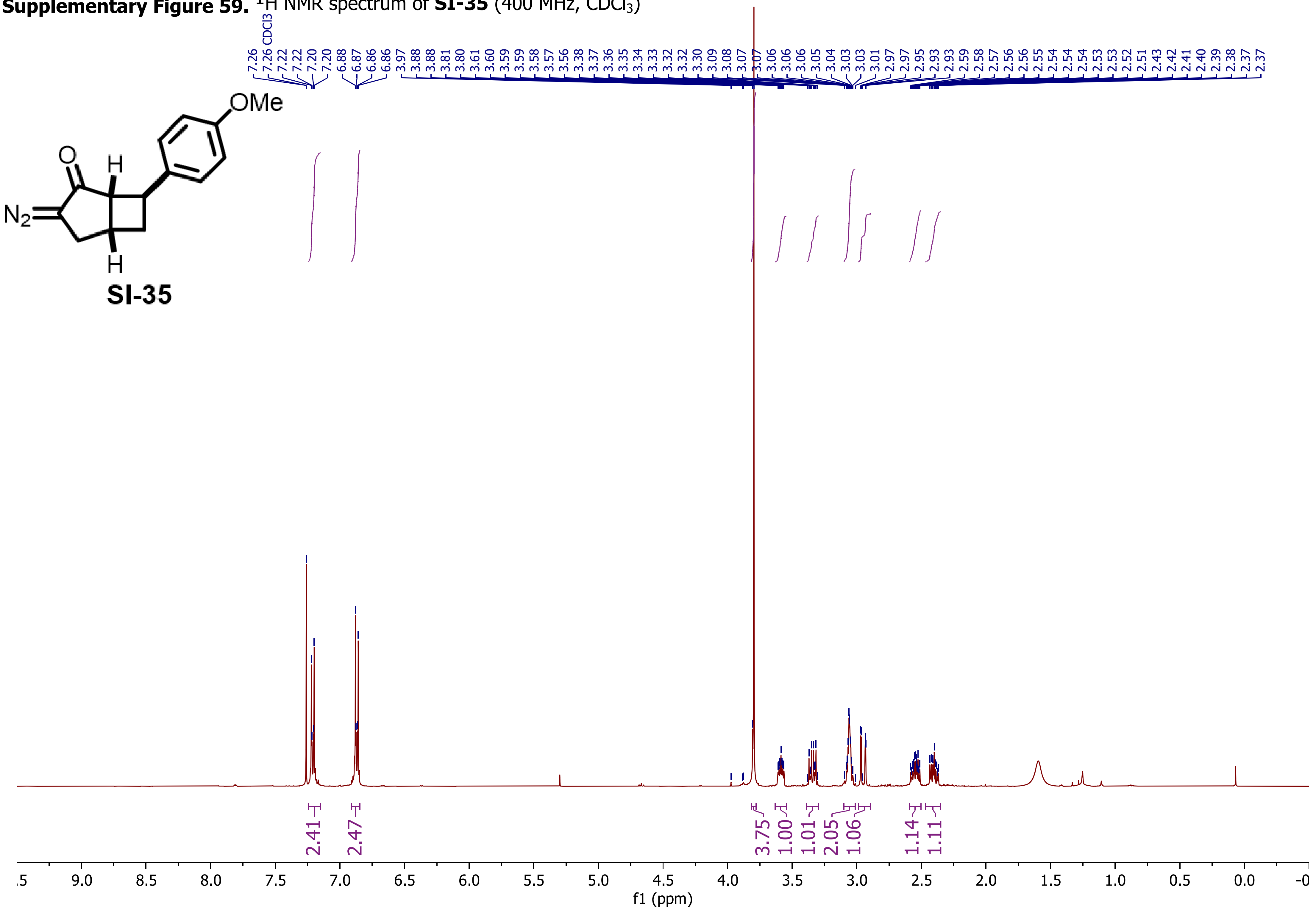

Supplementary Figure 60. ^{13}C NMR spectrum of **SI-35** (101 MHz, $CDCl_3$)

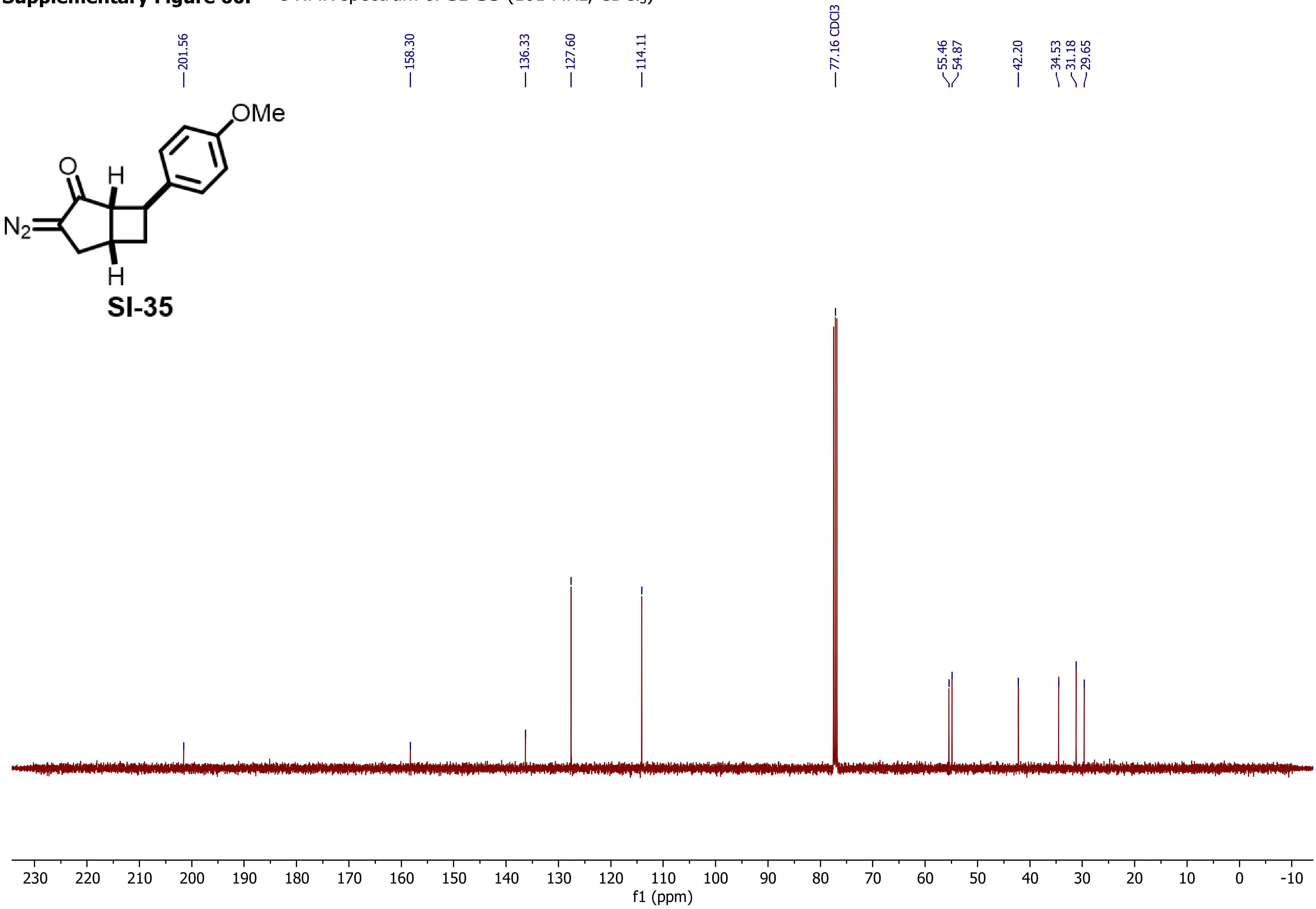

Supplementary Figure 61. ^{1}H NMR spectrum of **SI-36** (400 MHz, $CDCl_3$)

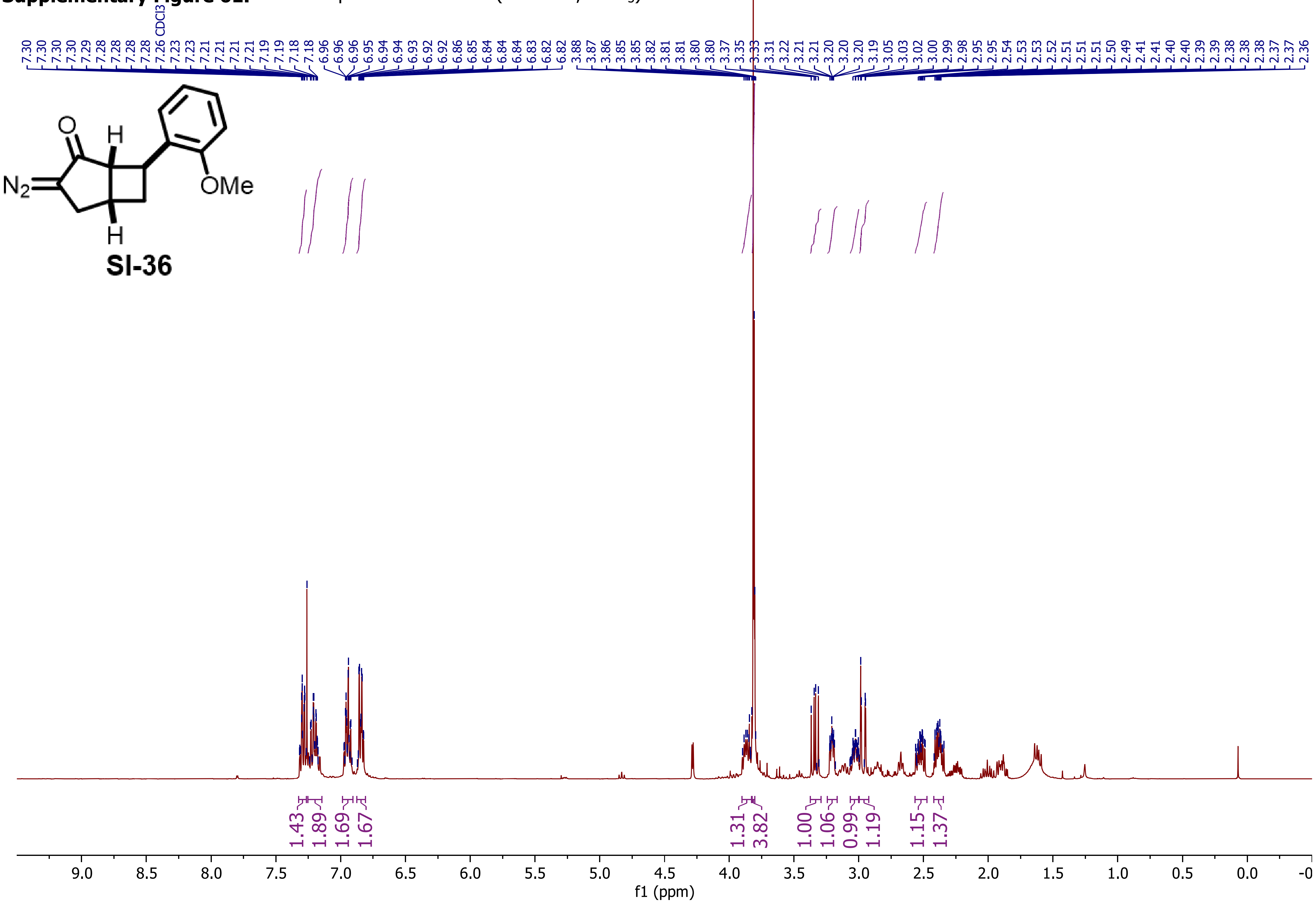

Supplementary Figure 62. ^{13}C NMR spectrum of **SI-36** (101 MHz, $CDCl_3$)

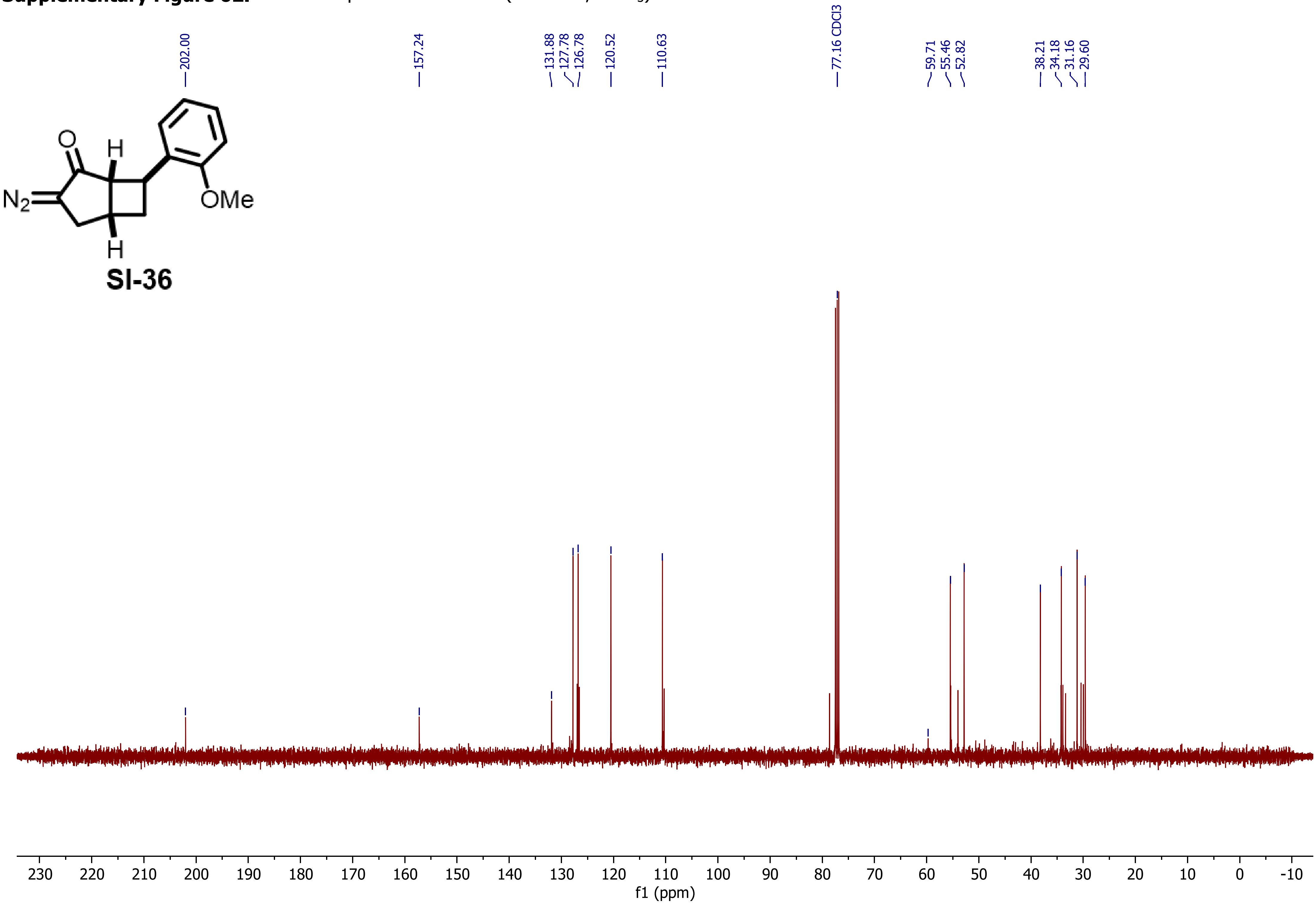

SI-36

Supplementary Figure 63. ^{1}H NMR spectrum of **SI-37** (300 MHz, $CDCl_3$)

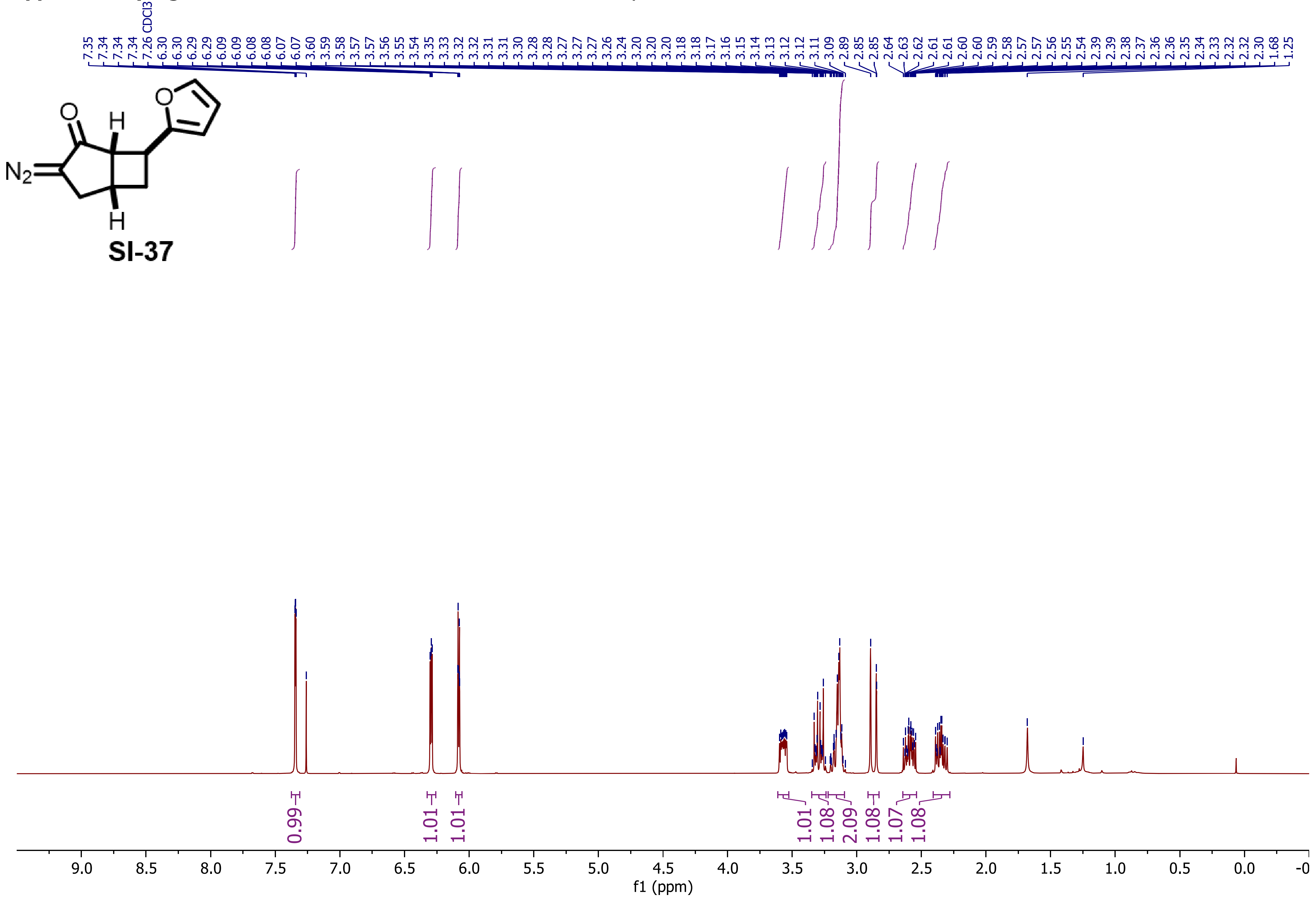

Supplementary Figure 64. ^{13}C NMR spectrum of **SI-37** (75 MHz, $CDCl_3$)

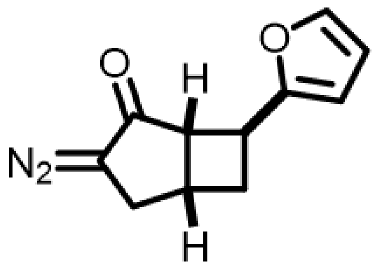

SI-37

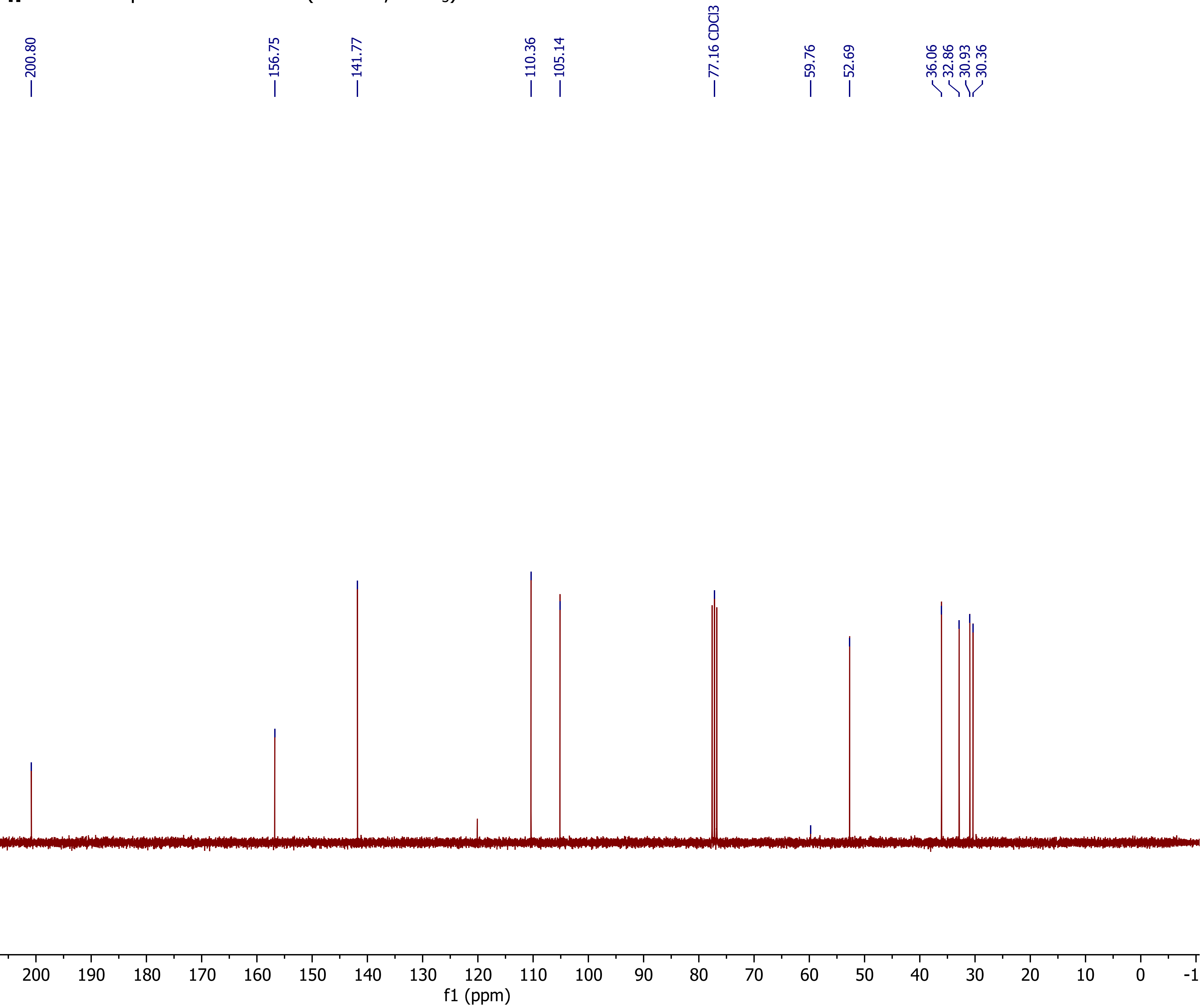

Supplementary Figure 65. ^{1}H NMR spectrum of **SI-38** (500 MHz, $CDCl_3$)

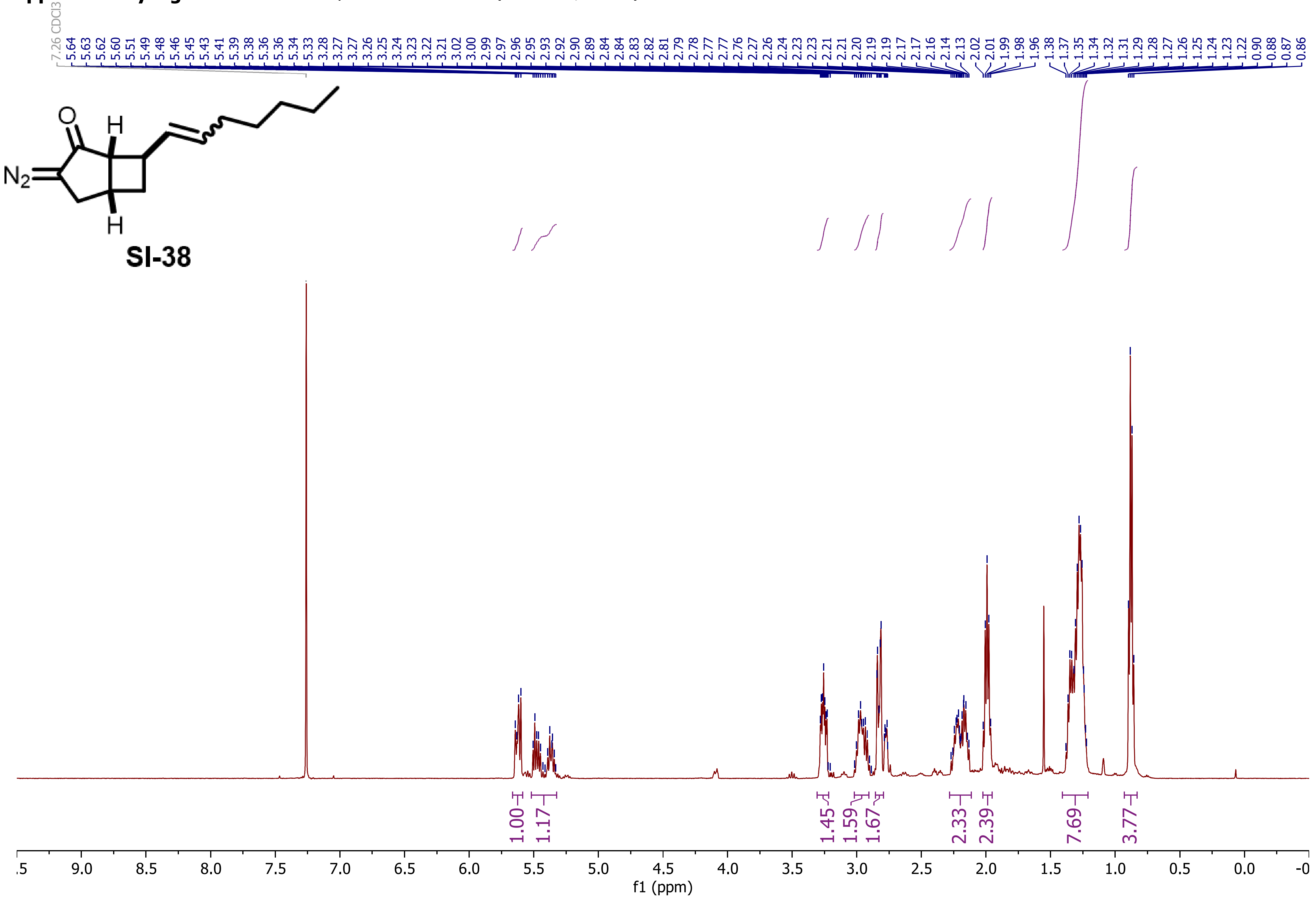

Supplementary Figure 66. ^{1}H NMR spectrum of **5a** (500 MHz, $CDCl_3$)

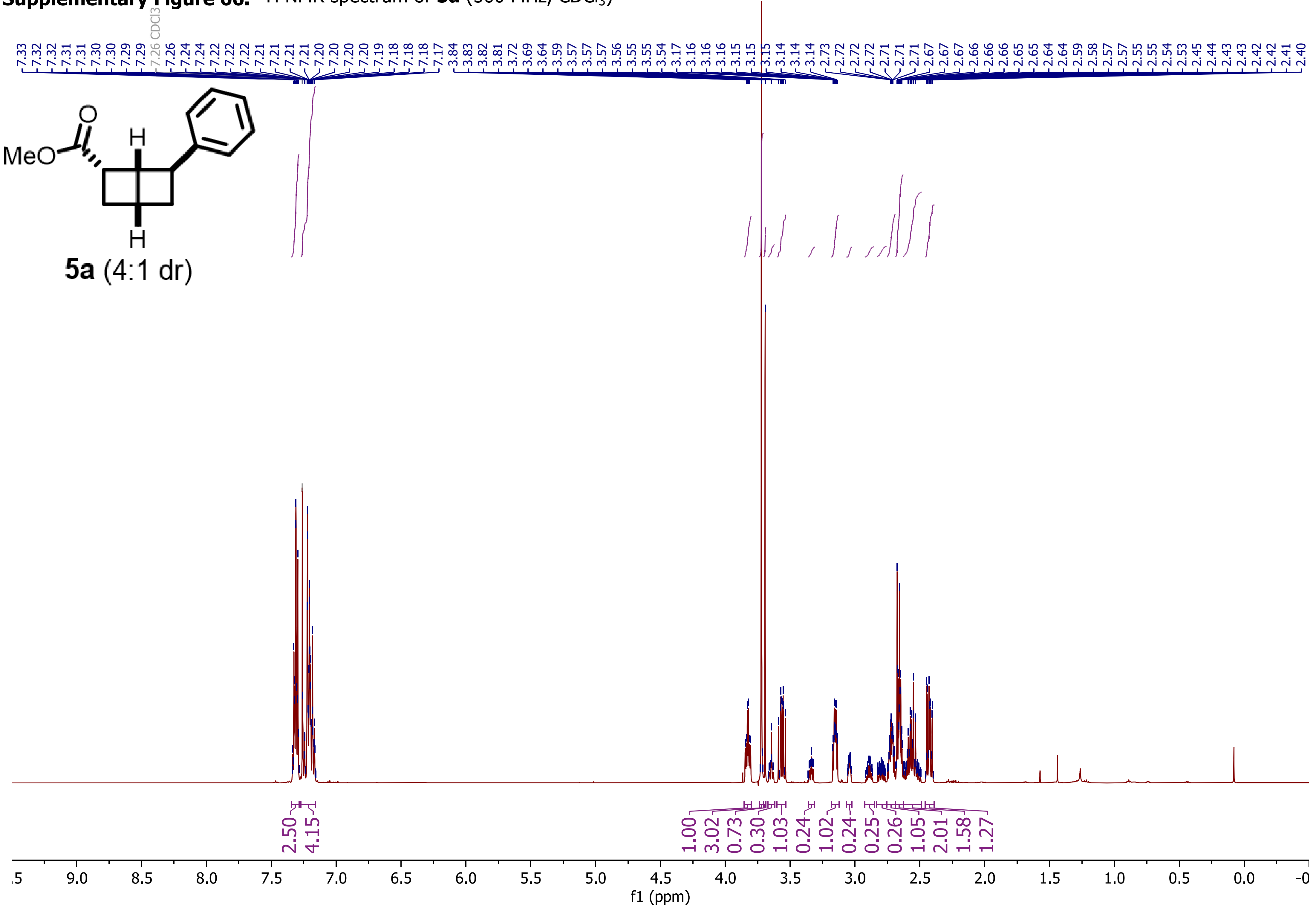

Supplementary Figure 67. ^{13}C NMR spectrum of **5a** (126 MHz, $CDCl_3$)

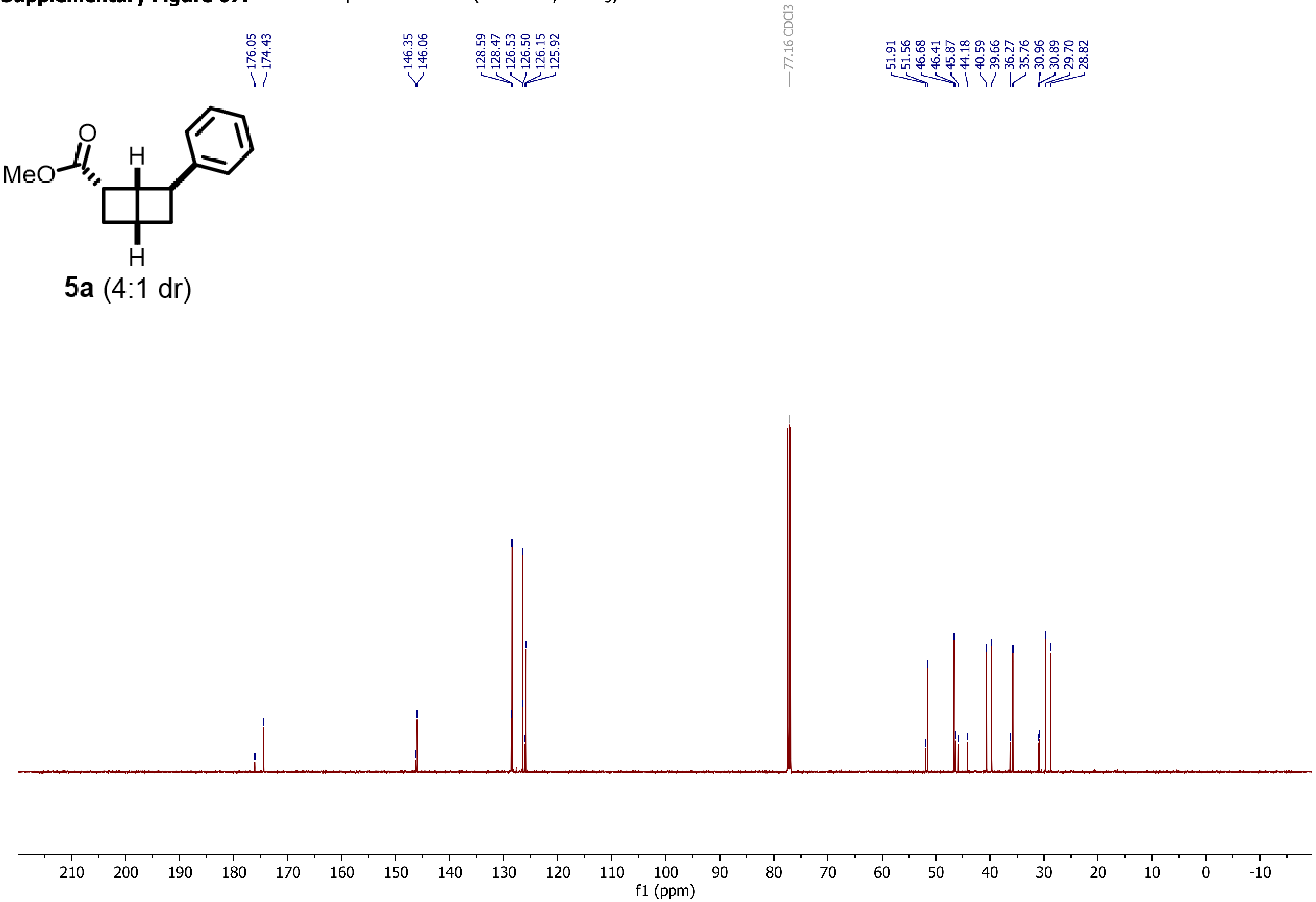

Supplementary Figure 68. ^{1}H NMR spectrum of **8** (300 MHz, $CDCl_3$)

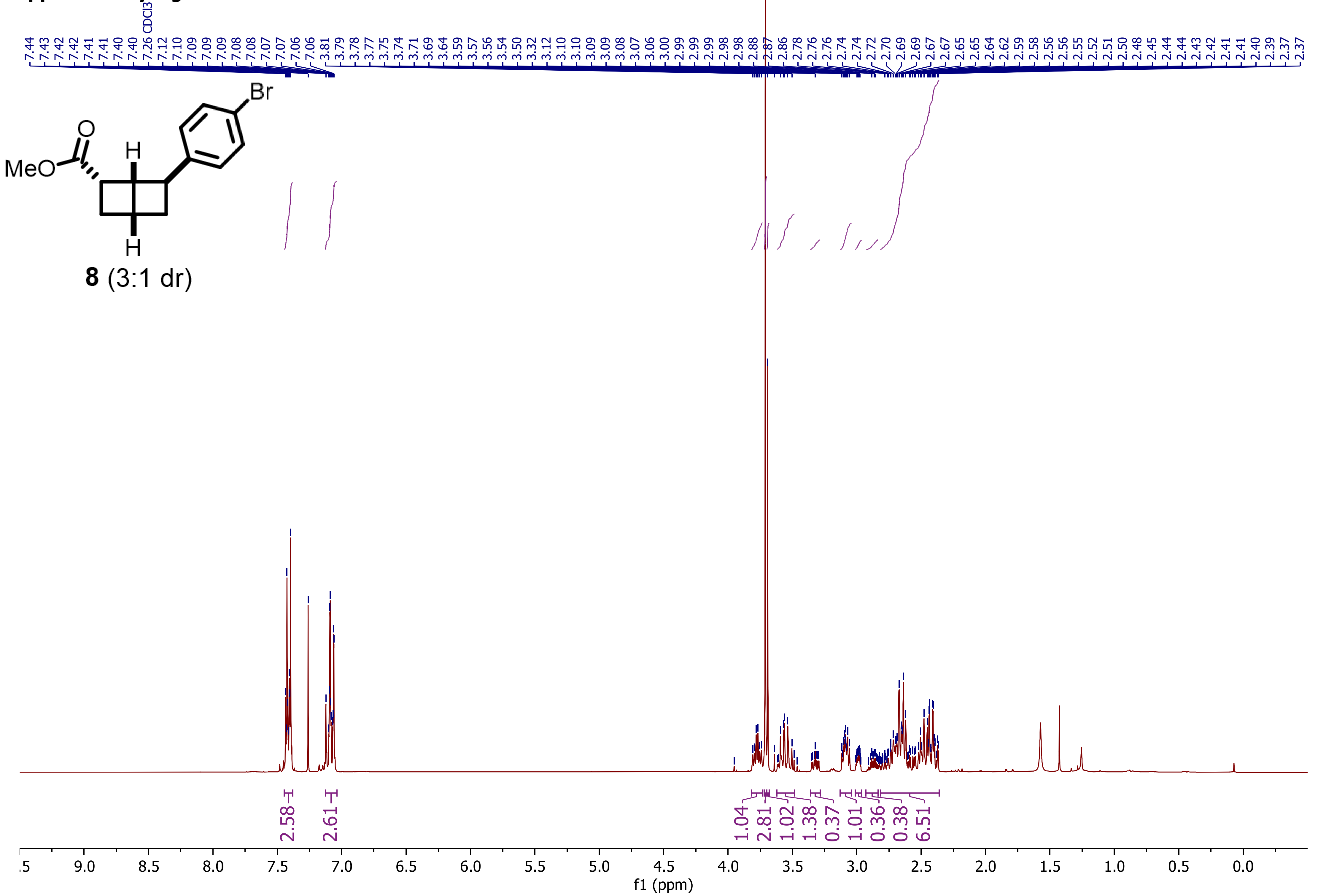

Supplementary Figure 69. ^{13}C NMR spectrum of **8** (75 MHz, $CDCl_3$)

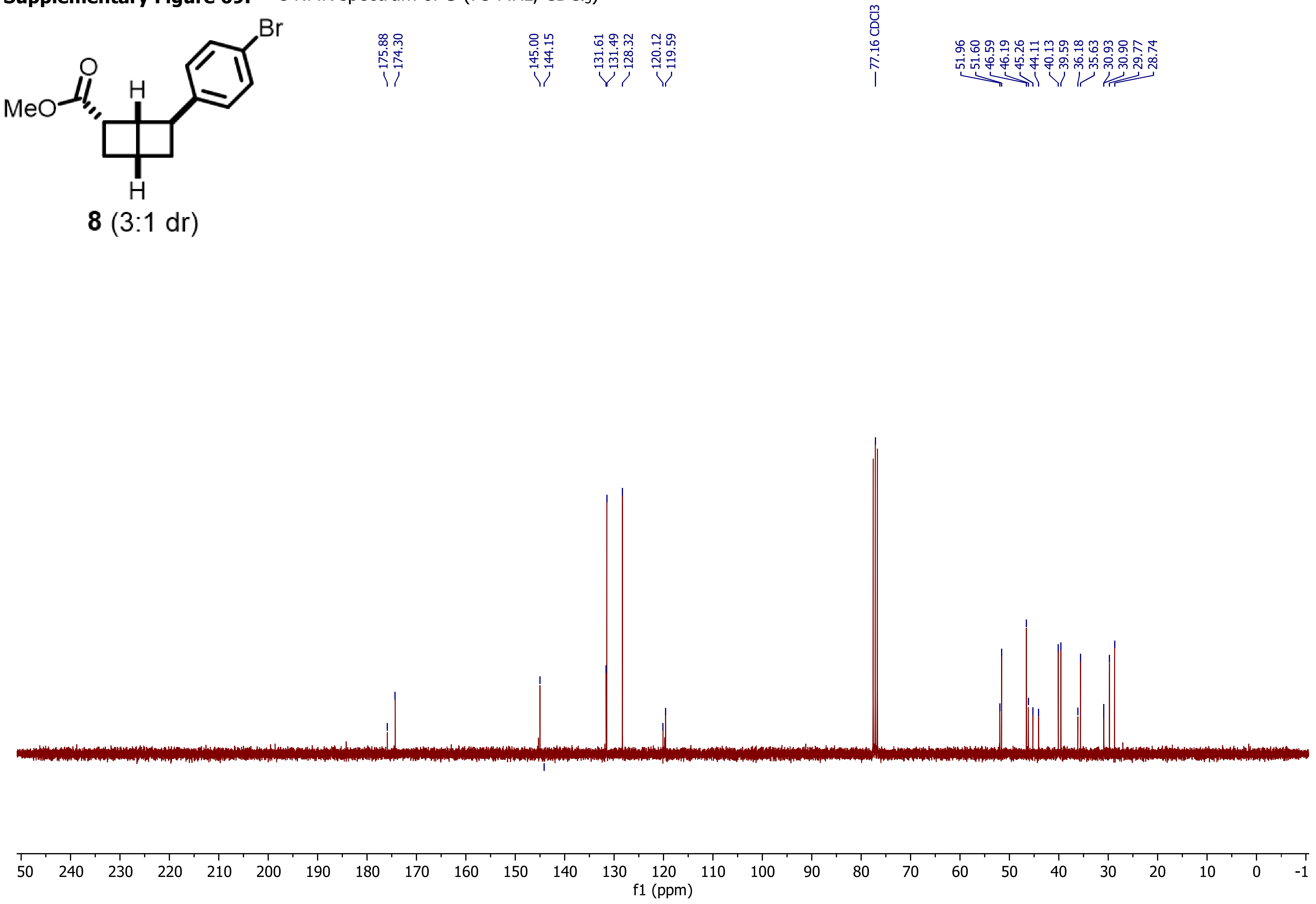

Supplementary Figure 70. ^{1}H NMR spectrum of **9** (400 MHz, $CDCl_3$)

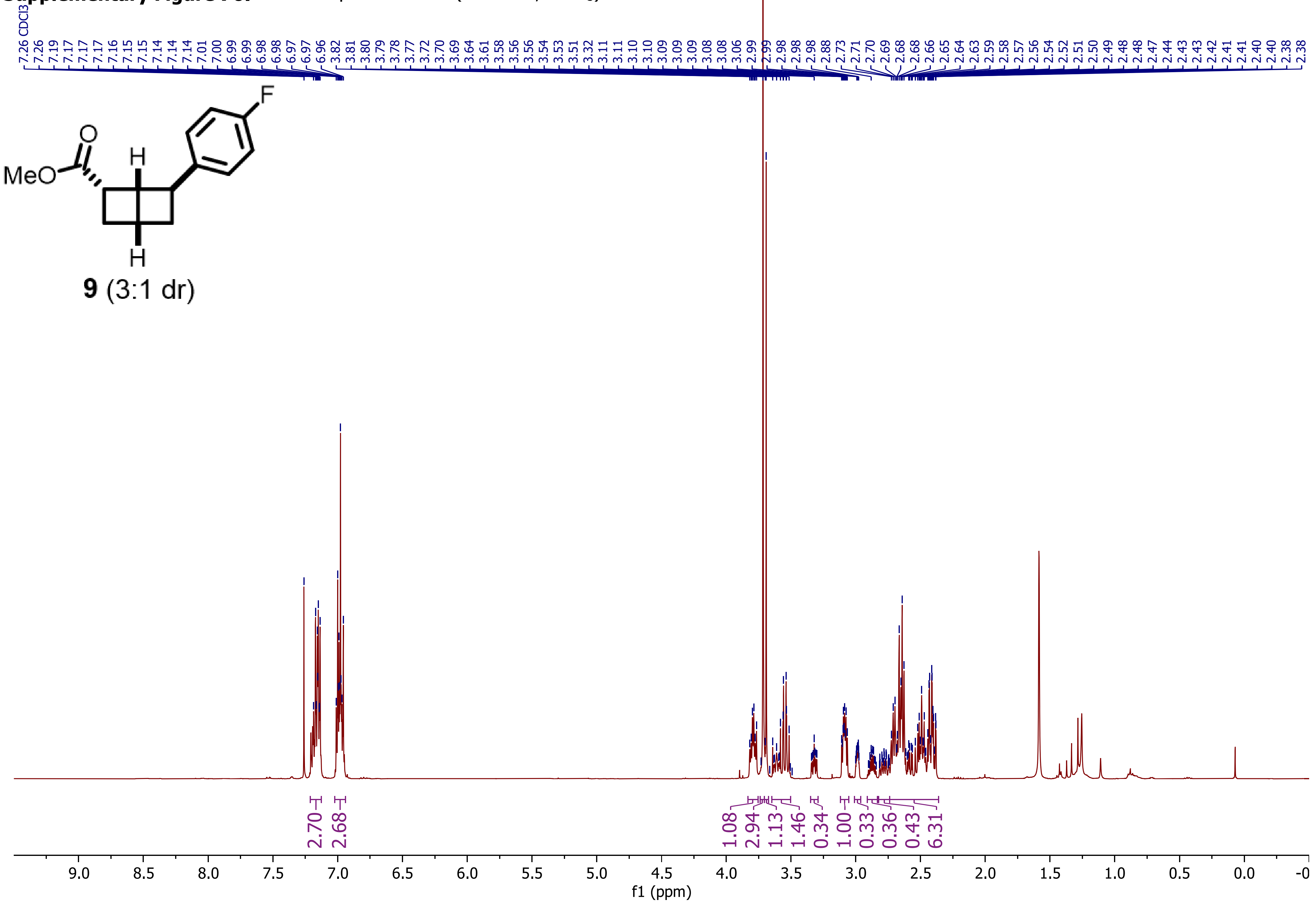

Supplementary Figure 71. ^{13}C NMR spectrum of **9** (101 MHz, $CDCl_3$)

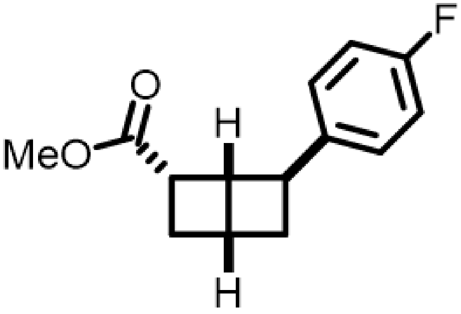

9 (3:1 dr)

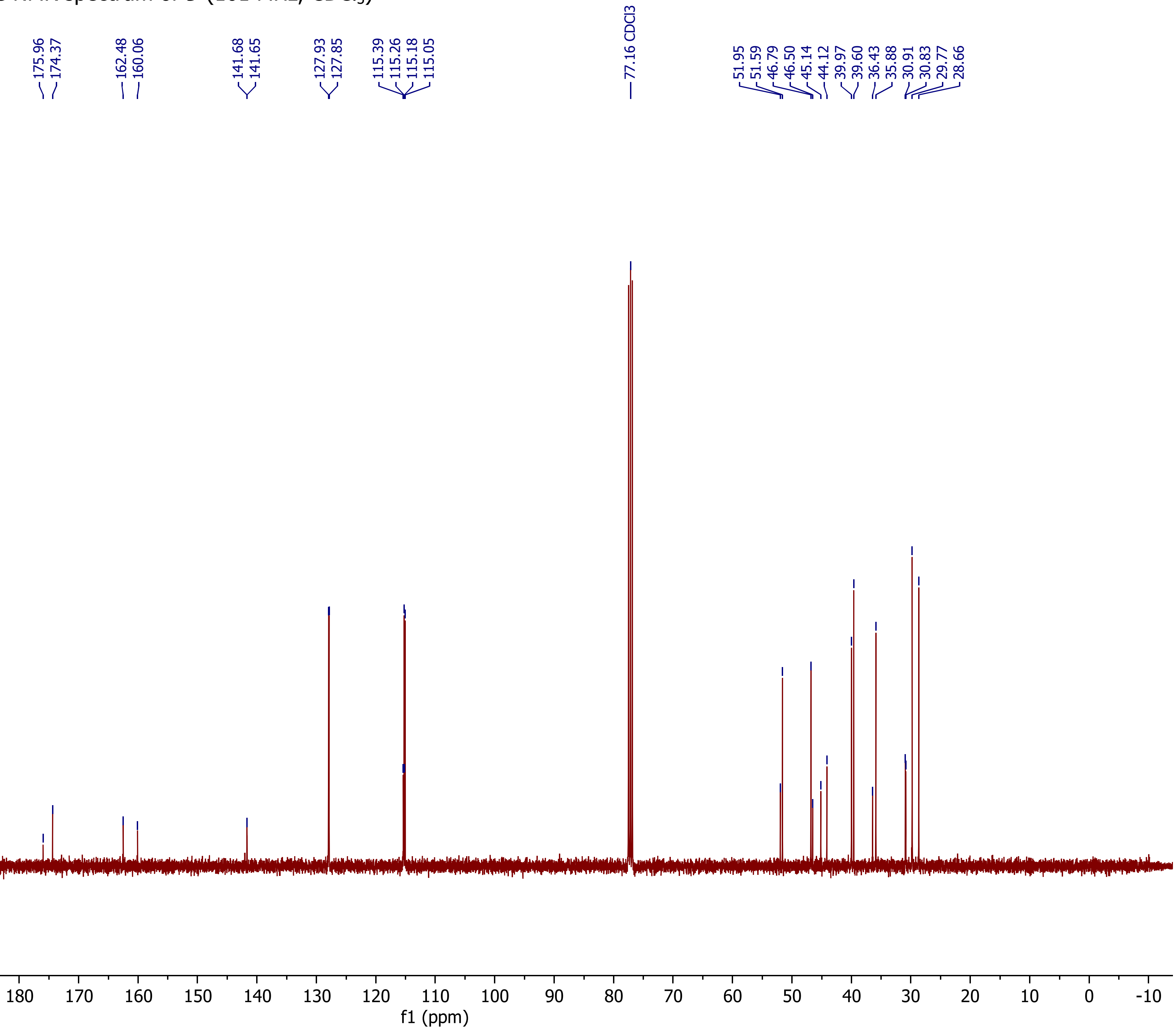

Supplementary Figure 72. ^{1}H NMR spectrum of **10** (400 MHz, $CDCl_3$)

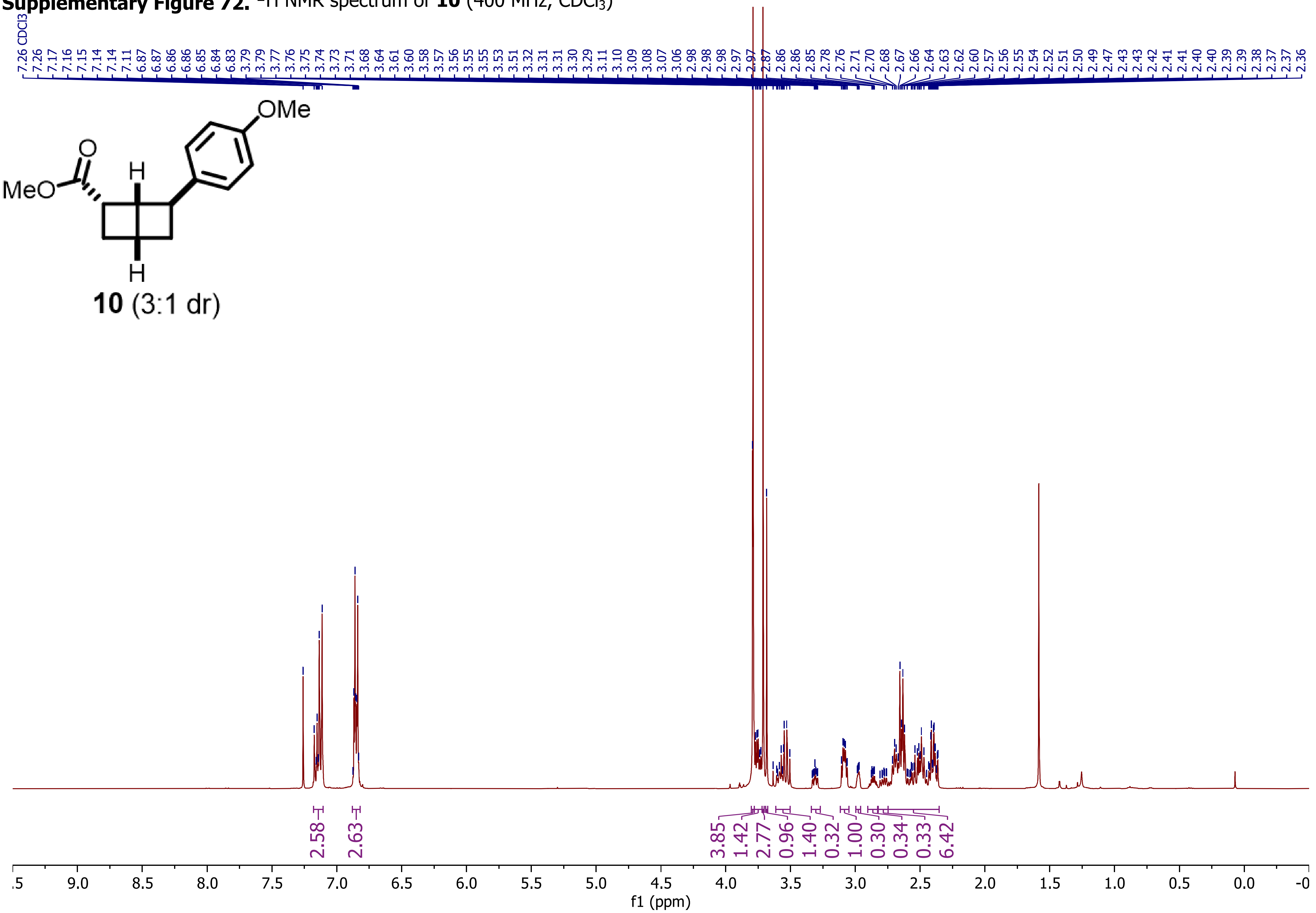

Supplementary Figure 73. ^{13}C NMR spectrum of **10** (101 MHz, $CDCl_3$)

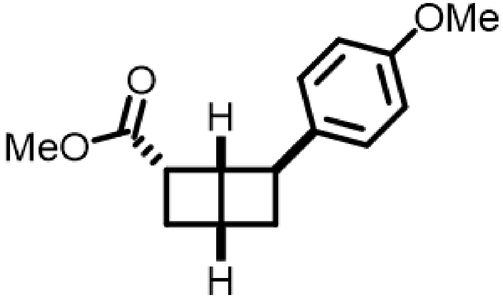

10 (3:1 dr)

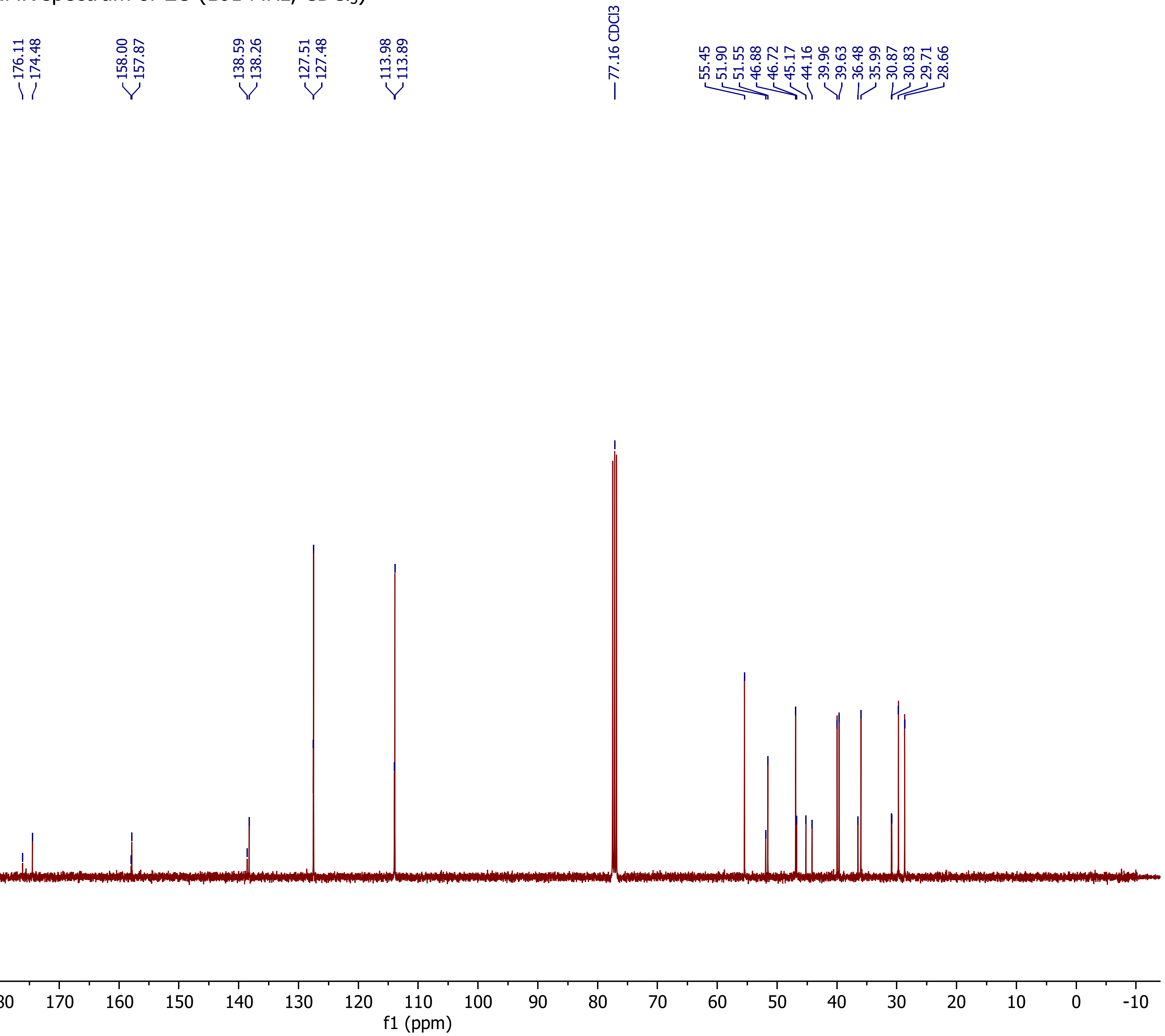

Supplementary Figure 74. ^{1}H NMR spectrum of **11** (400 MHz, $CDCl_3$)

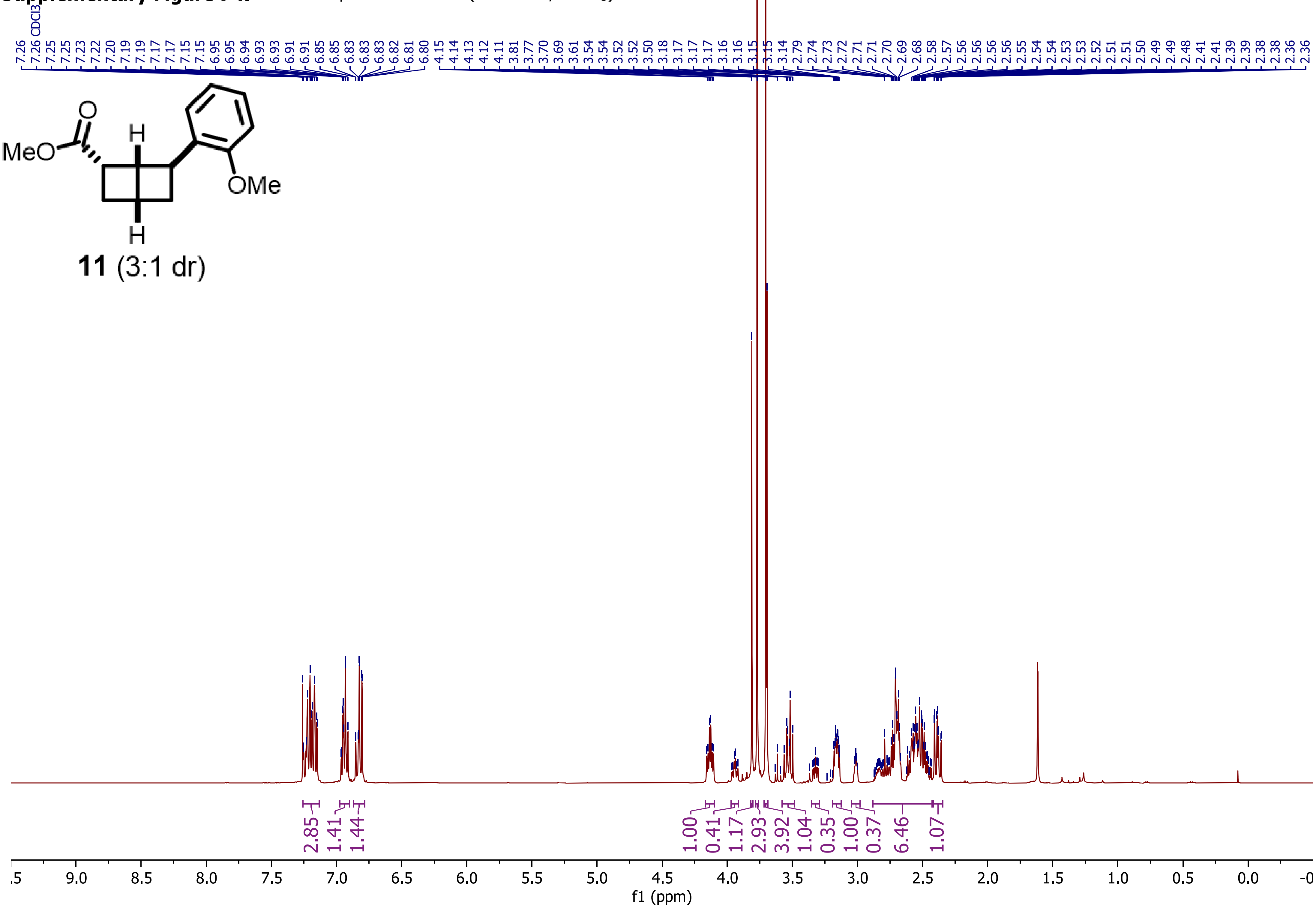

Supplementary Figure 75. ^{13}C NMR spectrum of **11** (101 MHz, $CDCl_3$)

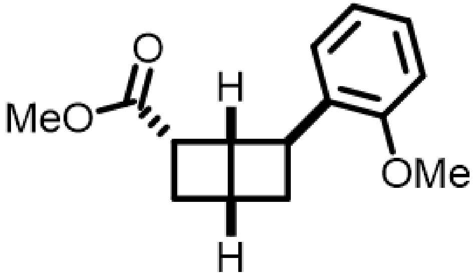

11 (3:1 dr)

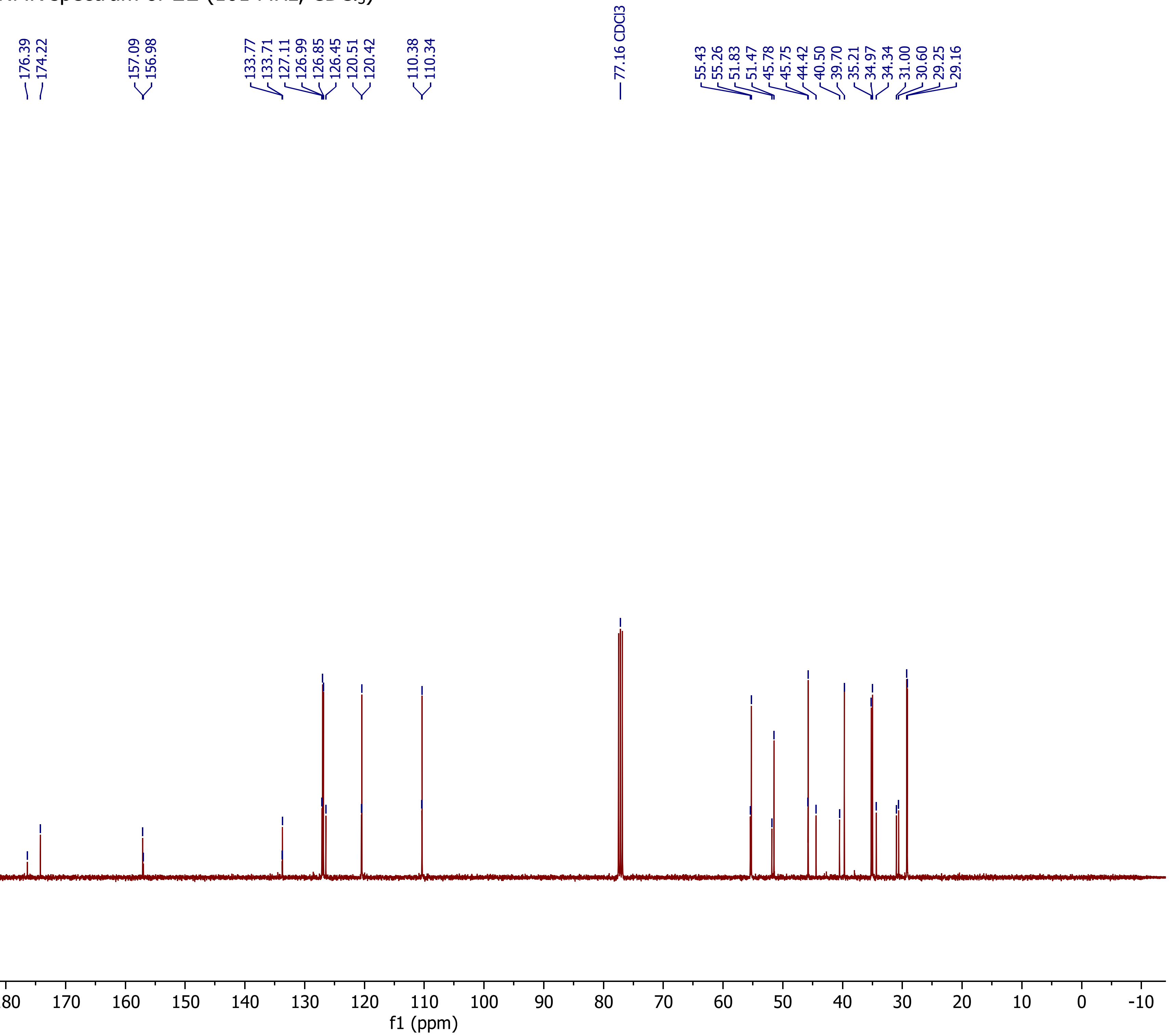

Supplementary Figure 76. ^{1}H NMR spectrum of **12** (400 MHz, $CDCl_3$)

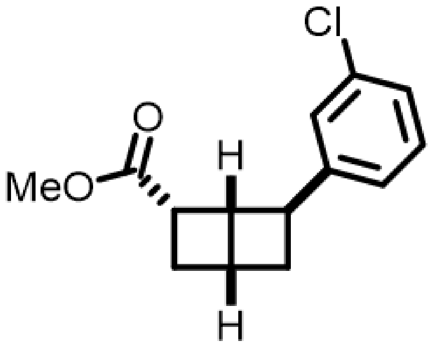

12 (4:1 dr)

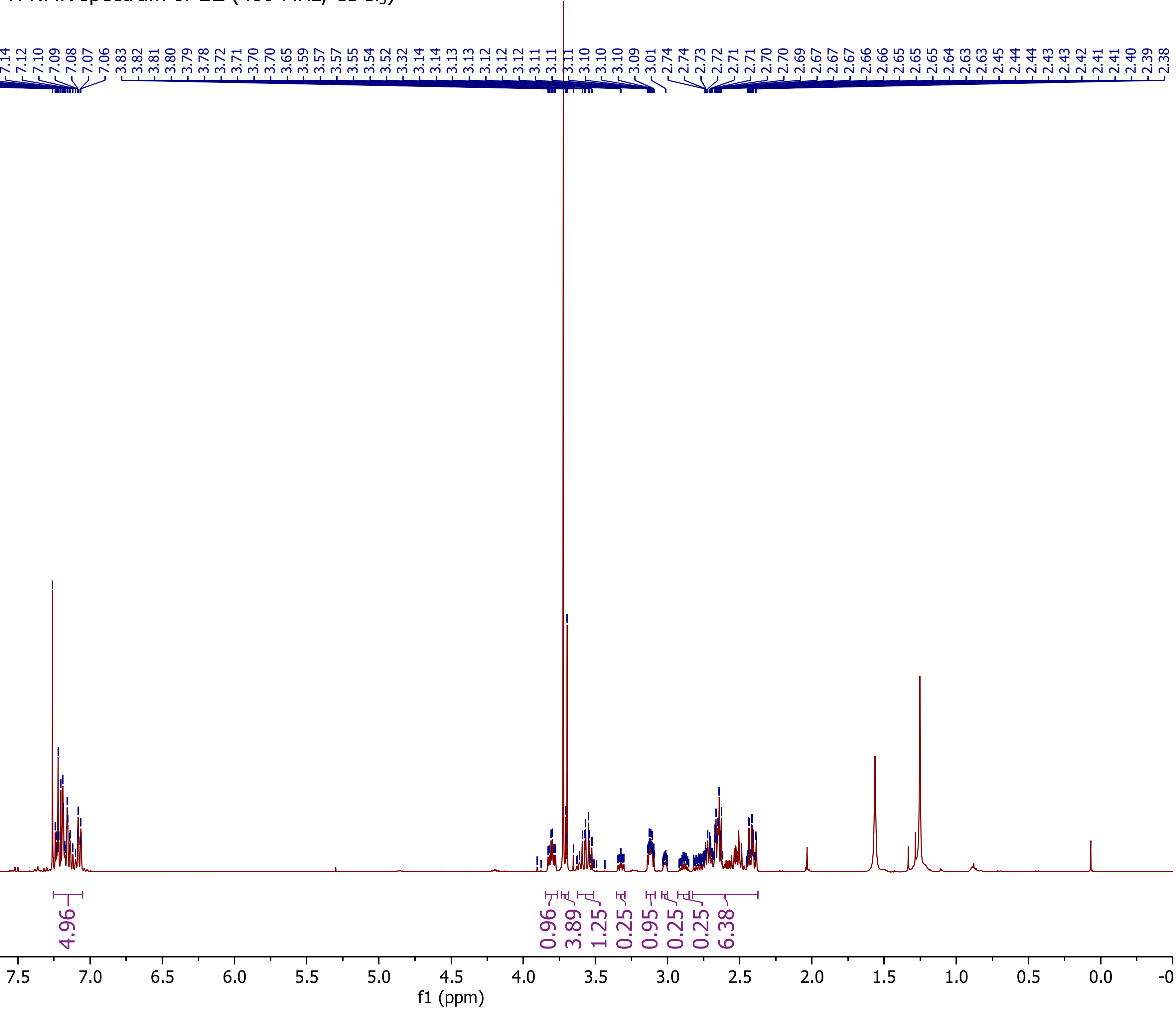

Supplementary Figure 77. ^{13}C NMR spectrum of **12** (101 MHz, $CDCl_3$)

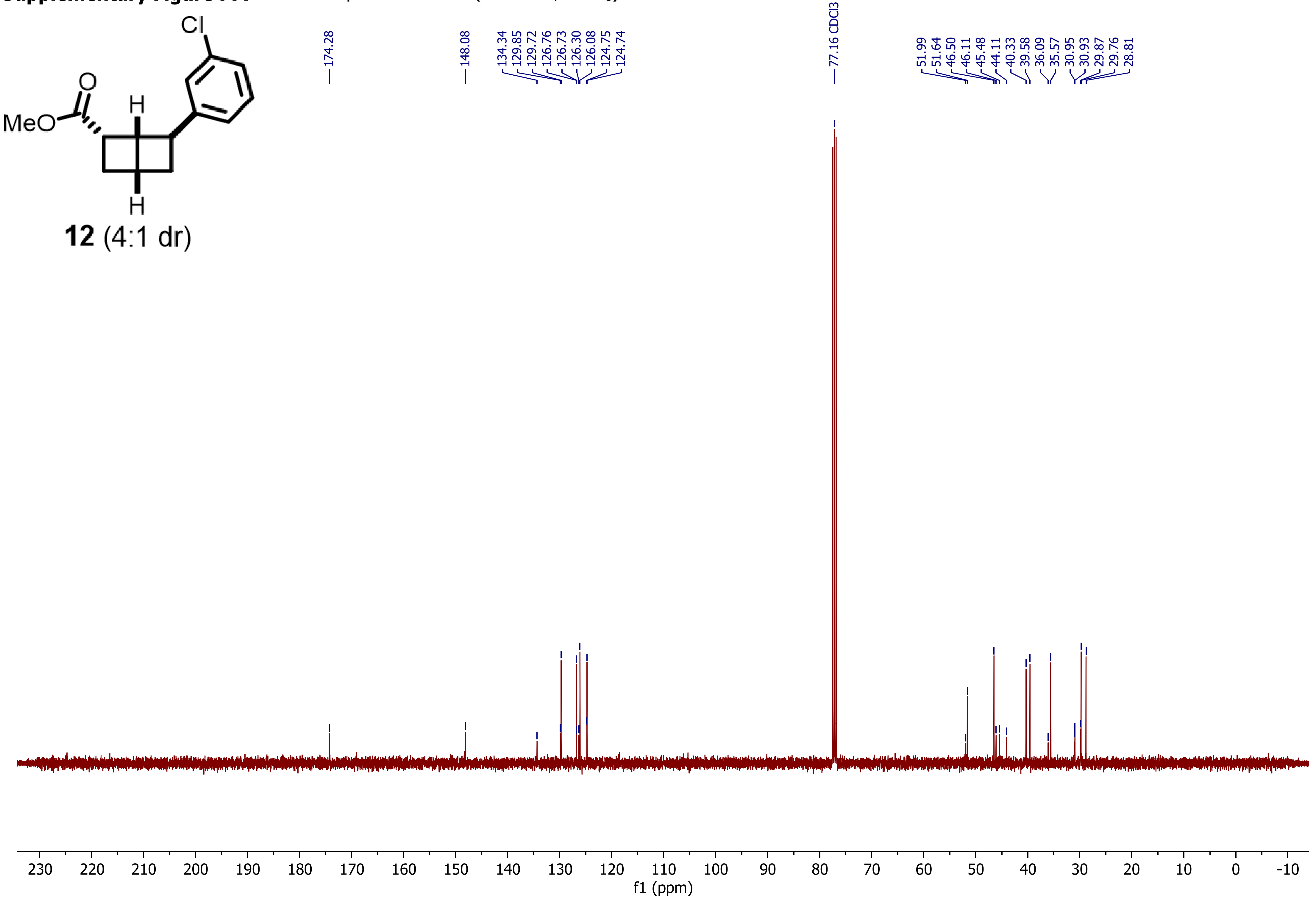

Supplementary Figure 78. ^{1}H NMR spectrum of **13** (400 MHz, $CDCl_3$)

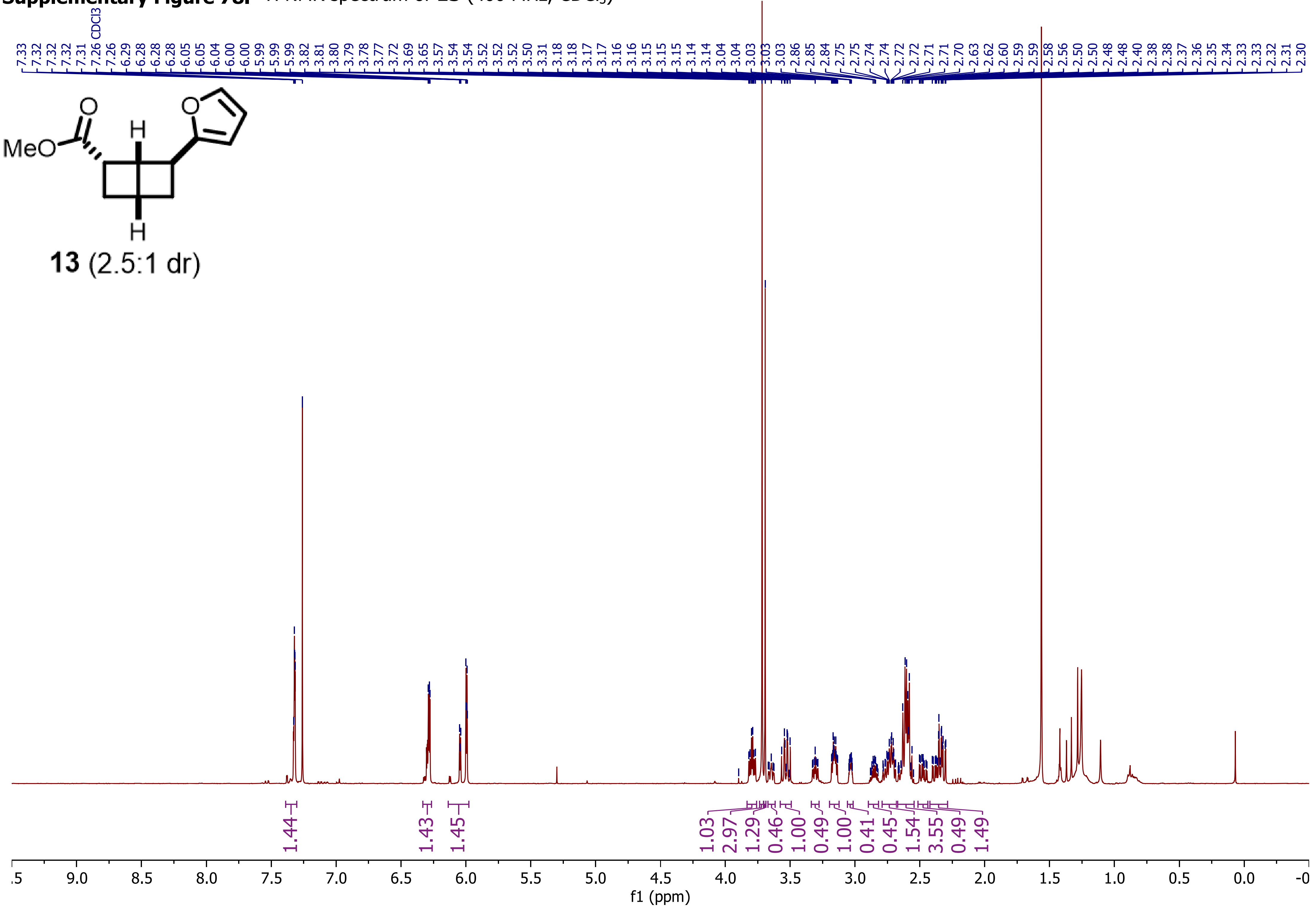

Supplementary Figure 79. ^{13}C NMR spectrum of **13** (101 MHz, $CDCl_3$)

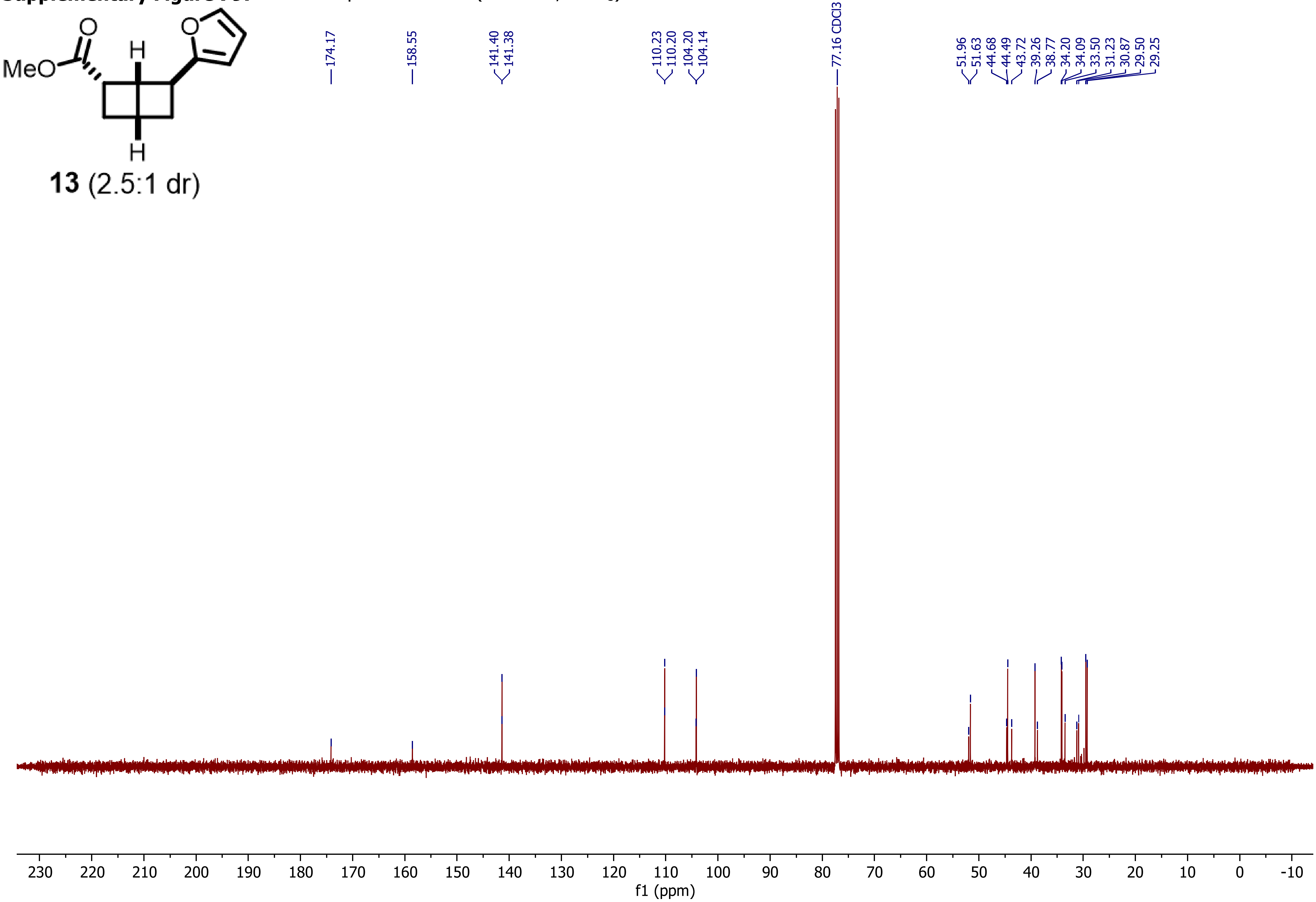

Supplementary Figure 80. ^{1}H NMR spectrum of **5b** (500 MHz, $CDCl_3$)

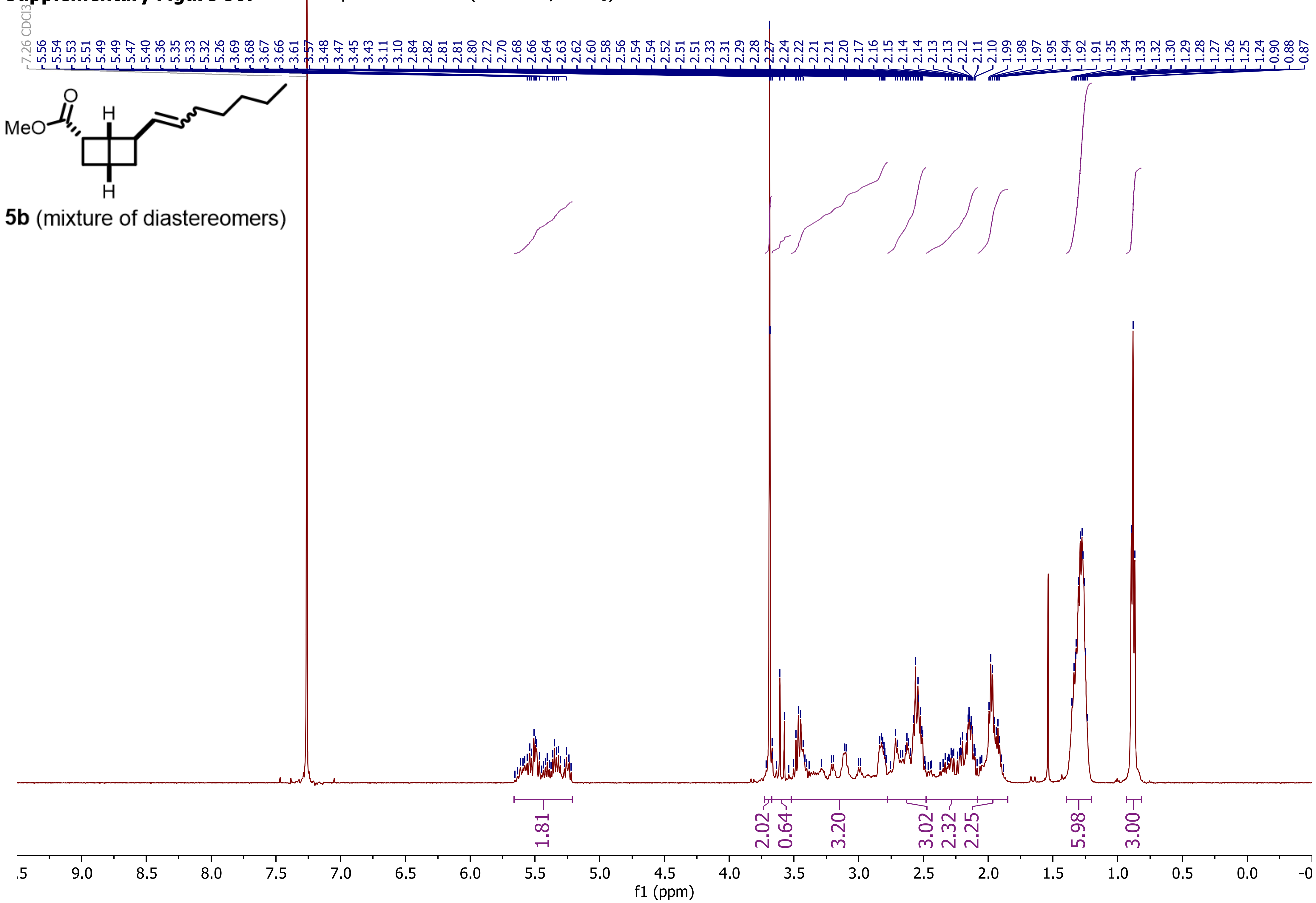

Supplementary Figure 81. ^{1}H NMR spectrum of **14** (500 MHz, $CDCl_3$)

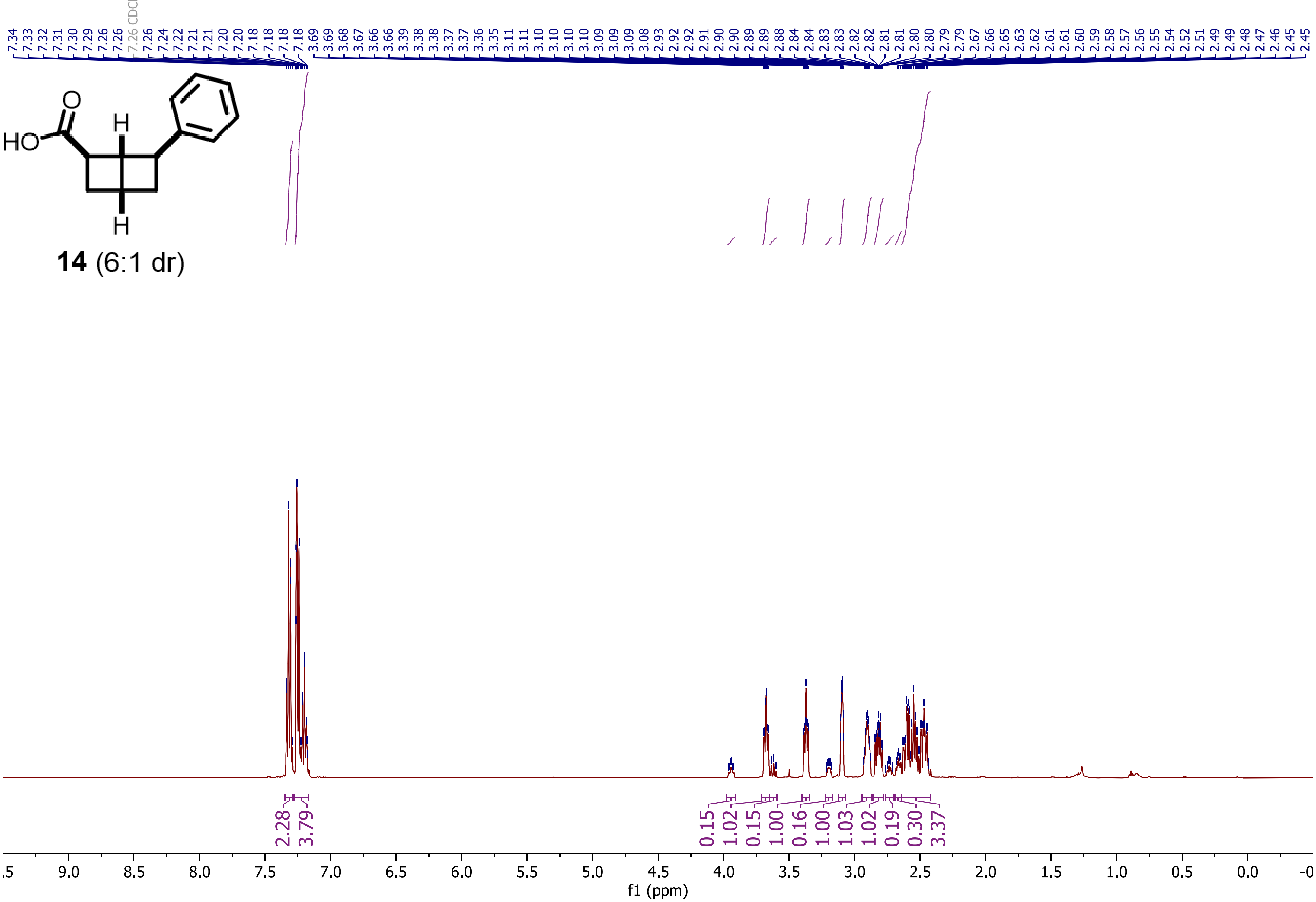

Supplementary Figure 82. ^{13}C NMR spectrum of **14** (126 MHz, $CDCl_3$)

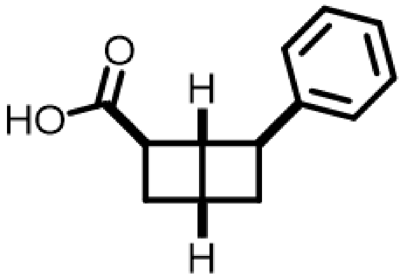

14 (6:1 dr)

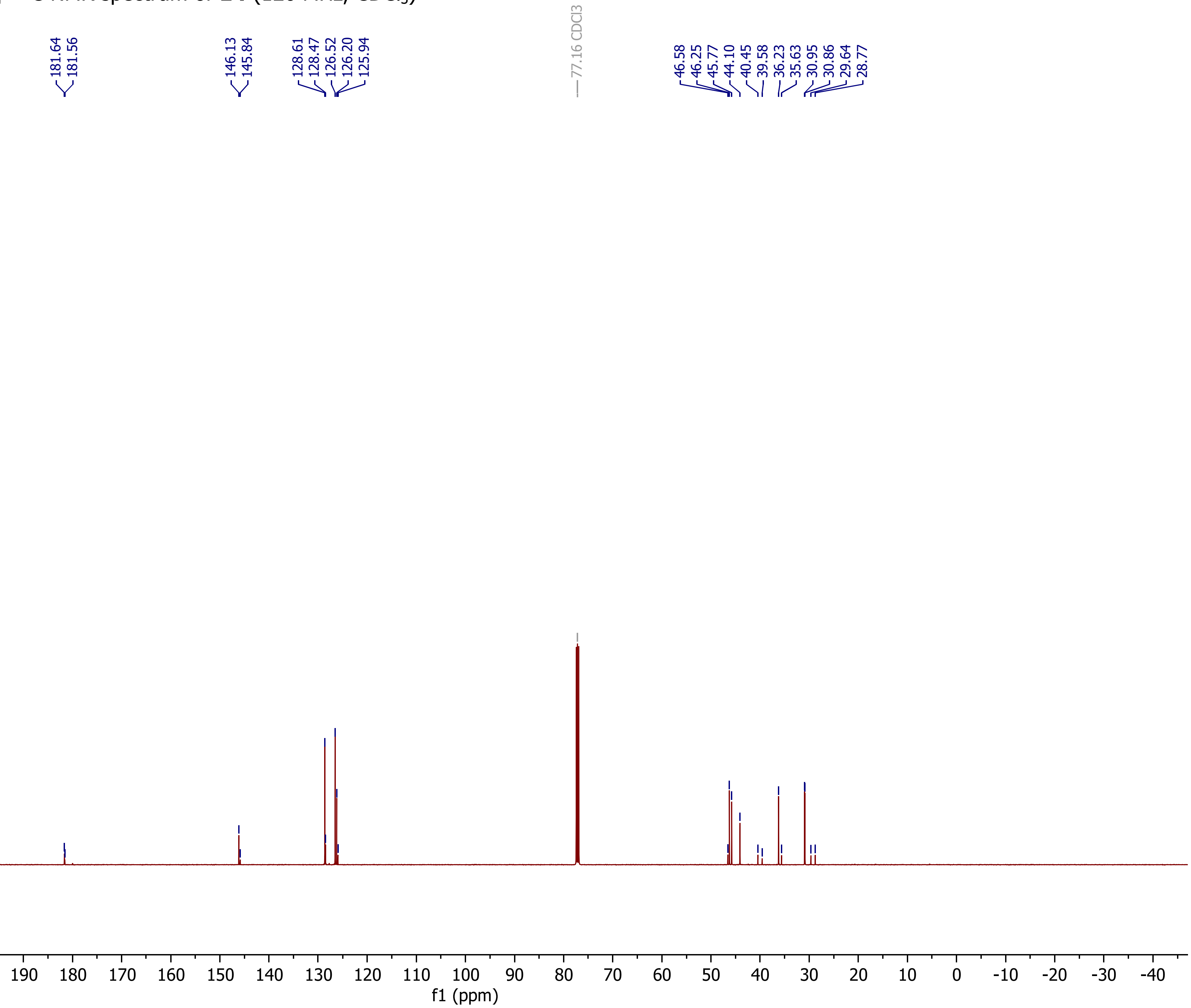

Supplementary Figure 83. ^{1}H NMR spectrum of **SI-39** (500 MHz, Acetone-d_6)

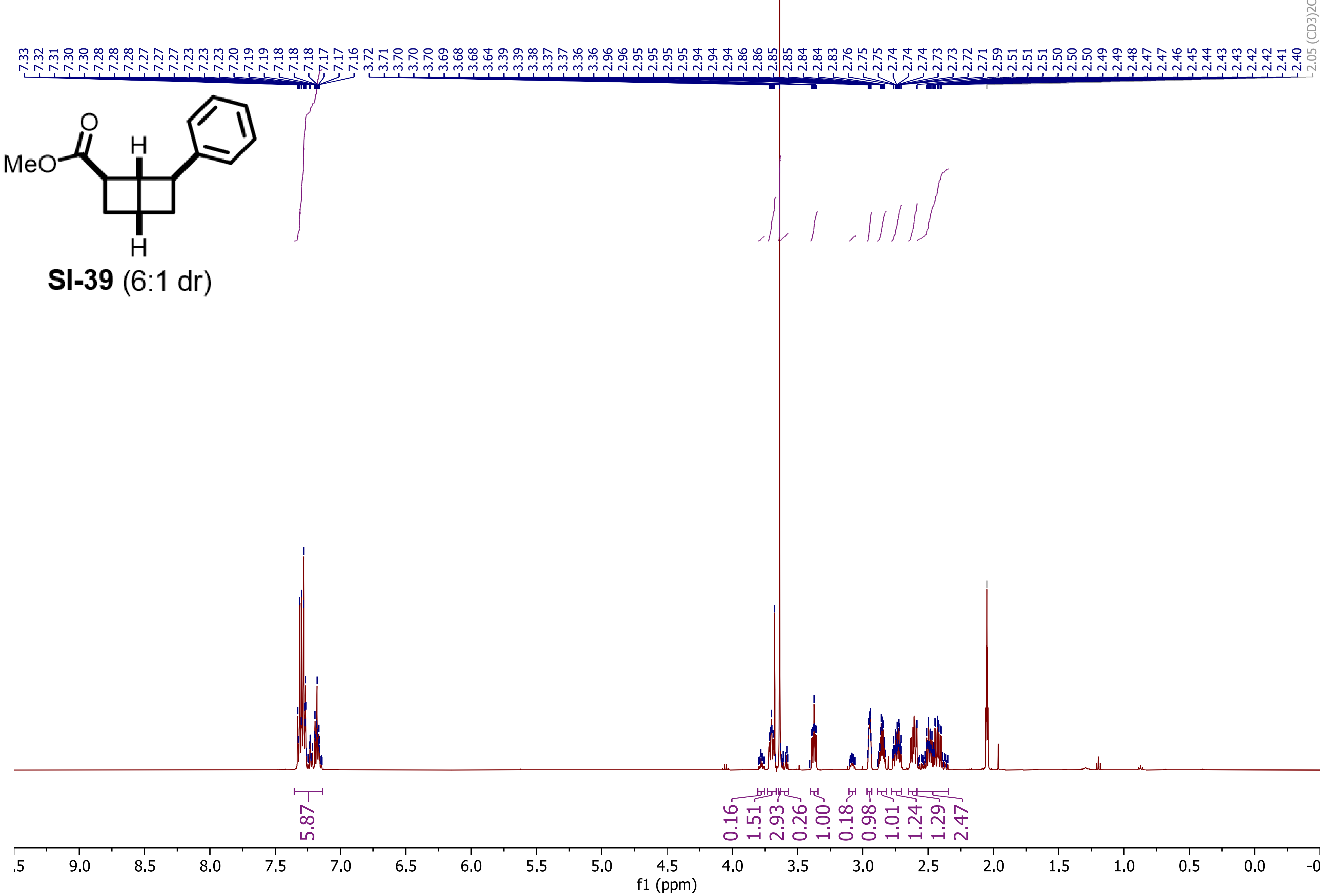

Supplementary Figure 84. ^{1}H NMR spectrum of **15** (500 MHz, $CDCl_3$)

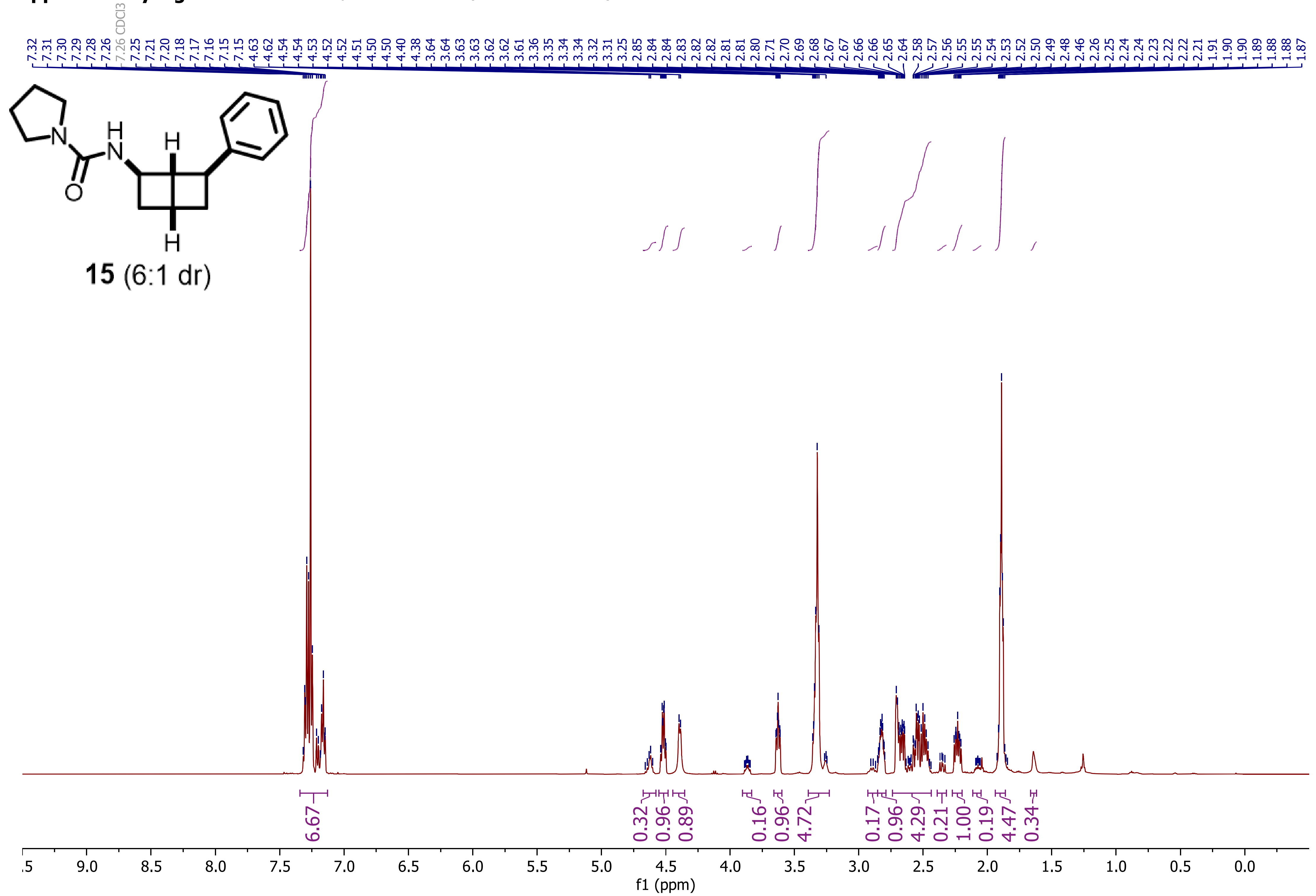

Supplementary Figure 85. ^{13}C NMR spectrum of **15** (126 MHz, $CDCl_3$)

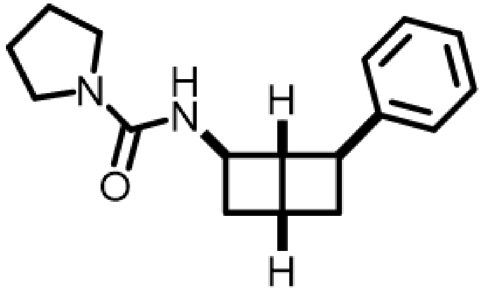

15 (6:1 dr)

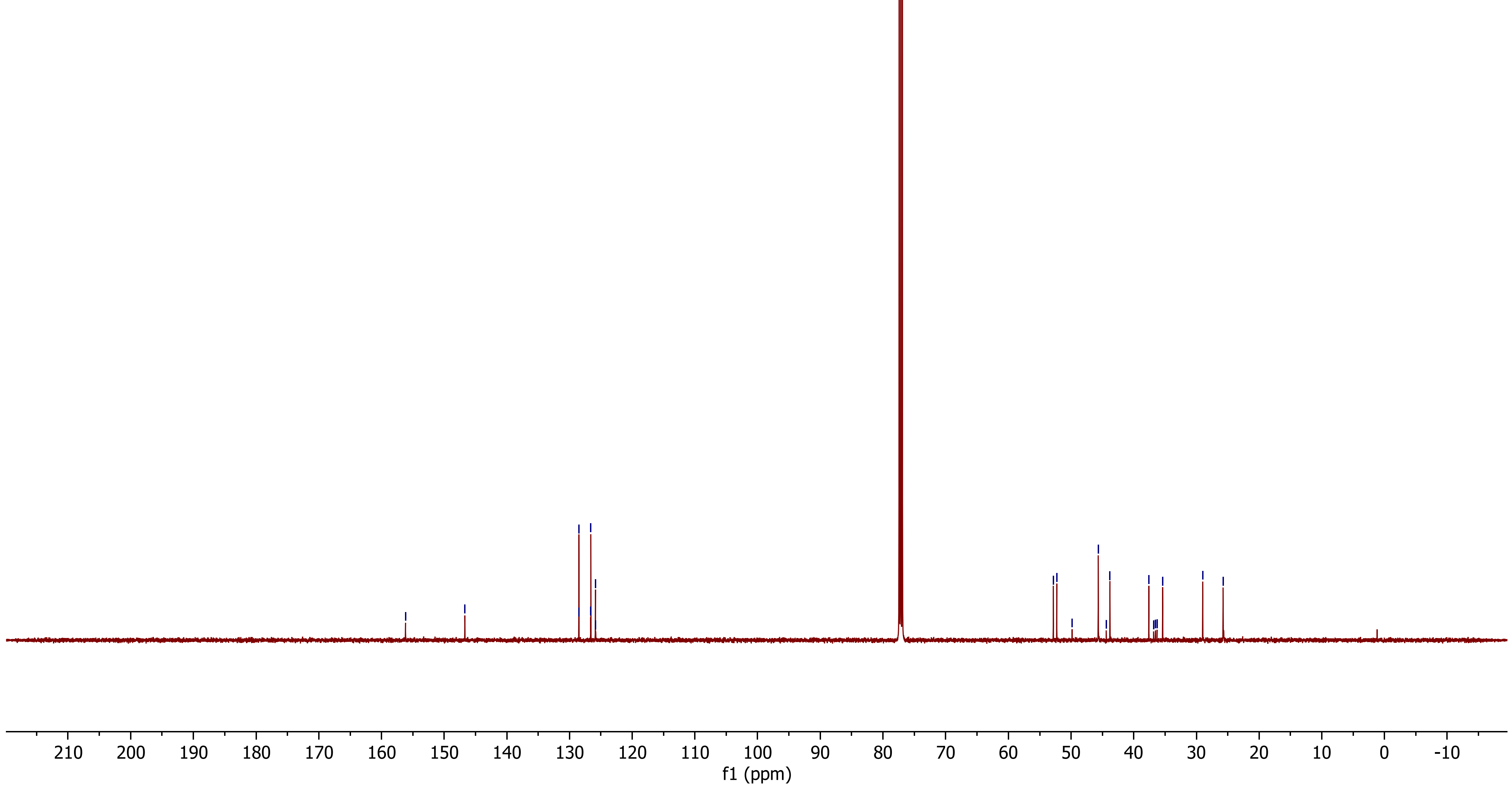

Supplementary Figure 86. ^{1}H NMR spectrum of **16** (500 MHz, $CDCl_3$)

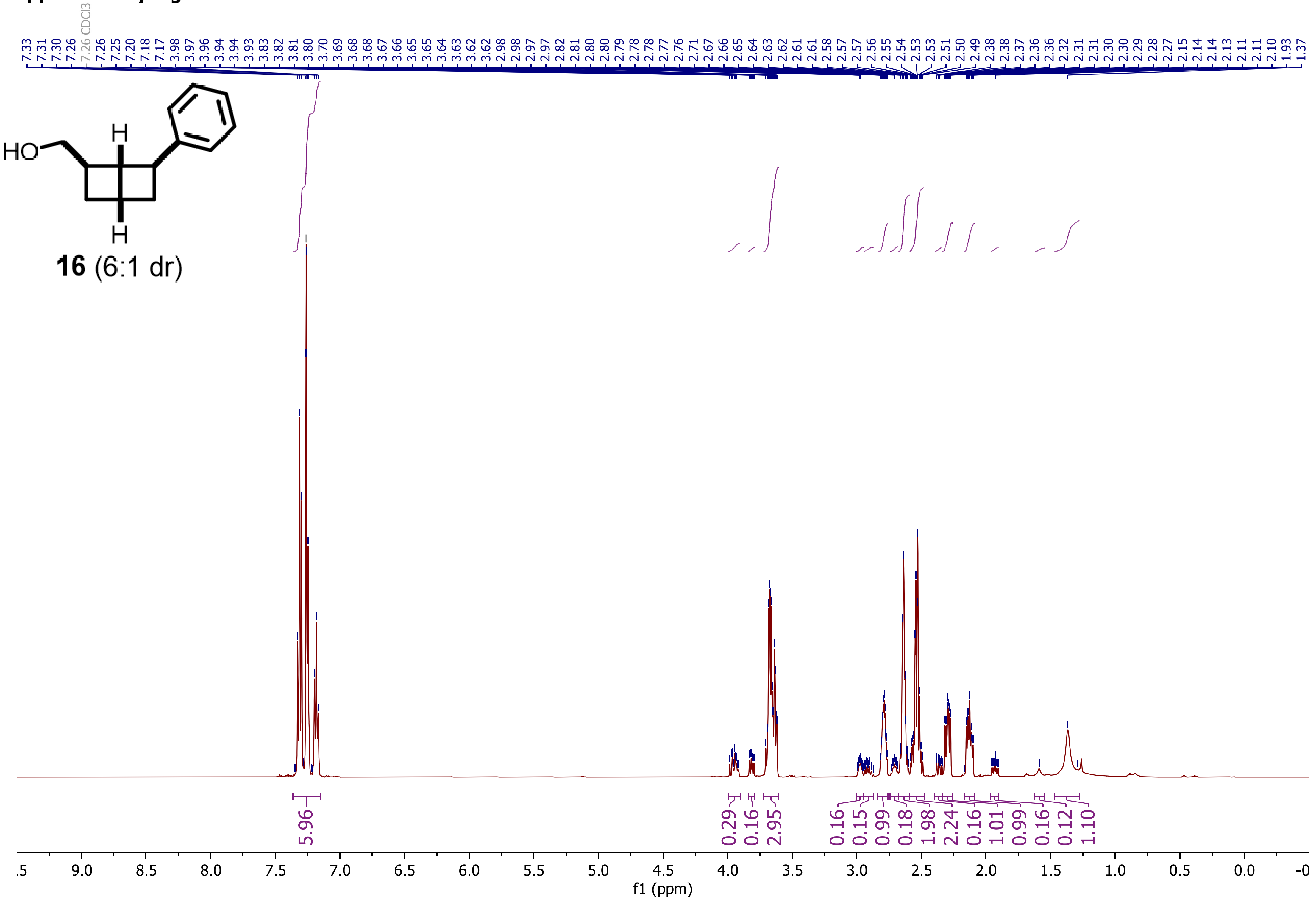

Supplementary Figure 87. ^{13}C NMR spectrum of **16** (126 MHz, $CDCl_3$)

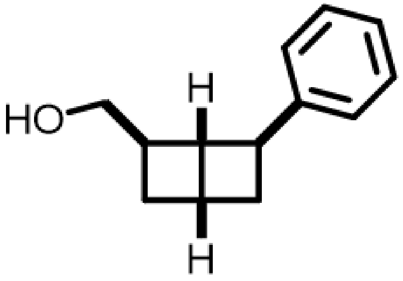

16 (6:1 dr)

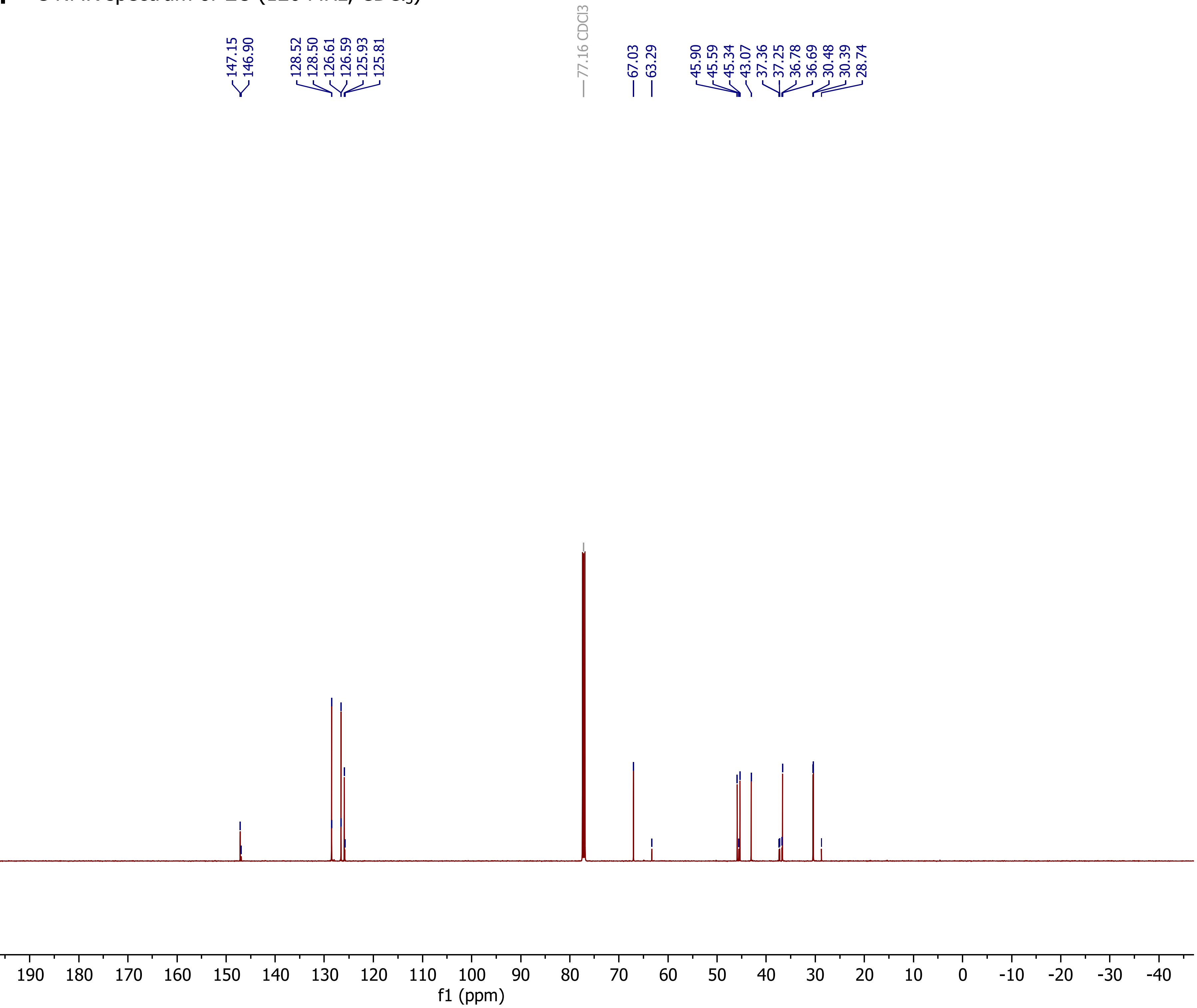

Supplementary Figure 88. ^{1}H NMR spectrum of **17** (500 MHz, $CDCl_3$)

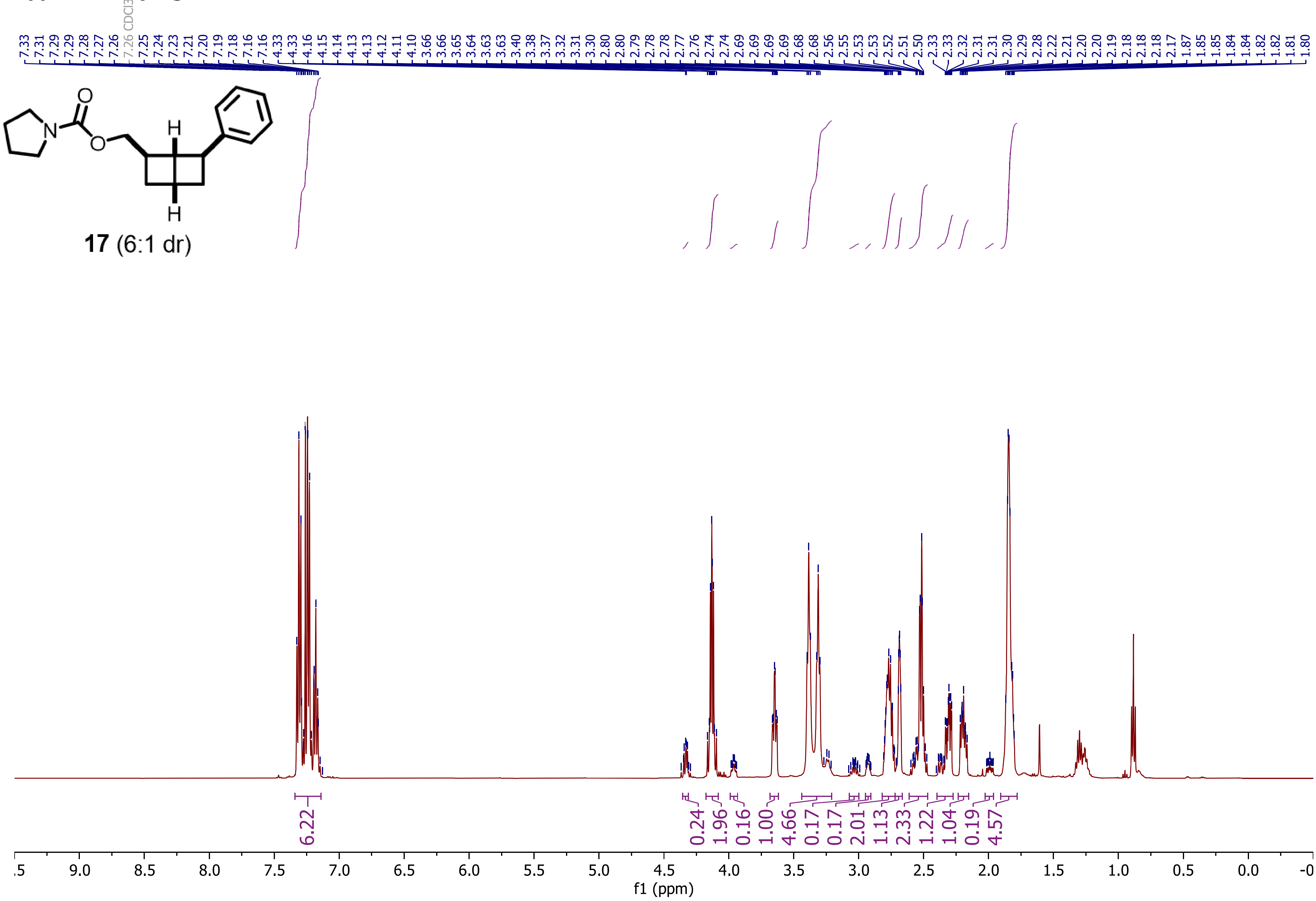

Supplementary Figure 89. ^{13}C NMR spectrum of **17** (126 MHz, $CDCl_3$)

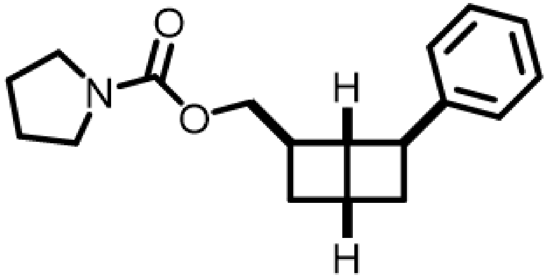

17 (6:1 dr)

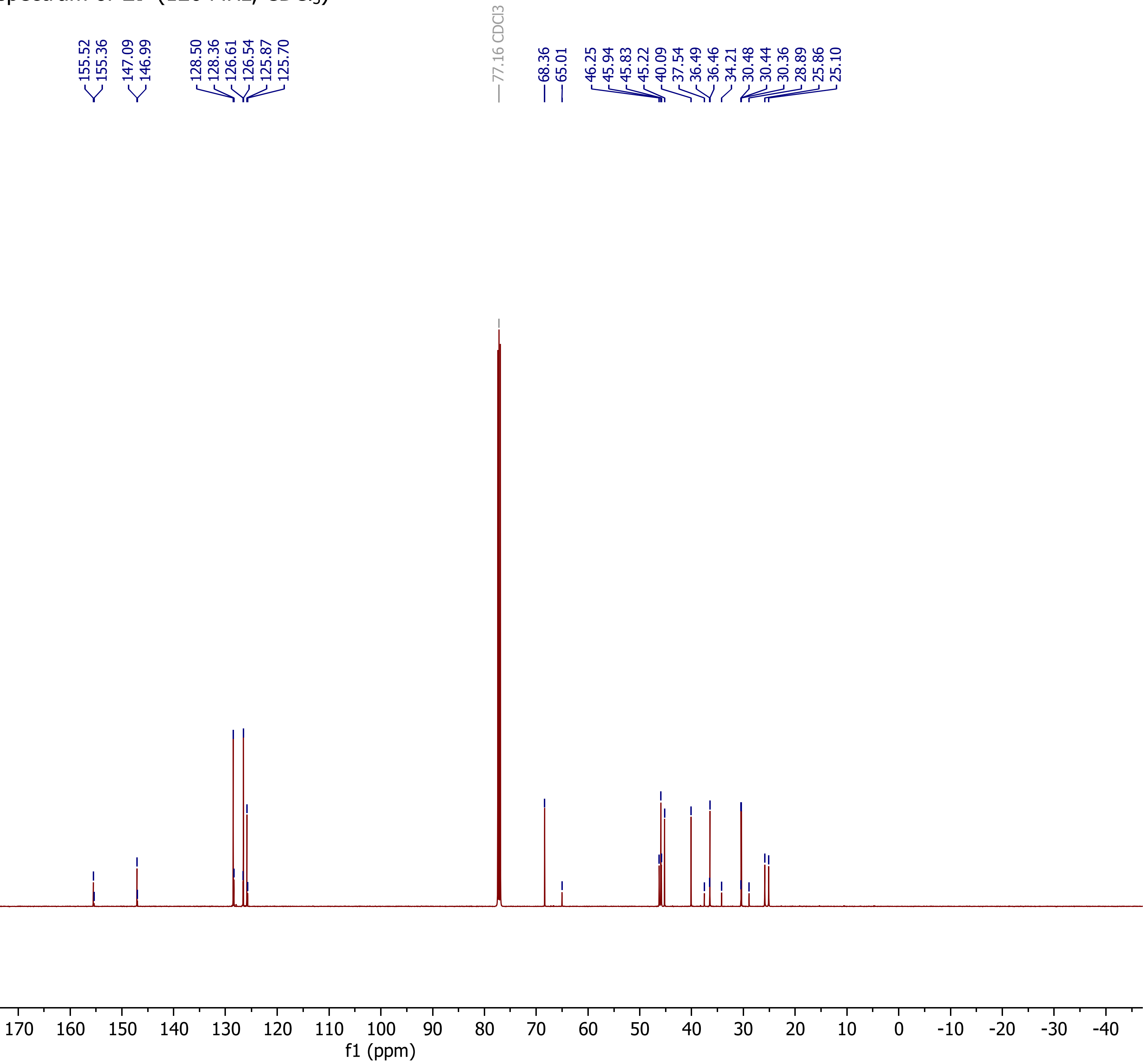

Supplementary Figure 90. ^{1}H NMR spectrum of **6** (500 MHz, Acetone-d_6)

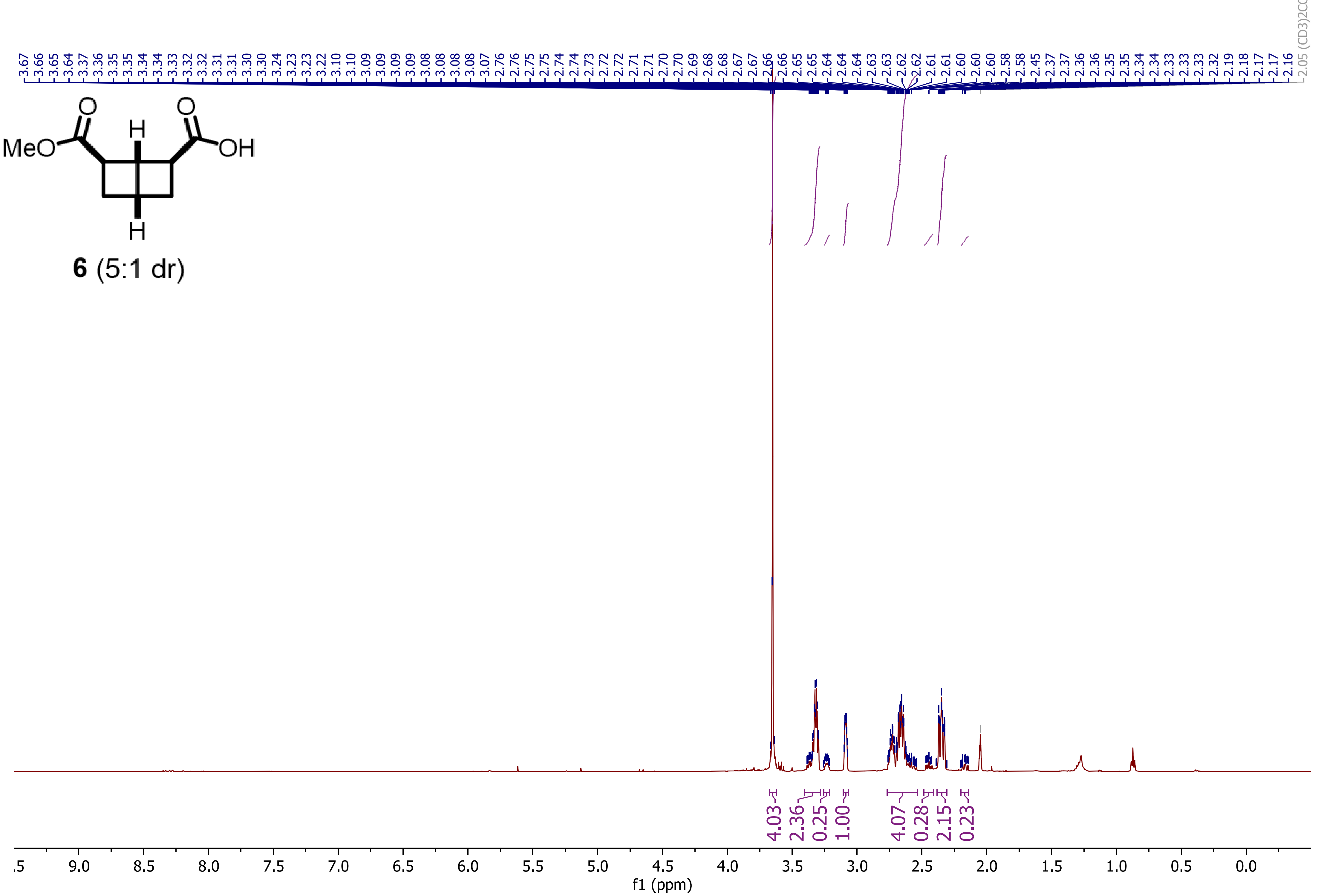

Supplementary Figure 91. ^{13}C NMR spectrum of **6** (126 MHz, Acetone-d_6)

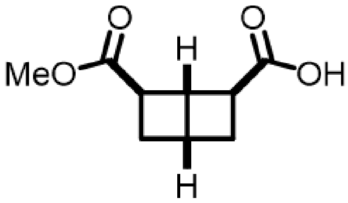

6 (5:1 dr)

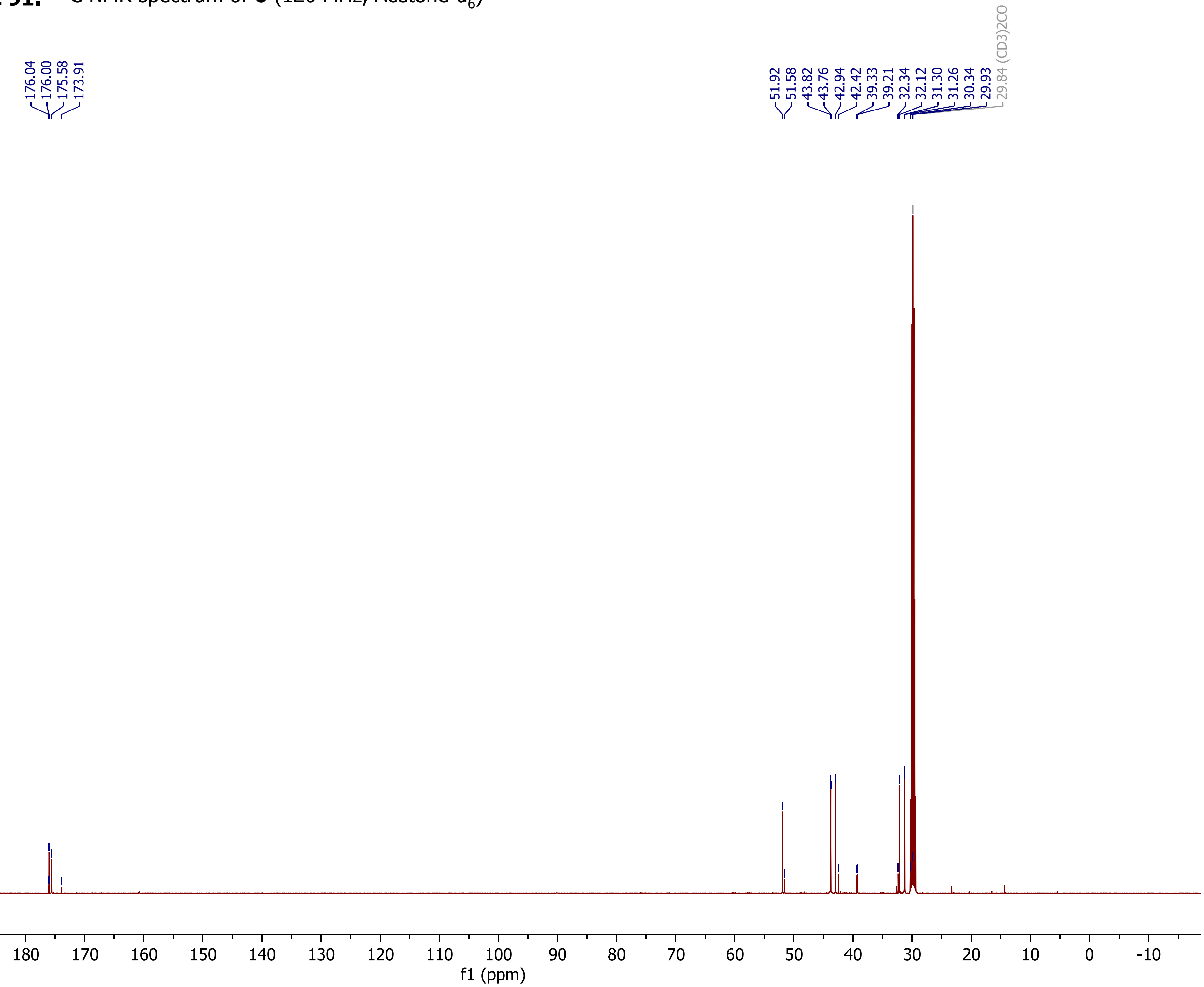

Supplementary Figure 92. ^{1}H NMR spectrum of **SI-40** (500 MHz, $CDCl_3$)

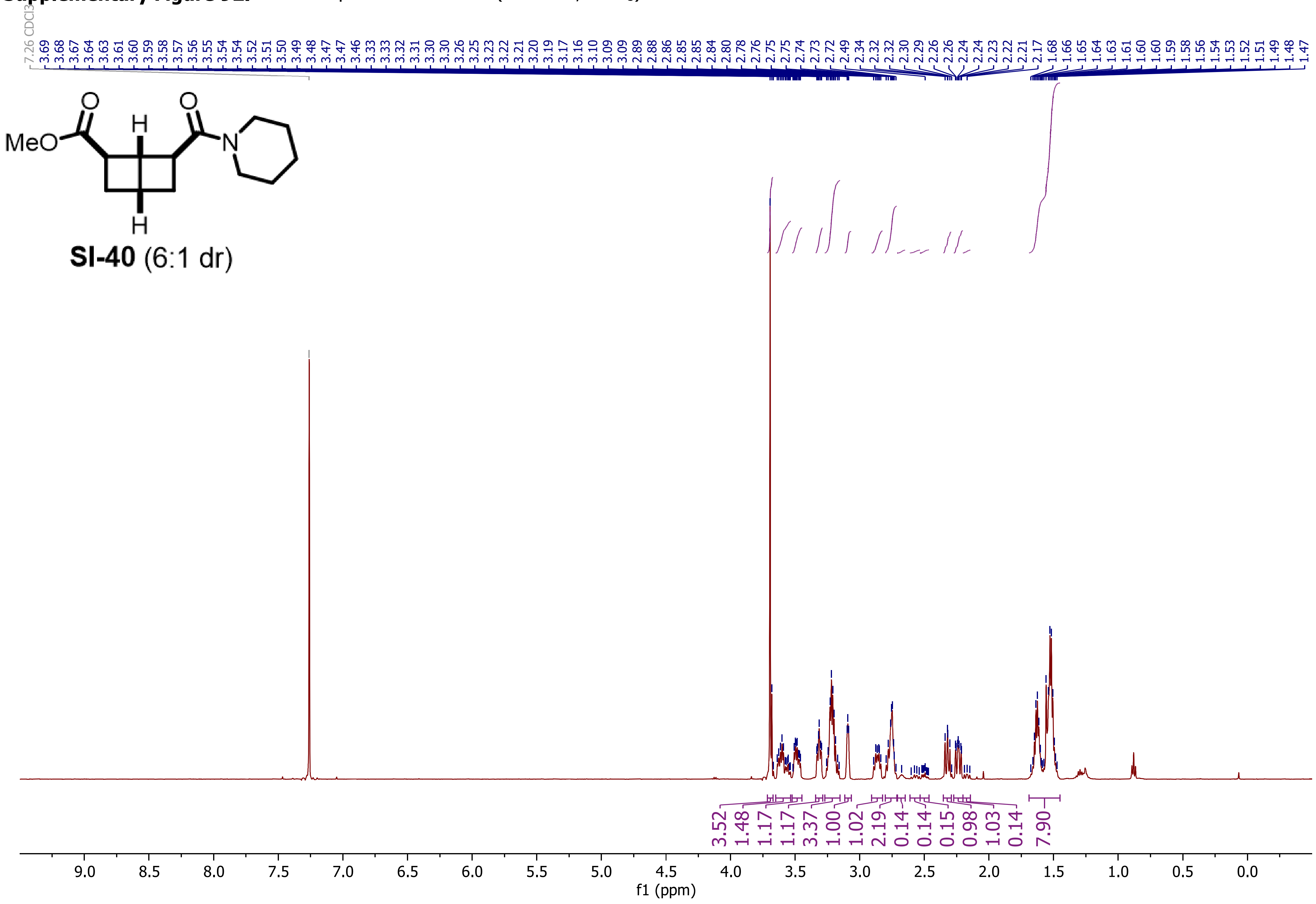

Supplementary Figure 93. ^{13}C NMR spectrum of **SI-40** (126 MHz, $CDCl_3$)

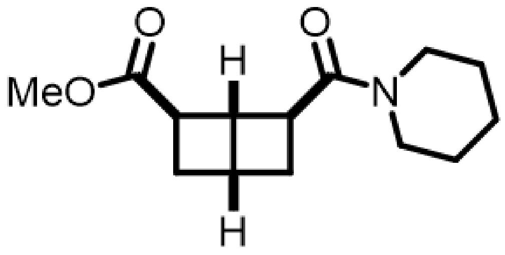

SI-40 (6:1 dr)

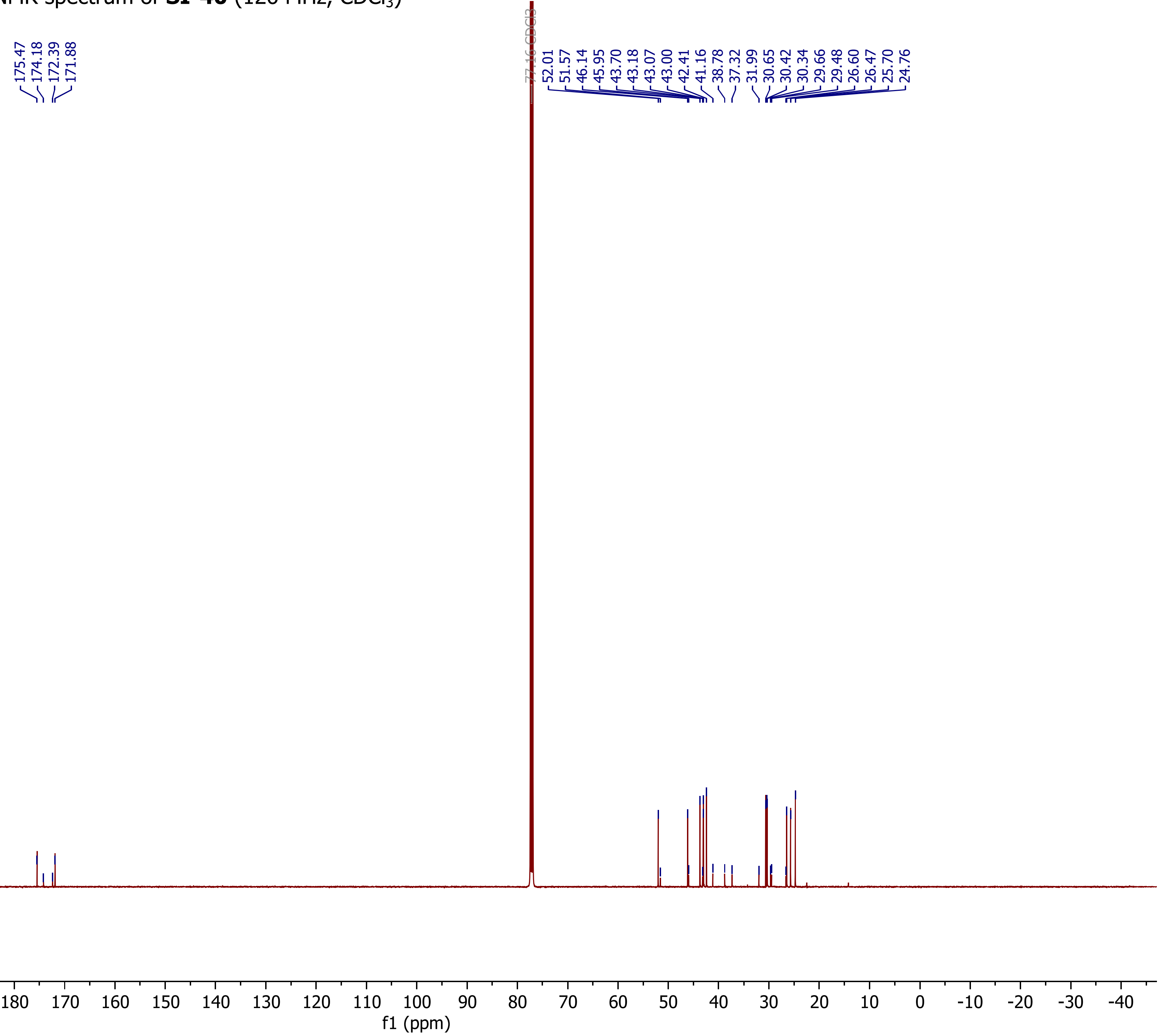

Supplementary Figure 94. ^{1}H NMR spectrum of **SI-41** (500 MHz, $CDCl_3$)

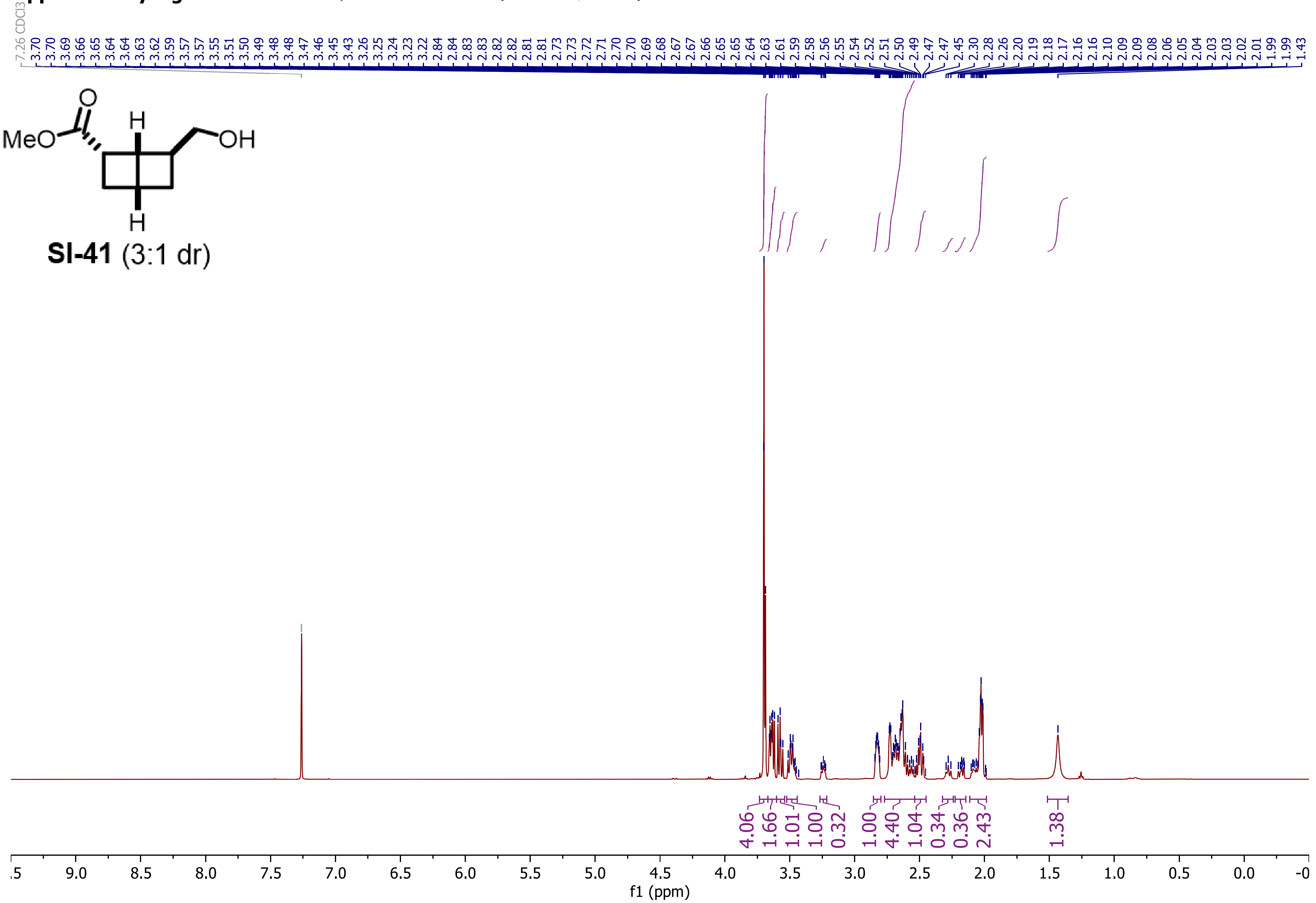

Supplementary Figure 95. ^{13}C NMR spectrum of **SI-41** (126 MHz, $CDCl_3$)

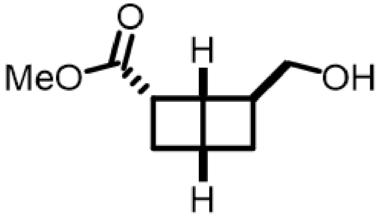

SI-41 (3:1 dr)

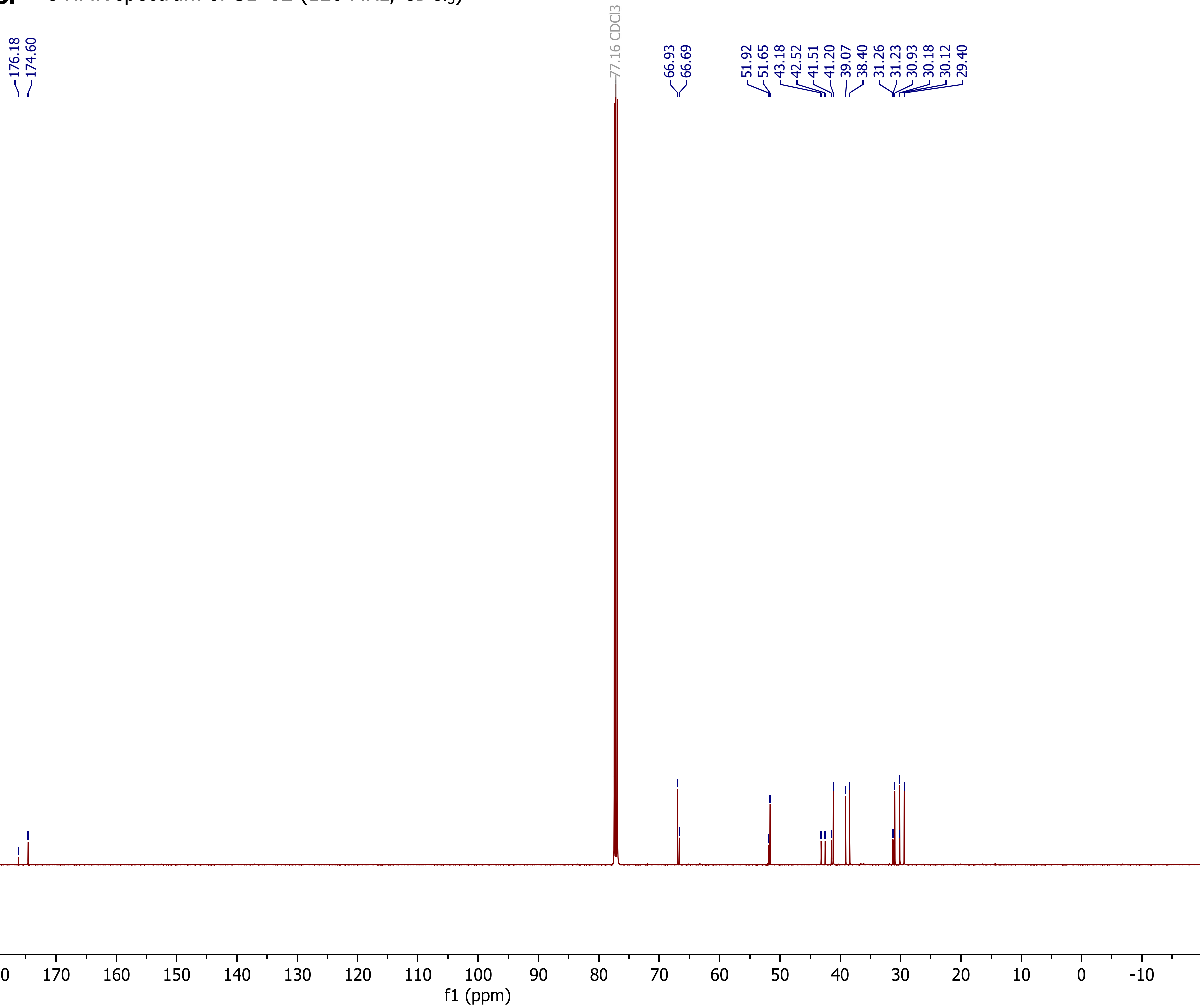

Supplementary Figure 96. ^{1}H NMR spectrum of **SI-42** (500 MHz, $CDCl_3$)

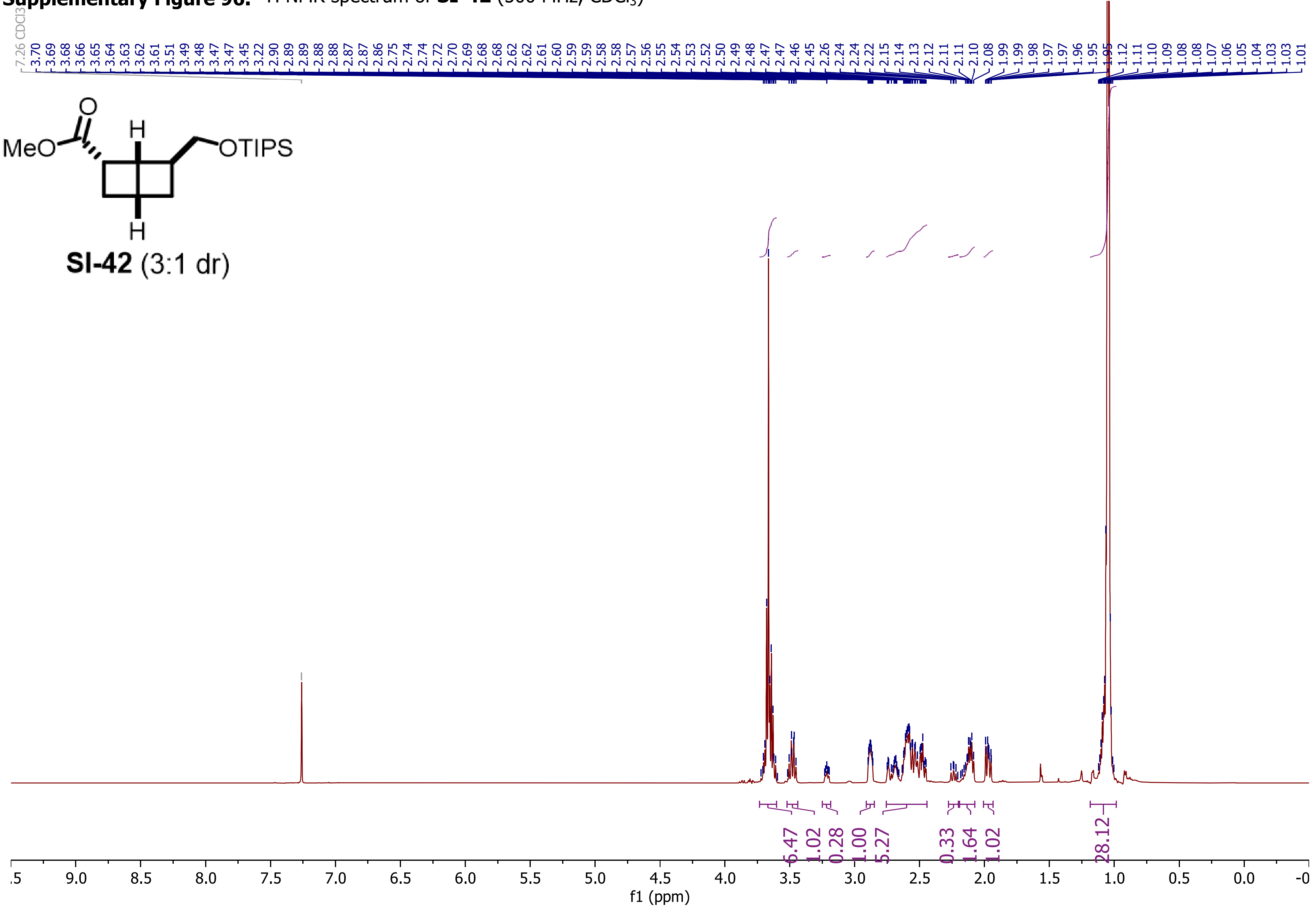

Supplementary Figure 97. ^{13}C NMR spectrum of **SI-42** (126 MHz, $CDCl_3$)

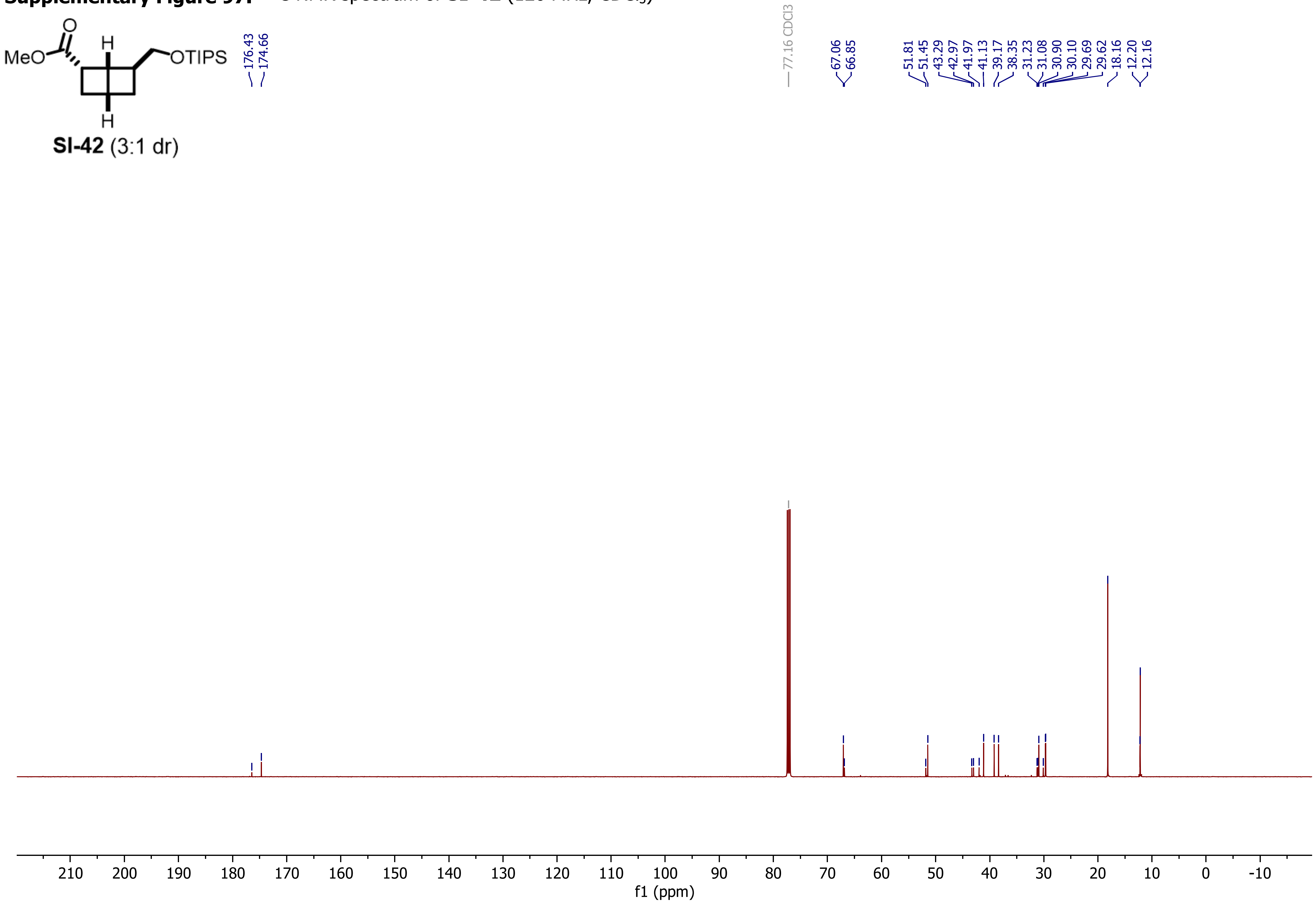

Supplementary Figure 98. ^{1}H NMR spectrum of **7** (500 MHz, $CDCl_3$)

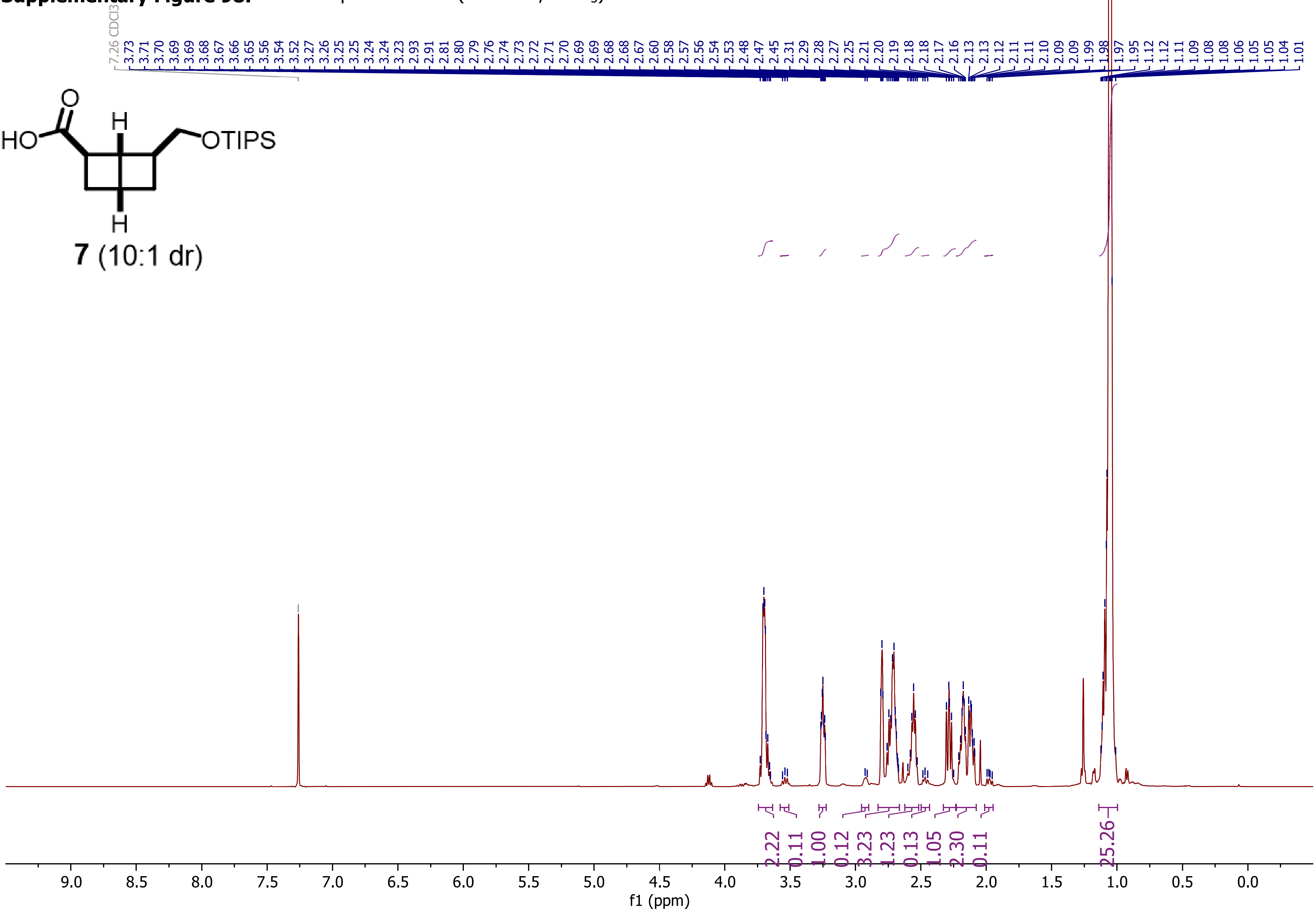

Supplementary Figure 99. ^{13}C NMR spectrum of **7** (126 MHz, $CDCl_3$)

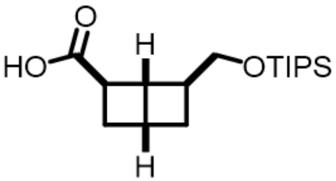

7 (10:1 dr)

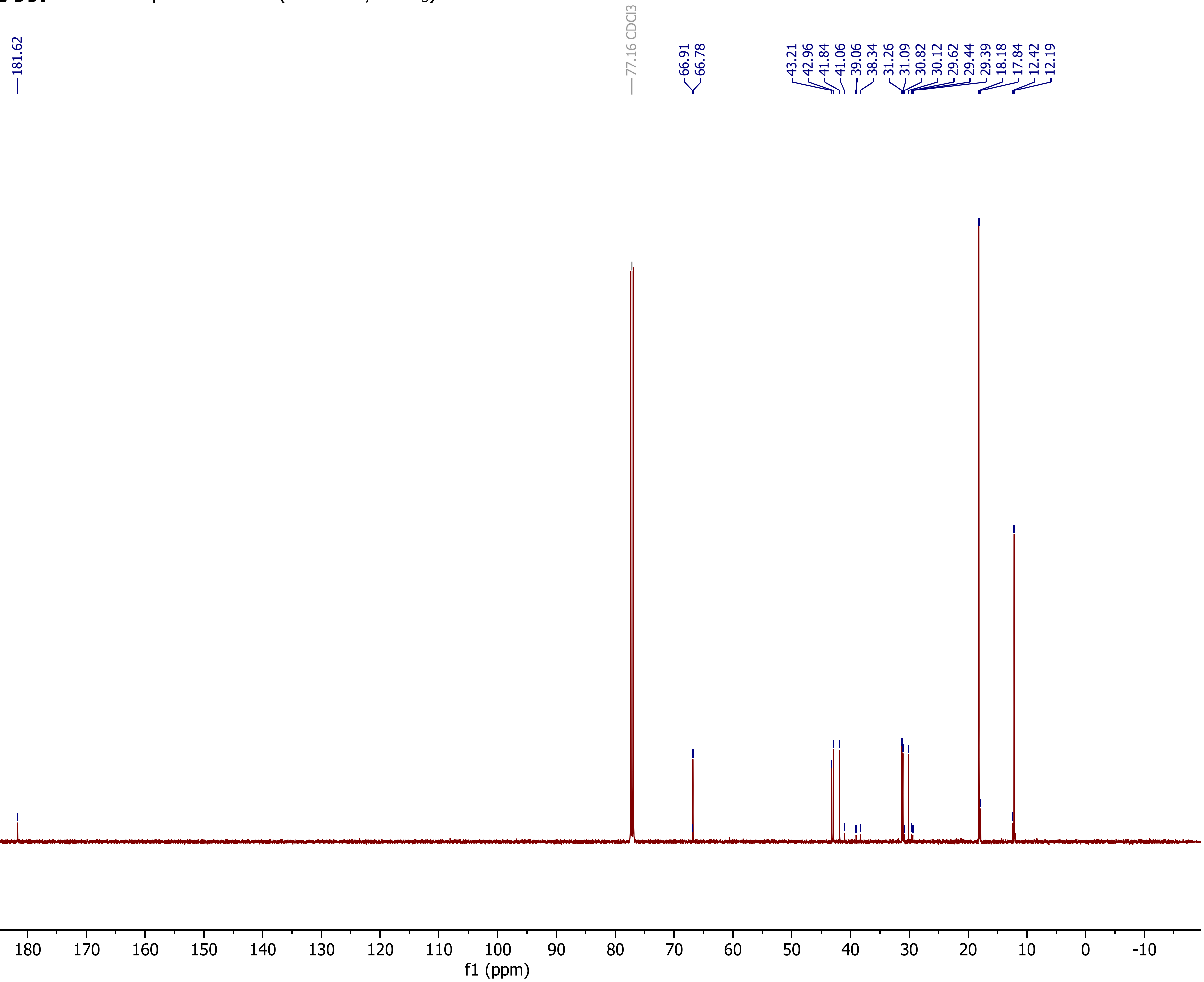

Supplementary Figure 100. COSY NMR spectrum of **7** (500 MHz, $CDCl_3$)

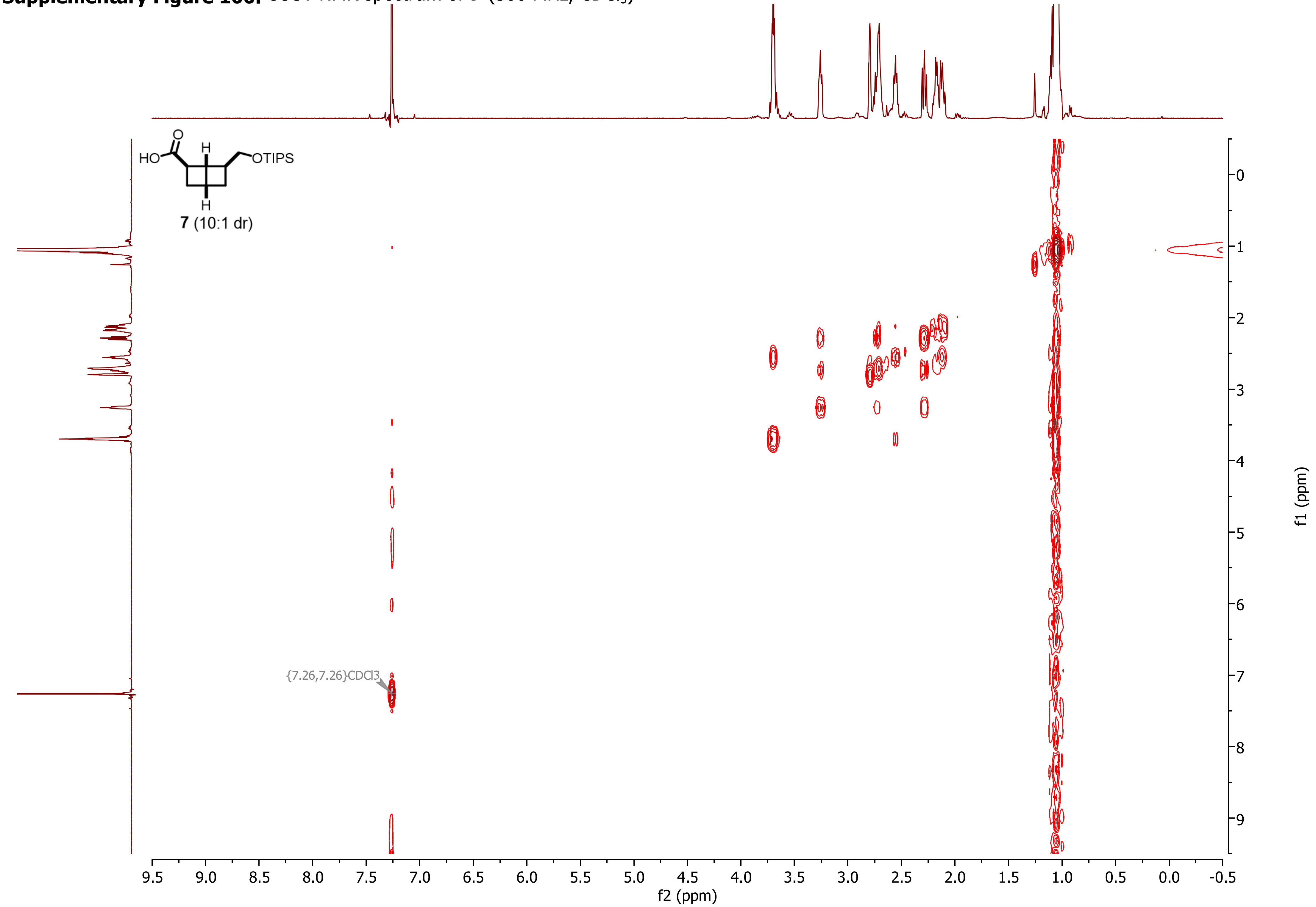

Supplementary Figure 101. NOESY NMR spectrum of **7** (500 MHz, $CDCl_3$)

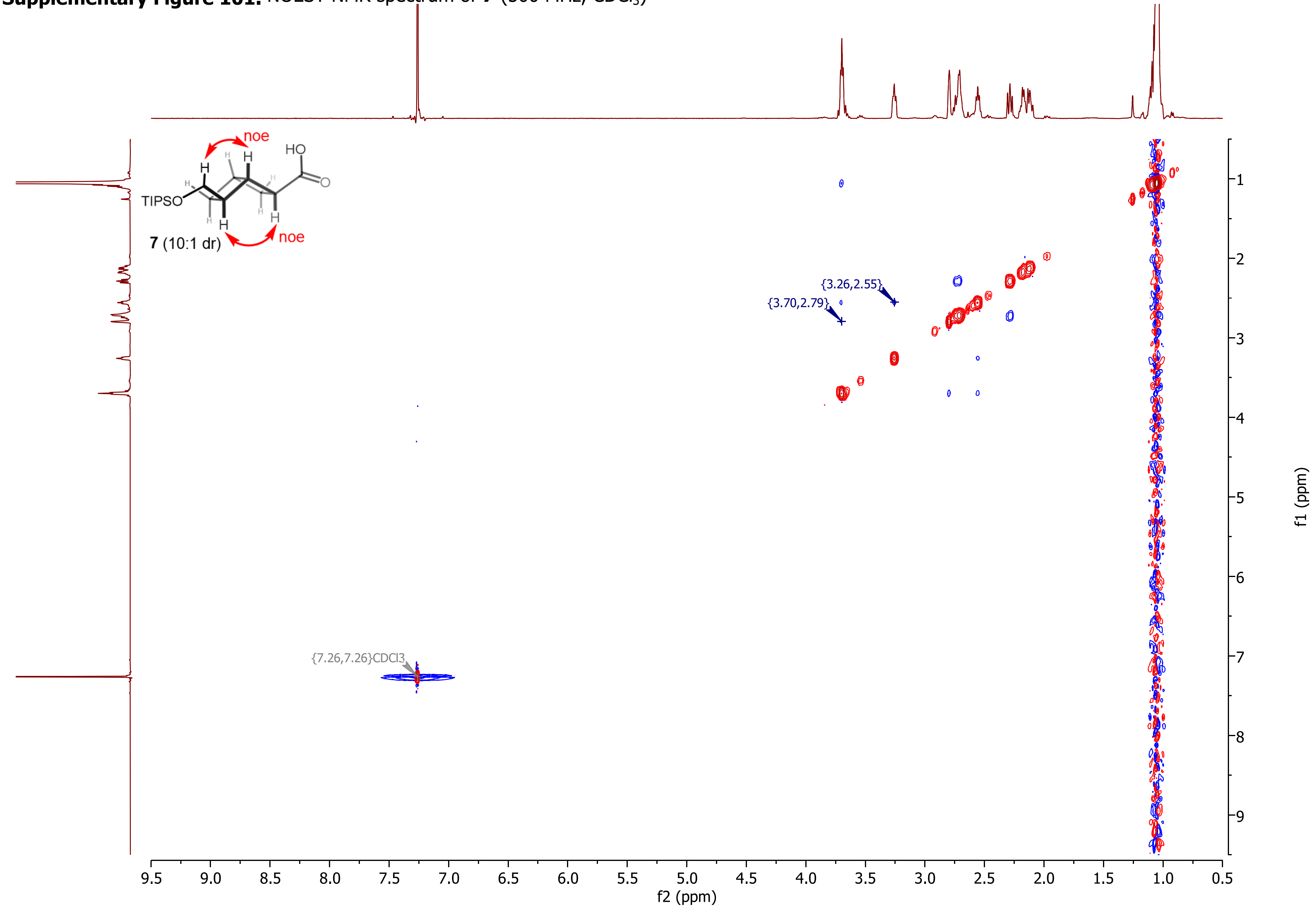

Supplementary Figure 102. ^{1}H NMR spectrum of **18** (500 MHz, $CDCl_3$)

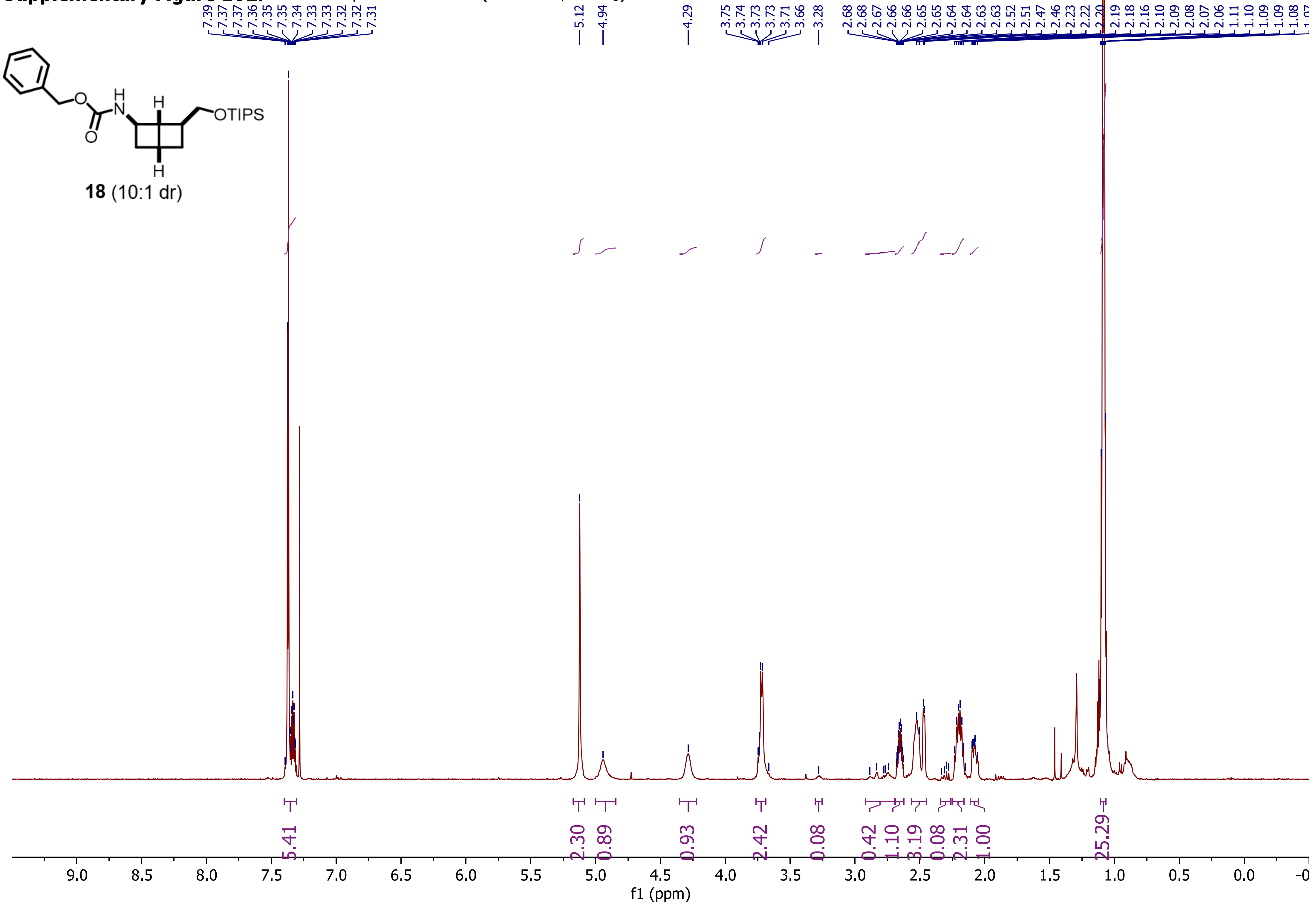

Supplementary Figure 103. ^{13}C NMR spectrum of **18** (126 MHz, $CDCl_3$)

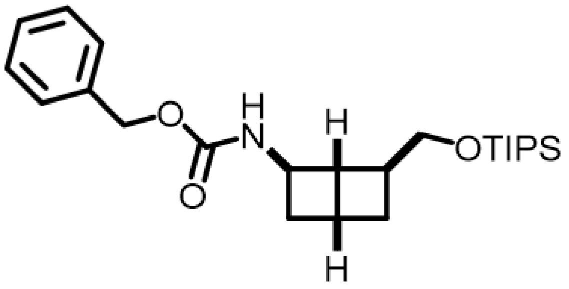

18 (10:1 dr)

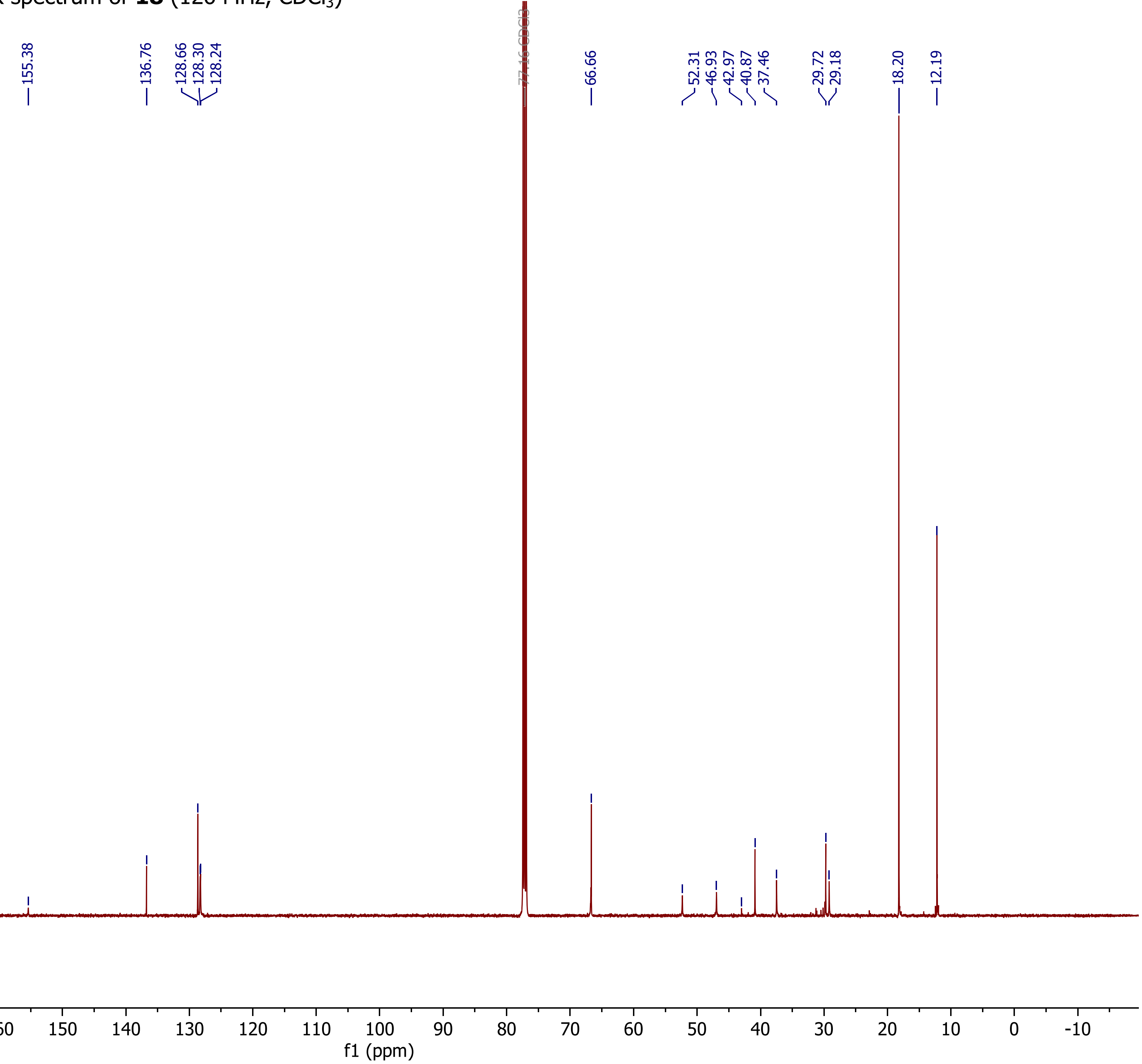

Supplementary Figure 104. ^{1}H NMR spectrum of **SI-43** (500 MHz, $CDCl_3$)

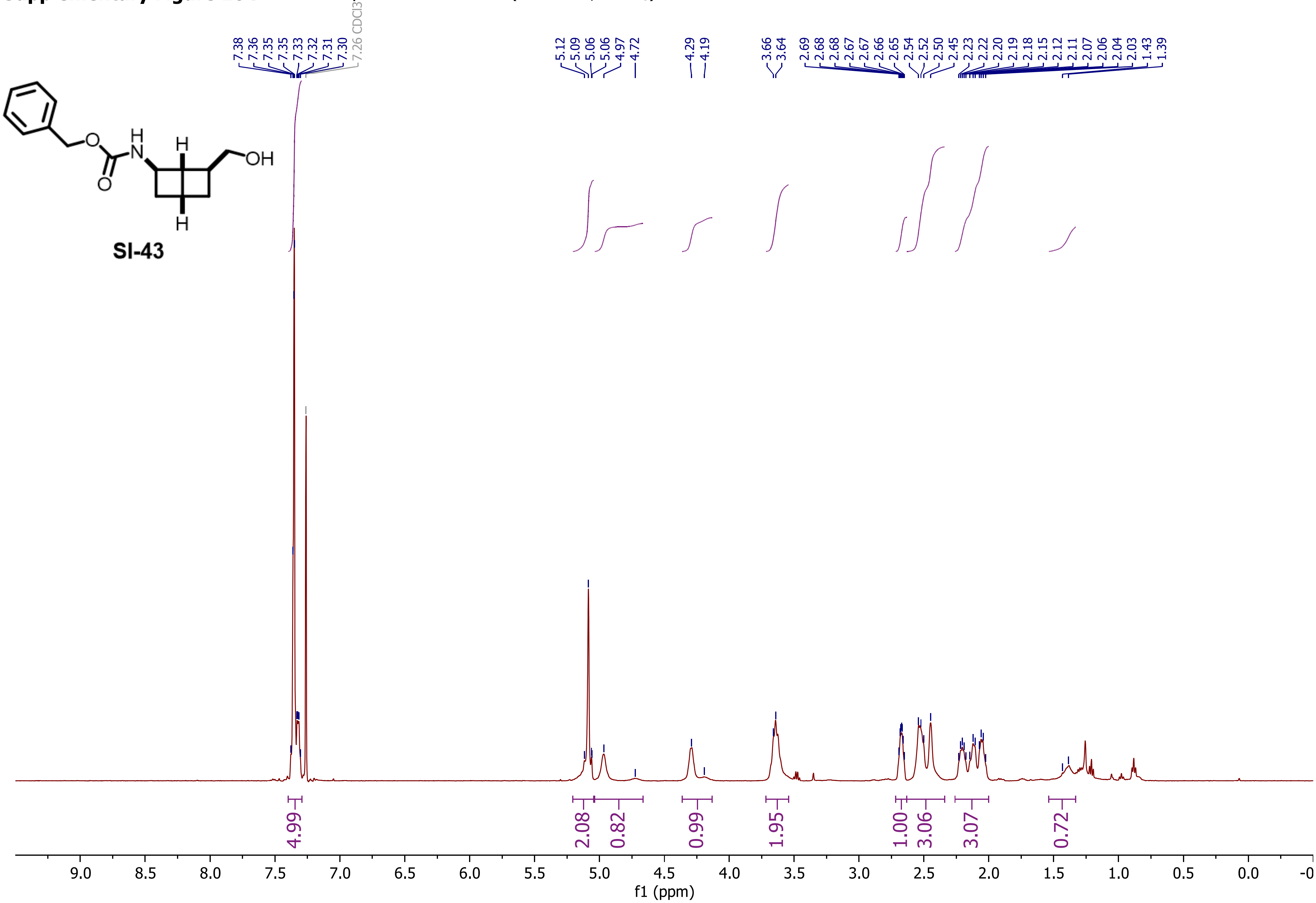

Supplementary Figure 105. ^{13}C NMR spectrum of **SI-43** (126 MHz, $CDCl_3$)

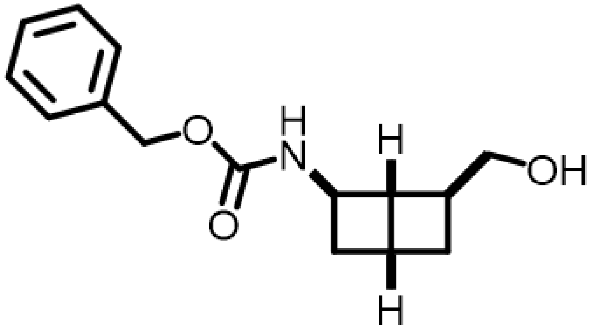

SI-43

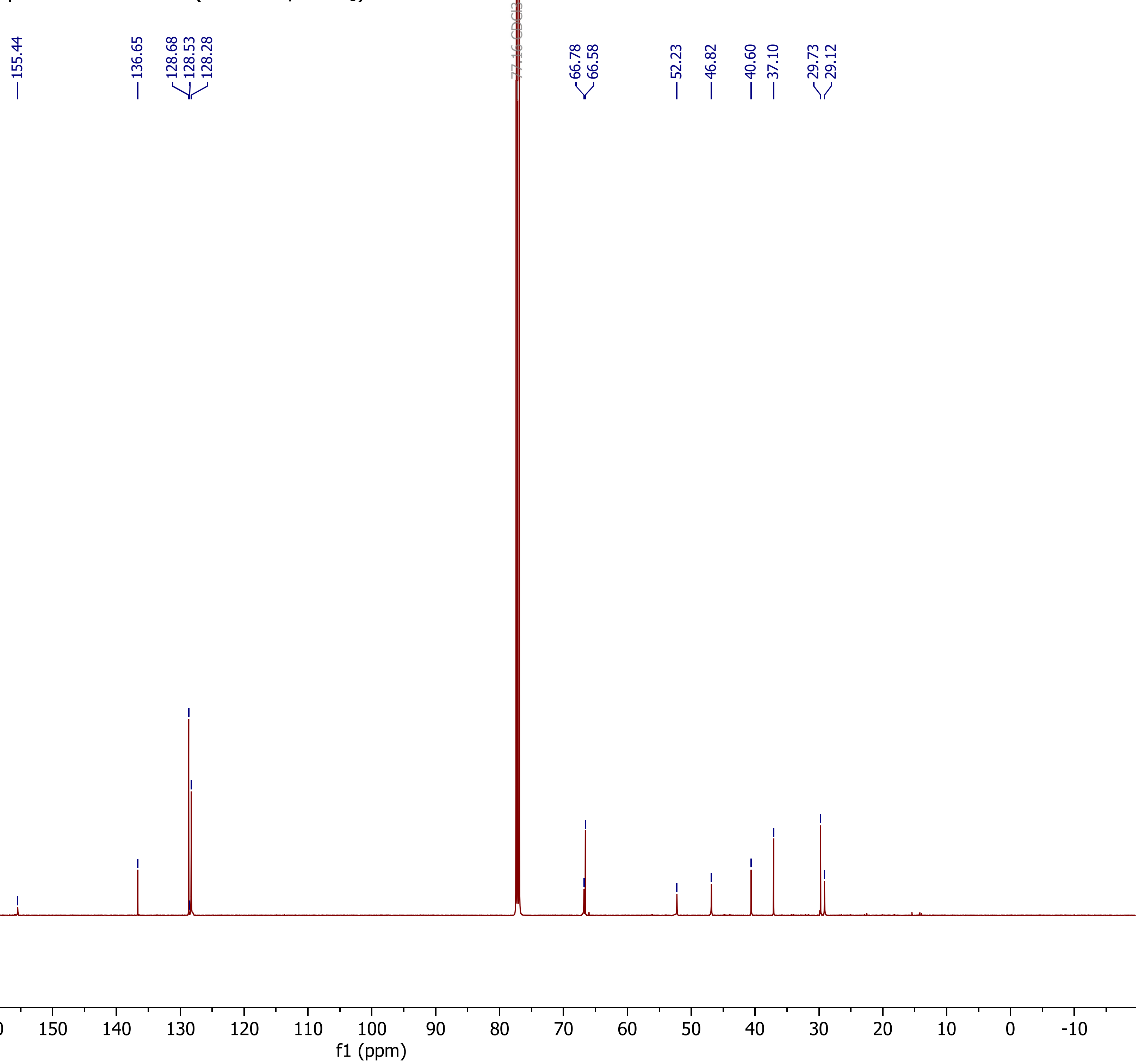

Supplementary Figure 106. ^{1}H NMR spectrum of **19** (500 MHz, $CDCl_3$, 40 °C)

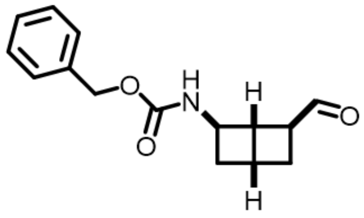

19

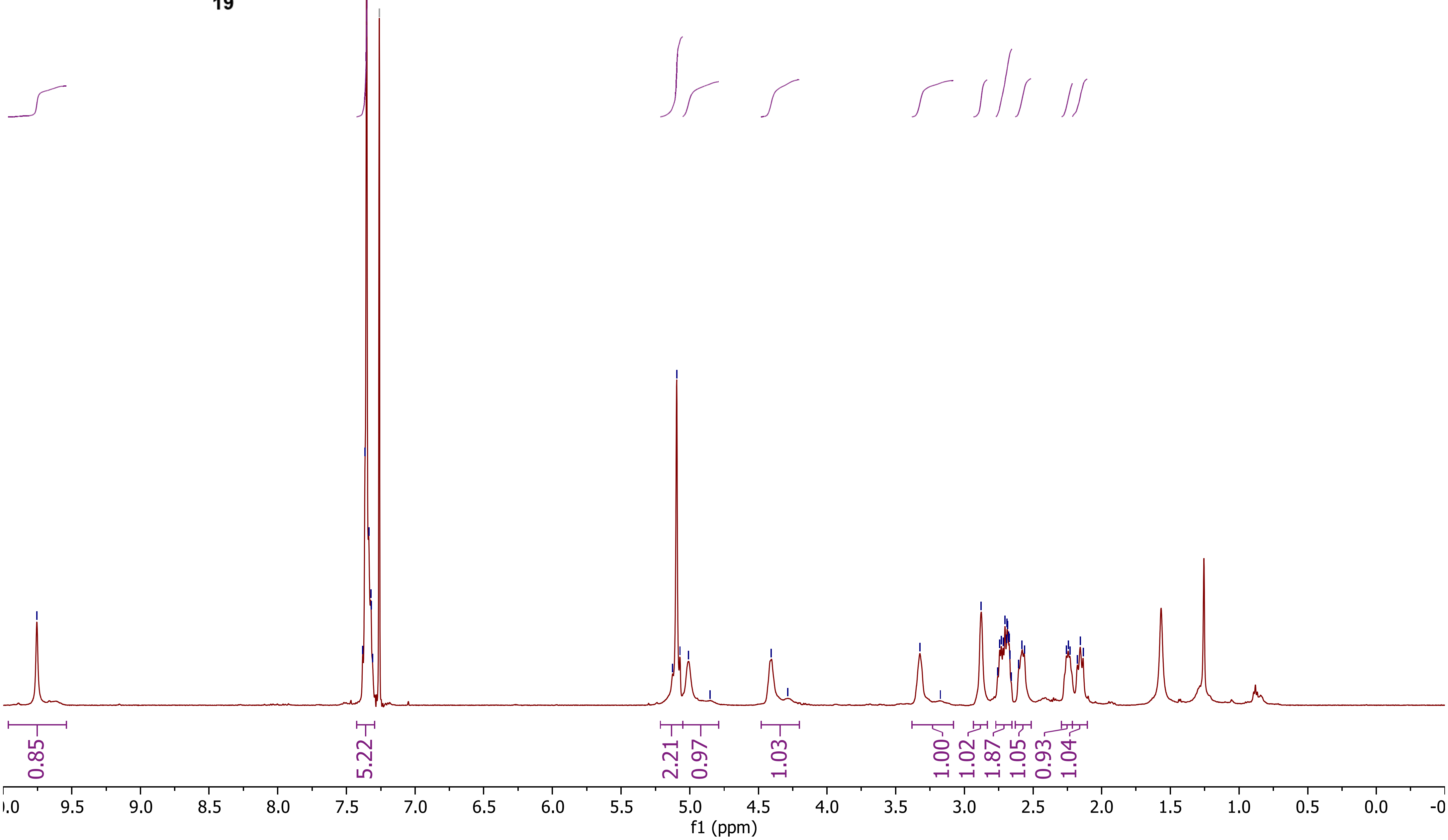

Supplementary Figure 107. ^{13}C NMR spectrum of **19** (126 MHz, $CDCl_3$)

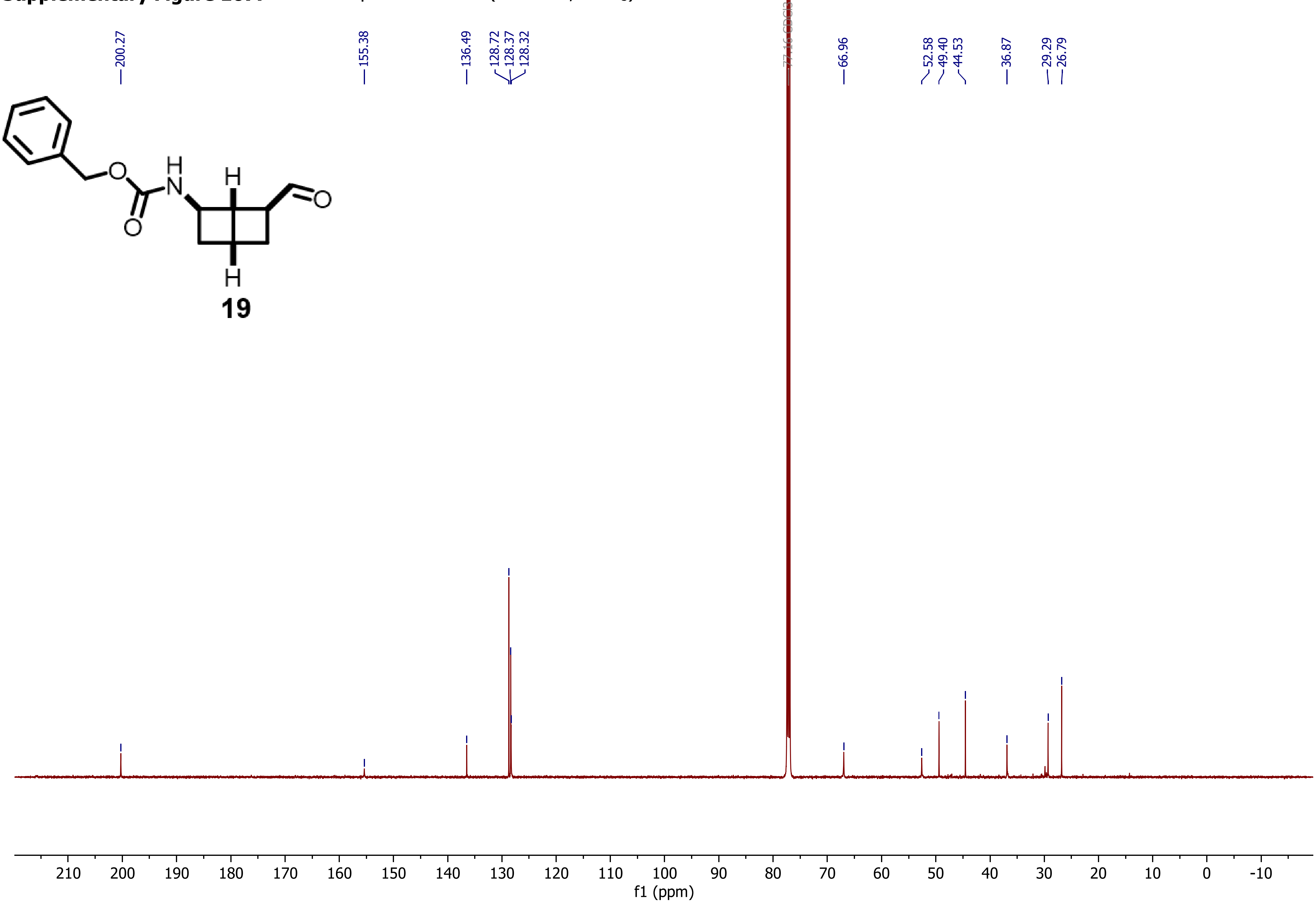

Supplementary Figure 108. ^{1}H NMR spectrum of **20** (500 MHz, $CDCl_3$)

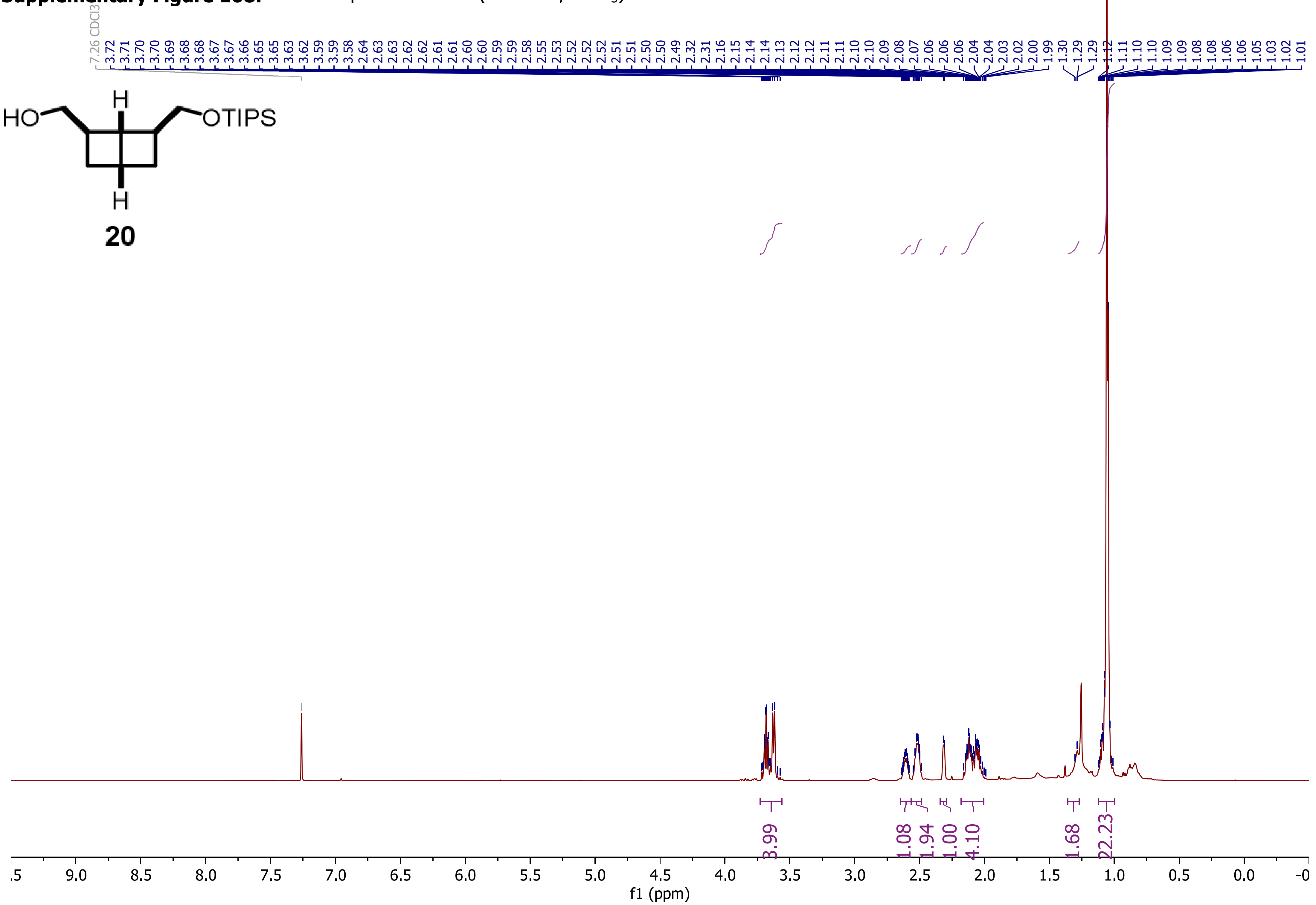

Supplementary Figure 109. ^{13}C NMR spectrum of **20** (126 MHz, $CDCl_3$)

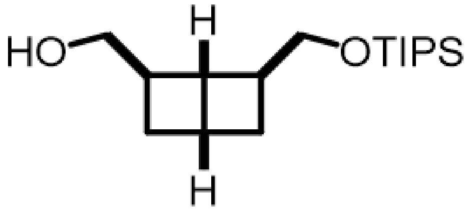

20

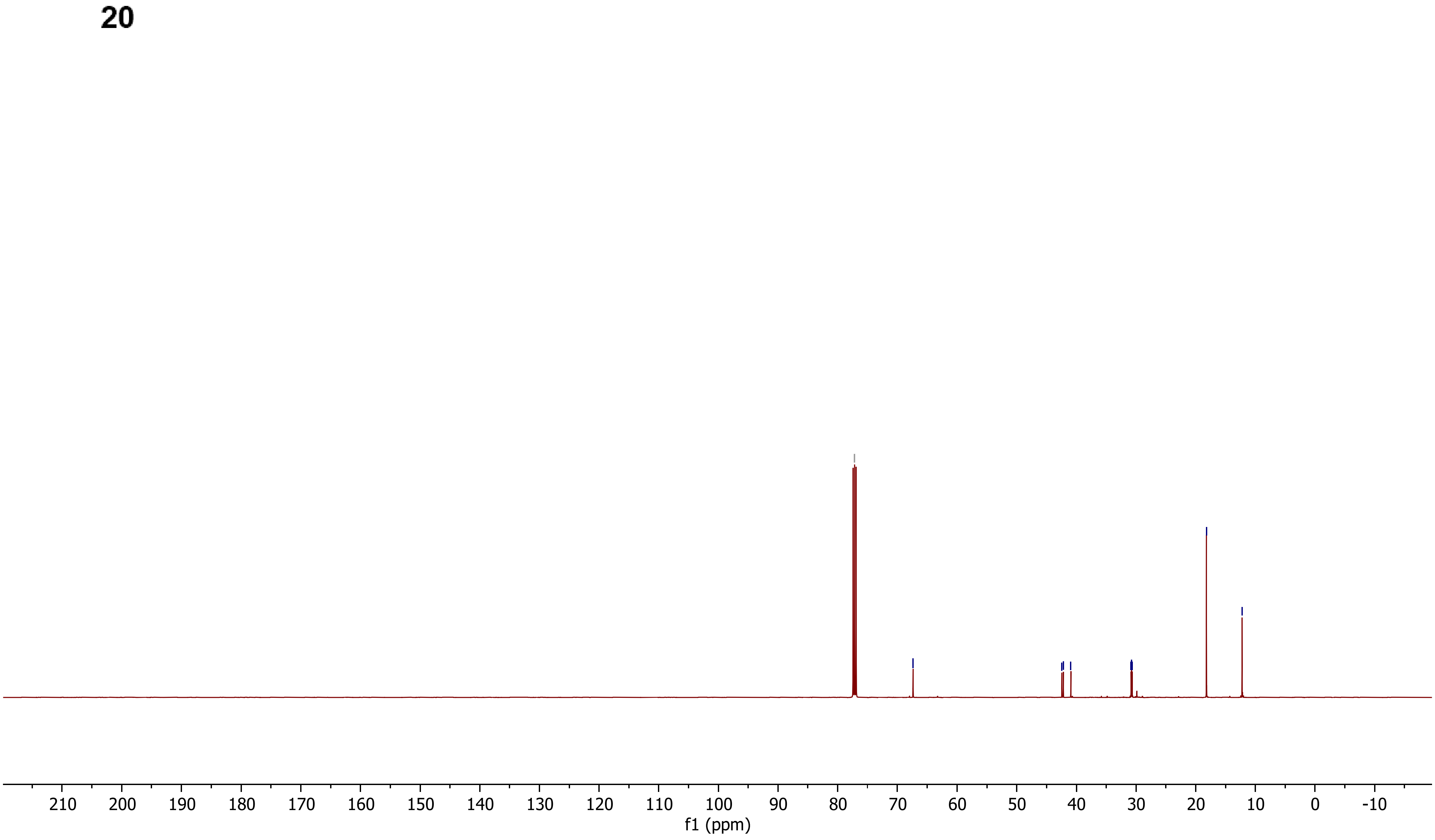

Supplementary Figure 110. ^{1}H NMR spectrum of **21** (500 MHz, $CDCl_3$)

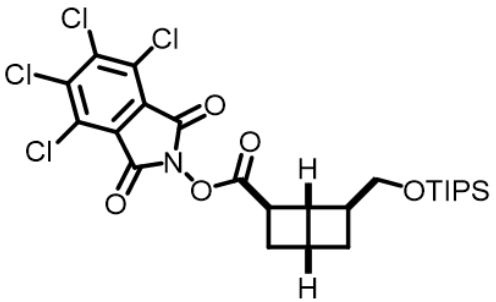

21 (10:1 dr)

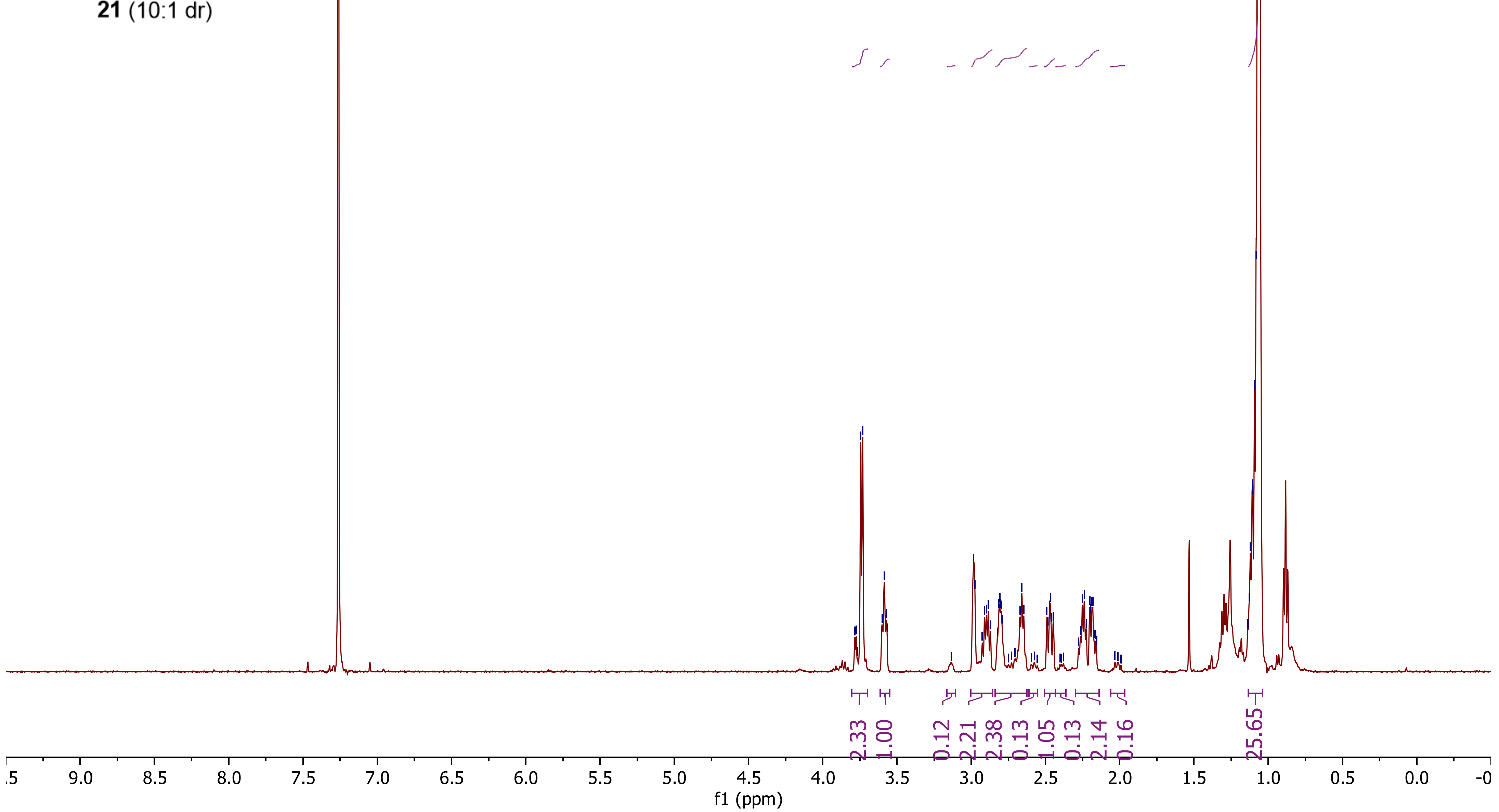

Supplementary Figure 111. ^{13}C NMR spectrum of **21** (126 MHz, $CDCl_3$)

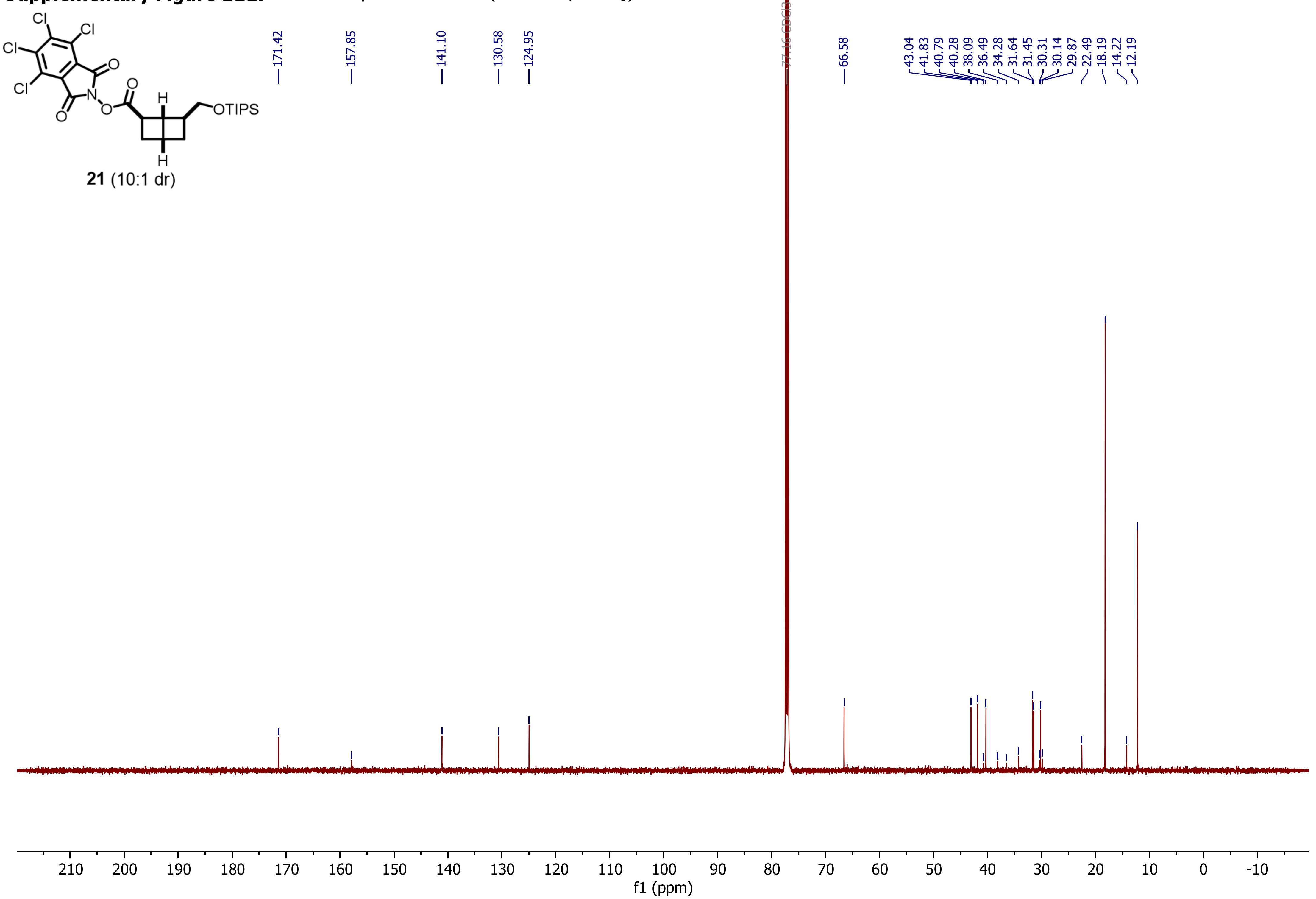

Supplementary Figure 112. ^1H NMR spectrum of **22** (500 MHz, $CDCl_3$)

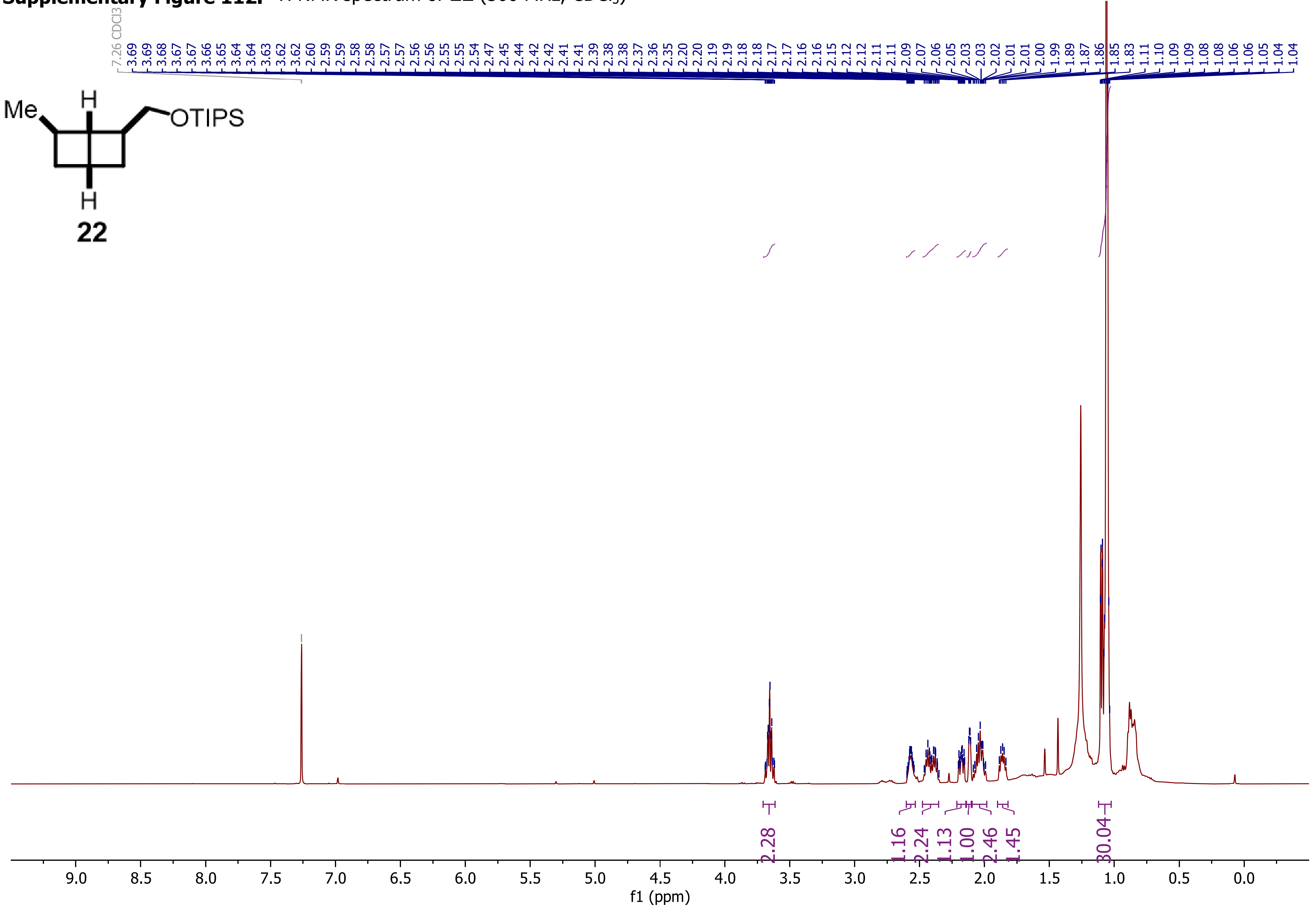

Supplementary Figure 113. ^{13}C NMR spectrum of **22** (126 MHz, $CDCl_3$)

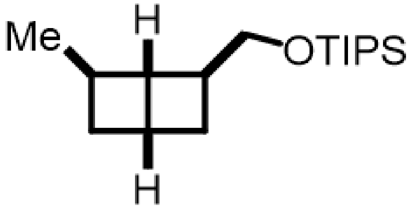

22

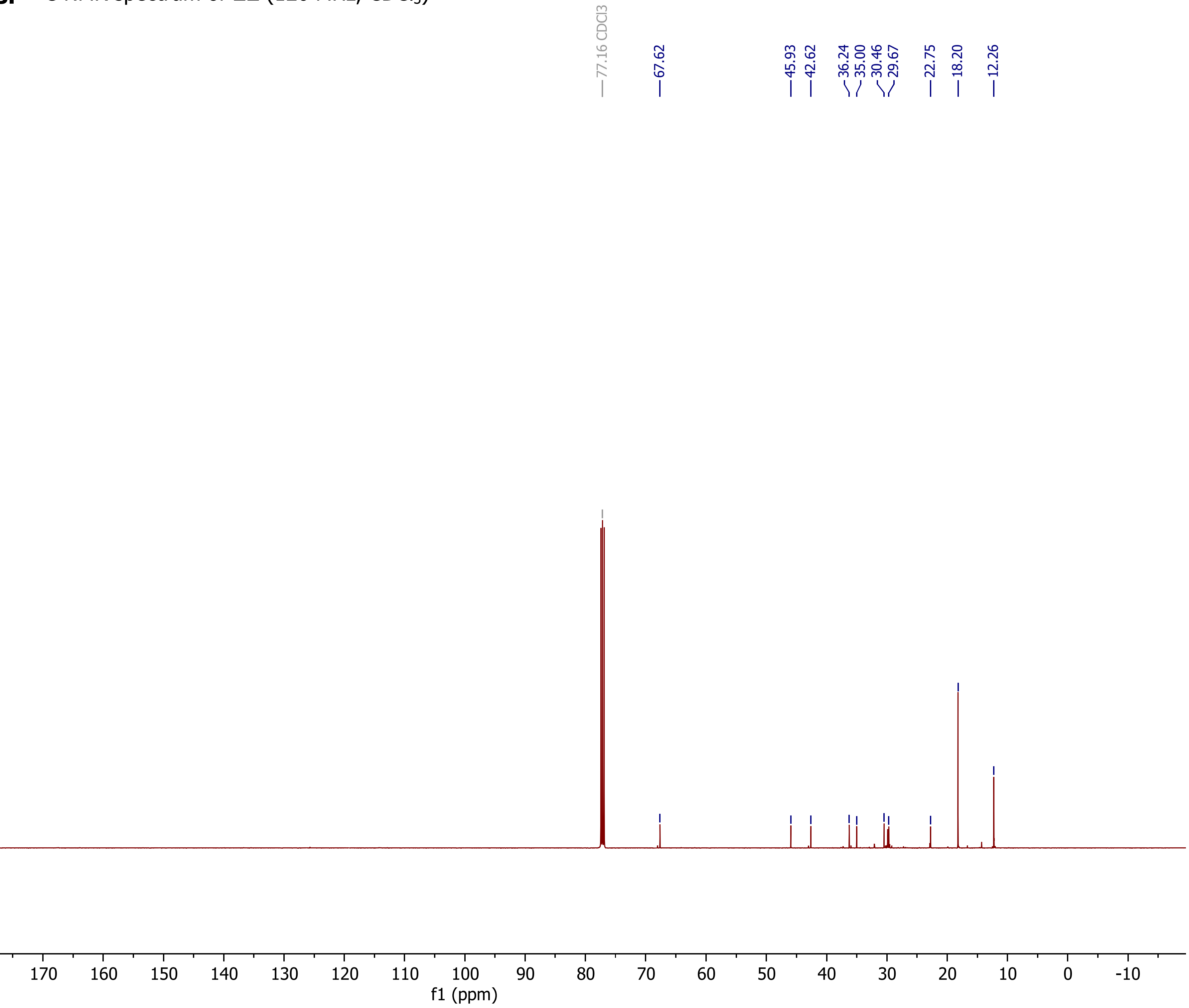

Supplementary Figure 114. ^{1}H NMR spectrum of **23** (500 MHz, $CDCl_3$)

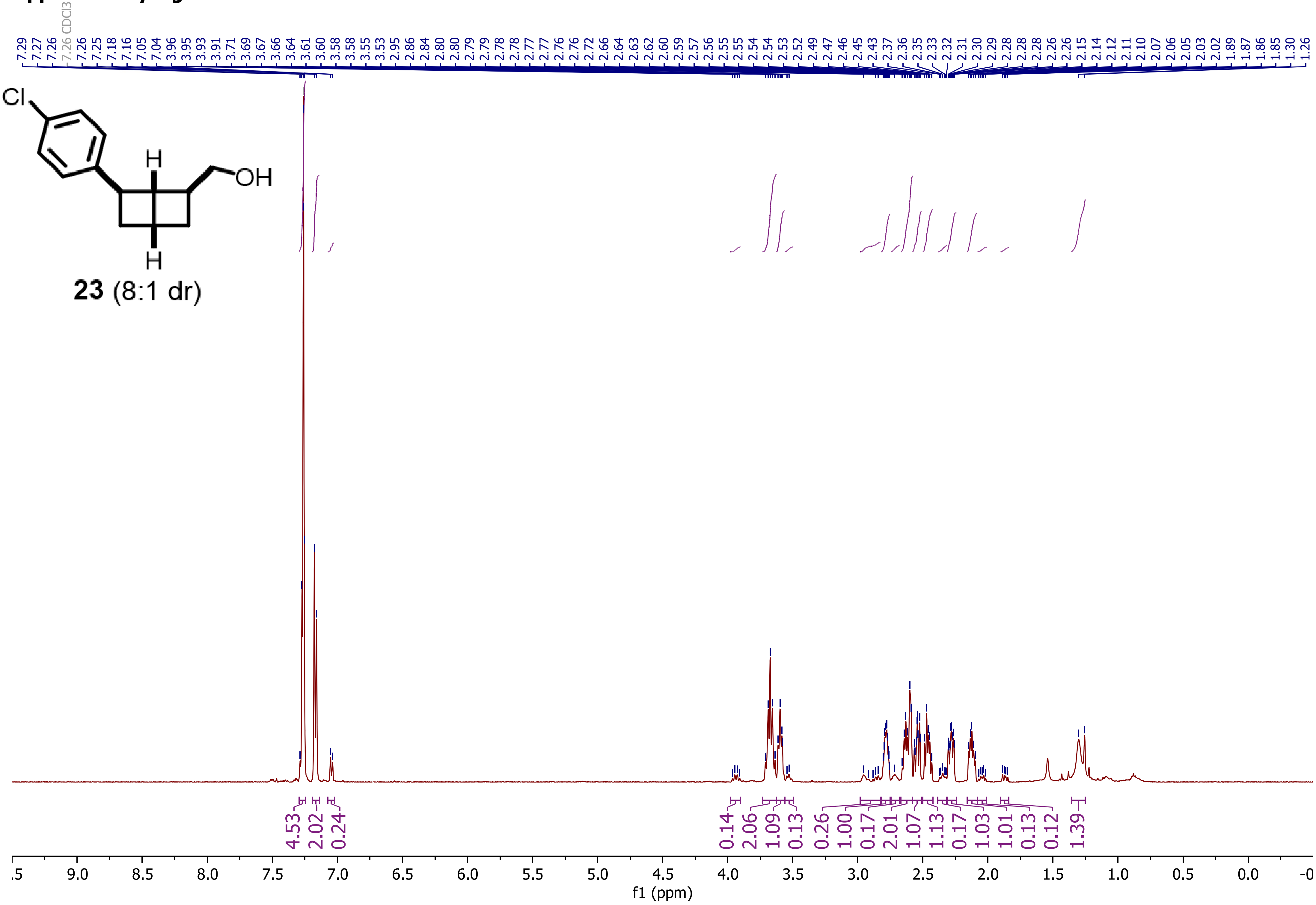

Supplementary Figure 115. ^{13}C NMR spectrum of **23** (126 MHz, $CDCl_3$)

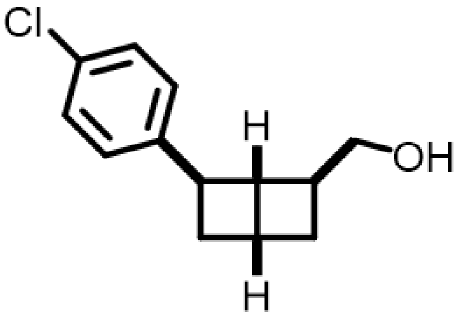

23 (8:1 dr)

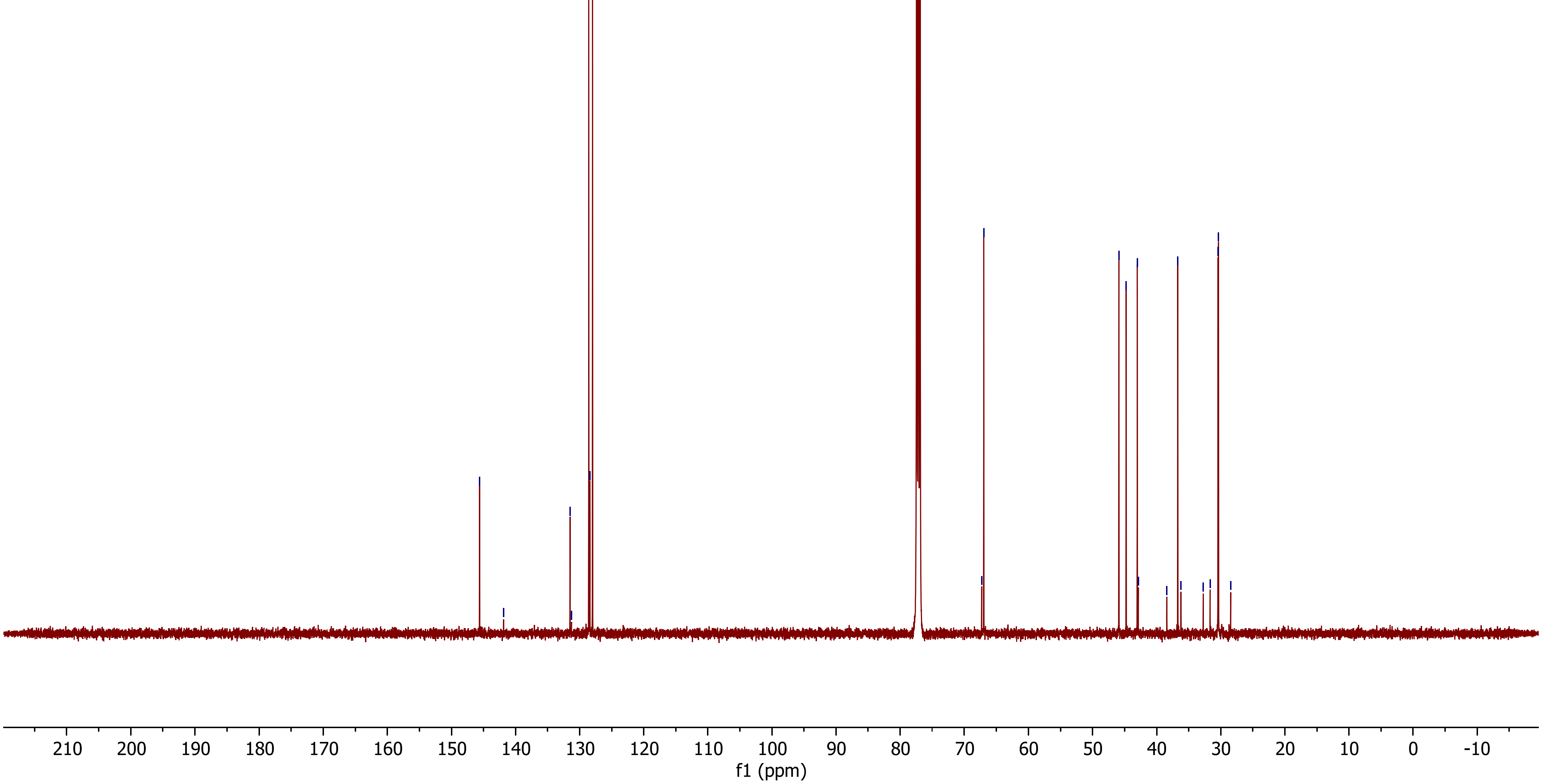

Supplementary Figure 116. ^{1}H NMR spectrum of **24** (500 MHz, $CDCl_3$)

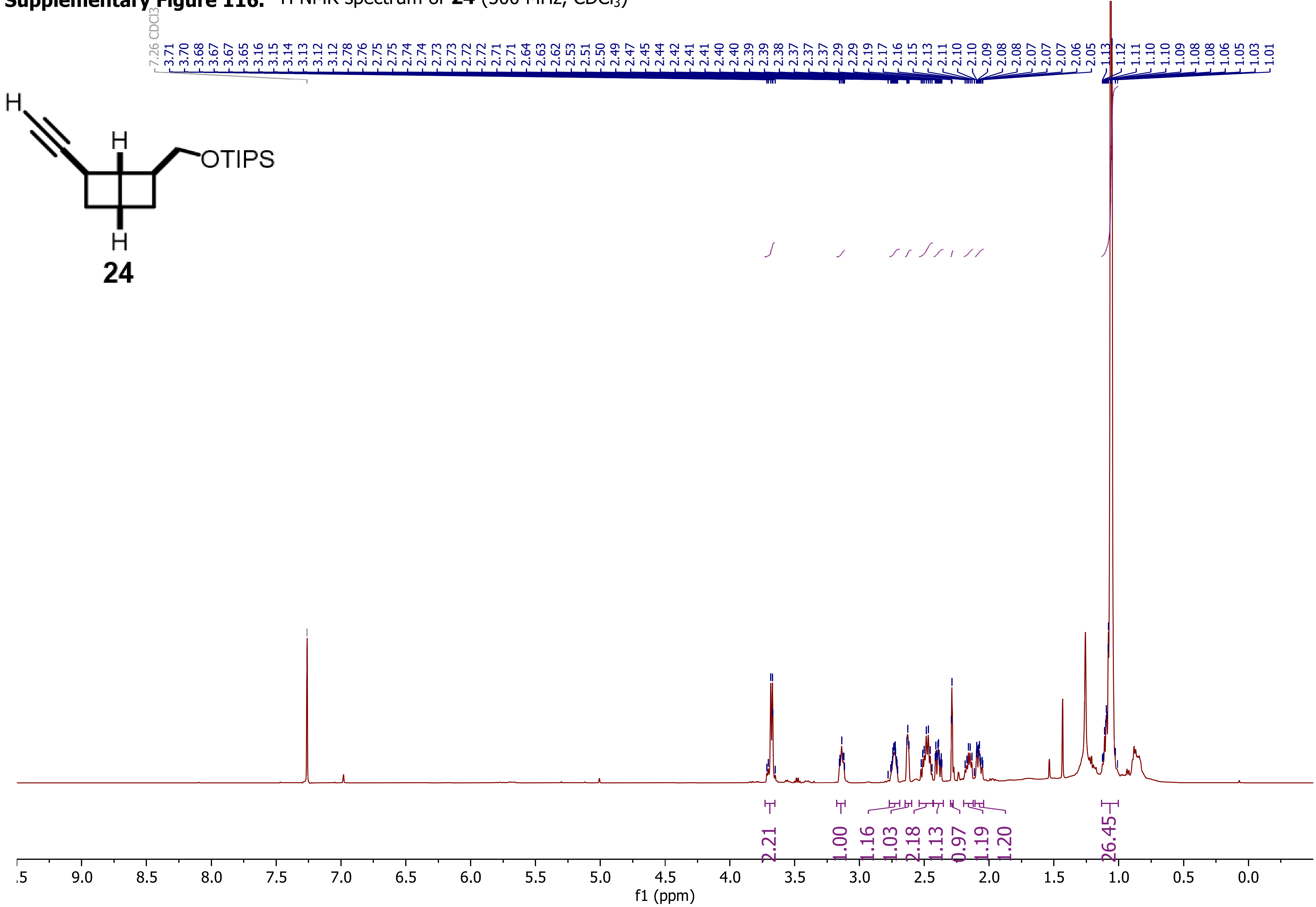

Supplementary Figure 117. ^{13}C NMR spectrum of **24** (126 MHz, $CDCl_3$)

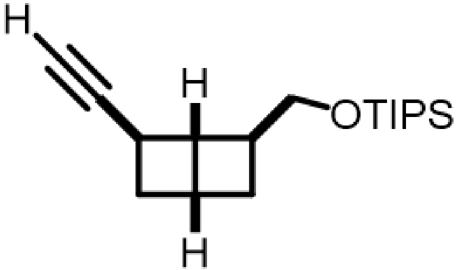

24

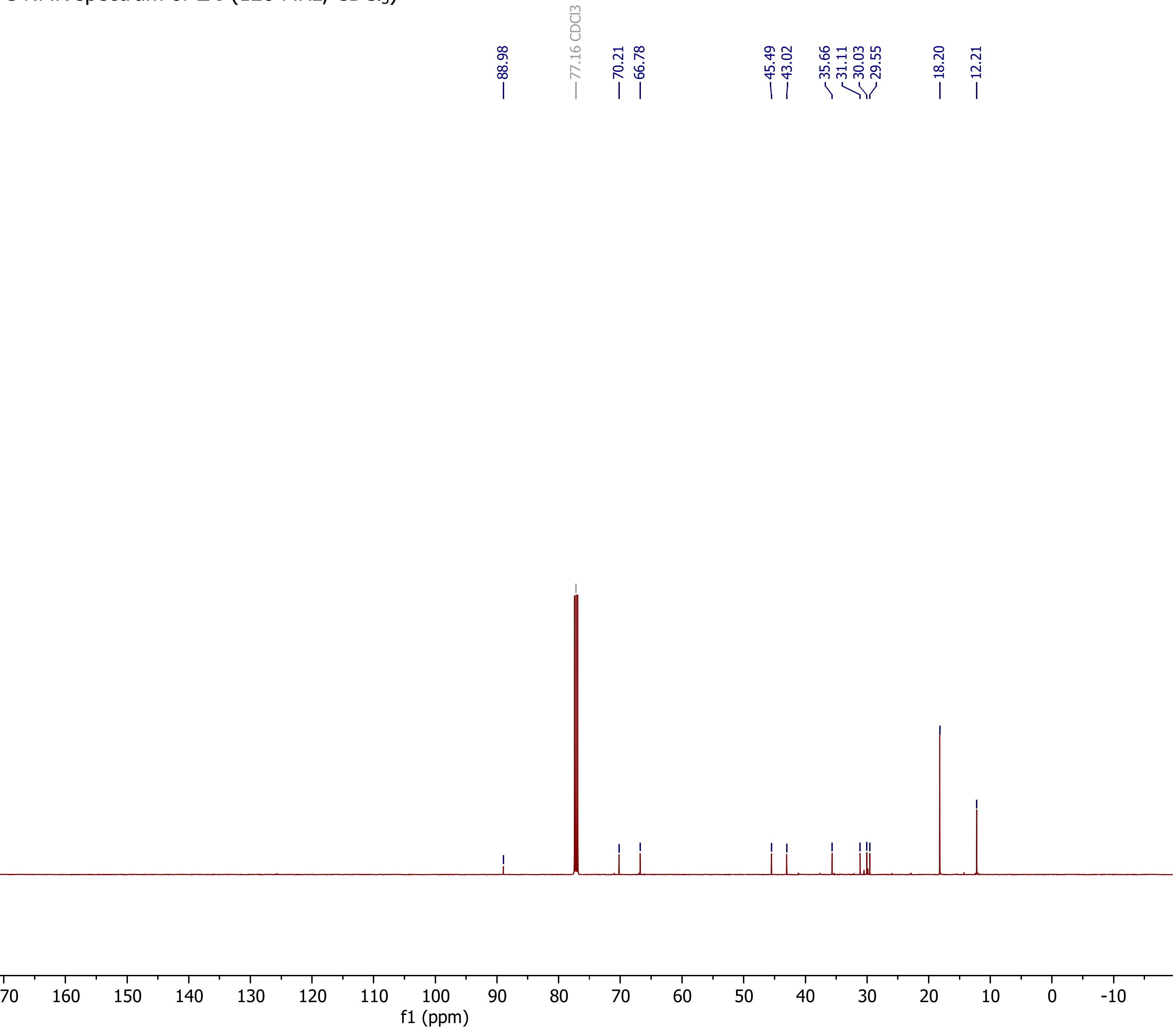

Supplementary Figure 118. COSY NMR spectrum of **24** (500 MHz, $CDCl_3$)

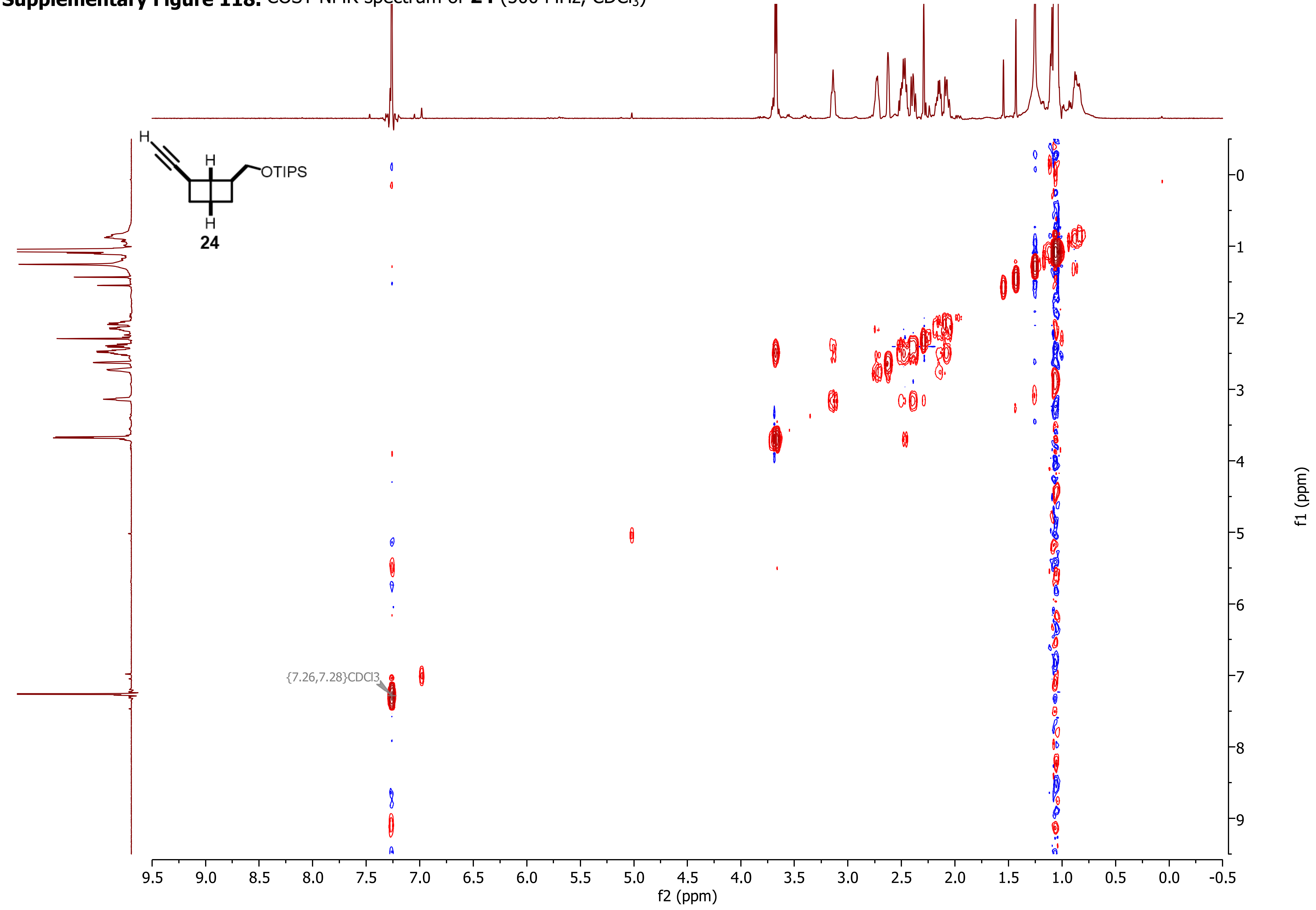

Supplementary Figure 119. NOESY NMR spectrum of **24** (500 MHz, $CDCl_3$)

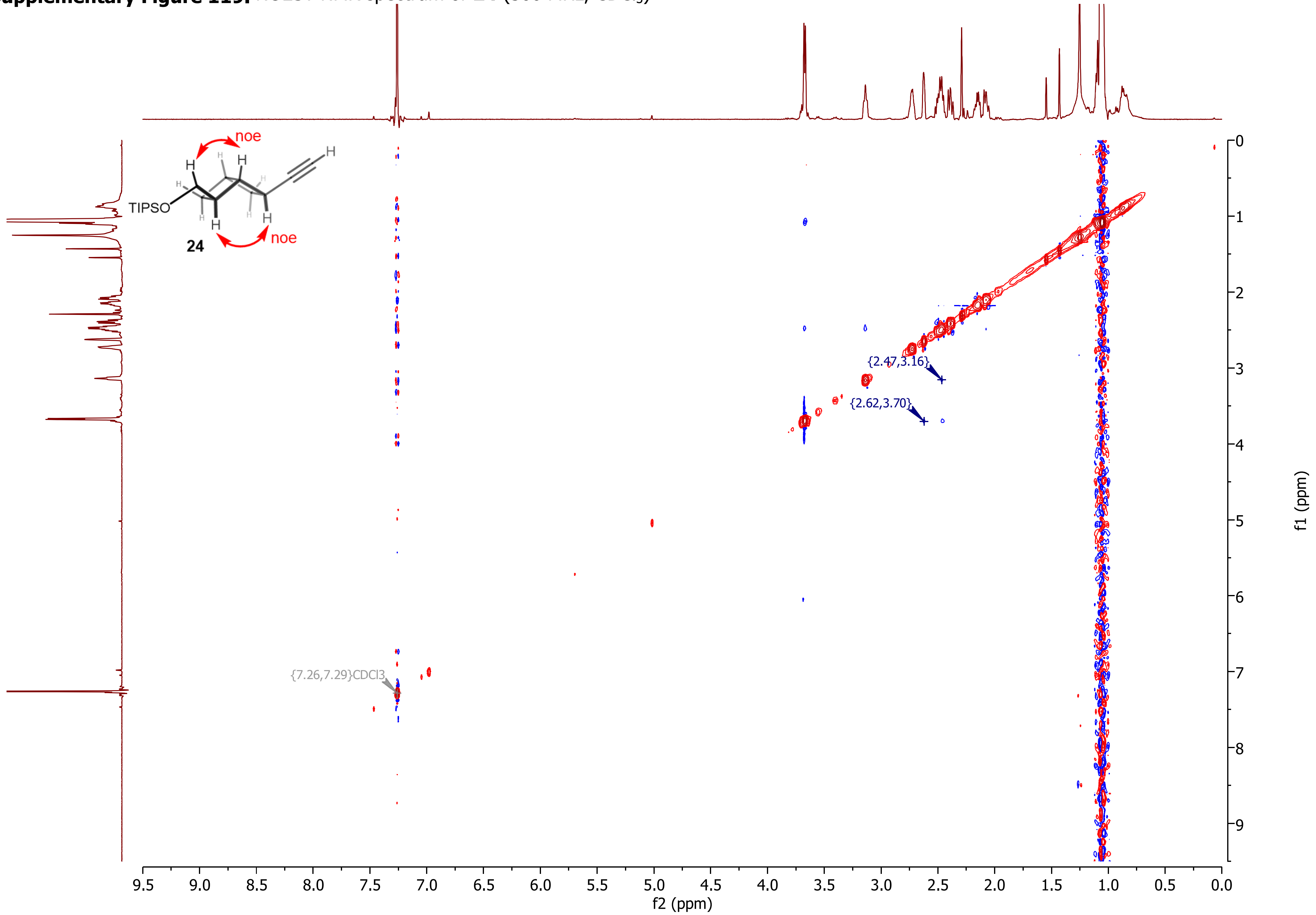

Supplementary Figure 120. ^{1}H NMR spectrum of **SI-45** (500 MHz, $CDCl_3$)

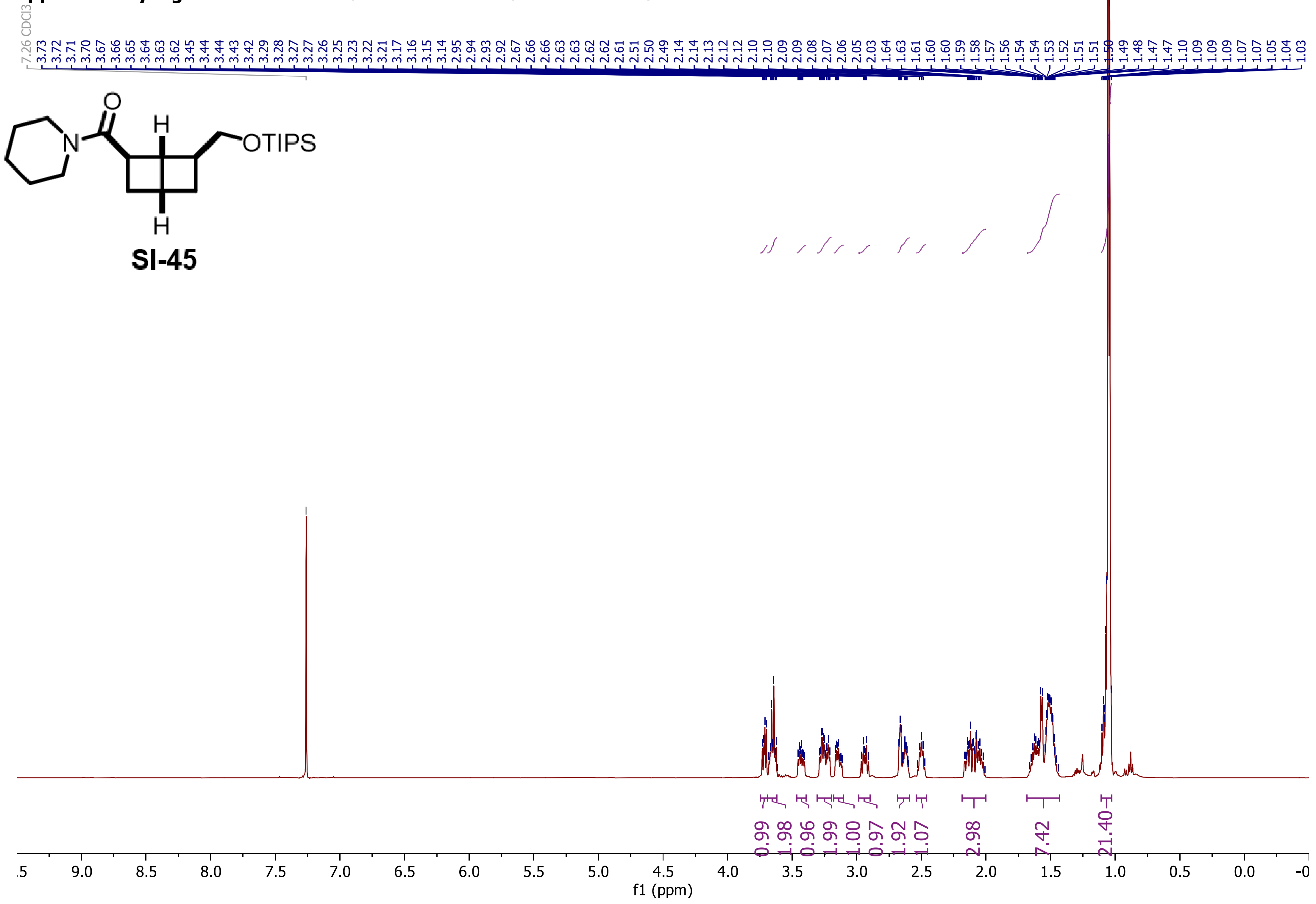

Supplementary Figure 121. ^{13}C NMR spectrum of **SI-45** (126 MHz, $CDCl_3$)

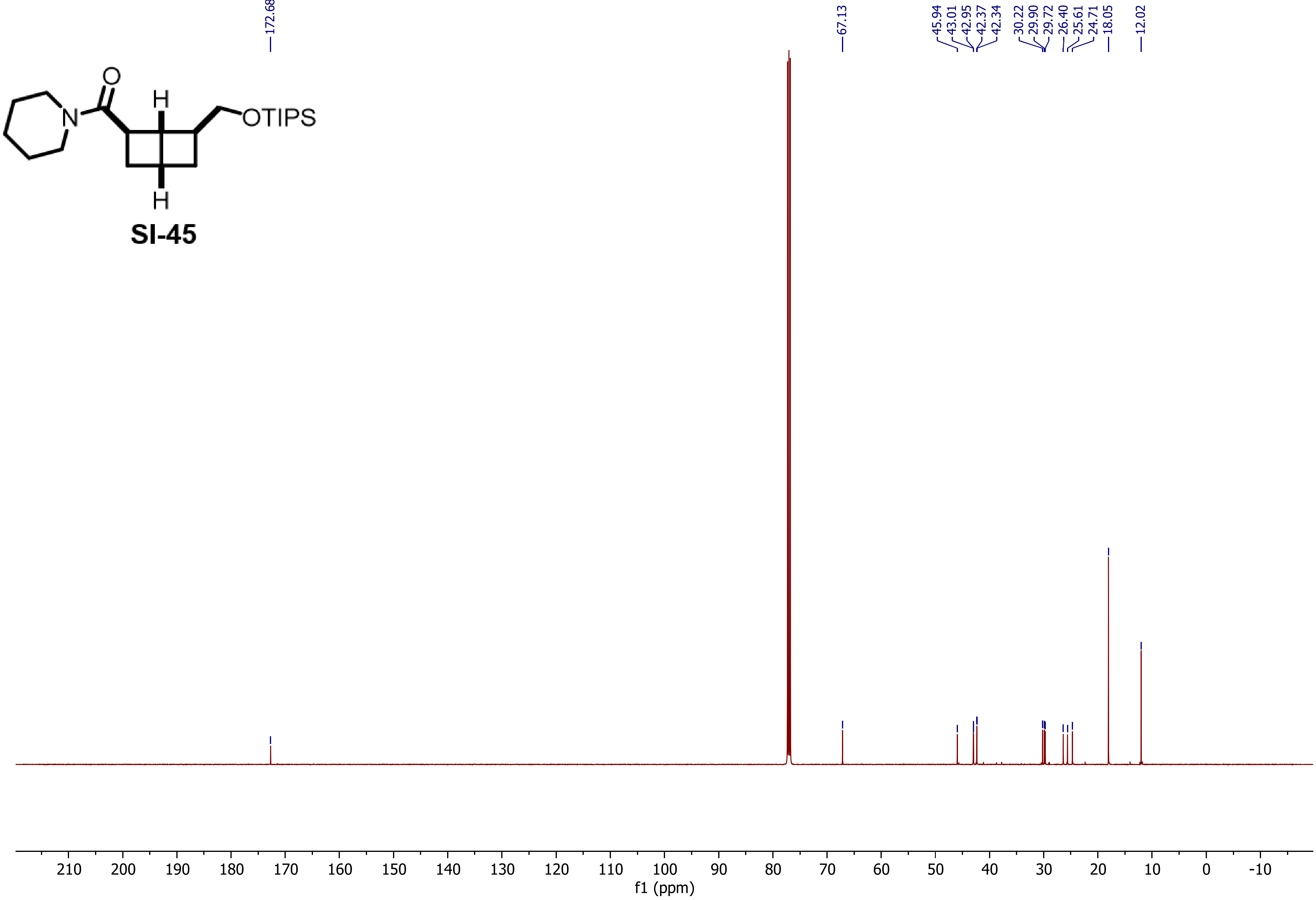

Supplementary Figure 122. ^{1}H NMR spectrum of **35** (500 MHz, $CDCl_3$)

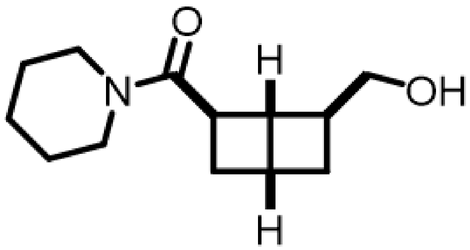

35

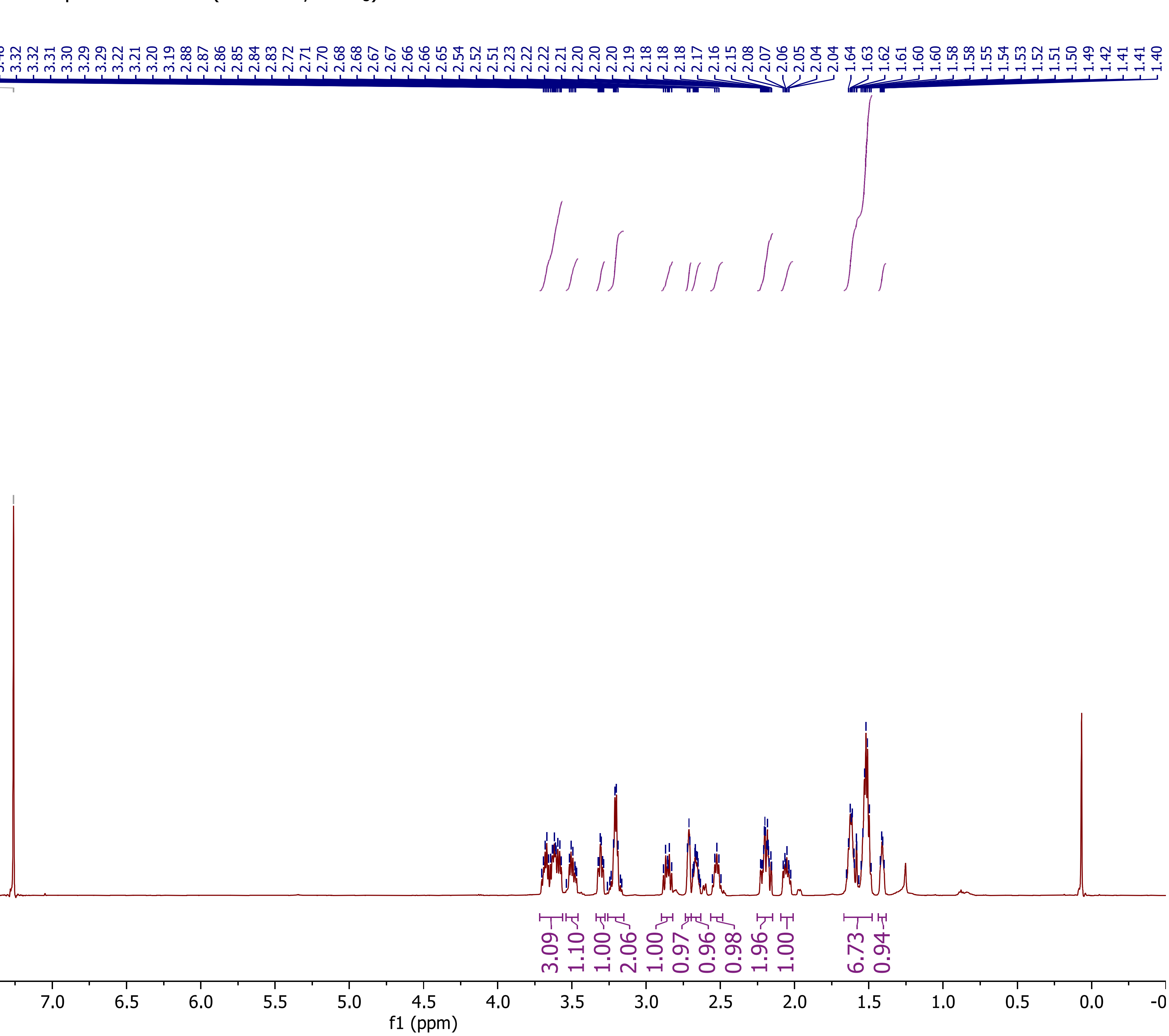

Supplementary Figure 123. ^{13}C NMR spectrum of **35** (126 MHz, $CDCl_3$)

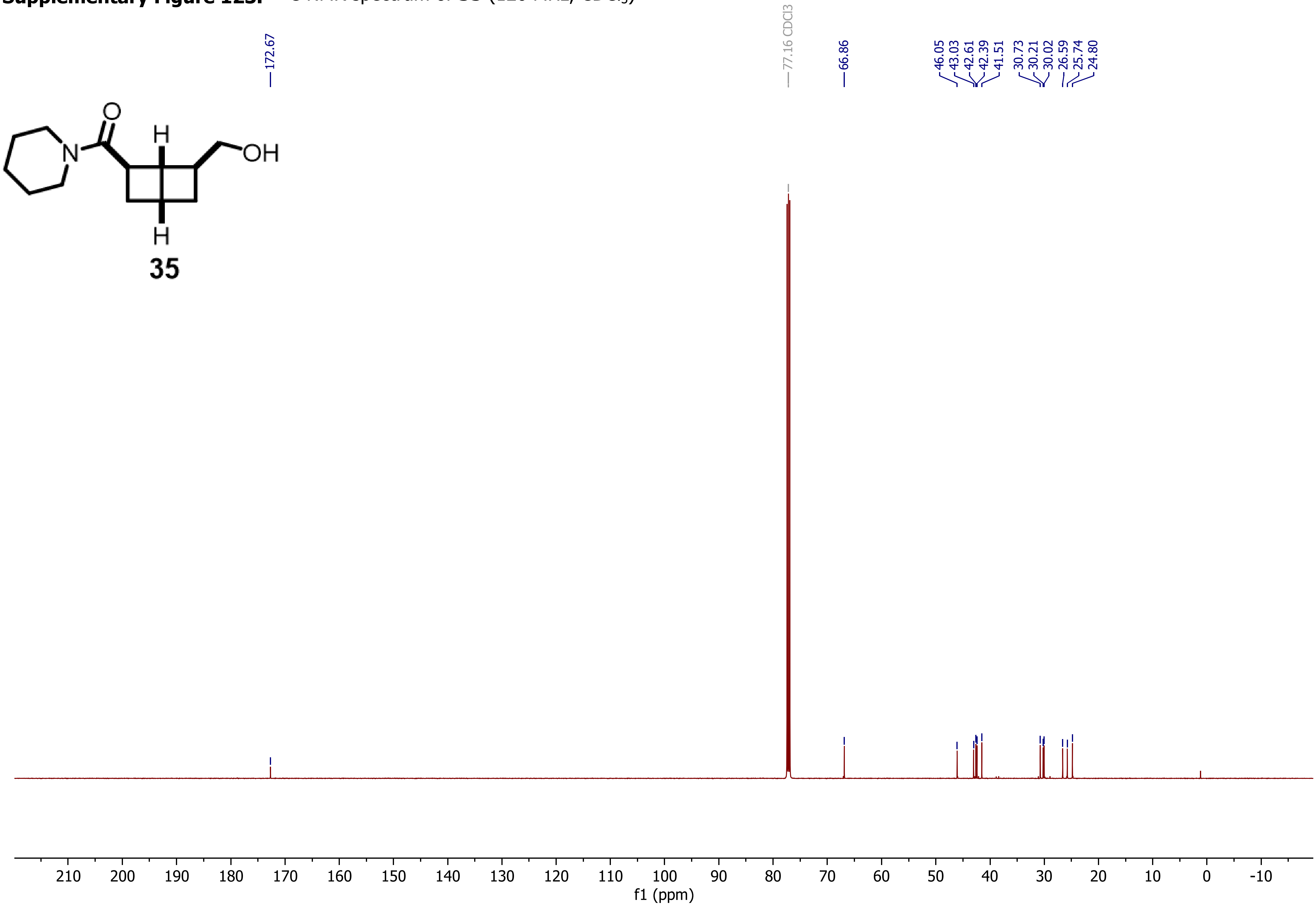

Supplementary Figure 124. ^{1}H NMR spectrum of **SI-46** (500 MHz, $CDCl_3$)

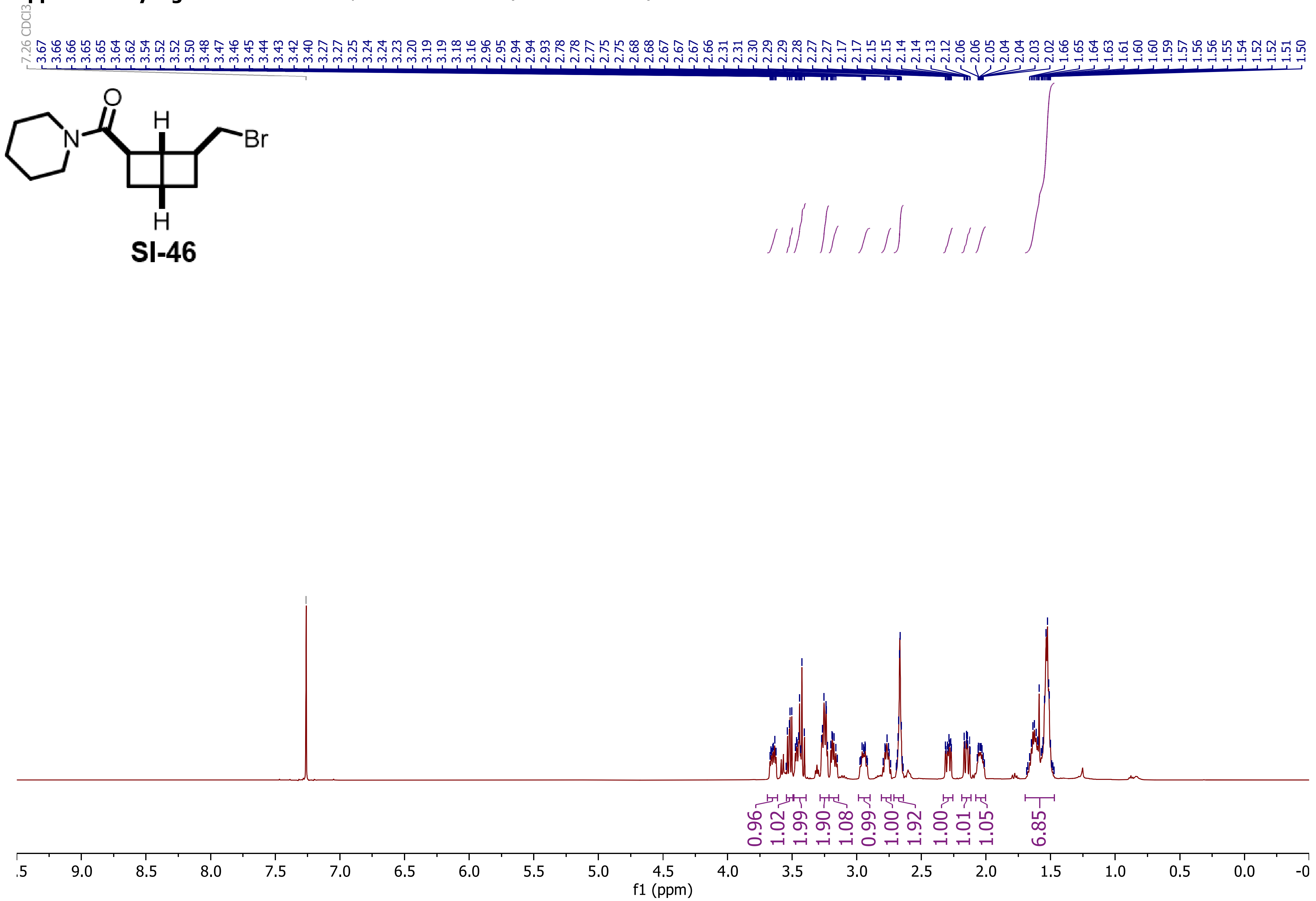

Supplementary Figure 125. ^{13}C NMR spectrum of **SI-46** (126 MHz, $CDCl_3$)

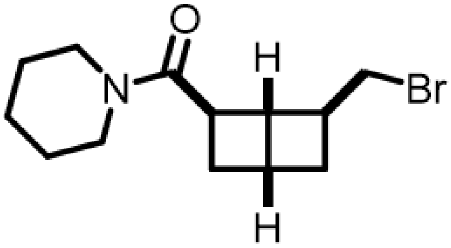

SI-46

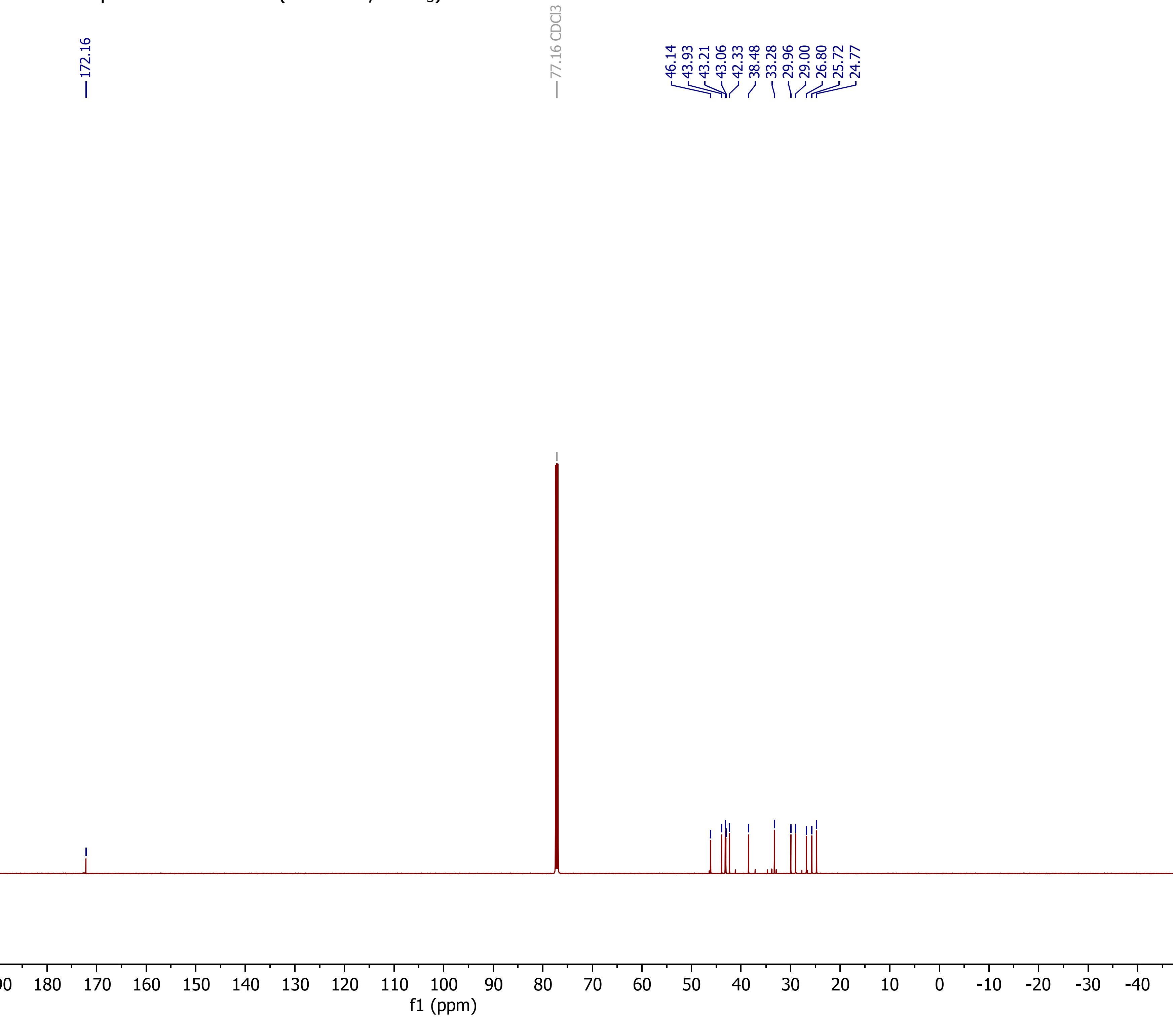

Supplementary Figure 126. ^{1}H NMR spectrum of **25** (500 MHz, $CDCl_3$)

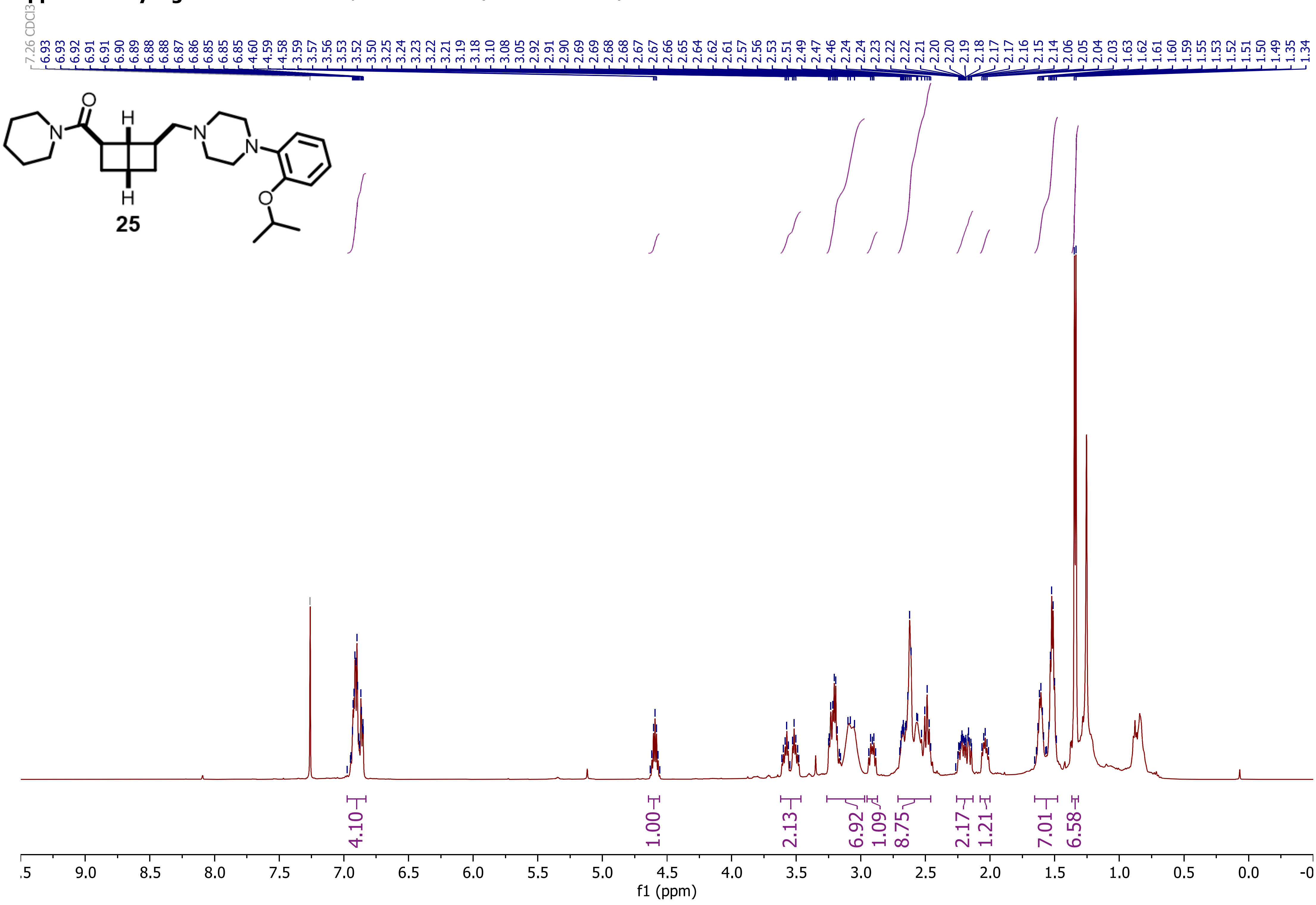

Supplementary Figure 127. ^{13}C NMR spectrum of **25** (126 MHz, $CDCl_3$)

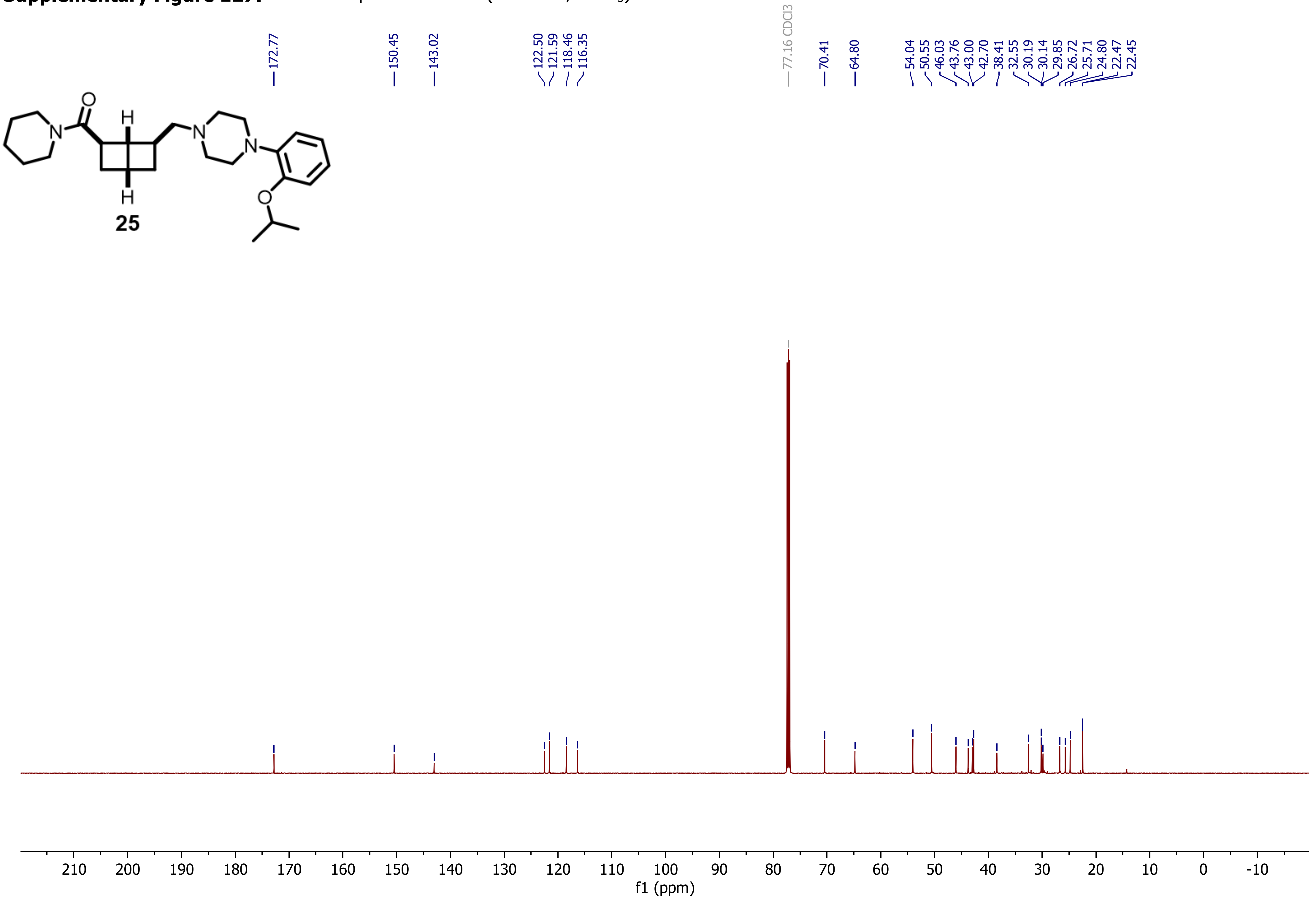

Supplementary Figure 138. ^{1}H NMR spectrum of **27** (400 MHz, CD_3OD)

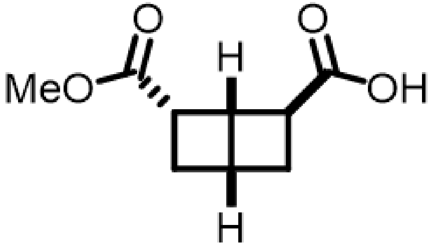

27 (6:1 dr)

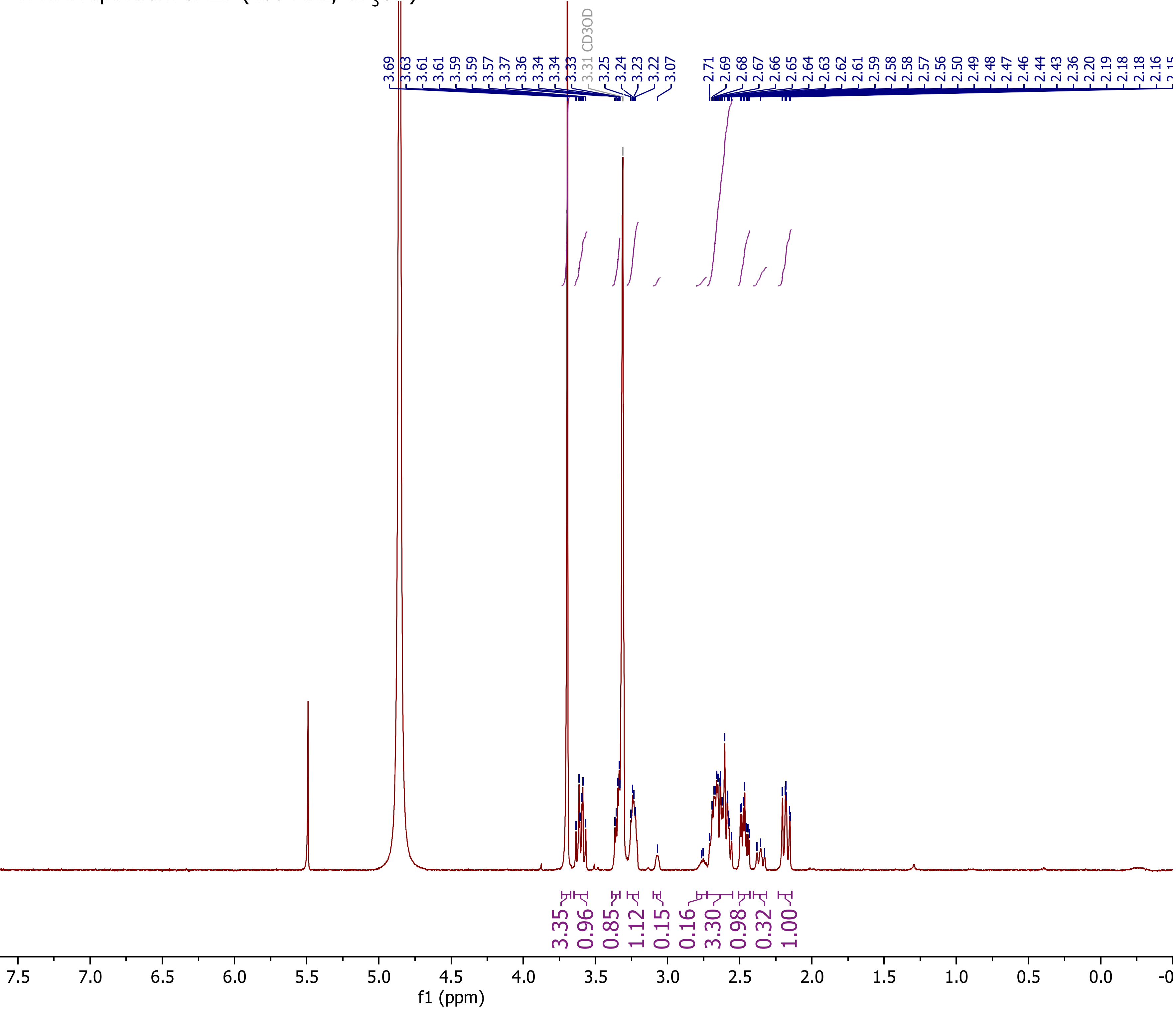

Supplementary Figure 139. ^{13}C NMR spectrum of **27** (126 MHz, CD_3OD)

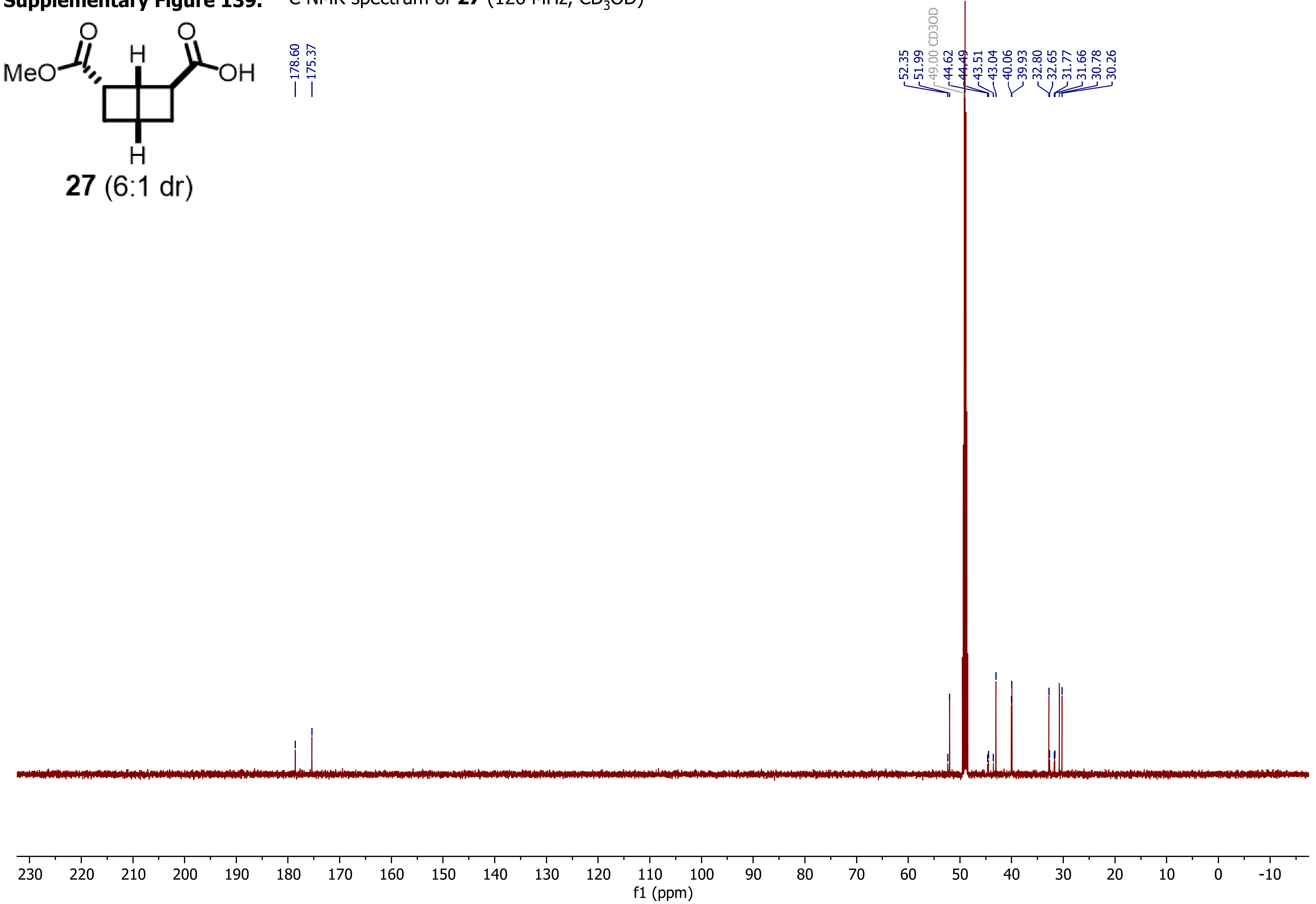

Supplementary Figure 140. ^{1}H NMR spectrum of **28** (500 MHz, $CDCl_3$)

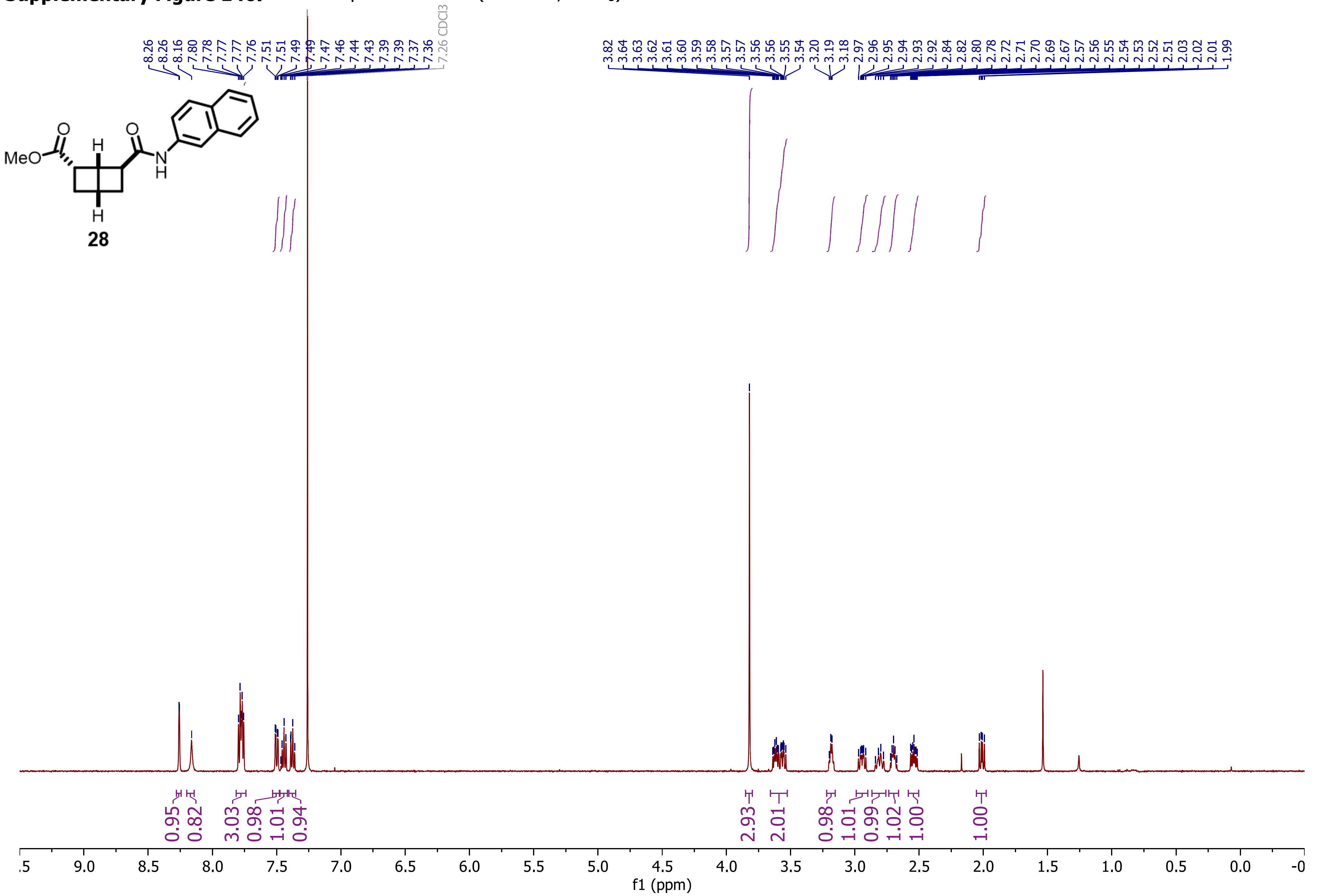

Supplementary Figure 141. ^{13}C NMR spectrum of **28** (126 MHz, $CDCl_3$)

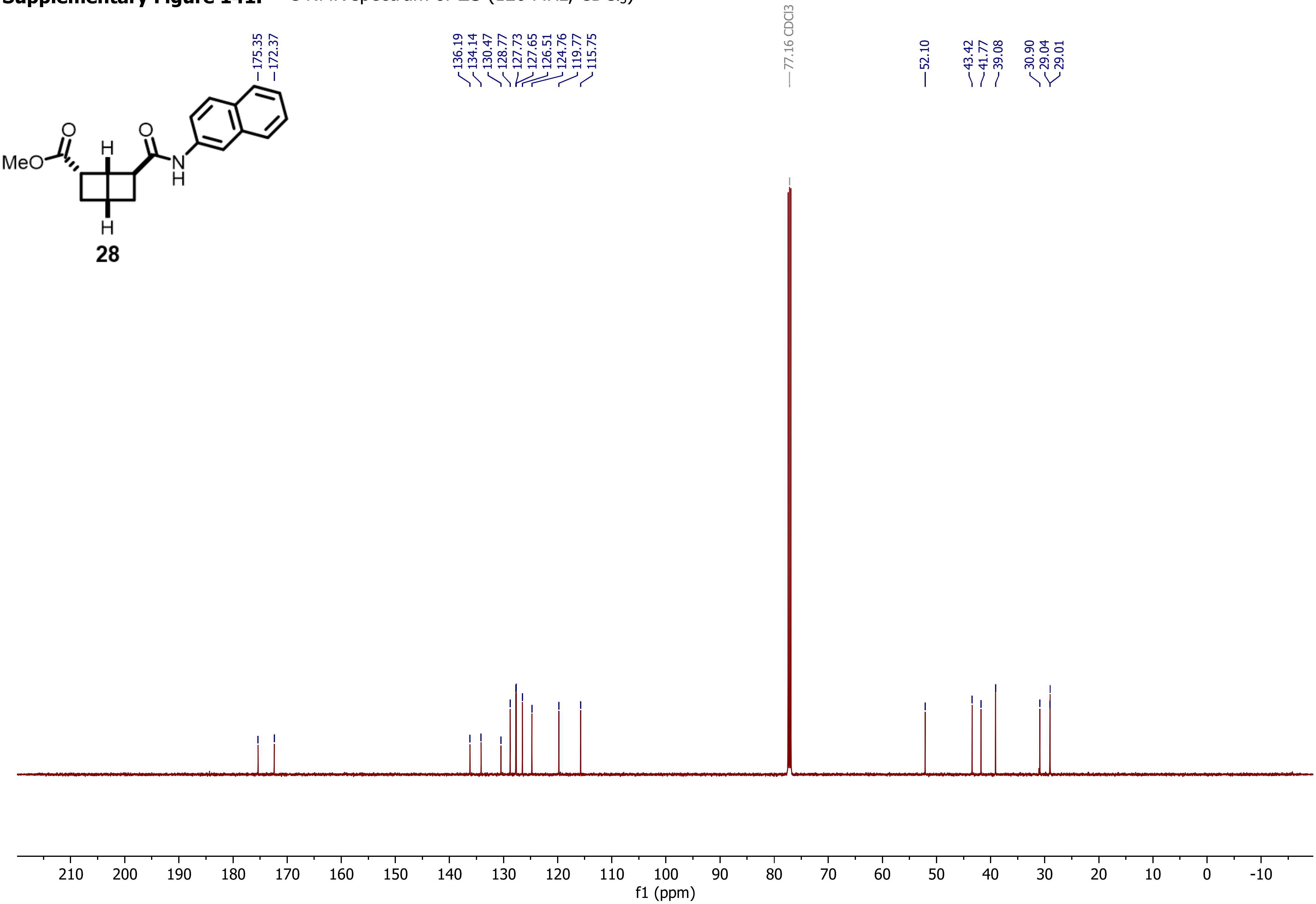

Supplementary Figure 142. ^{1}H NMR spectrum of **31** (500 MHz, $CDCl_3$)

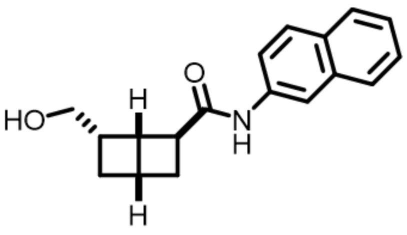
31

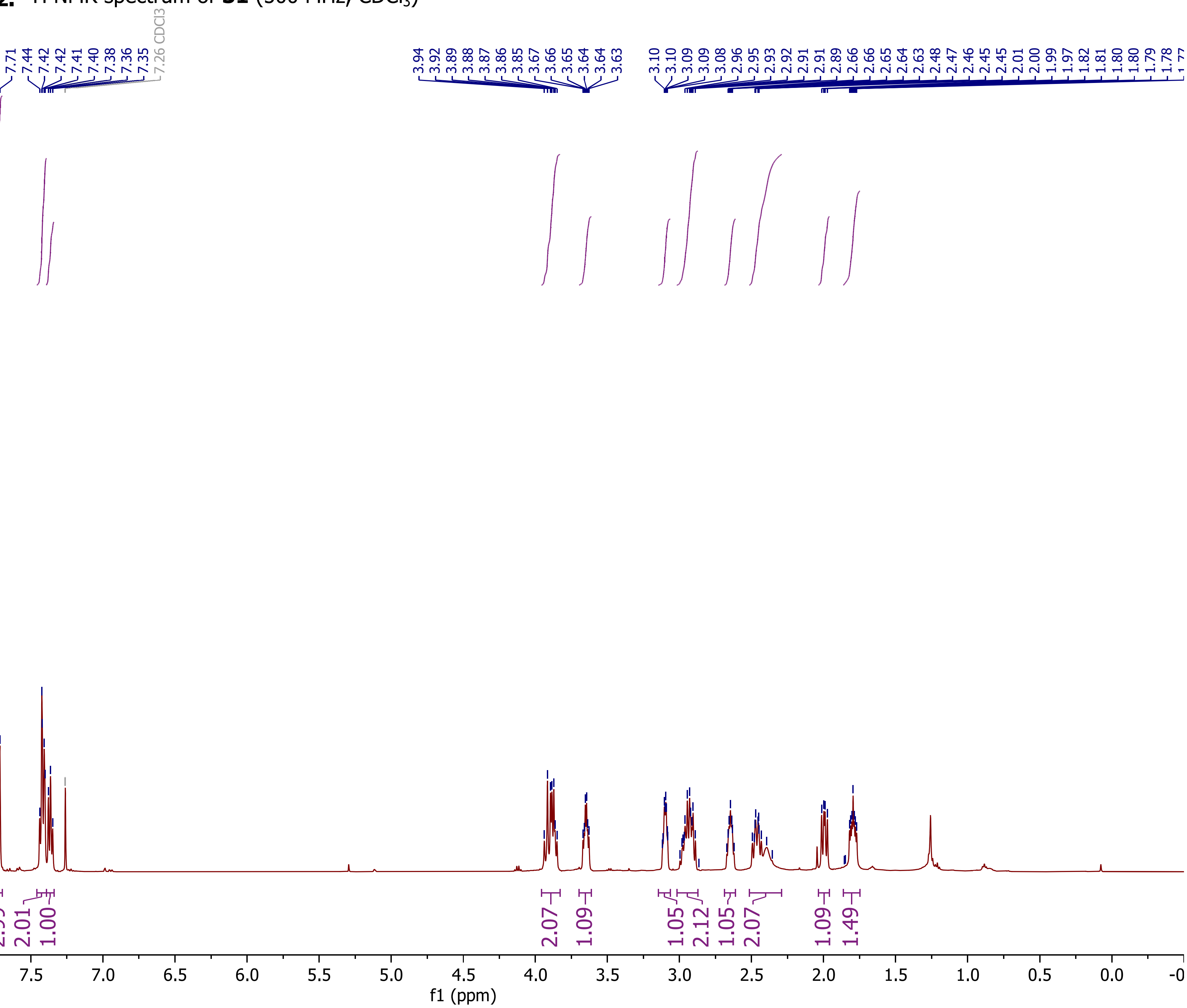

Supplementary Figure 143. ^{13}C NMR spectrum of **31** (126 MHz, $CDCl_3$)

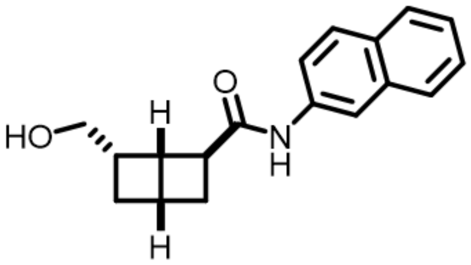

31

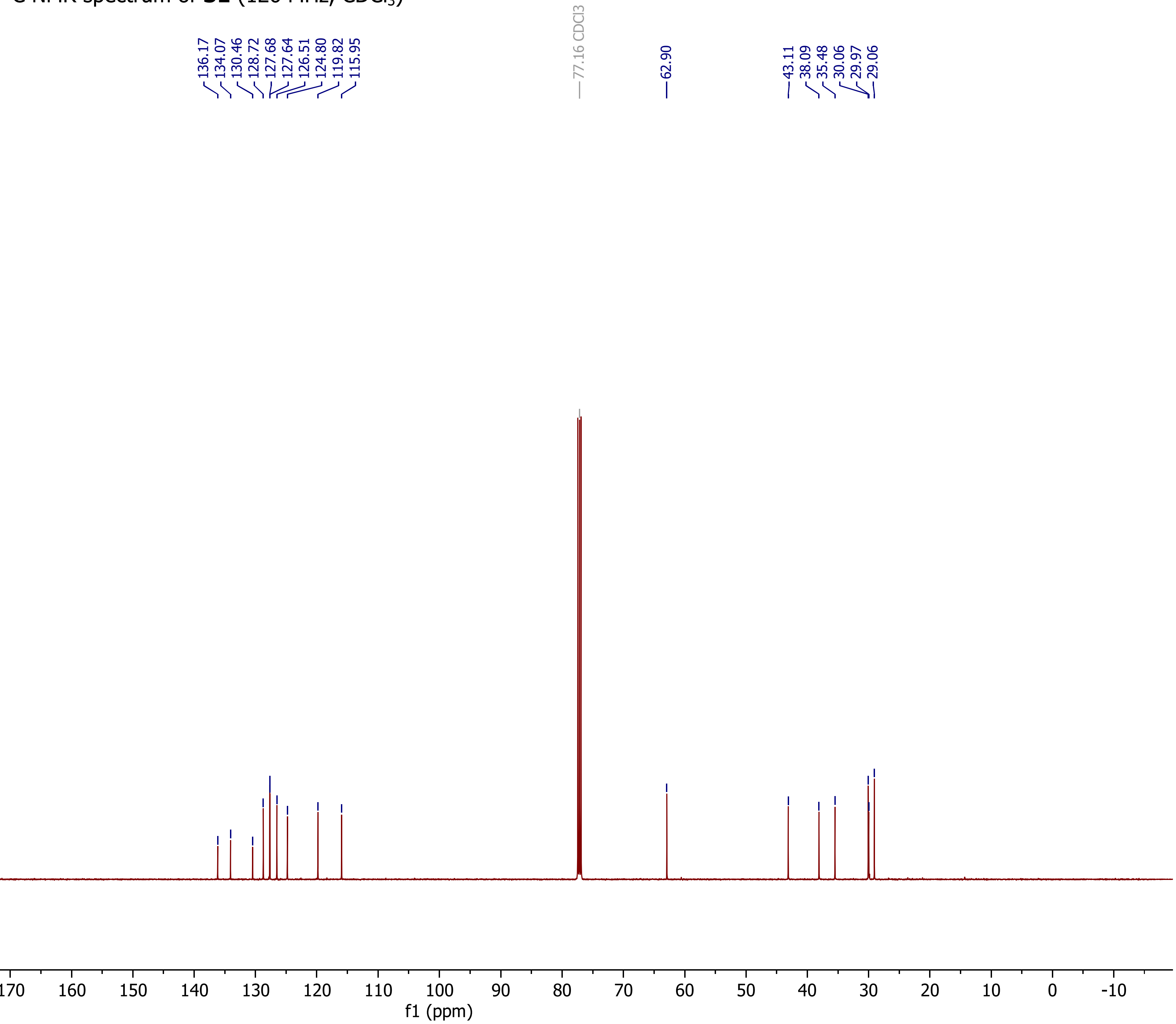

Supplementary Figure 144. ^1H NMR spectrum of **32** (500 MHz, $CDCl_3$)

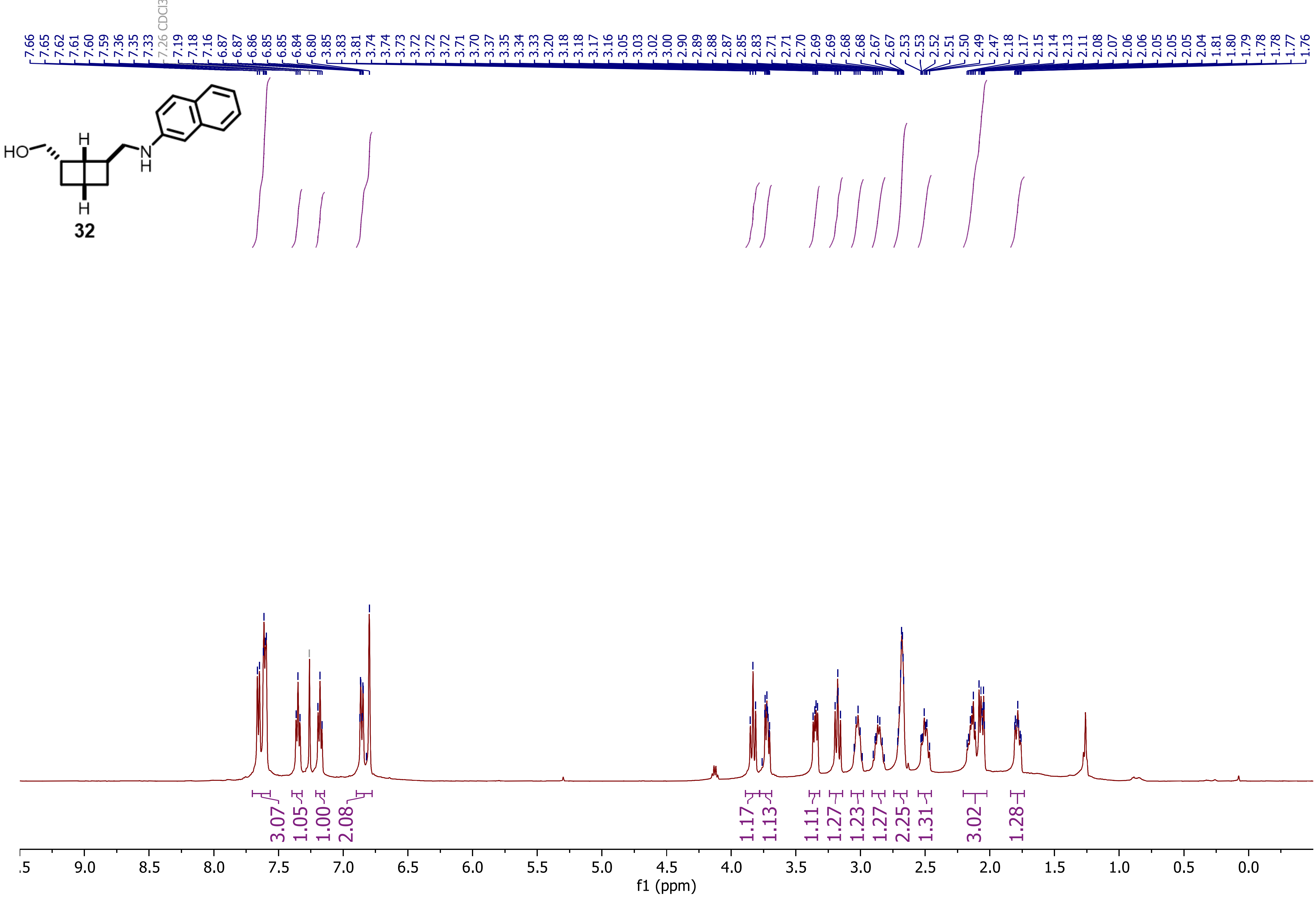

Supplementary Figure 145. ^{13}C NMR spectrum of **32** (126 MHz, $CDCl_3$)

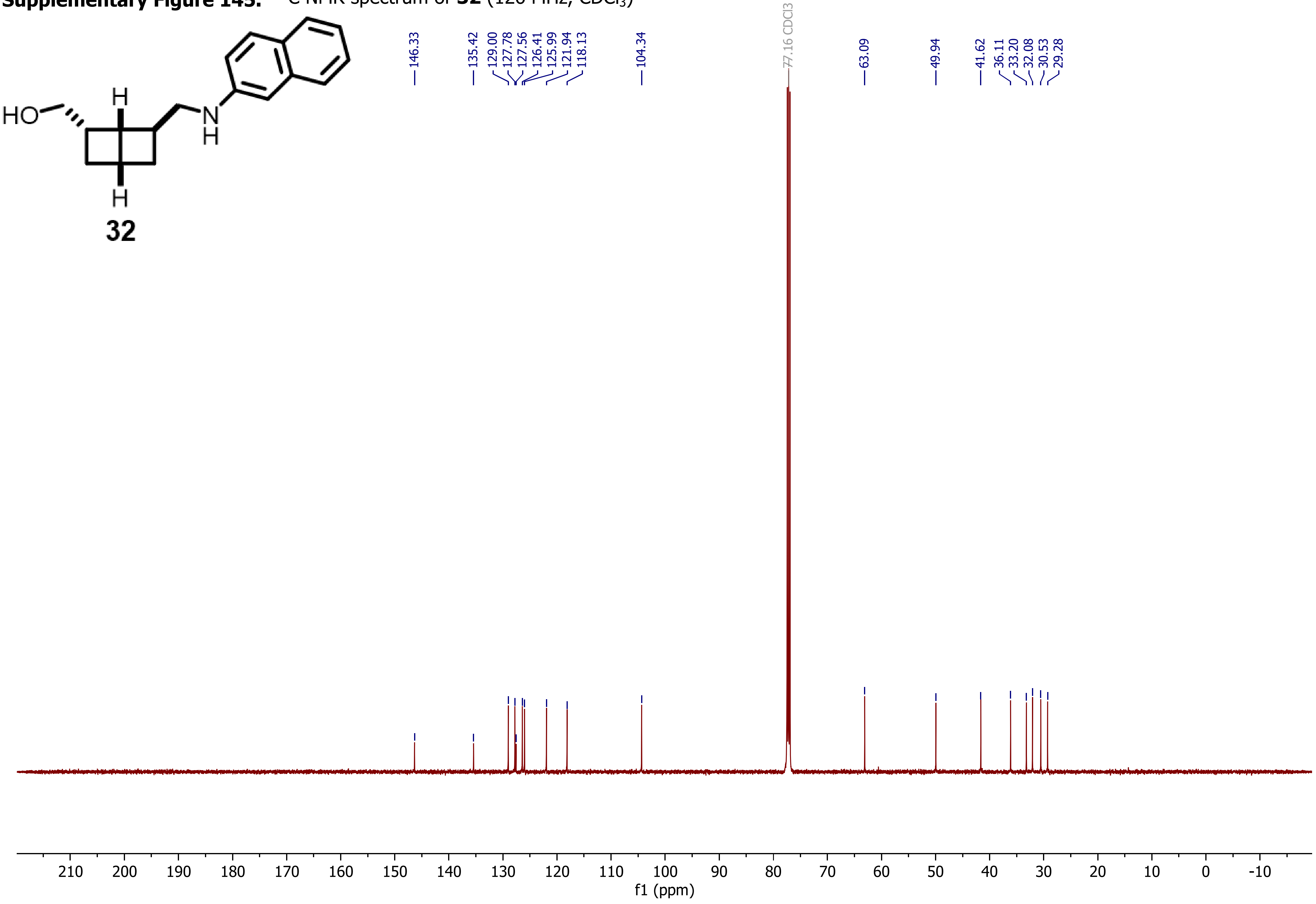

32

Supplementary Figure 146. ^{1}H NMR spectrum of **30** (400 MHz, $CDCl_3$)

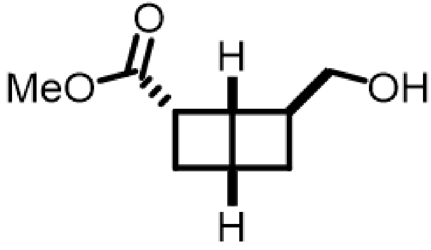

30 (6:1 dr)

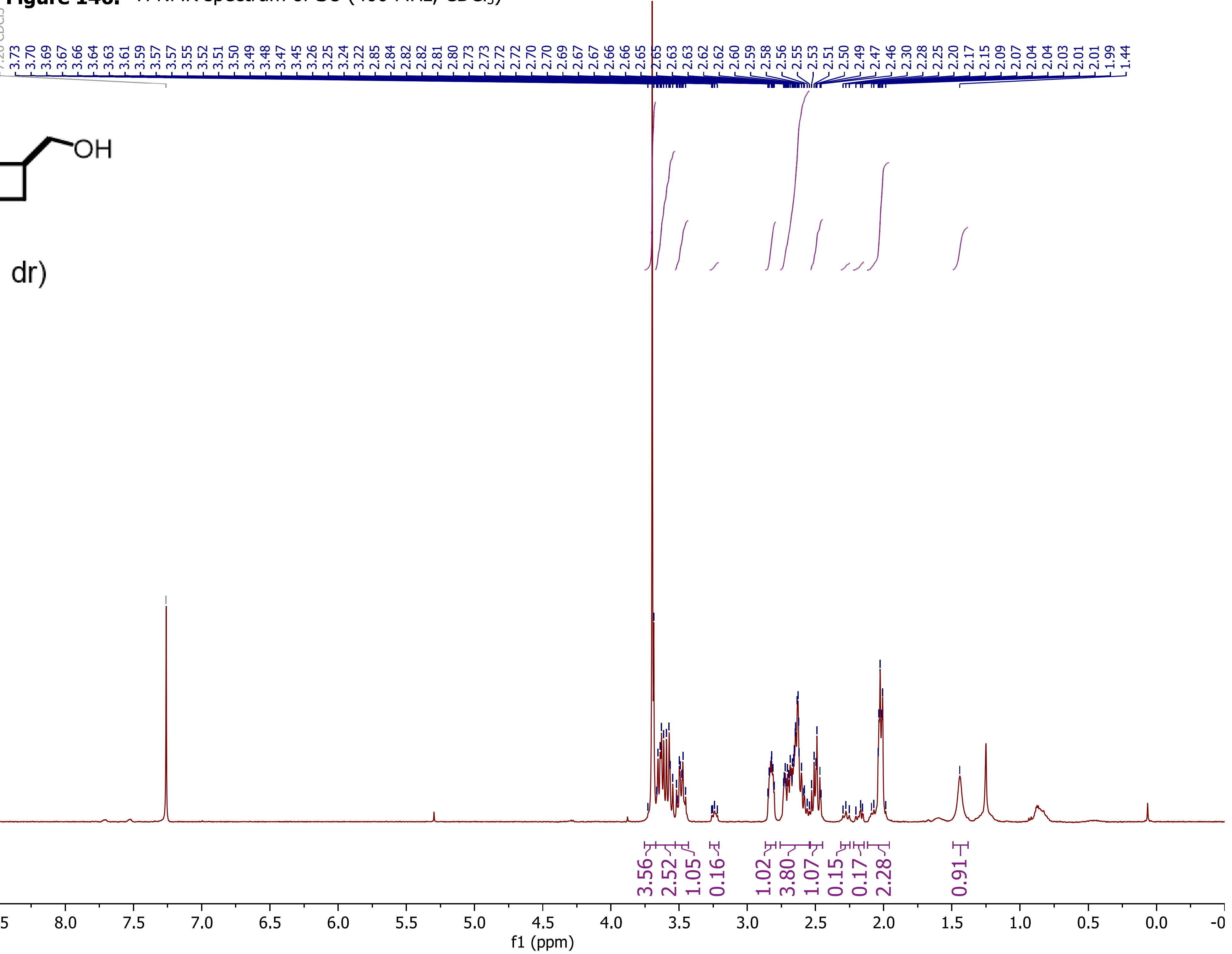

Supplementary Figure 147. ^{13}C NMR spectrum of **30** (126 MHz, $CDCl_3$)

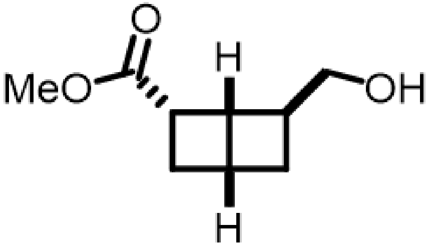

30 (6:1 dr)

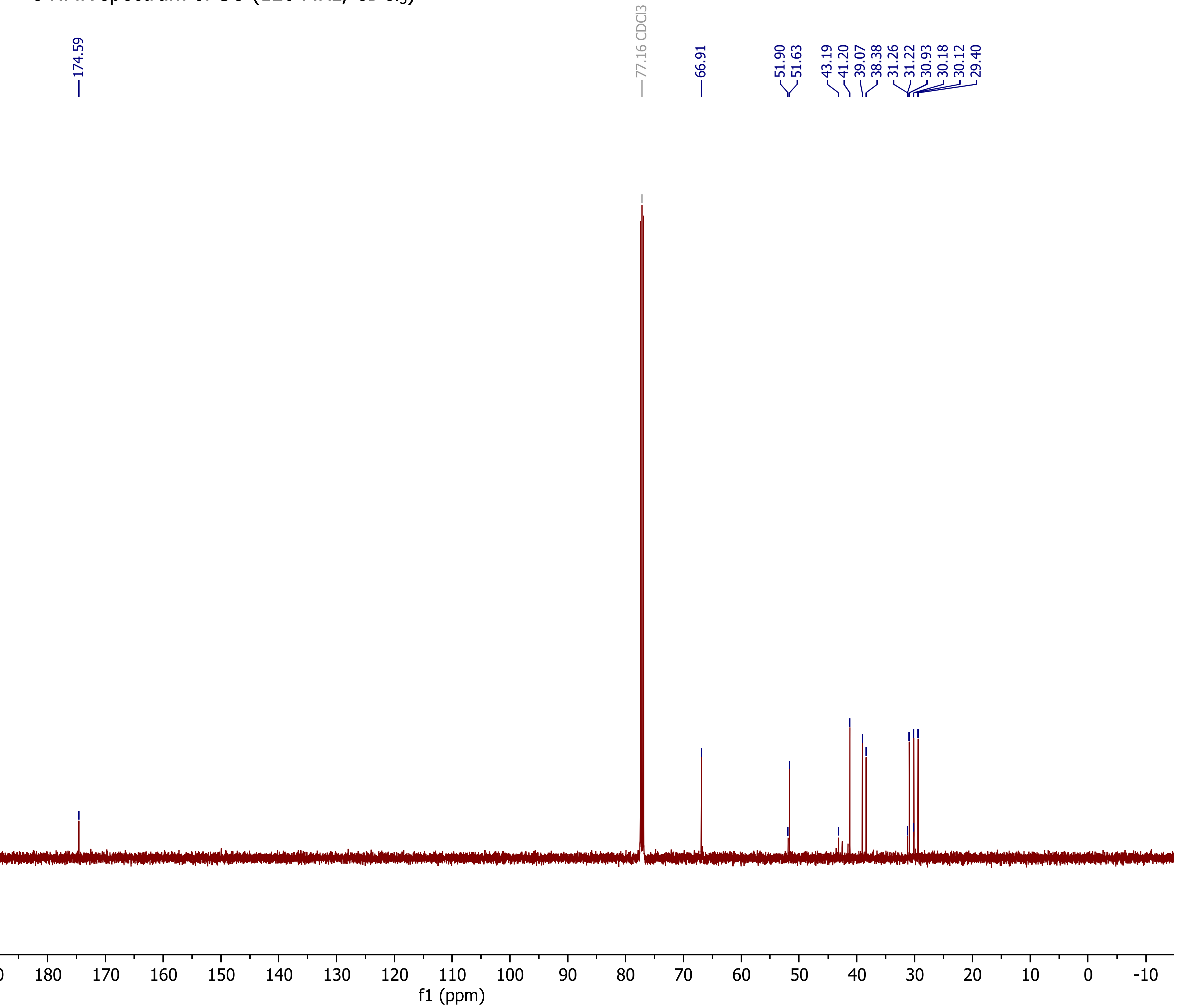

Supplementary Figure 148. ^{1}H NMR spectrum of **29** (500 MHz, C_6D_6, 47 °C)

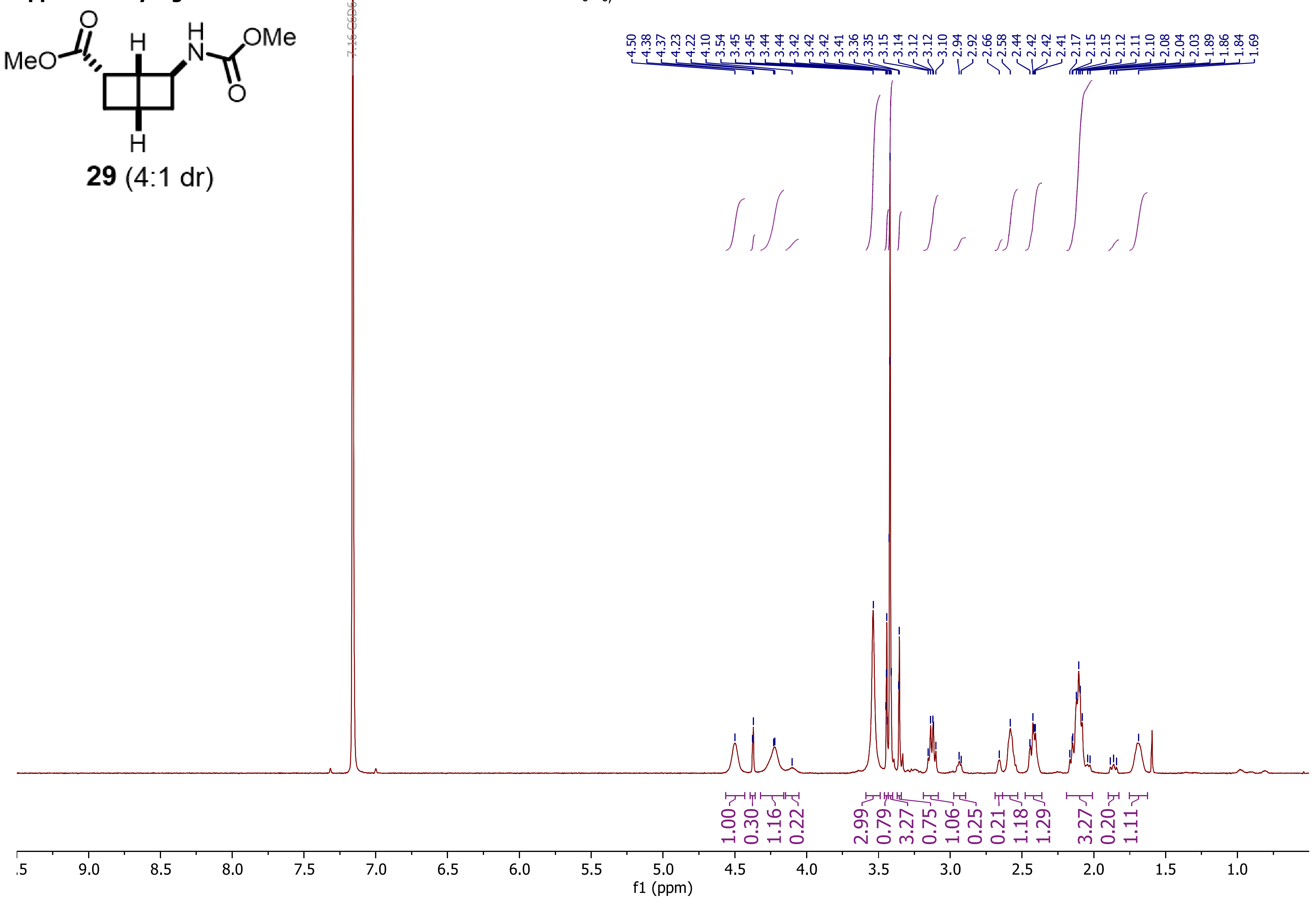

Supplementary Figure 149. ^{13}C NMR spectrum of **29** (126 MHz, C_6D_6, 47 °C)

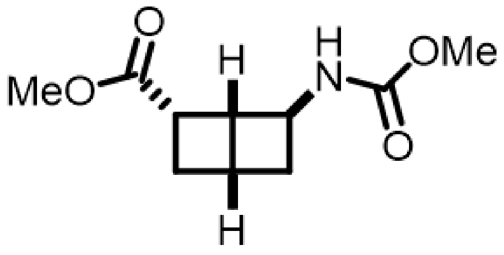

29 (4:1 dr)

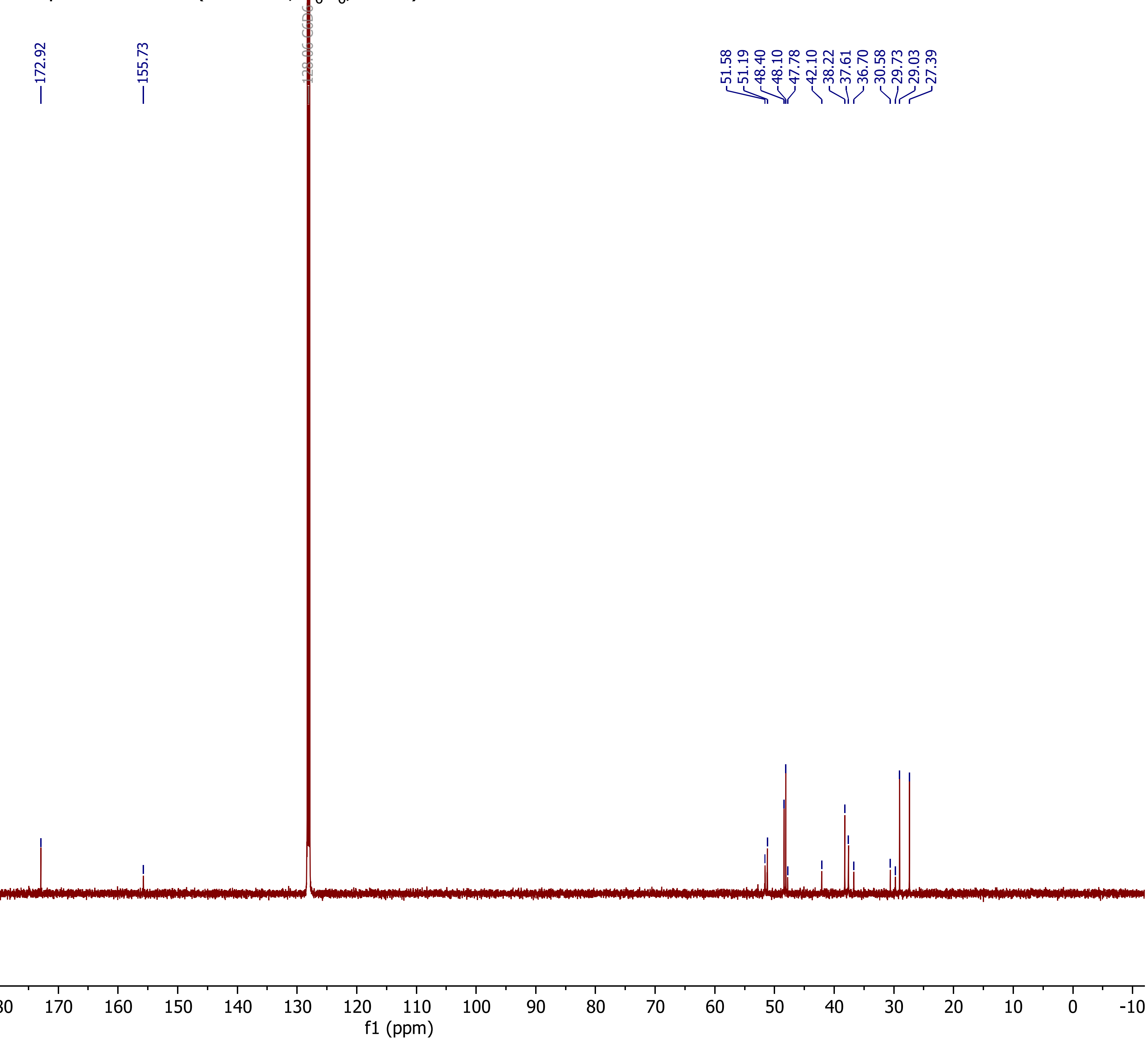

Supplementary Figure 150. ^{1}H NMR spectrum of **33** (400 MHz, $CDCl_3$)

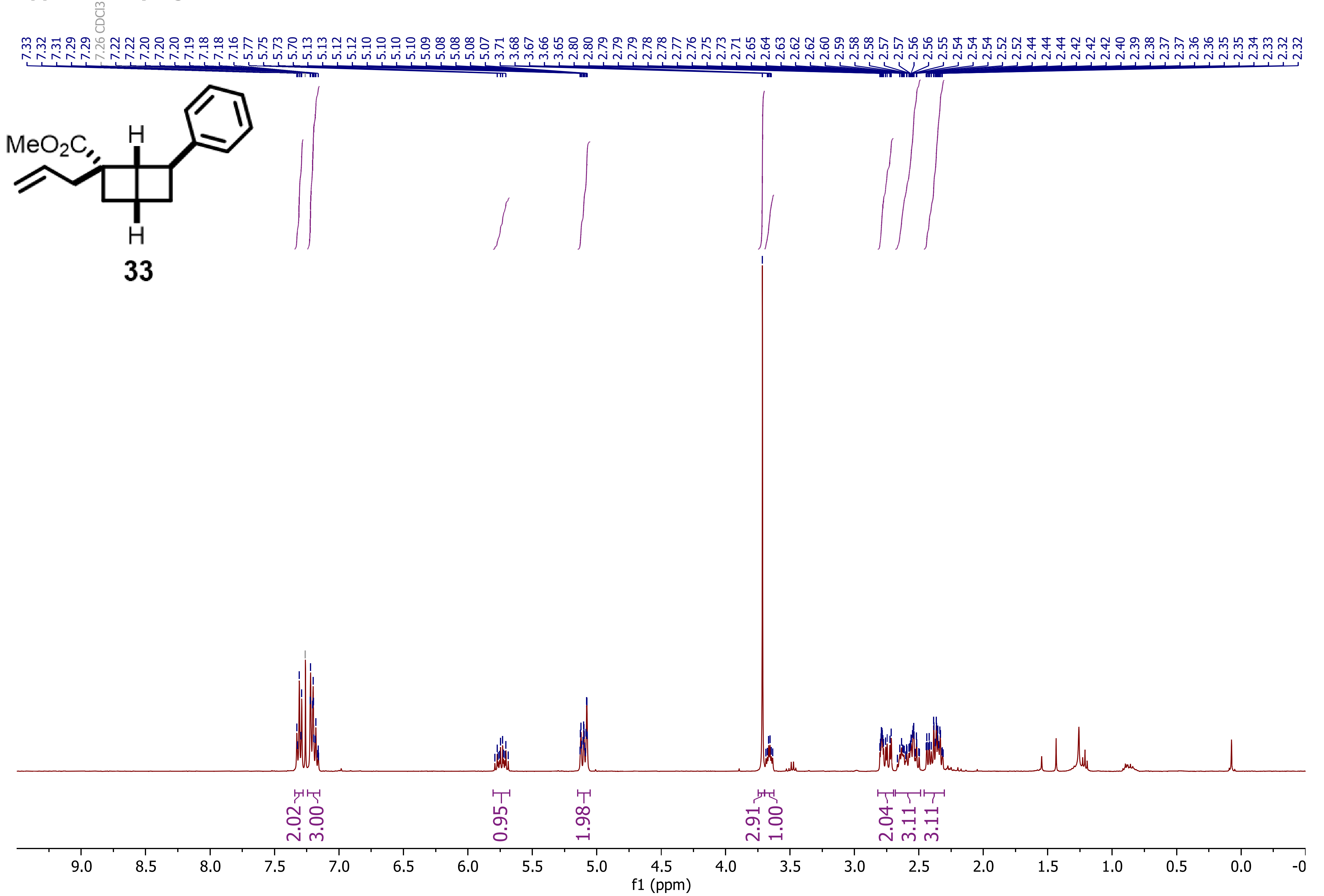

Supplementary Figure 151. ^{13}C NMR spectrum of **33** (126 MHz, $CDCl_3$)

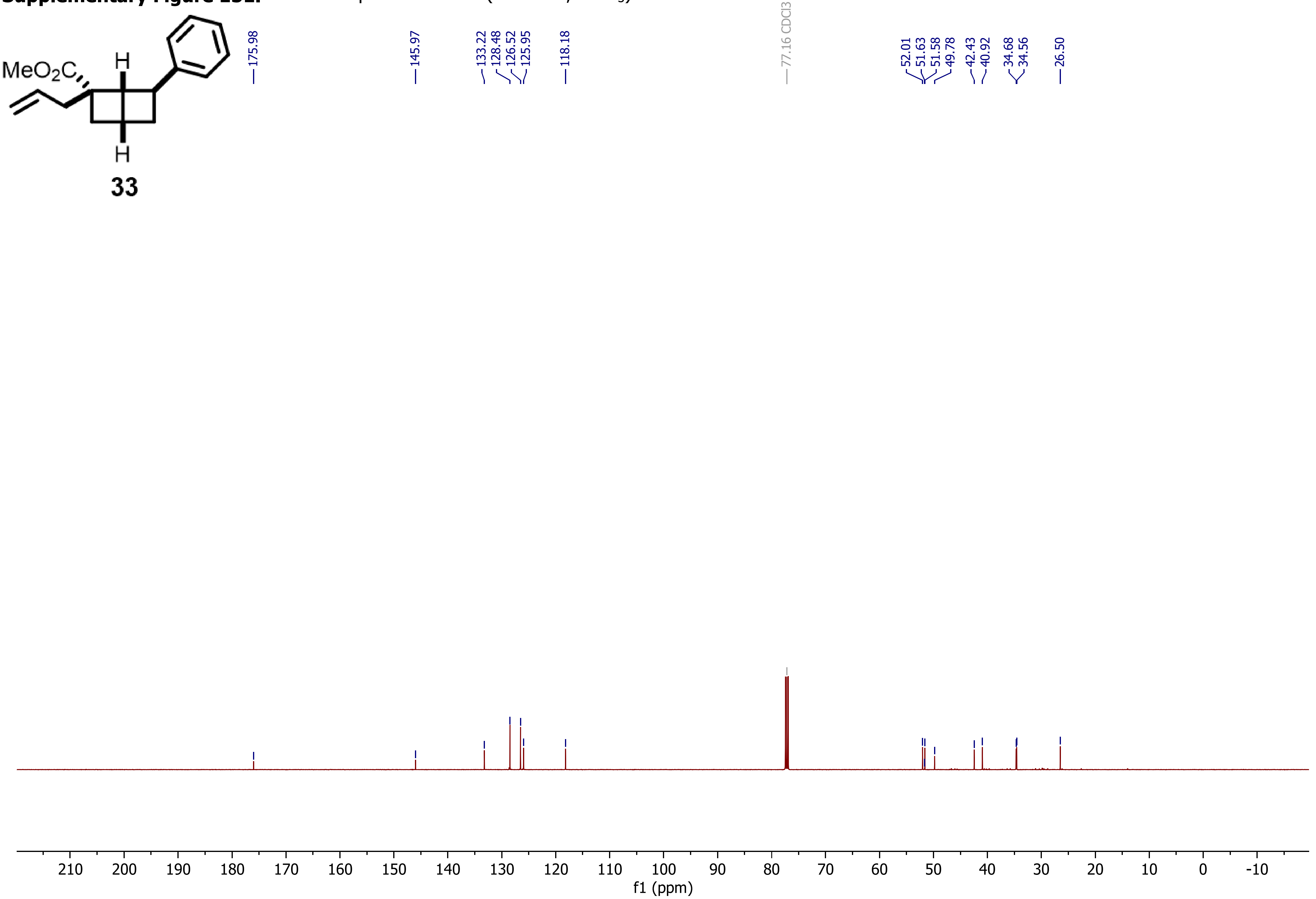

Supplementary Figure 128. ^{1}H NMR spectrum of **38** (500 MHz, $CDCl_3$)

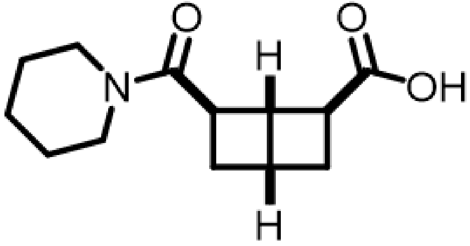

38

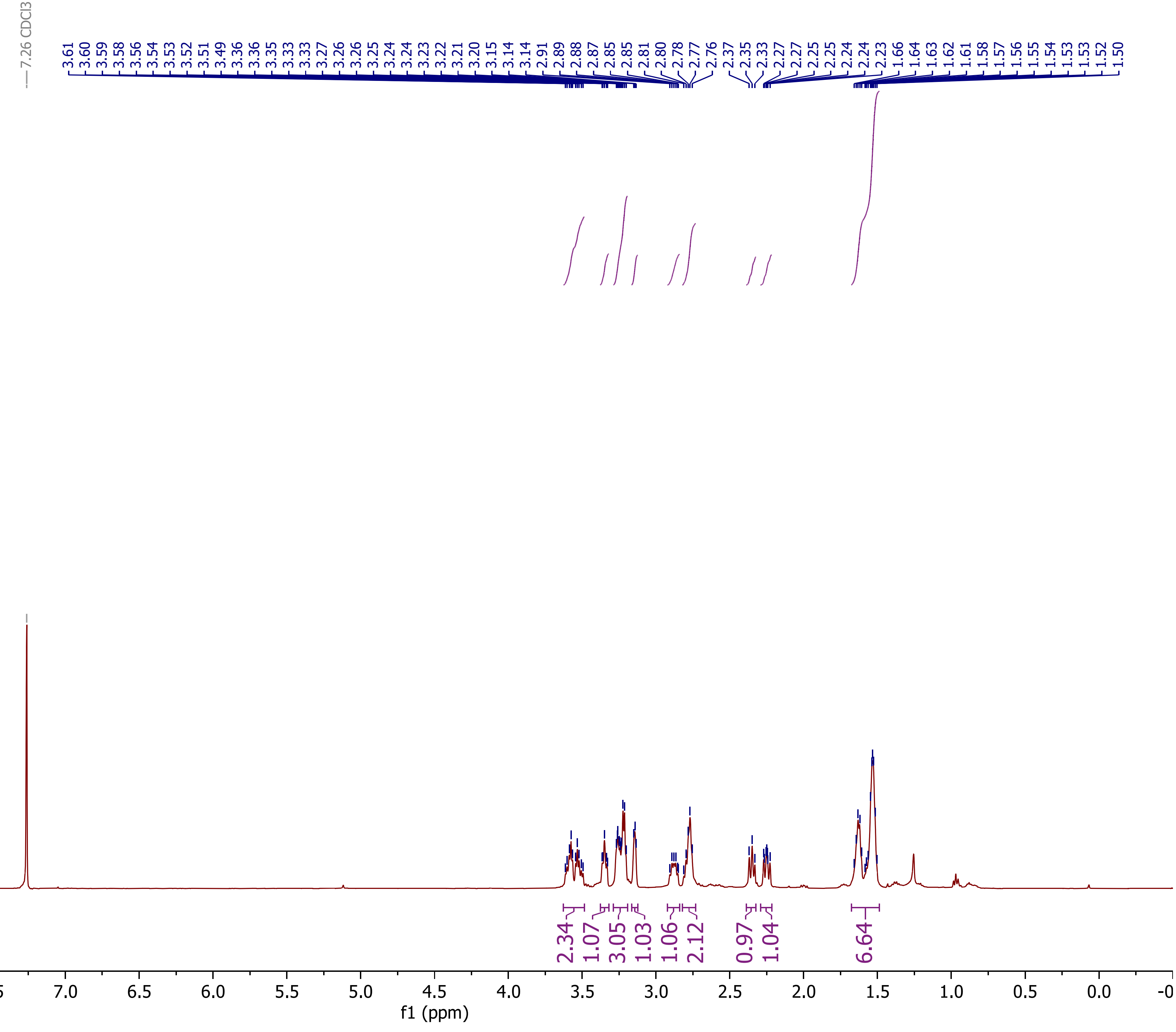

Supplementary Figure 129. ^{13}C NMR spectrum of **38** (126 MHz, $CDCl_3$)

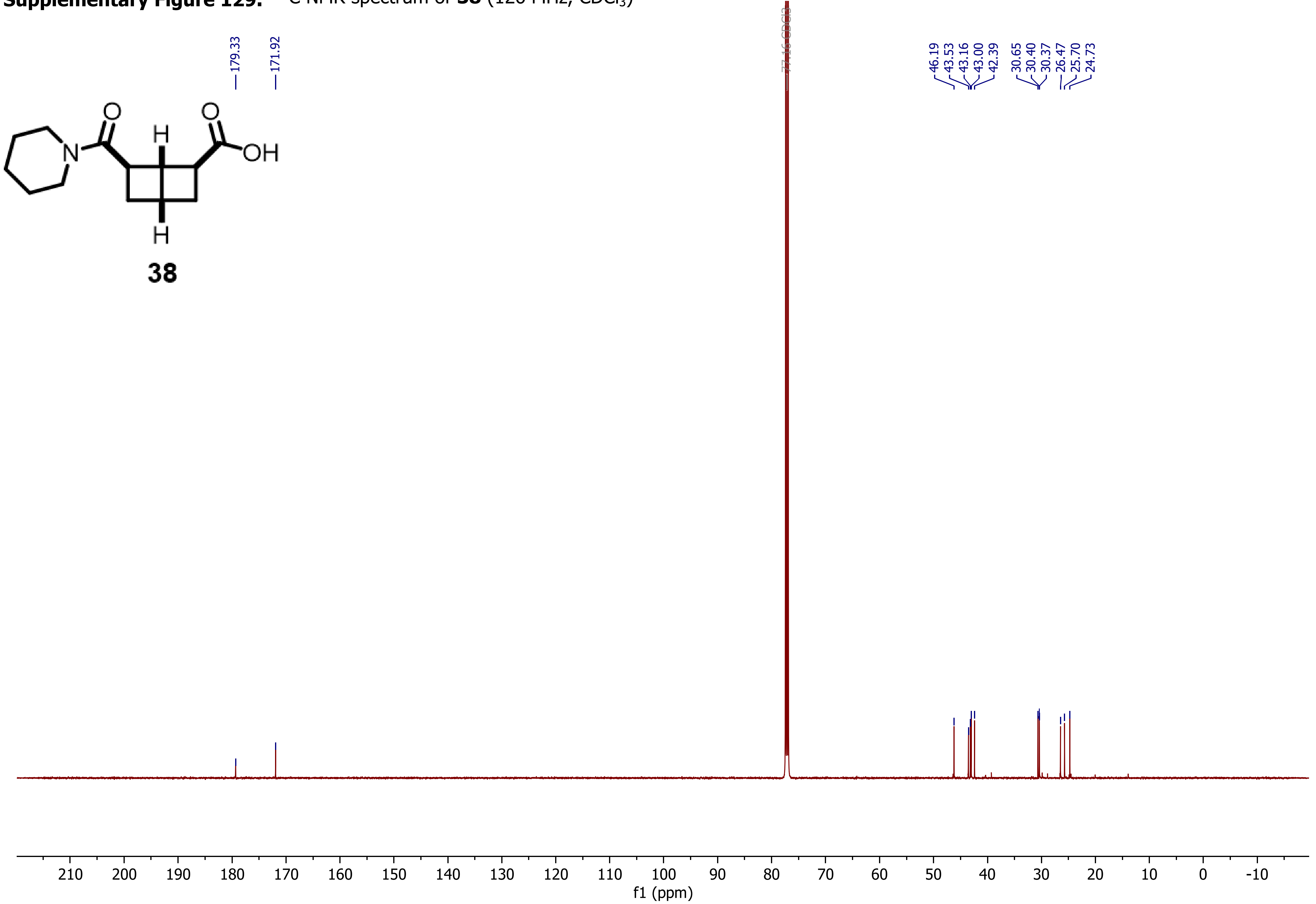

Supplementary Figure 130. ^{1}H NMR spectrum of **36** (500 MHz, $CDCl_3$)

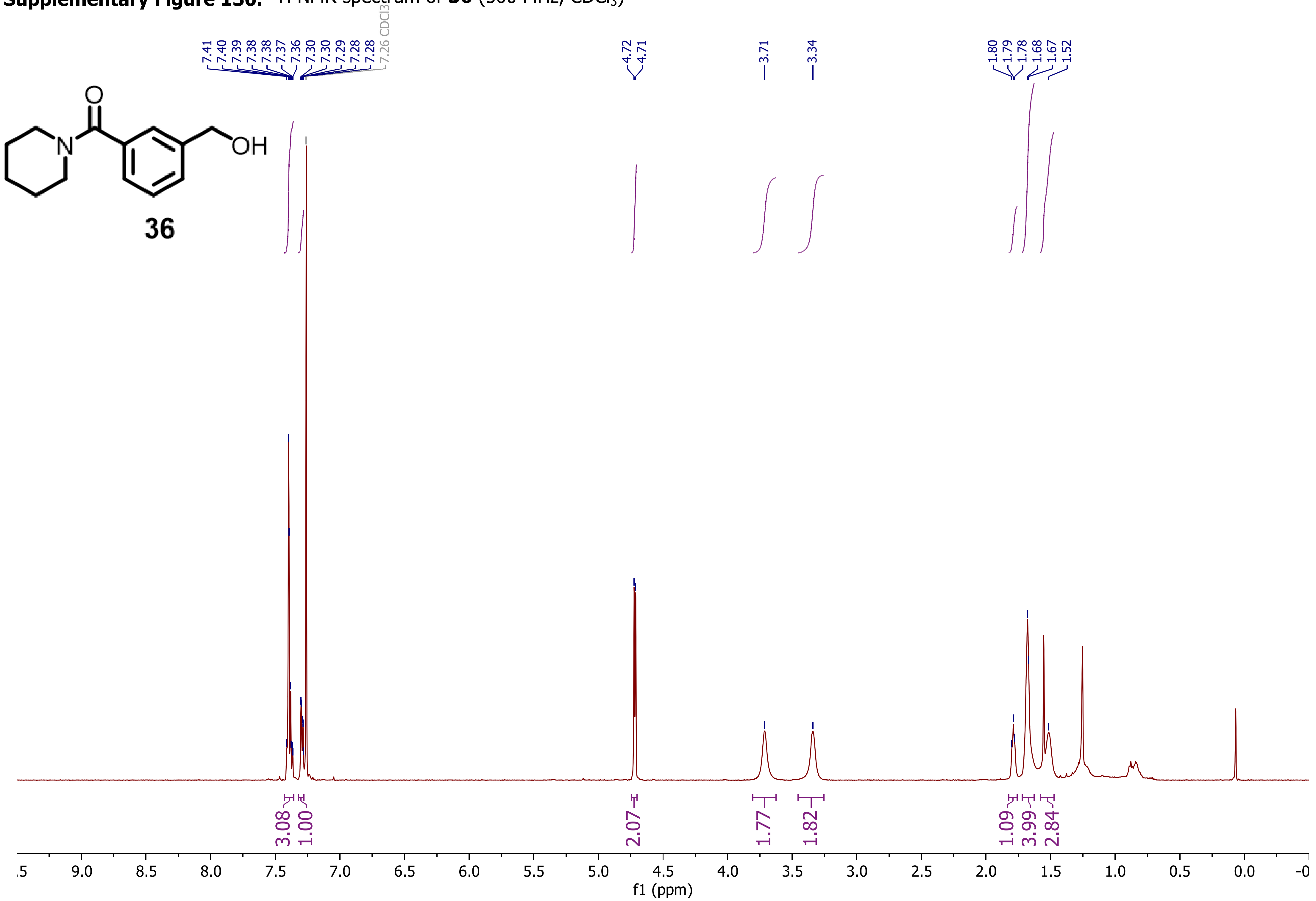

Supplementary Figure 131. ^{13}C NMR spectrum of **36** (126 MHz, $CDCl_3$)

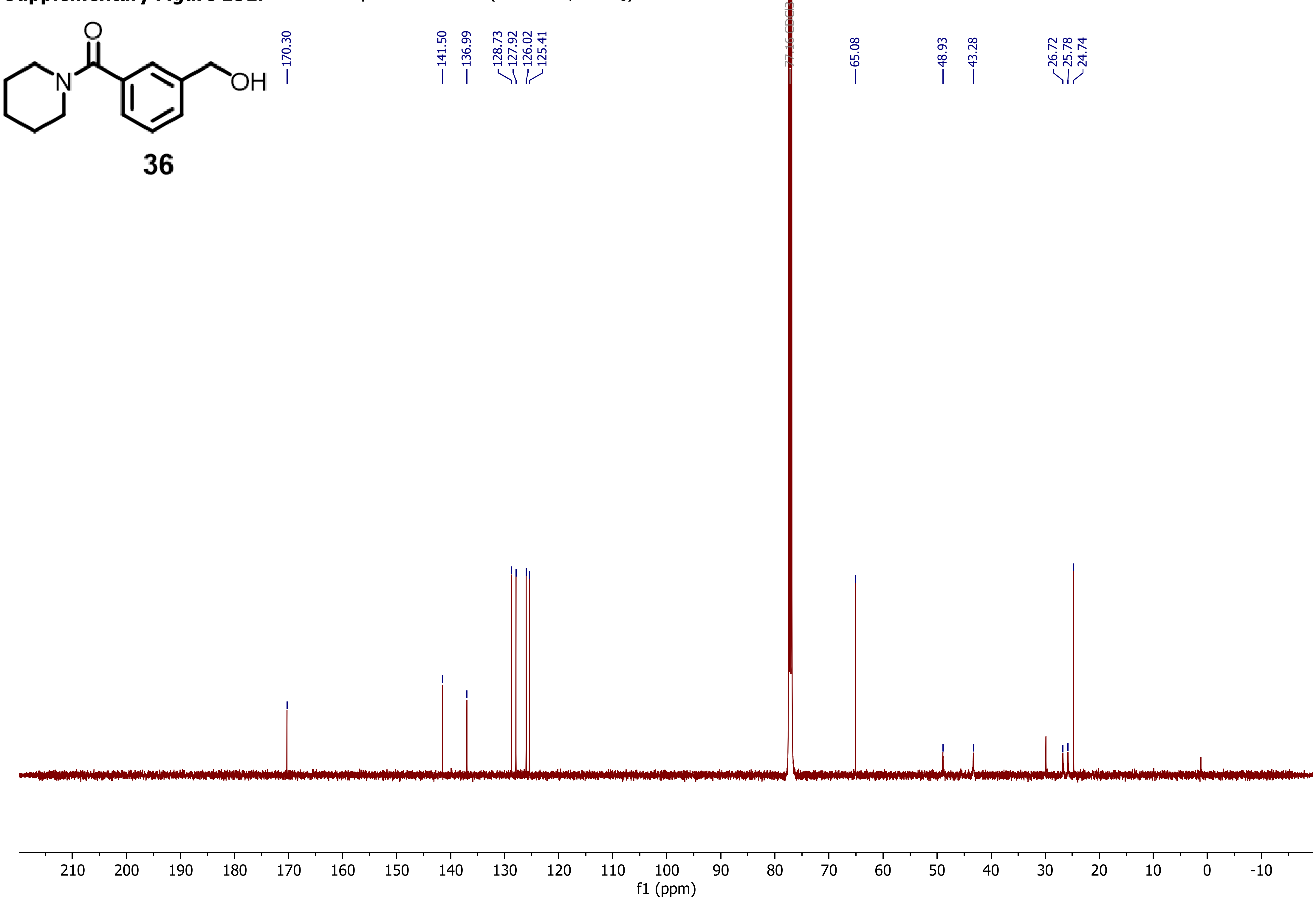

Supplementary Figure 132. ^{1}H NMR spectrum of **SI-47** (500 MHz, $CDCl_3$)

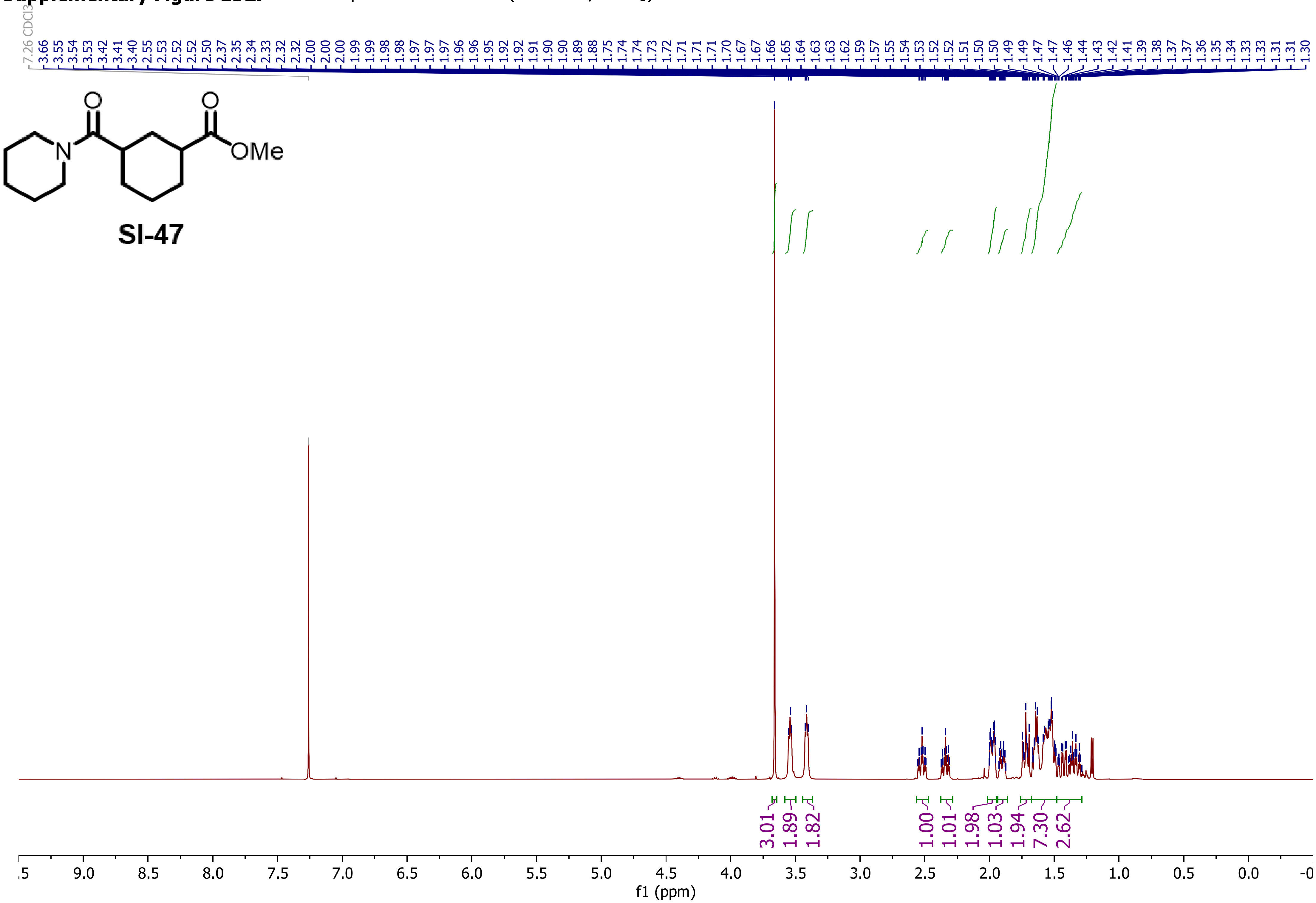

Supplementary Figure 133. ^{13}C NMR spectrum of **SI-47** (126 MHz, $CDCl_3$)

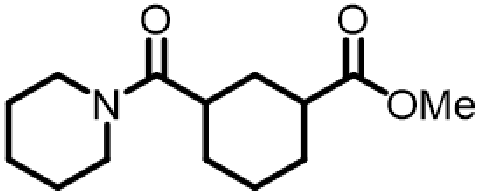

SI-47

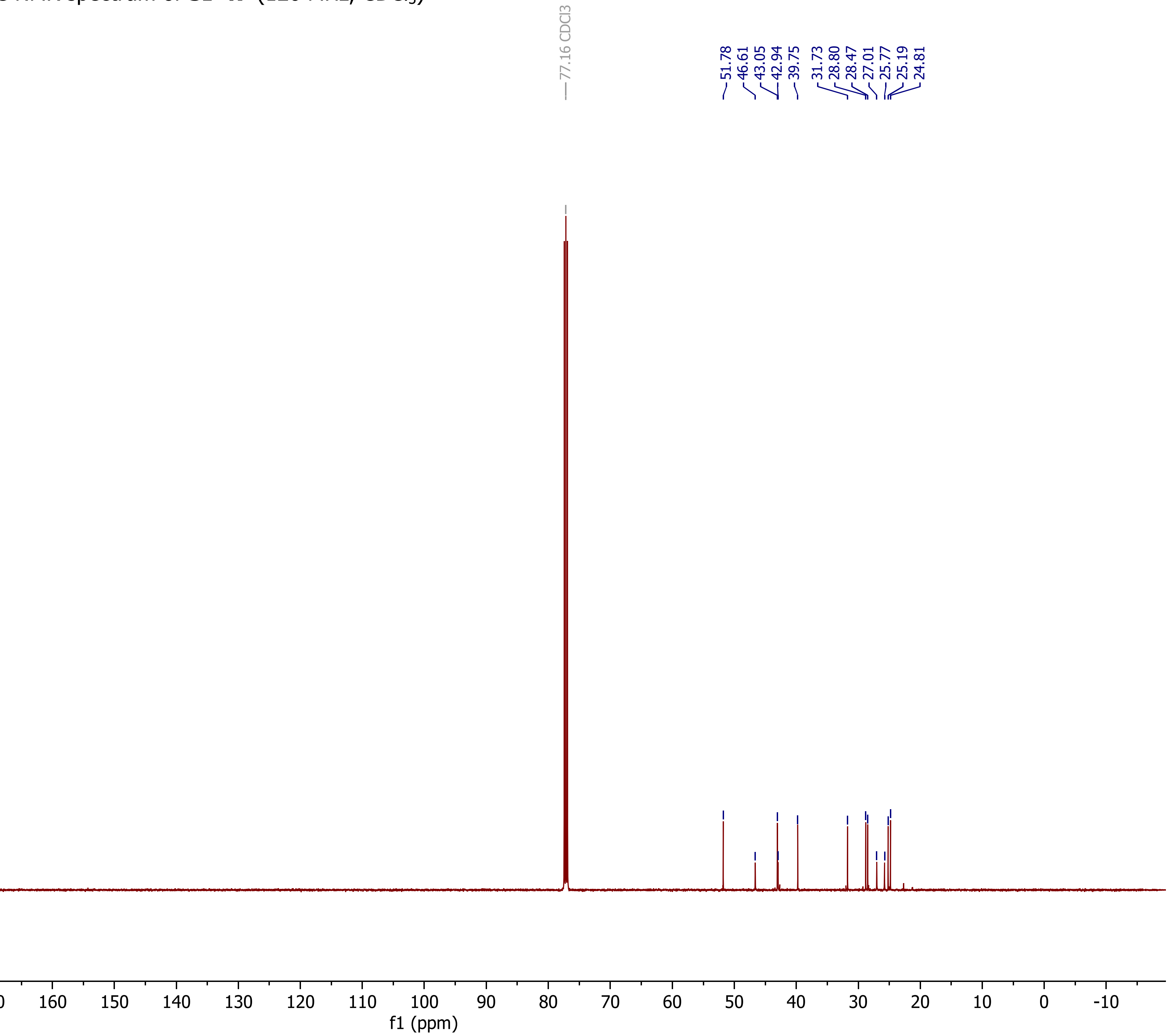

Supplementary Figure 134. ^{1}H NMR spectrum of **37** (500 MHz, $CDCl_3$)

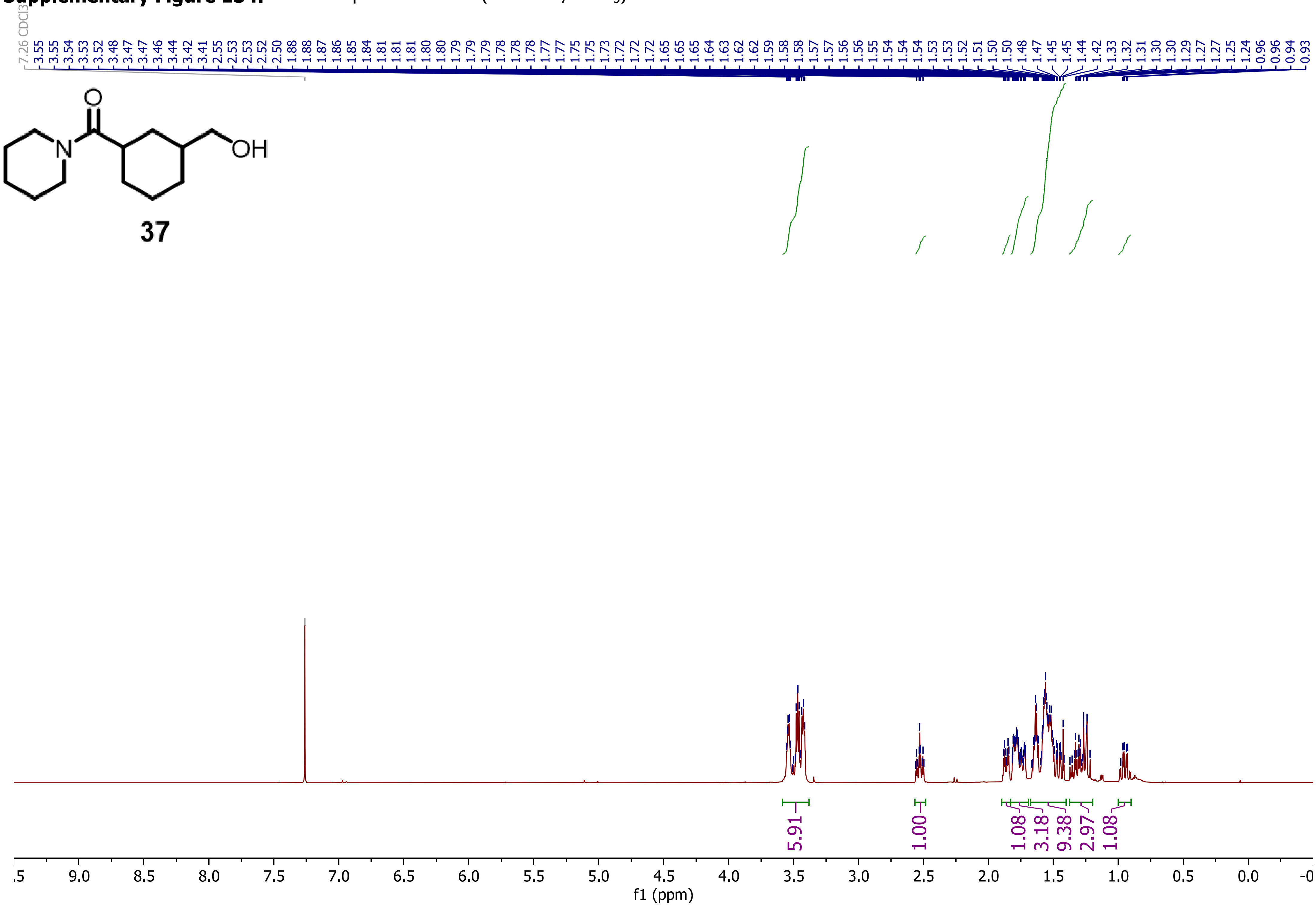

Supplementary Figure 135. ^{13}C NMR spectrum of **29** (126 MHz, $CDCl_3$)

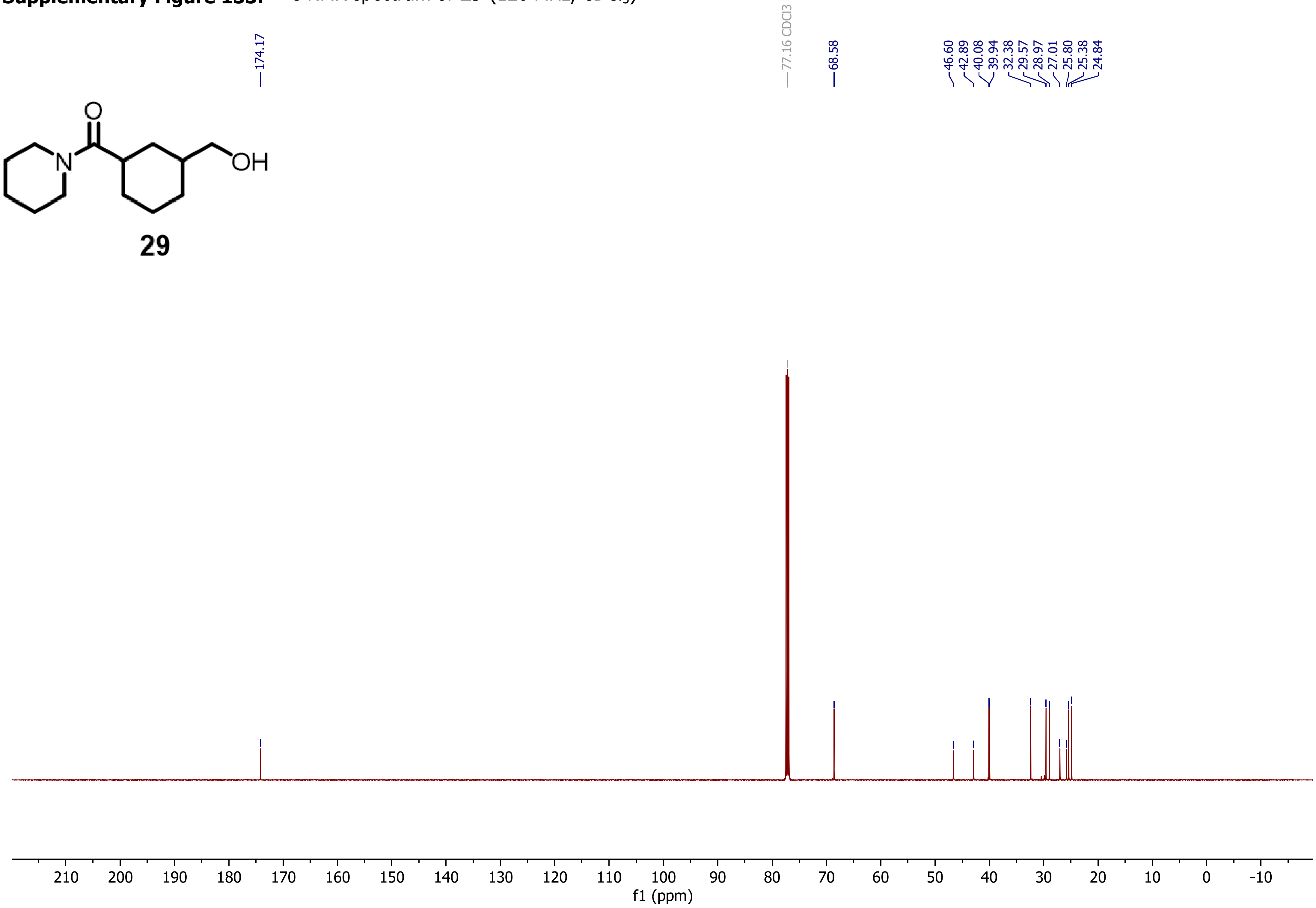

Supplementary Figure 136. ^{1}H NMR spectrum of **40** (500 MHz, $CDCl_3$)

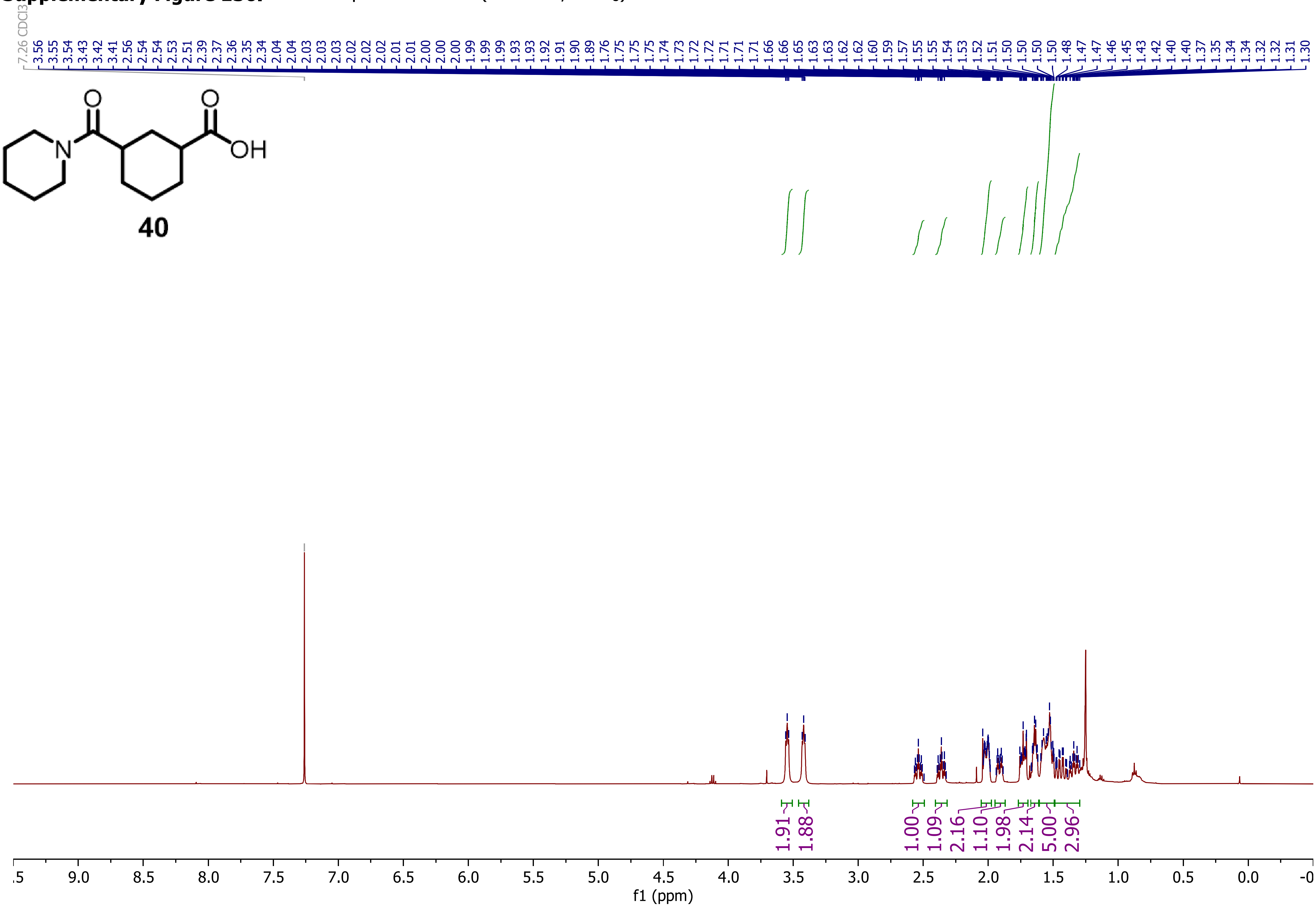

Supplementary Figure 137. ^{13}C NMR spectrum of **40** (126 MHz, $CDCl_3$)

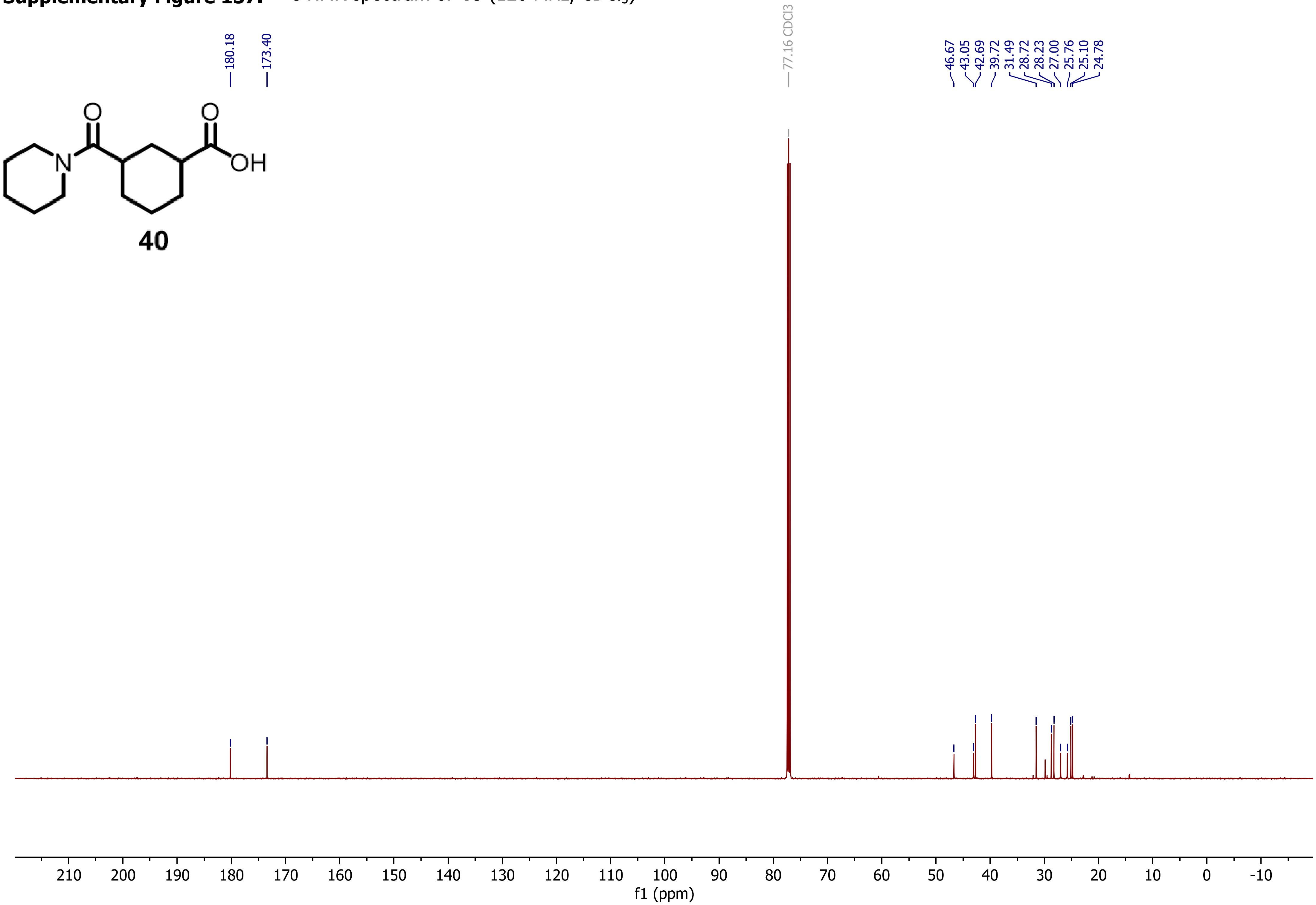

Supplementary References

(1) Love, B. E. & Jones, E. G. The use of salicylaldehyde phenylhydrazone as an indicator for the titration of organometallic reagents. *J. Org. Chem.* **64**, 3755–3756 (1999).

(2) Qin, T. et al. A general alkyl-alkyl cross-coupling enabled by redox-active esters and alkylzinc reagents. *Science* **352**, 801–805 (2016).

(3) Smith, J. M. et al. Decarboxylative alkynylation. *Angew. Chemie Int. Ed.* **56**, 11906–11910 (2017).

(4) Sandfort, F., O'Neill, M. J., Cornella, J., Wimmer, L., & Baran, P. S. Alkyl−(hetero)aryl bond formation via decarboxylative cross-coupling: a systematic analysis. *Angew. Chemie Int. Ed.* **56**, 3319–3323 (2017).

(5) Simonetti, S. O., Larghi, E. L., & Kaufman, T. S. Alternate and step-economic synthesis of the β-methylstyrene chelating pre-ligand of the Hoveyda-Grubbs' II catalyst. *Organic Preparations and Procedures International* **47**, 227–231 (2015).

(6) Richardson, T. I. et al. Synthesis and structure-activity relationships of novel arylpiperazines as potent and selective agonists of the melanocortin subtype-4 receptor. *J. Med. Chem.* **47**, 744–755 (2004).

(7) Smeenk, L. E. J., Dailly, N., Hiemstra, H., Van Maarseveen, J. H., & Timmerman, P. Synthesis of water-soluble scaffolds for peptide cyclization, labeling, and ligation. *Org. Lett.* **14**, 1194–1197 (2012).

(8) Elliott, L. D., Kayal, S., George, M. W., & Booker-Milburn, K. Rational design of triplet sensitizers for the transfer of excited state photochemistry from UV to visible. *J. Am. Chem. Soc.* **142**, 14947–14956 (2020).

(9) Clark, M. P. et al. Discovery of a novel, first-in-class, orally bioavailable azaindole inhibitor (VX-787) of influenza PB2. *J. Med. Chem.* **57**, 6668–6678 (2014).

(10) Nikiforov, P. O. et al. Fragment-sized EthR inhibitors exhibit exceptionally strong ethionamide boosting effect in whole-cell mycobacterium tuberculosis assays. *ACS Chem. Biol.* **12**, 1390–1396 (2017).

(11) Nunez, M. T. & Martin, V. S. Efficient oxidation of phenyl groups to carboxylic acids with ruthenium tetraoxide. A simple synthesis of (*R*)-7-caprolactone, the pheromone of trogoderma granarium. *J. Org. Chem.* **55**, 1928–1932 (1990).

(12) Zirimwabagabo, J. O. & Harrity, J. P. A. Pd-catalyzed kinetic resolution of cyclic enol ethers. An enantioselective route to functionalised pyrans. *Chem. Commun.* **50**, 2735–2737 (2014).

(13) Hancock, E. N., Kuker, E. L., Tantillo, D. J., & Brown, M. K. Lessons in strain and stability: enantioselective synthesis of (+)-[5]-ladderanoic acid. *Angew. Chemie Int. Ed.* **59**, 436–441 (2020).

(14) Fonsi, M., Orsale, M. V., & Monteagudo, E. High-throughput microsomal stability assay for screening new chemical entities in drug discovery. *J. Biomol. Screen.* **13**, 862–869 (2008).

(15) Zhou, L., Yang, L., Tilton, S., & Wang, J. Development of a high throughput equilibrium solubility assay using miniaturized shake-flask method in early drug discovery. *J. Pharm. Sci.* **96**, 3052–3071 (2007).

(16) Huth, F. et. al. Predicting oral absorption for compounds outside the rule of five property space. *J. Pharm. Sci.* **110**, 2562–2569 (2021).

(17) Levterov, V. V., Panasyuk, Y., Pivnytska, V. O., & Mykhailiuk, P. K. Water-soluble non-classical benzene mimetics. *Angew. Chemie Int. Ed.* **59**, 7161–7167 (2020).
